# Synthesis of γ-alkylidene lactones *via* molecular stitching of carboxylic acids and olefins†

**DOI:** 10.1039/d5sc03349g

**Published:** 2025-07-04

**Authors:** Edis Crnovrsanin, Sourjya Mal, Manuel van Gemmeren

**Affiliations:** a Otto Diels-Institut für Organische Chemie, Christian-Albrechts Universität zu Kiel Otto-Hahn-Platz 4 Kiel 24118 Germany vangemmeren@oc.uni-kiel.de

## Abstract

In this study, we describe a direct palladium-catalyzed tandem β-C(sp^3^)–H olefination/lactonization strategy for the one-step synthesis of γ-alkylidene lactones. The reaction is enabled by an *N*-acyl sulfonamide ligand and utilizes readily available and inexpensive carboxylic acids and styrenes as coupling partners, enabling the efficient construction of structurally diverse γ-alkylidene lactones through the consecutive functionalization of three C–H bonds. Notable highlights include excellent functional group tolerance and derivatization of the resulting γ-alkylidene lactones to obtain various synthetically useful motifs.

## Abstract 2

A palladium-catalyzed synthesis of γ-alkylidene lactones from free carboxylic acids and olefins is described. Enabled by an *N*-acyl sulfonamide (NASA) ligand three C–H bonds are functionalized in a β-C(sp^3^)–H olefination/lactonization sequence.

## Graphical abstract

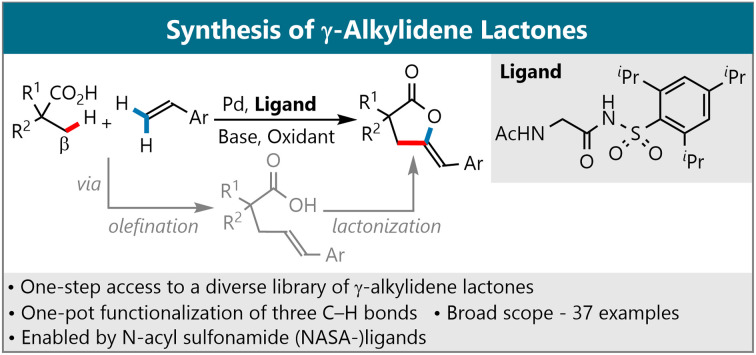

## Introduction

**Scheme 1:**
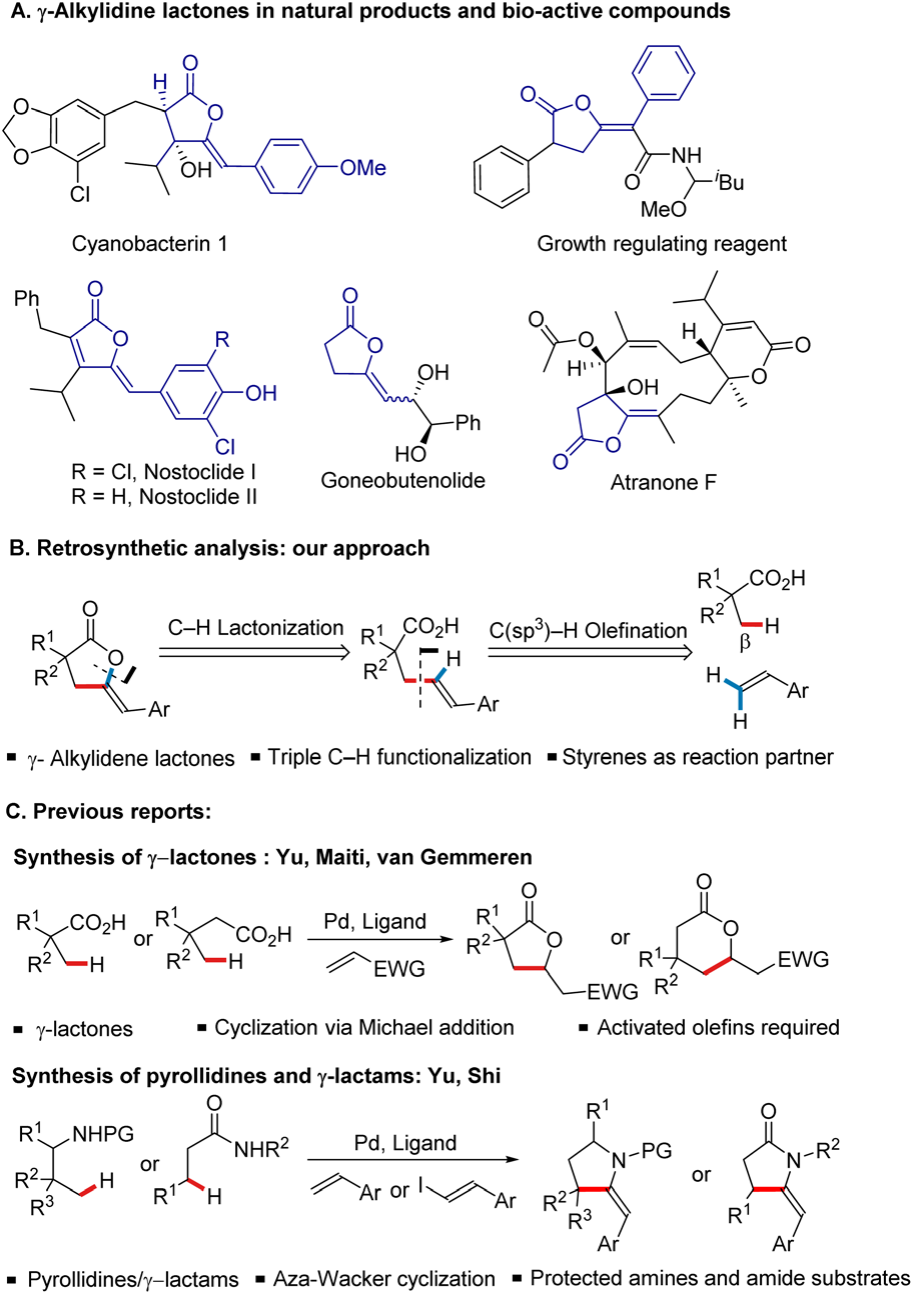
(A) Importance of γ-alkylidene lactones as bio-active compounds; (B) retrosynthetic analysis of γ-alkylidene lactones; (C) literature reports describing a different reactivity pattern between carboxylic acid and electron-poor olefins and an analogous reactivity pattern of protected amines and amides.

γ-Alkylidene lactones represent an important structural unit due to their widespread occurrence in various classes of natural products^1^ and diverse biological activities.^2^ Besides the great potential of these scaffolds to be used as a synthetic linchpin, their utilization in asymmetric synthesis has received considerable attention.^3^ Consequently, substantial efforts have been devoted towards their synthesis, which typically rely on Lewis acid-catalyzed cyclization reactions of acetylenic acids.^4^ However, the multistep synthetic sequences required for the preparation of the acetylenic acid precursors limit their broader application. A more general and practical approach would be to access γ-alkylidene lactones in one step from the commercially available feedstock chemicals, enabling an atom- and step-economic synthesis. Based on our previous experience in the area of carboxylic acid directed C(sp^3^)–H activation reactions,^5^ we envisioned that they could, in principle, be made by molecular stitching^6^ of olefins and carboxylic acids *via* a triple C–H functionalization strategy (Scheme 1B), which could be achieved by a sequence of carboxylic acid directed β-C(sp^3^)–H activation/olefination and subsequent cyclization *via* carboxylic acid directed vinylic C(sp^2^)–H activation/lactonization. Notably, this strategy differs fundamentally from the previously reported reactivity of carboxylic acids with activated olefins (*i.e.* acrylates, vinyl sulfones, vinyl phosphonates, *etc.*) and allylic electrophiles, in which the initially formed olefinated acid tends to undergo subsequent cyclization *via* intramolecular Michael addition, resulting in the formation of γ-lactones (Scheme 1C).^7^ Further motivation to pursue this strategy came from the description of a somewhat analogous reactivity with amides and protected amines^8^ as substrates (Scheme 1C).^9^ Therefore, building upon our recent contributions to carbon–heteroatom bond forming reactions,^10^ we herein report the development of a protocol for the direct synthesis of a wide range of γ-alkylidene lactones from readily available and inexpensive aliphatic carboxylic acids and styrenes *via* the one-pot functionalization of three C–H bonds.

## Results and discussion

**Scheme 2:**
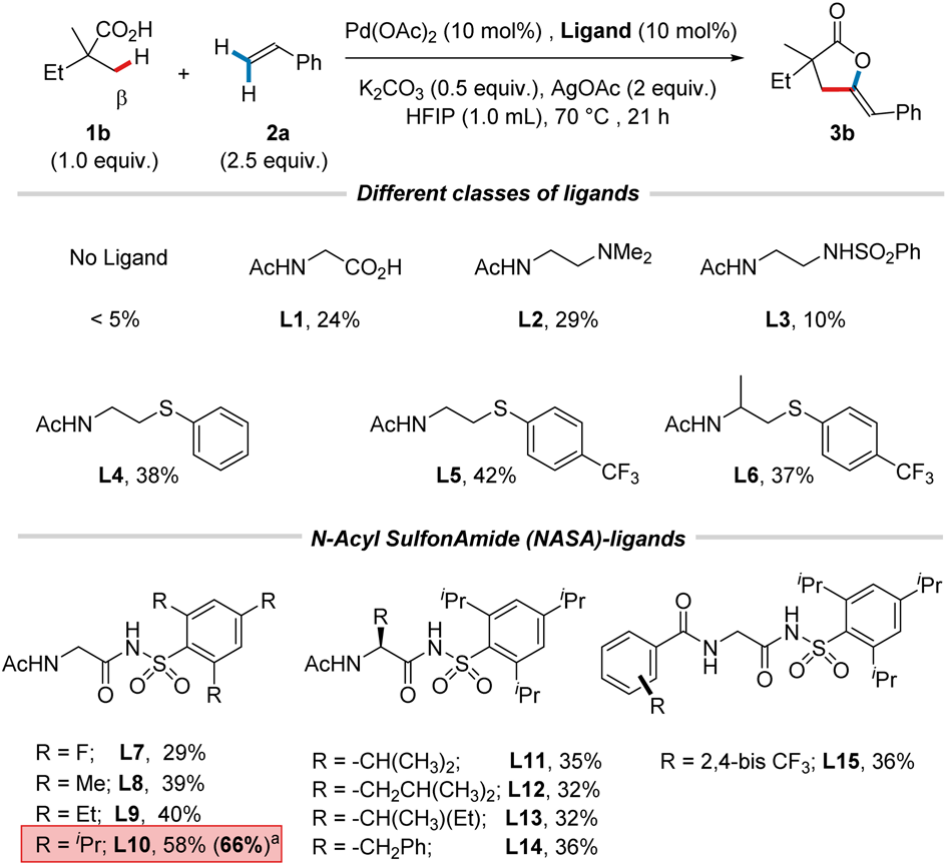
Optimization of the reaction conditions. Reactions were conducted on a 0.1 mmol scale. Yields were determined by ^1^H NMR analysis of the crude reaction mixture using CH_2_Br_2_ as an internal standard. ^a^ MnO_2_ (20 mol%) was added.

We commenced our studies using 2,2-dimethyl butyric acid (1b) as a model substrate and styrene (2a) as a coupling partner. Given the poor reactivity of styrenes in the analogous olefination reactions of free carboxylic acids,^7*a*–*d*^ we anticipated that the choice of a suitable ligand scaffold would be essential for promoting the particularly challenging carbopalladation step alongside other key steps of the catalytic cycle. Consequently, a comprehensive initial screening of ligands (see the ESI† for more details) led to the discovery of the *N*-acyl sulfonamide (NASA) ligand L10 as a particularly promising ligand, and we optimized the reaction conditions using this ligand. After identifying the best reaction conditions, we re-evaluated common ligand classes (Scheme 2). As expected, only traces of product (3b) were detected in the absence of ligand, indicating the crucial role of the external ligand in achieving the desired reactivity. We observed that amino acid-derived ligand (L1, 24%)^11^ and ethylenediamine-based ligands^12^ (L2, 29% and L3, 10%) continued to deliver unsatisfying results. Next, a series of *N*-acetyl thioether ligands,^7*a*^ the second-best ligand class in the initial screening, was tested (L4, 38%; L5, 42%; and L6, 37%), delivering improved results, albeit with still moderate yields. Due to the well-known isosteric relationship between the sulfonamide moiety and carboxylic acid,^13^ we hypothesized that replacing the carboxylic acid donor with a sulfonamide would not only allow for fine-tuning of the p*K*_a_ of the donor group, but also enable the introduction of steric bulk near the donor atom. Taking this into consideration and prompted by our recent study in which NASA-ligands were shown to be highly effective for Pd-catalyzed C(sp^2^)–H deuteration^14^ and the success of these ligands in challenging C(sp^3^)–H functionalizations,^15^ a range of NASA-ligands with electronically different substituents on the phenyl ring was examined. While electron-withdrawing substituents on the phenyl ring (L7, 29%) continued to offer moderate yield, electron-donating substituents (L8, 39% and L9, 40%) improved the reaction outcome. The catalytic efficiency was strongly enhanced by the introduction of a 2,4,6-triisopropyl group (L10, 58%), presumably due to the changes in the sulfonamide geometry caused by the steric bulk. Various substituents on the backbone (L11–L14) resulted in decreased yields. Furthermore, switching to a different concerted metalation–deprotonation (CMD)-promoting amide group (L15, 36%) gave no further improvement. Lastly, a series of co-oxidants^16^ was tested (see the ESI† for more details) in combination with the optimal ligand L10. Gratifyingly, the addition of MnO_2_ (20 mol%) proved beneficial, affording 3b in 66% yield.

Having identified suitable reaction conditions, we first explored the scope of aliphatic acid substrates. As expected from the optimization studies, the desired lactone derived from model substrate 1b was obtained in 65% (3b : 3b′ = 10 : 1, Scheme 3) isolated yield. Aliphatic acids with varying chain lengths and branching patterns on the side chain afforded their respective γ-alkylidene lactones in satisfactory yields, including methyl (45%, 3a : 3a′ = 11 : 1), *n*-butyl (47%, 3c : 3c′ = 6 : 1), and isobutyl (3d, 50%) substituted products. Substrates bearing a five- (47%, 3e : 3e′ = 20 : 1) or six- (3f, 52%) membered ring in the side chain were well-tolerated. Interestingly, 1g, which typically tends to undergo dehydrogenation *via* functionalization of methylene C(sp^3^)–H bonds,^17^ afforded 3g (70%) in excellent yield. Additionally, substrates having halogens (3h, 56% and 3i, 44%), trifluoromethyl (3j, 42%), or a protected hydroxyl group (3k, 35%) on the side chain were well-tolerated by our protocol. We then tested 4-aryl-substituted aliphatic acids featuring reactive benzylic C(sp^3^)–H bonds with electronically diverse arenes. While 1l bearing simple phenyl afforded the respective lactone in 50% (3l : 3l′ = 10 : 1) yield, electron-deficient arene improved the yield to 60% (3m : 3m′ = 20 : 1), implying that the reaction is sensitive to the electronic properties of the aryl group at this position. Next, we evaluated a series of 3-aryl-substituted aliphatic acids featuring equidistant benzylic C–H bonds, the activation of which would trigger side reactions/decomposition pathways, rendering these substrates particularly challenging. Notably, besides the simple phenyl group (3n, 40%), a range of synthetically useful functional groups, including an ester (3o, 41%), a nitro (3p, 43%), a trifluoromethylthio (3q, 44%), and a trifluoromethyl (3r, 45%) group, were found to be amenable with our protocol. Furthermore, phenyl acetic acid-derived 1s, in which *ortho* C(sp^2^)–H activation/functionalization competes with the desired reactivity,^18^ afforded 3s, albeit with moderate yield (34%). Our method was then applied on substrates bearing a single methyl group, which exhibited the desired reactivity, giving the corresponding lactones in 47% (3t : 3t′ = 11 : 1) and 50% (3u) yield, respectively. Additionally, substrate 1v, bearing a single methyl group and featuring reactive benzylic C(sp^3^)–H bonds, afforded the desired lactone in satisfactory yield (3v, 40%). Finally, we tested our method on gemfibrozil, an oral lipid-lowering drug, and obtained 3w in 53% yield. These results underscore the applicability of our protocol across a wide range of aliphatic carboxylic acid substrates. Next, we proceeded to study the scope with respect to the olefinic reaction partner. Styrenes with halogens at *ortho* (3x, 52% and 3y, 52%), *meta* (3z, 52% and 3aa, 48%), and *para* positions [3ab, 54%; 52% (3ac : 3ac′ = 12 : 1); and 3ad, 32%] underwent smooth product formation. A series of styrenes featuring various substituents at the *para* position was examined next. Notably, a broad range of synthetically useful functional groups, such as trifluoromethyl (61%, 3ae : 3ae′ = 3 : 1), ester (62%, 3af 47% + 3af′ 15%), nitro (61%, 3ag 51% + 3ag′ 10%), and protected amine (57%, 3ah : 3ah′ = 4 : 1) were found to be well tolerated, affording their respective lactones in moderate to good yields. Additionally, *meta*-substituted styrenes, with an aldehyde (53%, 3ai : 3ai′ = 1.6 : 1) or protected hydroxyl (3aj, 43%) group, as well as an *ortho*-substituted styrene (3ak, 52%), were found to be compatible with our protocol. Notably, since an excess of styrene is employed in our reaction, the presence of Lewis basic heteroatom-containing functional groups on the styrene could, in principle, be detrimental to the reaction outcome by acting as catalyst poisons. The successful application of our method to such cases thus demonstrates the versatility and robustness of our method. It is worth highlighting that although lactone 3 was exclusively formed in most cases, electron-deficient styrenes led to a relatively higher ratio of 3′ to 3 (see the ESI† for more details).

**Scheme 3:**
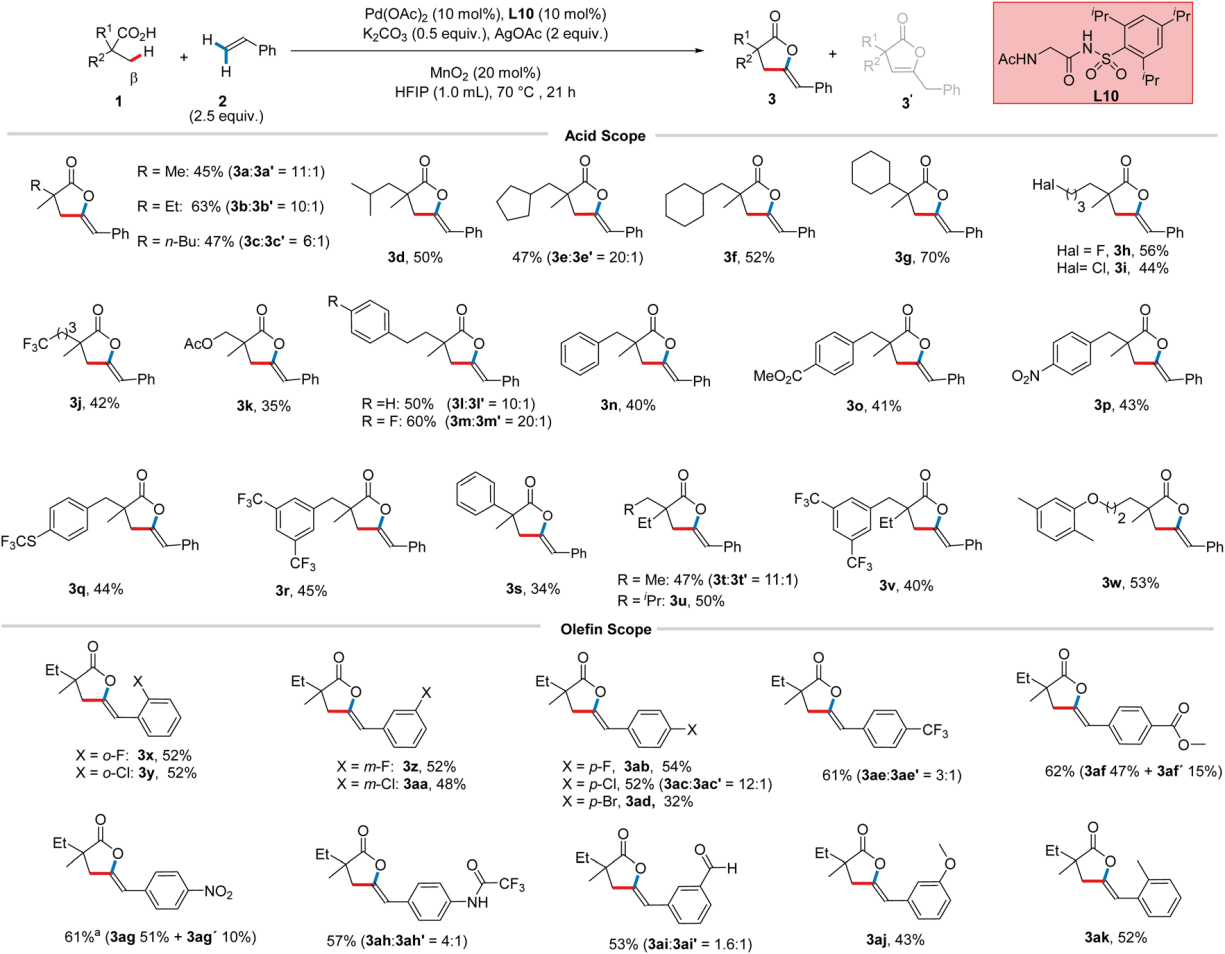
Scope studies. Reactions were performed on a 0.2 mmol scale. ^a^L6 was used instead of L10. The ratio of 3 to 3′ was determined using ^1^H NMR analysis of the isolated mixture. 3af′ and 3ag′ were isolated separately from their respective major isomers.

**Scheme 4:**
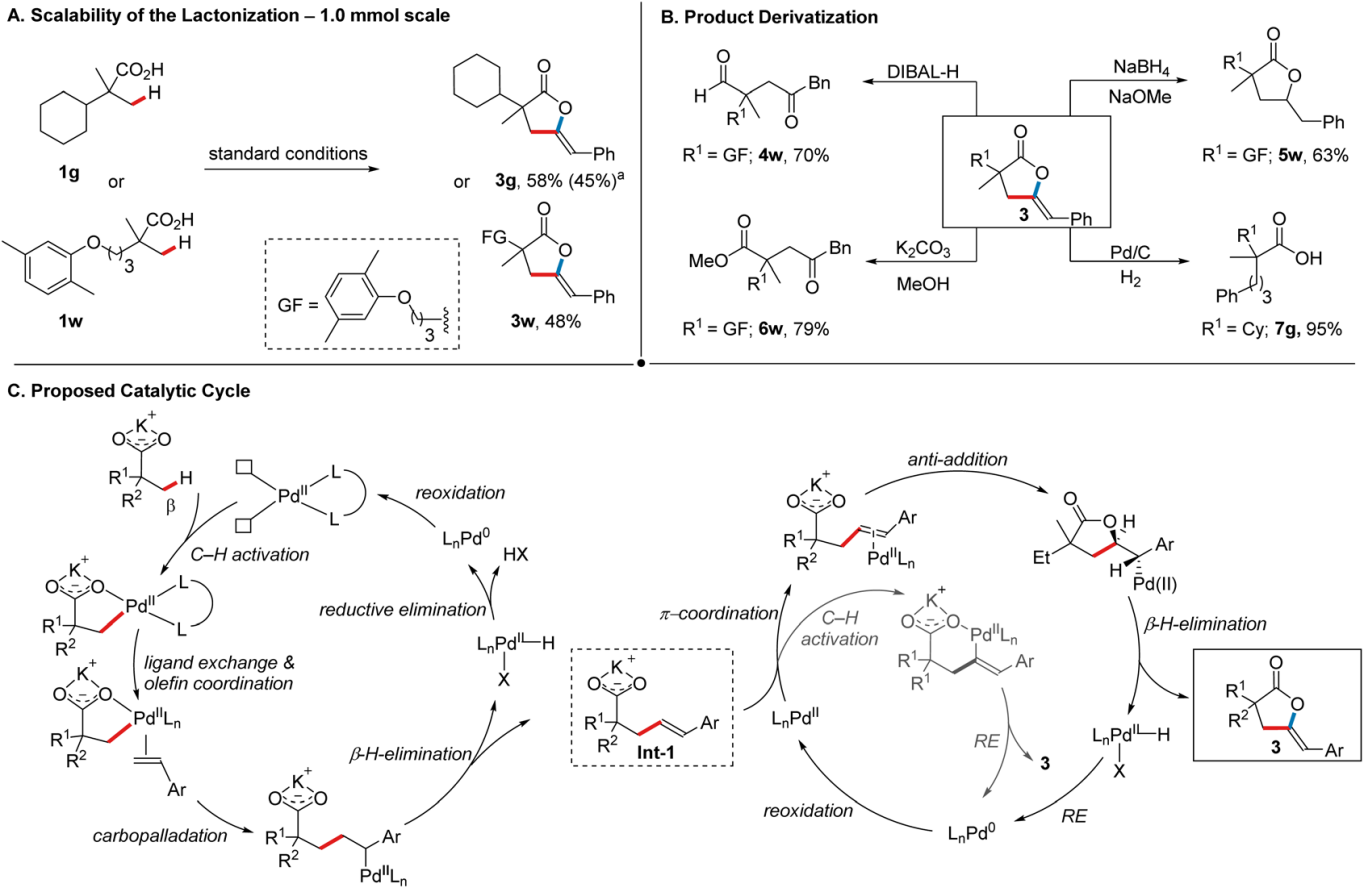
Representative synthetic applications and proposed catalytic cycle. (A) Scalability of the lactonization; (B) derivatization of the γ-alkylidene lactones; (C) proposed catalytic cycle.^a^ The reaction was performed on a 5.0 mmol scale.

In order to further showcase the synthetic utility of our protocol, we began by testing the scalability of the process. Two representative products, 3g (58%) and 3w (48%) could be obtained in synthetically useful yields on a 1.0 mmol scale (Scheme 4A). Furthermore, scaling up the reaction to 5.0 mmol delivered 3g (45%) with a slightly reduced yet satisfactory yield, even without extensive re-optimization of the reaction conditions. Next, we investigated possible derivatization reactions of the γ-alkylidene lactones. Given the importance of 1,4-dicarbonyl motifs across various fields and the challenges associated with their synthesis,^19^ we evaluated the utility of γ-alkylidene lactones in this context. First, the reduction of gemfibrozil-derived lactone 3w with DIBAL-H delivered 1,4-keto aldehyde 4w in good yield (70%, Scheme 4B), while methanolysis of 3w delivered 1,4-keto ester 6w (79%). Next, selective reduction of the exocyclic double bond in 3w using NaBH_4_ in the presence of catalytic NaOMe afforded 5w (63%), thus offering another avenue for the synthesis of bio-active lactones.^20^ The treatment of 3g with 1 atm H_2_ in the presence of Pd/C resulted in the formation of 7g (95%). Compounds such as 7g could, for example, be further derivatized into benzosuberone analogues *via* an intramolecular Friedel–Crafts acylation, highlighting the potential of our method in the generation of compound libraries.^21^

Based on literature precedence,^7*b*,*c*,22^ we propose that our transformation proceeds *via* a Pd^II^/Pd^0^ catalytic cycle, as depicted in Scheme 4C. First, the coordination of an external ligand to Pd(OAc)_2_ generates the active catalyst. The subsequent β-C(sp^3^)–H activation step is accelerated by both the counter-cation (K^+^) and the ligand, occurring through a CMD mechanism.^22*a*^ Following this, a sequence of ligand exchange, carbopalladation, β-hydride elimination, and decoordination results in the formation of C–H olefination product Int-1.^7*b*,*c*,22*b*^ The product decoordination concomitantly gives a Pd^II^–hydride species that can undergo a reductive elimination (RE) giving a Pd^0^ species, which is then re-oxidized by the terminal oxidants employed in the reaction. From this stage, there are two plausible pathways that lead to the product formation: Int-1 could undergo a sequence of oxypalladation followed by β-H elimination to form 3. In this sequence, anti-oxypalladation would be favored over a *syn*-pathway, as indicated by the observed *Z* stereochemistry in 3.^22*b*,*c*^ Alternatively, Int-1 could undergo a carboxylic acid-directed vinylic C(sp^2^)–H activation^7*e*,23^ and subsequent C–O RE to furnish 3. Both of these pathways result in the concomitant formation of Pd^0^, which is subsequently re-oxidized. Given that the olefination and cyclization steps collectively require the removal of four electrons from Pd by oxidant(s), either four equivalents of Ag(i) or a combination of silver salt, MnO_2_, and air are required, with the latter demonstrating superior efficiency in our case. Based on the mechanistic considerations regarding the cyclization step, we conducted a series of experiments. When independently synthesized Int-1 was subjected to the standard reaction conditions, 3 was obtained in 38% NMR-yield (see the ESI† for more details). The *Z*-isomer of Int-1, in contrast, gave *E*-configured 3 in 48% NMR-yield when subjected to the standard reaction conditions. These results indicate that Int-1 is an intermediate in our reaction and confirm a stereospecific pathway from Int-1 to the final product. Based on the current data and literature reports, neither of the proposed pathways for the final cyclization step can be fully excluded. However, considering the relative ease with which such an olefinated intermediate is expected to undergo palladium-mediated intramolecular attack by a nucleophilic functional group to form a kinetically favored five-membered ring^22*c*,*d*^ and the relatively unfavorable formation of a thermodynamically labile six-membered palladacycle *via* C–H activation,^24^ the oxypalladation/β-H elimination pathway appears to be the more plausible scenario (for a discussion of possible pathways leading to the formation of side product 3′; see the ESI†).

## Conclusions

In summary, we have developed a Pd-catalyzed triple C–H functionalization protocol for the synthesis of γ-alkylidene lactones by molecular stitching of readily available carboxylic acids and styrenes. The reaction is enabled by the use of an *N*-acyl sulfonamide (NASA)-ligand and exhibits excellent functional group tolerance, allowing for the efficient one-step synthesis of structurally diverse γ-alkylidene lactones. Our study demonstrates the scalability of the protocol, a number of synthetically valuable derivatizations of the obtained products, and provides mechanistic insights. This novel access to γ-alkylidene lactones is expected to prove highly useful in various disciplines. Additionally, the superior performance of *N*-acyl sulfonamide (NASA-) ligands in this transformation is expected to trigger further research toward the utilization of this promising ligand class.

## Author contributions

EC and SM: methodology, investigation, writing – original draft, review & editing. MvG: conceptualization, writing – original draft, review & editing, supervision, funding acquisition.

## Conflicts of interest

There are no conflicts to declare.

## Supplementary Material

SC-016-D5SC03349G-s001

## Data Availability

Full experimental results are available in the ESI.†

## References

[cit1] Negishi E.-i., Kotora M. (1997). Tetrahedron.

[cit2] Lee E.-S. J., Gleason F. K. (1994). Plant Sci..

[cit3] Ge Y., Han Z., Wang Z., Feng C.-G., Zhao Q., Lin G.-Q., Ding K. (2018). Angew. Chem., Int. Ed..

[cit4] Sayantika Bhakta M., Ghosh T. (2021). Asian J. Org. Chem..

[cit5] Uttry A., van Gemmeren M. (2018). Synlett.

[cit6] Tomanik M., Yu J.-Q. (2023). J. Am. Chem. Soc..

[cit7] Zhuang Z., Yu C.-B., Chen G., Wu Q.-F., Hsiao Y., Joe C. L., Qiao J. X., Poss M. A., Yu J.-Q. (2018). J. Am. Chem. Soc..

[cit8] Jiang H., He J., Liu T., Yu J.-Q. (2016). J. Am. Chem. Soc..

[cit9] Ding Y., Han Y.-Q., Wu L.-S., Zhou T., Yao Q.-J., Feng Y.-L., Li Y., Kong K.-X., Shi B.-F. (2020). Angew. Chem., Int. Ed..

[cit10] Mal S., Jurk F., Hiesinger K., van Gemmeren M. (2024). Nat. Synth..

[cit11] Engle K. M., Wang D.-H., Yu J.-Q. (2010). J. Am. Chem. Soc..

[cit12] Shen P.-X., Hu L., Shao Q., Hong K., Yu J.-Q. (2018). J. Am. Chem. Soc..

[cit13] Lassalas P., Gay B., Lasfargeas C., James M. J., Tran V., Vijayendran K. G., Brunden K. R., Kozlowski M. C., Thomas C. J., Smith III A. B., Huryn D. M., Ballatore C. (2016). J. Med. Chem..

[cit14] Farizyan M., Mondal A., Mal S., Deufel F., van Gemmeren M. (2021). J. Am. Chem. Soc..

[cit15] Strassfeld D. A., Chen C.-Y., Park H. S., Phan D. Q., Yu J.-Q. (2023). Nature.

[cit16] Janssen M., De Vos D. E. (2019). Chem.–Eur. J..

[cit17] Das J., Ali W., Ghosh A., Pal T., Mandal A., Teja C., Dutta S., Pothikumar R., Ge H., Zhang X., Maiti D. (2023). Nat. Chem..

[cit18] Wang D.-H., Mei T.-S., Yu J.-Q. (2008). J. Am. Chem. Soc..

[cit19] Piancatelli G., D'Auria M., D'Onofrio F. (1994). Synthesis.

[cit20] Pawar V. U., Ghosh S., Chopade B. A., Shinde V. S. (2010). Bioorg. Med. Chem. Lett..

[cit21] Tanpure R. P., George C. S., Strecker T. E., Devkota L., Tidmore J. K., Lin C.-M., Herdman C. A., MacDonough M. T., Sriram M., Chaplin D. J., Trawick M. L., Pinney K. G. (2013). Bioorg. Med. Chem..

[cit22] Engle K. M. (2016). Pure Appl. Chem..

[cit23] Giri R., Yu J.-Q. (2008). J. Am. Chem. Soc..

[cit24] Dolui P., Das J., Chandrashekar H. B., Anjana S. S., Maiti D. (2019). Angew. Chem., Int. Ed..

