# Supplementary material for: Synthesis of γ-alkylidene lactones via molecular stitching of carboxylic acids and olefins
Source: Chem Sci. 2025 Jul 4;16(32):14578–83. doi: 10.1039/d5sc03349g (PMC12257166; doi:10.1039/d5sc03349g)
Supplement: SC-016-D5SC03349G-s001 [file SC-016-D5SC03349G-s001.pdf]

Supplementary Information (SI) for Chemical Science.
This journal is © The Royal Society of Chemistry 2025

This version of the ESI published 14/07/2025 replaces the previous version published 04/07/2025. The product ratios for compounds 3c and 3t are corrected.

Synthesis of γ-Alkylidene Lactones via Molecular Stitching of Carboxylic Acids and Olefins

Edis Crnovrsanin,‡,[a] Sourjya Mal,‡,[a] and Manuel van Gemmeren[a],*

[a]Otto Diels-Institut für Organische Chemie, Christian-Albrechts Universität zu Kiel, Otto-Hahn-Platz 4, 24118 Kiel

[‡] These authors contributed equally to this work

1. General Information 2
2. Preparation of Ligands 4
3. Optimization of the Reaction Conditions 10
4. Synthesis of Carboxylic Acids and Styrenes 21
5. Scope Studies 22
6. Preparative Scale Applications and Derivatizations 45
7. Preliminary Mechanistic Investigations 51
8. References 57
9. NMR Data 58

1. General Information

Solvents, Reagents and Techniques

All reactions were conducted in oven-dried glassware (100 °C). Reaction temperatures refer to the temperature of the aluminum-block or oil bath surrounding the reaction vessel. Commercially available chemicals were obtained from ABCR, Acros Organics, BLD-pharm, Alfa Aesar, Deutero, Eurisotop, Fluorochem, Sigma Aldrich, or TCI Europe and used as received. HFIP was purchased from Fluorochem and used as received. Solvents used for column chromatography were distilled prior to use.

Chromatography

Analytical thin layer chromatography (TLC) was performed on silica gel ALUGRAM Xtra SIL G/UV254 plates (Macherey-Nagel) or aluminum oxide 150 F254, neutral plates (Merck). Compounds were visualized by ultraviolet light (254 nm or 366 nm) or by staining with $KMnO_4$ (1 g $KMnO_4$, 6 g K_2CO_3 and 0.1 g KOH in 100 mL of H_2O) or bromocresol green (40 mg bromocresol green in 100 mL EtOH; addition of $0.1M_{(aq.)}$ NaOH until the blue color appears in the solution) and developed with a heat gun if necessary. Flash chromatography was performed on silica gel 60M (0.04-0.063 mm) or aluminum oxide (aluminum oxide 90, neutral, activity level 1) with a positive nitrogen overpressure.

Nuclear Magnetic Resonance (NMR) Spectroscopy

^{1}H, ^{13}C, and ^{19}F NMR spectra were recorded at 25 °C on a Bruker AvanceNeo 500 or a Bruker Avance 600 device. Chemical shifts (δ) are given relative to tetramethylsilane (TMS) and using the residual solvent peaks for calibration. ^{19}F-NMR spectra are externally referenced with CCl_3F. Chemical shifts are reported with two decimal numbers (^{1}H) or one decimal number (^{13}C, ^{19}F). Data is reported in the following order: Chemical shift (multiplicity [s = singlet, d = doublet, t = triplet, q = quartet, quint = quintet, hept = septet, m = multiplet, br = broad signal], coupling constant (*J*, Hz) and integration). All NMR-spectra were processed using MestReNova.

Infrared spectroscopy (IR)

IR-spectroscopy was performed on a Perkin Elmer ATR spectrometer. Samples were measured neat. The wave numbers (ν) of recorded IR-signals are reported in cm^{-1}.

Mass Spectrometry (MS)

High resolution mass spectra (HRMS) were recorded on a Jeol AccuTOF (EI) or a ThermoFisher Orbitrap (ESI) device.

2. Preparation of Ligands

General Procedure A:

DMAP (2.0 equiv.) and EDC·HCl (1.5 equiv.) were dissolved in CH_2Cl_2 (3 mL/mmol) and stirred at room temperature until all the solids were dissolved. After cooling to 0 °C, the corresponding acid (1.0 equiv.) was added, followed by the corresponding sulfonamide (1.1 equiv.) and the mixture was stirred at rt for 21 h. The aqueous phase was acidified with 2M HCl to reach a pH value of 1 and extracted with EtOAc (3 × 60 mL). The combined organic phases were dried over $MgSO_4$, filtered, and concentrated under reduced pressure. The crude product was purified via flash column chromatography using CH_2Cl_2/MeOH/HCO_2H (97:2.5:0.5)

2-acetamido-N-((2,4,6-triethylphenyl)sulfonyl)acetamide

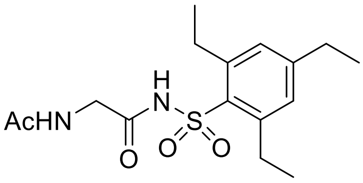

L9

Following the general procedure **A** using DMAP (623 mg, 5.12 mmol, 2.0 equiv.), EDC·HCl (737 mg, 3.84 mmol, 1.5 equiv.), N-Ac-Gly-OH (300 mg, 2.56 mmol, 1.0 equiv.), and 2,4,6-triethylbenzenesulfonamide (680 mg, 2.82 mmol, 1.1 equiv.) the target compound **L9** was obtained as a colorless solid (457 mg, 1.34 mmol, 52 %).

^{1}H-NMR (500 MHz, DMSO-d$_6$): δ = 12.27 (s, 1H), 8.05 (t, *J* = 5.9 Hz, 1H), 7.09 (s, 2H), 3.75 (d, *J* = 5.9 Hz, 2H), 3.04 (q, *J* = 7.4 Hz, 4H), 2.60 (q, *J* = 7.6 Hz, 2H), 1.79 (s, 3H), 1.21 – 1.17 (m, 9H) ppm.

^{13}C-NMR (151 MHz, DMSO-d$_6$): δ = 169.6, 168.5, 148.8, 145.7, 132.9, 129.1, 41.8, 27.6, 27.0, 22.2, 16.6, 14.8 ppm.

HRMS (ESIpos) m/z: Calcd for $C_{16}H_{24}N_2O_4SNa$ 363.1349, Found 363.1344.

IR (cm^{-1}): 3331, 2965, 1722, 1611, 1561, 1345, 1148.

2-acetamido-N-((2,4,6-triisopropylphenyl)sulfonyl)acetamide

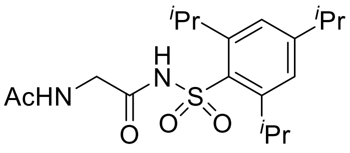

L10

Following the general procedure **A** using DMAP (853 mg, 6.99 mmol, 2.0 equiv.), EDC·HCl (1.00 g, 5.24 mmol, 1.5 equiv.), N-Ac-Gly-OH (409 mg, 3.49 mmol, 1.0 equiv.), and 2,4,6-triisopropylbenzenesulfonamide (1.00 g, 3.84 mmol, 1.1 equiv.) the target compound **L10** was obtained as a colorless solid (750 mg, 1.96 mmol, 62 %).

^{1}H-NMR (600 MHz, DMSO-d$_6$): δ = 12.35 (s, 1H), 8.08 (t, J = 5.8 Hz, 1H), 7.24 (s, 2H), 4.20 (hept, J = 6.7 Hz, 2H), 3.75 (d, J = 5.9 Hz, 2H), 2.91 (hept, J = 6.7 Hz, 1H), 1.79 (s, 3H), 1.27 – 1.11 (m, 18H) ppm.

^{13}C-NMR (151 MHz, DMSO-d$_6$): δ = 169.6, 168.6, 153.0, 150.3, 123.7, 41.8, 33.4, 28.4, 24.4, 23.4, 22.3 ppm.

HRMS (ESIpos) m/z: Calcd for $C_{19}H_{31}N_2O_4S$ 383.1999, Found 383.1991.

IR (cm^{-1}): 3303, 2958, 1703, 1627, 1493, 1374, 1160.

(*S*)-2-acetamido-3-methyl-N-((2,4,6-triisopropylphenyl)sulfonyl)butanamide

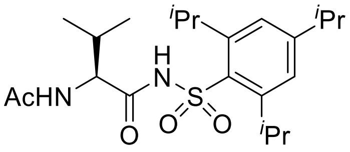

L11

Following the general procedure **A** using DMAP (768 mg, 6.28 mmol, 2.0 equiv.), EDC·HCl (903 mg, 4.71 mmol, 1.5 equiv.), N-Ac-Val-OH (500 mg, 3.14 mmol, 1.0 equiv.), and 2,4,6-triisopropylbenzenesulfonamide (979 mg, 3.46 mmol, 1.1 equiv.) the target compound L was obtained as a colorless solid (770 mg, 1.81 mmol, 58 %).

^{1}H-NMR (600 MHz, DMSO-*d*$_6$): δ = 12.3 (s, 1H), 7.91 (d, J = 8.8 Hz, 1H), 7.24 (s, 2H), 4.27 (dd, J = 8.8, 6.3 Hz, 1H), 4.18 (hept, J = 6.9 Hz, 2H), 2.91 (hept, J = 6.9 Hz, 1H), 2.03 – 1.95 (m, J = 6.6 Hz, 1H), 1.81 (s, 3H), 1.22 – 1.19 (m, 12H), 1.16 (d, J = 6.8 Hz, 6H), 0.84 (d, J = 6.8 Hz, 3H), 0.77 (d, J = 6.9 Hz, 3H) ppm.

^{13}C NMR (151 MHz, DMSO- d_6): δ = 171.2, 169.4, 153.1, 150.5, 132.4, 123.6, 57.3, 33.4, 30.2, 28.5, 24.7, 24.1, 23.4, 23.3, 22.3, 19.1, 17.5 ppm.

HRMS (ESIpos) m/z: Calcd for $C_{22}H_{37}N_2O_4S$ 425.2468 Found 425.2462.

IR (cm^{-1}): 3383, 3006, 1725, 1648, 1457, 1275, 1032.

(*S*)-2-acetamido-4-methyl-N-((2,4,6-triisopropylphenyl)sulfonyl)pentanamide

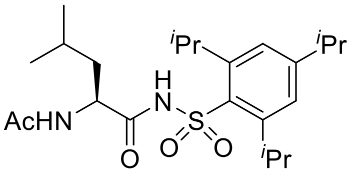

L12

Following the general procedure **A** using DMAP (705 mg, 5.77 mmol, 2.0 equiv.), EDC·HCl (830 mg, 4.33 mmol, 1.5 equiv.), N-Ac-Leu-OH (500 mg, 2.89 mmol, 1.0 equiv.), and 2,4,6-triisopropylbenzenesulfonamide (900 mg, 3.18 mmol, 1.1 equiv.) the target compound **L12** was obtained as a colorless solid (710 mg, 1.62 mmol, 56 %).

^{1}H-NMR (600 MHz, DMSO-d_6): δ **=** 12.38 (s, 1H), 8.01 (d, *J* = 8.2 Hz, 1H), 7.24 (s, 2H), 4.38 – 4.41 (m, 1H), 4.18 (hept, *J* = 6.7 Hz, 2H), 2.91 (hept, *J* = 7.0 Hz, 1H), 1.78 (s, 3H), 1.62 – 1.53 (m, 1H), 1.40 – 1.35 (m, 2H), 1.22 – 1.15 (m, 18H), 0.86 (d, *J* = 6.7 Hz, 3H), 0.83 (d, *J* = 6.6 Hz, 3H) ppm.

^{13}C NMR (151 MHz, DMSO- d_6): δ = 172.2, 169.2, 153.1, 150.6, 132.3, 123.7, 50.9, 33.4, 28.5, 24.5, 24.3, 24.3, 23.4, 23.3, 23.1, 22.3, 20.9 ppm.

HRMS (ESIpos) m/z: Calcd for $C_{23}H_{39}N_2O_4S$ 439.2625, Found 439.2619.

IR (cm^{-1}): 3323, 2969, 1704, 1467, 1275, 1144, 1034.

(*S*)-2-acetamido-3-phenyl-N-((2,4,6-triisopropylphenyl)sulfonyl)propanamide

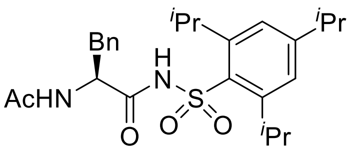

L14

Following the general procedure **A** using DMAP (590 mg, 4.83 mmol, 2.0 equiv.), EDC·HCl (694 mg, 3.62 mmol, 1.5 equiv.), N-Ac-Phe-OH (500 mg, 2.41 mmol, 1.0 equiv.), and 2,4,6-triisopropylbenzenesulfonamide (750 mg, 2.65 mmol, 1.1 equiv.) the target compound **L14** was obtained as a colorless solid (475 mg, 1.00 mmol, 42 %).

^{1}H-NMR (600 MHz, DMSO-*d*$_6$): δ **=** 12.57 (s, 1H), 8.14 (d, *J* = 8.5 Hz, 1H), 7.28 – 7.24 (m, 6H), 7.19 (m, 1H), 4.53 (m, 1H), 4.23 (hept, *J* = 6.7 Hz, 2H), 2.99 (dd, *J* = 13.6, 3.6 Hz, 1H), 2.93 (hept, *J* = 6.9 Hz, 1H), 2.64 (dd, *J* = 13.6, 11.0 Hz, 1H), 1.68 (s, 3H), 1.23 – 1.18 (m, 18H) ppm.

^{13}C NMR (151 MHz, DMSO- *d*$_6$): δ = 171.1, 169.2, 153.2, 150.6, 137.3, 132.2, 129.1, 128.1, 126.5, 123.8, 54.0, 36.8, 33.4, 28.5, 24.5, 24.3, 23.4, 23.3, 22.3 ppm.

HRMS (ESIpos) m/z: Calcd for $C_{26}H_{36}N_2O_4SNa$ 495.2288, Found 495.2287.

IR (cm^{-1}): 3344, 2956, 1697, 1658, 1453, 1363, 1180.

2-acetamido-N-((2,3,5,6-tetramethylphenyl)sulfonyl)acetamide

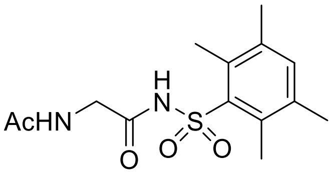

L17

Following the general procedure **A** using DMAP (313 mg, 2.56 mmol, 2.0 equiv.), EDC·HCl (368 mg, 1.92 mmol, 1.5 equiv.), N-Ac-Gly-OH (150 mg, 1.28 mmol, 1.0 equiv.), and 2,3,5,6-tetramethylbenzenesulfonamide (301 mg, 1.41 mmol, 1.1 equiv.) the target compound **L17** was obtained as a colorless solid (92 mg, 0.3 mmol, 23 %).

^{1}H NMR (600 MHz, Methanol-*d*$_4$) δ = 7.23 (s, 1H), 3.85 (s, 2H), 2.58 (s, 6H), 2.28 (s, 6H), 1.93 (s, 3H) ppm.

^{13}C NMR (151 MHz, Methanol-*d*$_4$) δ = 173.8, 170.1, 137.4, 137.3, 137.2, 128.8, 43.4, 22.2, 21.0, 17.8 ppm.

HRMS (ESIpos) m/z: Calcd for $C_{14}H_{21}N_2O_4S$ 313.1216 Found 313.1209.

IR (cm^{-1}): 2958, 1730, 1635, 1525, 1448, 1275, 1033.

2-acetamido-N-((2,4,6-triisopropylphenyl)sulfonyl)propanamide

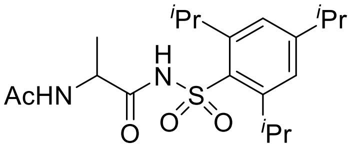

L18 Following the general procedure **A** using DMAP (556 mg, 4.55 mmol, 2.0 equiv.), EDC·HCl (658 mg, 3.43 mmol, 1.5 equiv.), N-Ac-Ala-OH (300 mg, 2.29 mmol, 1.0 equiv.), and 2,4,6-triisopropylbenzenesulfonamide (713 mg, 2.52 mmol, 1.1 equiv.) the target compound **L18** was obtained as a colorless solid (570 mg, 1.44 mmol, 63 %).

^{1}H-NMR (600 MHz, DMSO-d_6): δ = 12.29 (s, 1H), 8.10 (d, *J* = 7.5 Hz, 1H), 7.24 (s, 2H), 4.28 (p, *J* = 7.2 Hz, 1H), 4.19 (hept, *J* = 6.4 Hz, 2H), 2.91 (hept, *J* = 7.5, 7.1 Hz, 1H), 1.78 (s, 3H), 1.27 – 1.13 (m, 18H), 1.18 (s, 3H) ppm.

^{1}H-NMR (600 MHz, DMSO-d_6): δ = 172.1, 169.0, 153.1, 150.5, 123.7, 48.2, 33.4, 28.5, 24.5, 24.3, 23.4, 22.3, 17.5 ppm.

HRMS (ESIpos) m/z: Calcd for $C_{20}H_{33}N_2O_4S$ 397.2155, Found 397.2149.

IR (cm^{-1}): 3408, 3200, 3957, 1730, 1635, 1448, 1365, 1144.

2-acetamido-N-(mesitylsulfonyl)propanamide

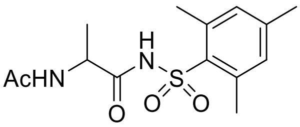

L19 Following the general procedure **A** using DMAP (932 mg, 7.63 mmol, 2.0 equiv.), EDC·HCl (1.1 g, 5.72 mmol, 1.5 equiv.), N-Ac-Ala-OH (500 mg, 3.81 mmol, 1.0 equiv.), and 2,4,6-trimethylbenzenesulfonamide (836 mg, 4.19 mmol, 1.1 equiv.) the target compound **L19** was obtained as a colorless solid (500 mg, 1.60 mmol, 42 %).

^{1}H NMR (600 MHz, DMSO-d_6): δ 12.18 (s, 1H), 7.03 (s, 2H), 4.22 (p, *J* = 7.1 Hz, 1H), 2.59 (s, 6H), 2.26 (s, 3H), 1.77 (s, 3H), 1.14 (d, *J* = 7.2 Hz, 3H) ppm.

^{13}C-NMR (151 MHz, DMSO-d$_6$): δ = 172.1, 169.1, 142.8, 139.6, 133.1, 131.5, 48.3, 22.2, 22.0, 20.5, 17.1 ppm.

HRMS (ESIpos) m/z: Calcd for $C_{14}H_{21}N_2O_4S$ 313.1216, Found 313.1208.

IR (cm^{-1}): 3339, 2982, 1708, 1645, 1542, 1172, 1145.

3-acetamido-N-(mesitylsulfonyl)propenamide

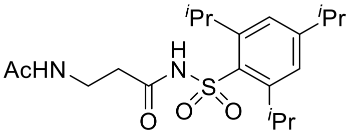

L20 Following the general procedure **A** using DMAP (969 mg, 7.93 mmol, 2.0 equiv.), EDC·HCl (1.14 g, 5.95 mmol, 1.5 equiv.), N-Ac-ß-Ala-OH (520 mg, 3.97 mmol, 1.0 equiv.), and 2,4,6-triisopropylbenzenesulfonamide (1.24 g, 4.36 mmol, 1.1 equiv.) the target compound **L20** was obtained as a colorless solid (1.10 g, 2.77 mmol, 70 %).

^{1}H-NMR (600 MHz, DMSO-*d*$_6$): δ = 12.2 (s, 1H), 7.9 (t, *J* = 5.6 Hz, 2H), 4.2 (p, *J* = 6.8 Hz, 2H), 3.1 (td, *J* = 6.8, 5.6 Hz, 2H), 2.9 (p, *J* = 6.9 Hz, 1H), 2.4 (t, *J* = 6.8 Hz, 2H), 1.7 (s, 3H), 1.2 (dd, *J* = 11.3, 6.8 Hz, 18H) ppm.

^{13}C NMR (151 MHz, DMSO- *d*$_6$): δ =170.2, 169.3, 153.0, 150.3, 132.4, 123.7, 35.3, 33.9, 33.4, 28.4, 24.3, 23.4, 22.4 ppm.

HRMS (ESIpos) m/z: Calcd for $C_{20}H_{32}N_2O_4SNa$ 419.1975, Found 419.1970.

IR (cm^{-1}): 3307, 2964, 1697, 1649, 1569, 1262, 1050.

3. Optimization of the Reaction Conditions

General Procedure for the Optimization Reactions:

An oven dried 10 mL Schlenk tube was charged with $Pd(OAc)_2$, ligand, base, silver salt, 2,2-dimethylbutanoic acid (0.1 mmol), styrene, and HFIP. The Schlenk tube was transferred to a preheated aluminum block and the reaction mixture was stirred while heating at the indicated temperature. After the indicated time, the reaction mixture was allowed to cool to room temperature. Formic acid (0.1 mL) was added to the reaction mixture and it was filtered over a pad of Celite® using CH_2Cl_2 (30 mL) to complete the elution. All volatiles were removed under reduced pressure followed by the addition of CH_2Br_2 (17.4 mg, 0.100 mmol) as internal standard. $CDCl_3$ (0.8 mL) was used to prepare a sample for NMR analysis. All yields during the optimization studies were determined by ^{1}H-NMR analysis of the crude reaction mixture using CH_2Br_2 as internal standard.

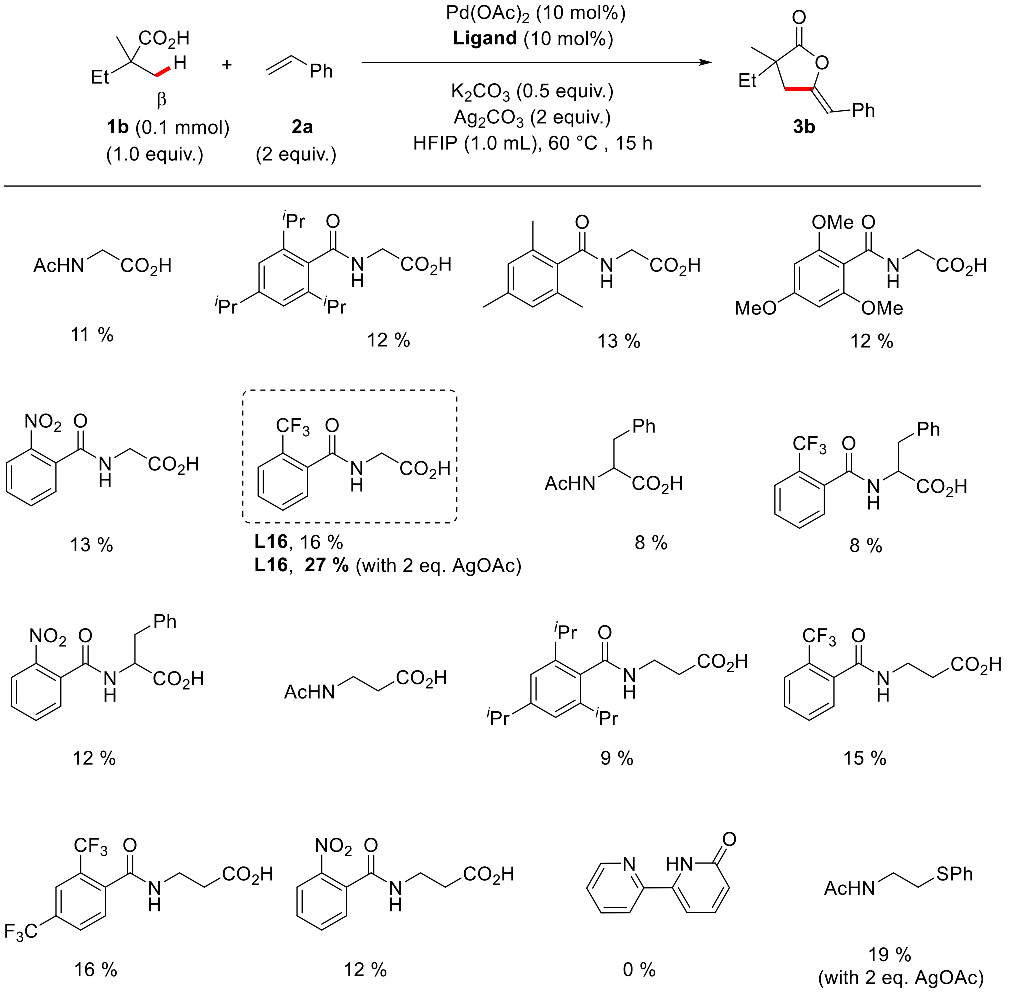

Scheme S1: Preliminary screening of different ligand classes with Ag_2CO_3

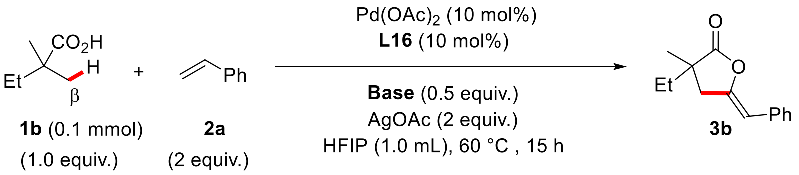

Entry	Base	NMR-Yield (%)	Entry	Base	NMR-Yield (%)
1.	$Na_2HPO_4 \cdot 7H_2O$	< 3	12.	K_2CO_3 (0.75 equiv.)	26
2.	Na_2HPO_4	7	13.	K_2CO_3 (1.0 equiv.)	24
3.	Na_2CO_3	16	14.	$KHCO_3$(1.0 equiv.)	19
4.	$NaHCO_3$ (1.0 equiv.)	16	15.	KO^tBu (0.75 equiv.)	24
5.	$NaOAc \cdot 3H_2O$ (1.0 equiv.)	16	16.	KO^tBu (1.0 equiv.)	23
6.	NaOAc (1.0 equiv.)	11	17.	KO^tBu (1.5 equiv.)	**26**
7.	NaO^tBu (1.0 equiv.)	16	18.	KO^tBu (2.0 equiv.)	17
8.	NaHFIP (0.75 equiv.)	19	19.	KOAc (1.0 equiv.)	15
9.	NaHFIP (1.0 equiv.)	23	20.	KHFIP (1.0 equiv.)	15
10.	NaHFIP (1.5 equiv.)	17	21.	Cs_2CO_3	17
11.	K_2CO_3	**27**	22.	CsOAc	10

Scheme S2: Screening of different bases with L16

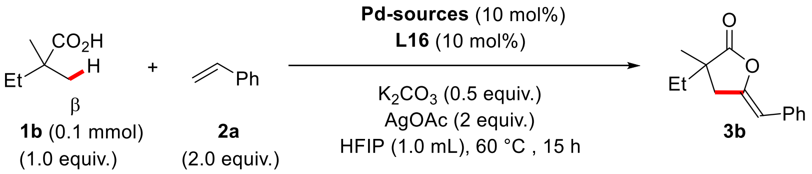

Entry	Pd-sources	NMR-Yield (%)
1.	$Pd(OAc)_2$	**27**
2.	$Pd(CF_3CO_2)_2$	21
3.	$Pd(CH_3CN)_2Cl_2$	17
4.	$[Pd(\pi\text{-cinnamyl})Cl]_2$	20

Scheme S3: Screening of different Pd-sources with L16

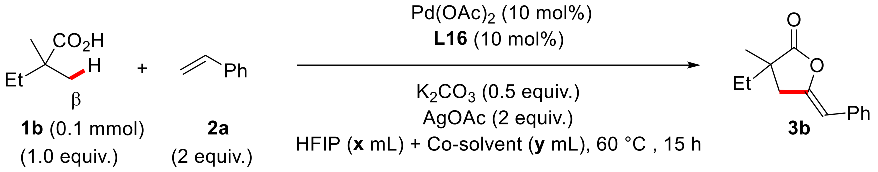

Entry	x (mL)	Co-solvent (y mL)	NMR-Yield (%)
1.	1.0	None	**27**
2.	0.95	TFE (0.05)	19
3.	0.95	CH_3CN (0.05)	14
4.	0.95	THF(0.05)	22
5.	0.95	*tert*-Amyl alcohol (0.05)	21
6.	0.95	DCE (0.05)	23
7.	0.95	Toluene (0.05)	11
8.	0.95	DMF(0.05)	21
9.	0.95	DMSO (0.05)	18
10.	0.95	1,4-Dioxane (0.05)	22

Scheme S4: Screening of co-solvents with L16

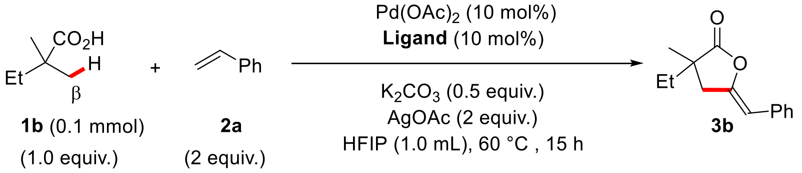

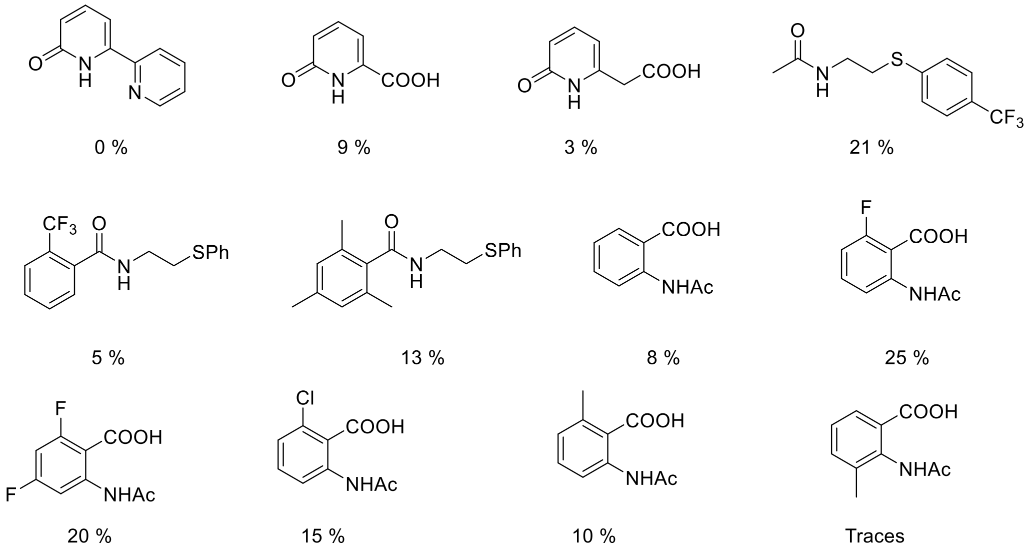

Scheme S5: Screening of different ligand classes with AgOAc

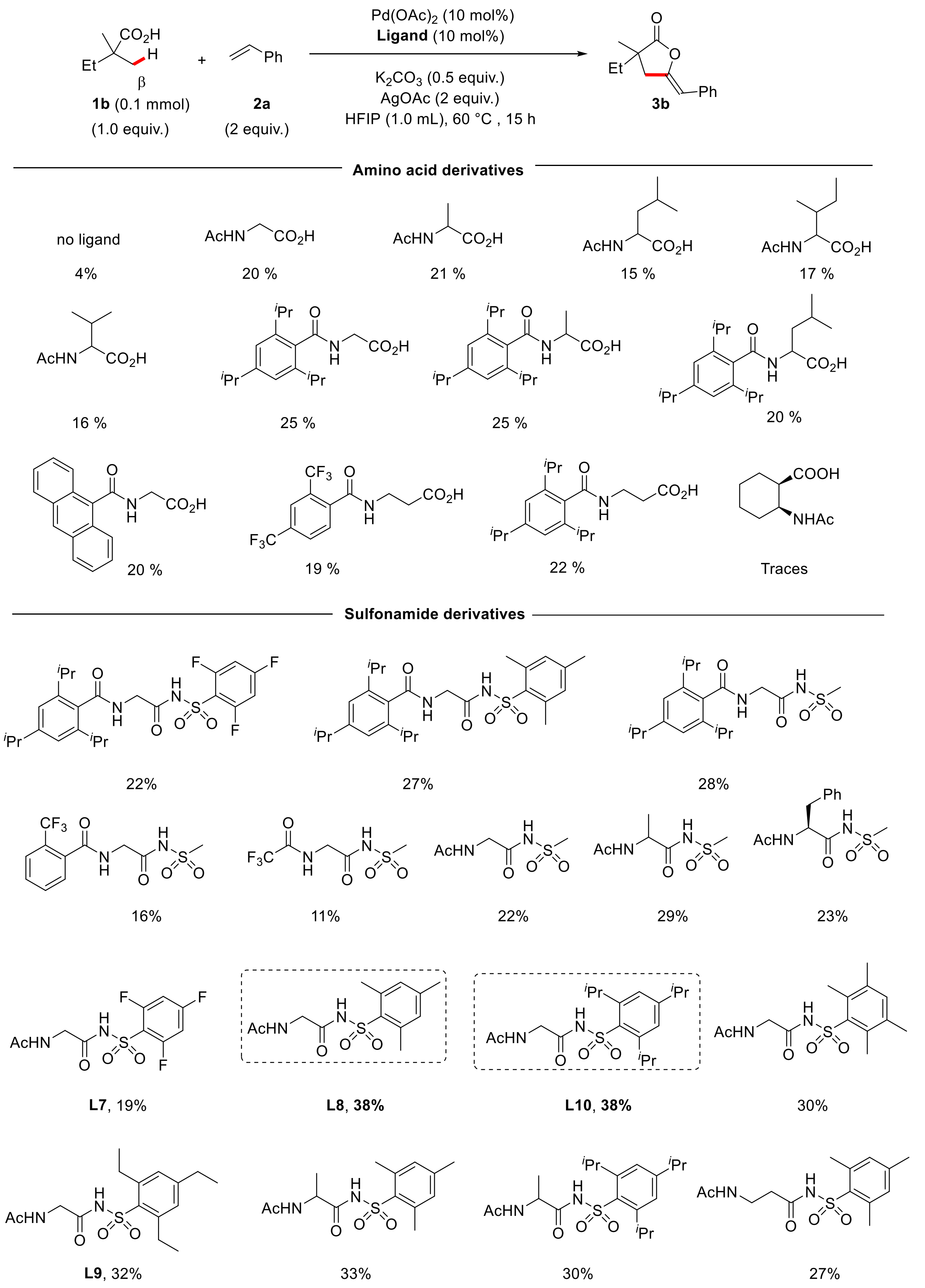

Scheme S6: Screening of amino acid and N-acylsulfonamide derivatives with AgOAc

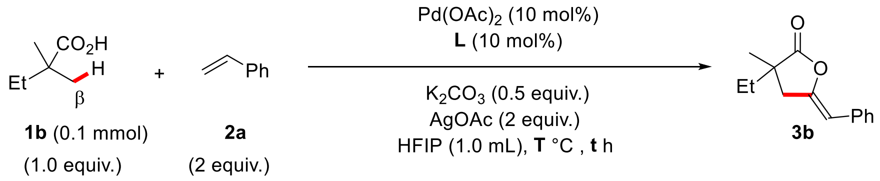

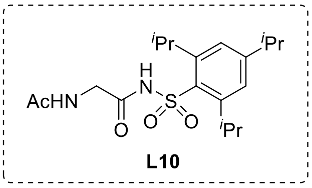

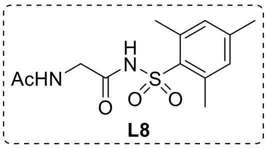

Entry	T (°C)	t (h)	NMR-Yield (%)
1.	50	16	27
2.	50	24	24
3.	60	15	38
4.	60	24	35
5.	70	12	38
6.	70	18	**40**
7.	70	24	37
8.	50	16	24
9.	50	24	30
10.	60	15	38
11.	60	24	33
12.	70	12	34
13	70	18	31

Scheme S7: Screening of time and temperature with L8 and L10

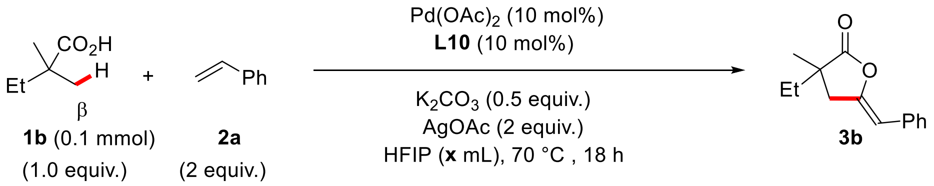

Entry	x (mL)	NMR-Yield (%)
1.	1.0	**45**
2.	1.3	36
3.	1.6	35
4.	1.9	36

Scheme S8: Screening of solvent amount with L10

Note: The experiments shown in **Scheme S8** were performed using a stock solution of **1b**, $Pd(OAc)_2$, and **L10** in HFIP. As the stock solution slightly improved the yield (Entry 1, **Scheme S8**), the following experiments (scheme **S9** - **S15**) have been performed using stock solution.

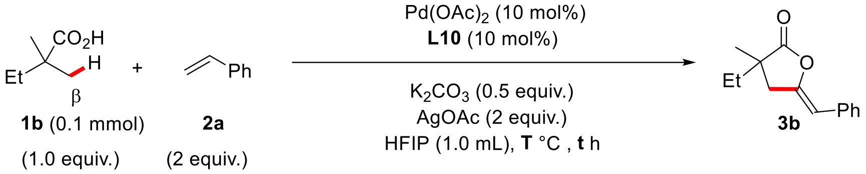

Entry	T (°C)	t (h)	NMR-Yield (%)
1.	70	18	45
2.	70	21	**49**
3.	70	24	44
4.	70	30	41
5.	80	15	31
6.	80	18	35

Scheme S9: Re-screening of temperature and time with L10 using a stock solution

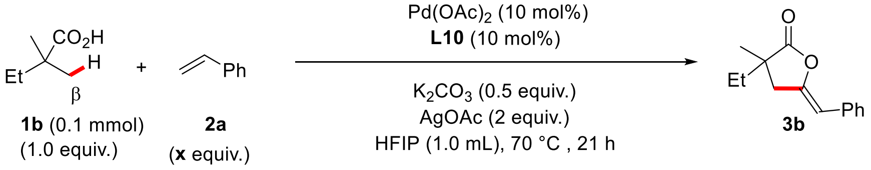

Entry	x (equiv.)	NMR-Yield (%)
1.	1.7	34
2.	2.0	49
3.	2.3	56
4.	2.5	**58**
5.	2.7	53
6.	2.9	48

Scheme S10: Screening of equivalents of styrene with L10

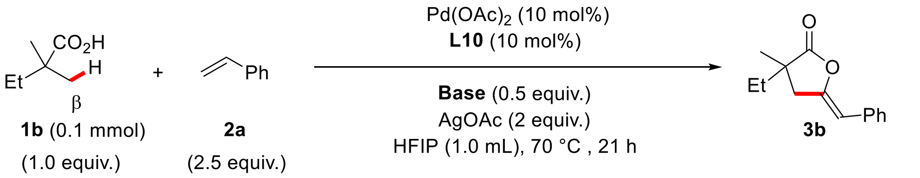

Entry	Base	NMR-Yield (%)	Entry	Base	NMR-Yield (%)
1.	no base	32	7.	Na_2CO_3	52
2.	K_2CO_3	**58**	8.	$NaOAc{\cdot}3H_2O$ (1.0 equiv.)	48
3.	K_2CO_3 (0.75 equiv.)	52	9.	NaOAc (1.0 equiv.)	49
4.	K_2CO_3 (1.0 equiv.)	47	10.	Cs_2CO_3	41
5.	KOAc (1.0 equiv.)	44	11.	CsOAc (1.0 equiv.)	28
6.	KO^tBu (1.0 equiv.)	52	12.	Li_2CO_3	52

Scheme S11: Re-screening of different bases with L10

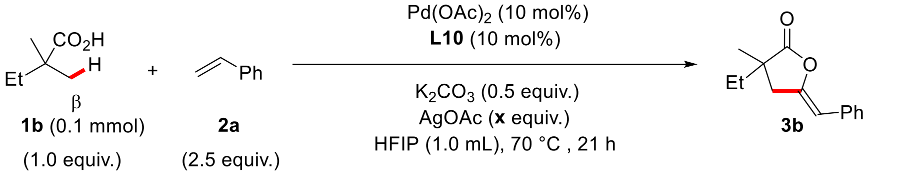

Entry	**x** (equiv.)	NMR-Yield (%)
1.	no AgOAc	< 4
2.	1.5	49
3.	2	**58**
4.	2.5	51
5.	3	49
6.	3.5	50

Scheme S12: Screening of equivalents of AgOAc with L10

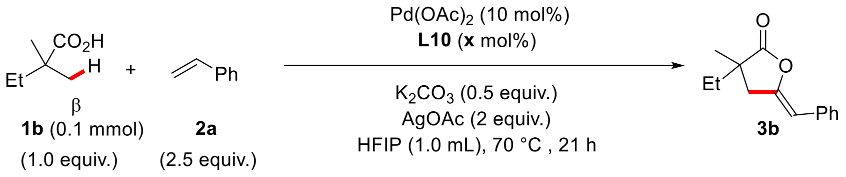

Entry	x (mol%)	NMR-Yield (%)
1.	5	35
2.	10	**58**
3.	15	53
4.	20	48

Scheme S13: Screening of ligand (L10) loading

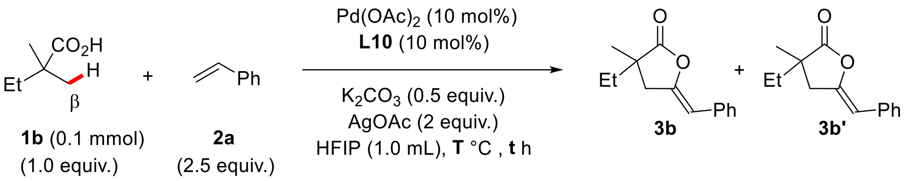

Entry	T (°C)	t (h)	3b (%)	3b' (%)
1.	60	24	53	6
2.	60	48	47	5
3.	60	72	46	6
4.	70	21	**58**	6
5.	70	24	54	6
6.	70	30	50	5

Scheme S14: Screening of temperature and time with L10

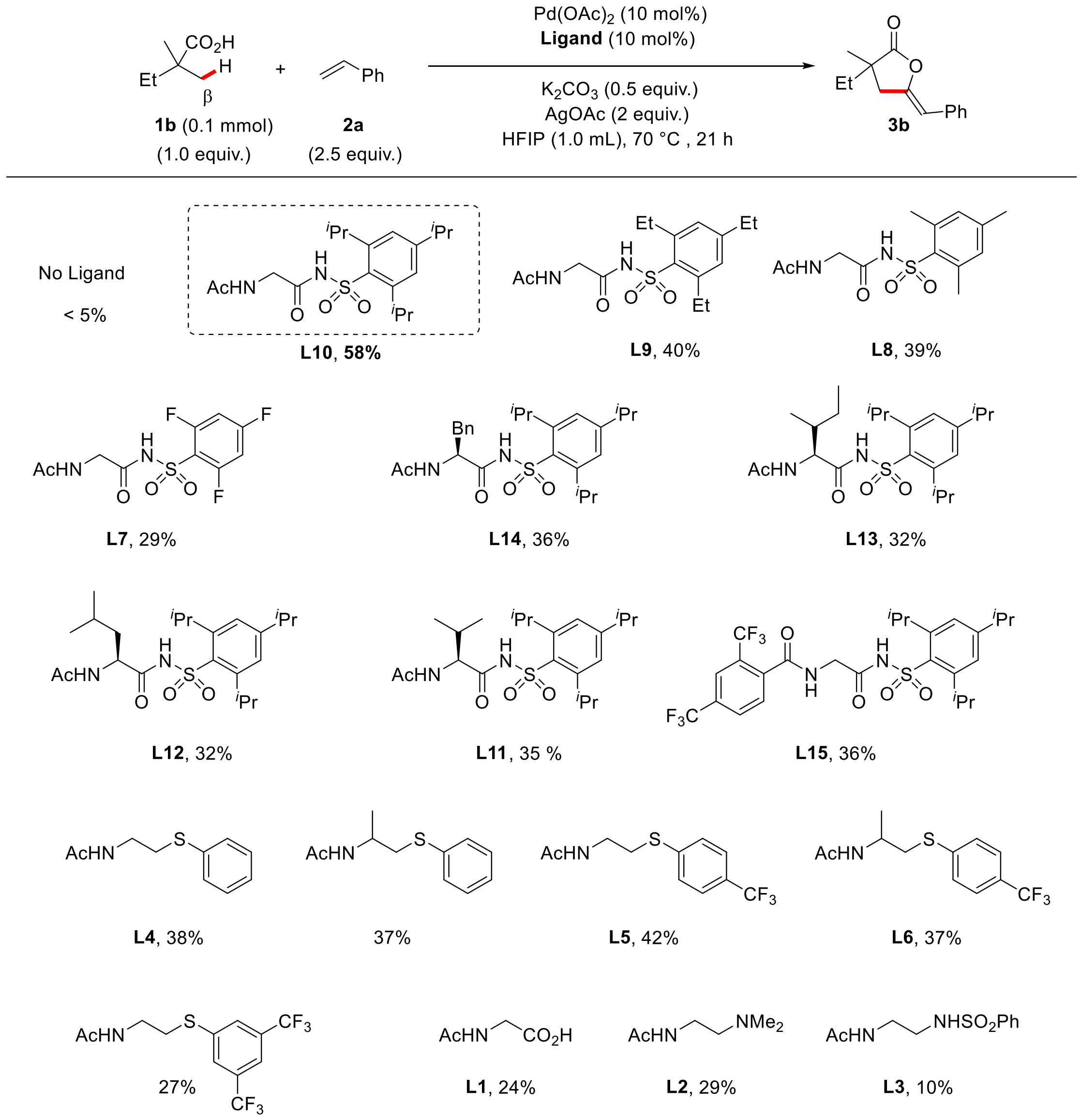

Scheme S15: Re-evaluation of the ligand structure under the newly optimized conditions

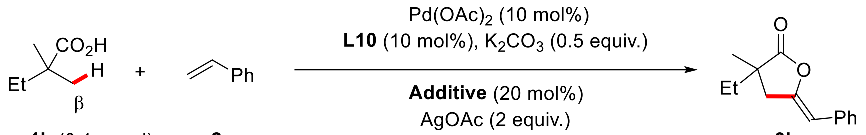

1b (0.1 mmol) (1.0 equiv.) + **2a** (2.5 equiv.) → **3b**

Entry	**Additive**	NMR-Yield
1.	no additive	58%
2.	CuO	53%
3.	$CuCl_2$	40%
4.	$CeCl_3$	43%
5.	$FeCl_3$	33%
6.	MnO_2	**66%**
7.	MnO_2 (35 mol%)	66%
8.	MnO_2 (50 mol%)	63%
9.	$KMnO_4$	56%
10.	$Fe(NO_3){\cdot}9H_2O$	44%

Scheme S16: Screening of additives with L10

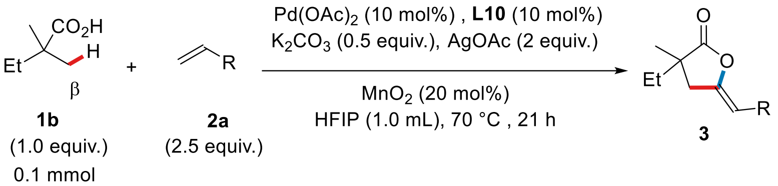

Entry	Olefin	NMR-Yield[a]
1.	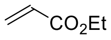	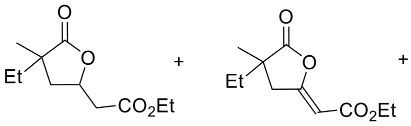 (10%) + (0%) + **1b**(> 60%)
2.	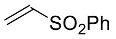	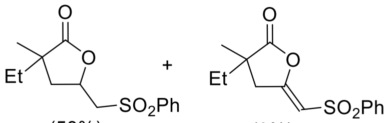 (58%) + (0%) + **1b** (40%)
3.	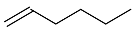	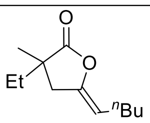 + **1b** (> 65%)
4.	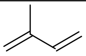	**1b** (> 95%)
5.	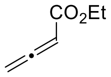	**1b** (> 95%)

[a]Yields were determined by ^1H NMR analysis of the crude reaction mixture using CH_2Br_2 as an internal standard

Scheme S17: Screening of varios olefinic partner.

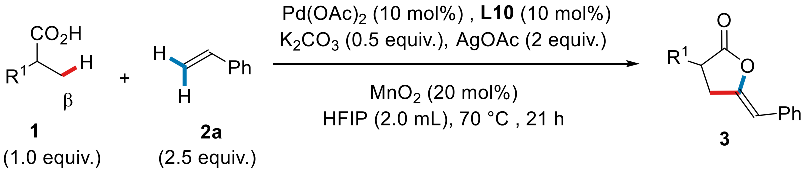

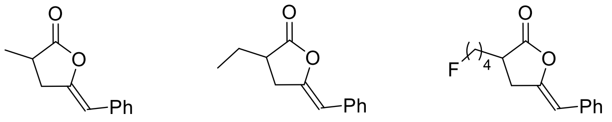

NMR-Yield[a]:	4%	6%	< 5%
Conversion[a]:	50%	10-15%	10-12%

[a]Yields and conversions were determined by ^1H NMR analysis of the crude reaction mixture using CH_2Br_2 as an internal standard

Scheme S18: Screening of α-non-quaternary acids.

4. Synthesis of Carboxylic Acids and Styrenes

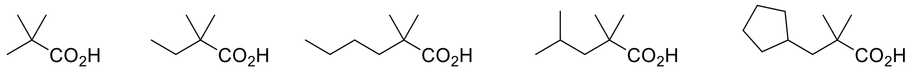

1a 1b 1c 1d 1e

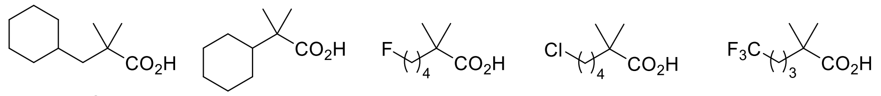

1f 1g 1h 1i 1j

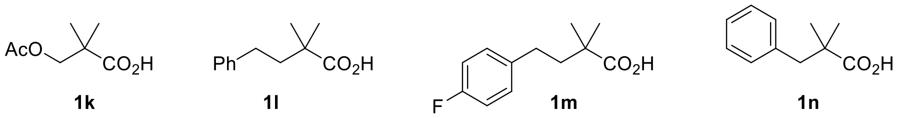

1k 1l 1m 1n

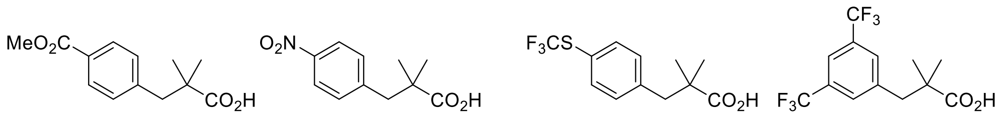

1o 1p 1q 1r

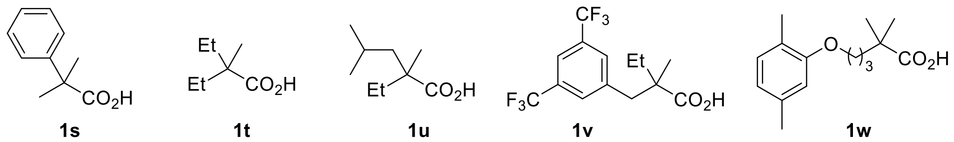

1s 1t 1u 1v 1w

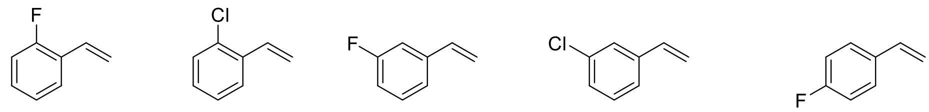

2b 2c 2d 2e 2f

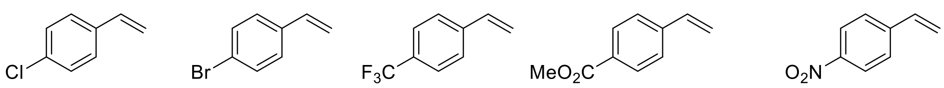

2g 2h 2i 2j 2k

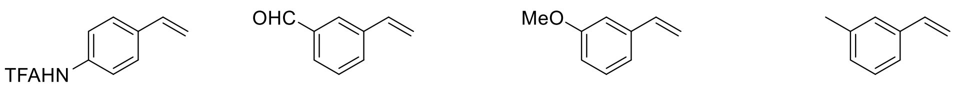

2l 2m 2n 2o

Carboxylic acids were obtained from either commercially available sources or synthesized using literature protocol.[1]

5. Scope Studies

General Procedure B: Carboxylic Acid Scope

An oven dried 10 mL Schlenk tube was charged with $Pd(OAc)_2$ (4.5 mg, 20 µmol, 10 mol%), **L10** (7.6 mg, 20 µmol, 10 mol%), K_2CO_3 (13.8 mg, 0.100 mmol, 0.5 equiv.), AgOAc (66.8 mg, 0.400 mmol, 2 equiv.), MnO_2 (3.5 mg, 40 µmol, 20 mol%), styrene **2a** (58 µL, 0.500 mmol, 2.5 equiv.), carboxylic acid **1** (0.200 mmol, 1.0 equiv.), and HFIP (2.0 mL). The reaction mixture was stirred at 70 °C for 21 h in a preheated metal block. After the mixture was allowed to cool to room temperature, formic acid (0.1 mL) was added to the reaction mixture and it was filtered over a pad of Celite® using CH_2Cl_2 (30 mL) to complete the elution. All volatiles were removed under reduced pressure, and the residue was purified by silica gel column chromatography.

General Procedure C: Olefin Scope

An oven dried 10 mL Schlenk tube was charged with $Pd(OAc)_2$ (4.5 mg, 20 µmol, 10 mol%), **L10** (7.6 mg, 20 µmol, 10 mol%), K_2CO_3 (13.8 mg, 0.100 mmol, 0.5 equiv.), AgOAc (66.8 mg, 0.400 mmol, 2 equiv.), MnO_2 (3.5 mg, 40 µmol, 20 mol%), styrene **2** (0.500 mmol, 2.5 equiv.), carboxylic acid **1b** (23.2 mg, 0.200 mmol, 1.0 equiv.), and HFIP (2.0 mL). The reaction mixture was stirred at 70 °C for 21 h in a preheated metal block. The mixture was allowed to cool to room temperature and formic acid (0.1 mL) was added to the reaction mixture. The mixture was filtered over a pad of Celite® using CH_2Cl_2 (30 mL) to complete the elution. All volatiles were removed under reduced pressure, and the residue was purified by silica gel column chromatography.

(*Z*)-5-Benzylidene-3,3-dimethyldihydrofuran-2(3H)-one

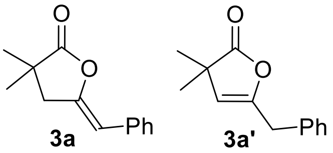

Following the general procedure **B**, using **1a** (20.4 mg) and pentane/Et_2O (98:2 v/v) as an eluent, the target compound was obtained as a colorless oil (18.2 mg, 0.899 mmol, 45%, **3a : 3a**´ = 11:1).

^{1}H NMR (600 MHz, $CDCl_3$): δ = 7.58 – 7.54 (m, $2H^{3a}$ + $2H^{3a'}$), 7.34 – 7.31 (m, $2H^{3a}$ + $2H^{3a'}$), 7.22 – 7.19 (m, $1H^{3a}$ + $1H^{3a'}$), 5.55 (t, *J* = 1.7 Hz, $1H^{3a}$), 5.07 (t, *J* = 1.7 Hz, $1H^{3a'}$), 2.60 (d, *J* = 1.1 Hz, $2H^{3a'}$), 2.85 (d, *J* = 1.7 Hz, $2H^{3a}$), 1.36 (s, $6H^{3a}$), 1.28 (s, $6H^{3a'}$) ppm.

^{13}C NMR (151 MHz, $CDCl_3$): δ = 182.7 (3a´), 180.3 (3a), 153.1 (3a´), 146.2 (3a), 135.4 (3a´), 134.1 (3a), 129.2 (3a´), 128.8 (3a´), 128.6 (3a), 128.5 (3a), 127.2 (3a´), 126.9 (3a), 112.1 (3a´), 105.4 (3a), 44.6 (3a´), 42.4 (3a), 39.4 (3a), 34.8 (3a´), 24.8 (3a), 24.5 (3a´) ppm.

HRMS (ESIpos) m/z: Calcd for $C_{13}H_{15}O_2$ 203.1066, Found 203.1064.

IR (cm^{-1}): 2971, 1794, 1689, 1449, 1275, 1260, 1059, 936, 750, 693.

(*Z*)-5-Benzylidene-3-ethyl-3-methyldihydrofuran-2(3H)-one

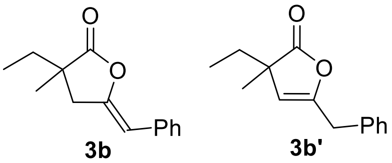

Following the general procedure **B**, using **1b** (23.2 mg) and pentane/Et_2O (98:2 v/v) as an eluent, the target compound was obtained as a colorless oil (28.9 mg, 0.134 mmol, 67%, **3b : 3b**´ = 10:1).

^{1}H NMR (600 MHz, $CDCl_3$): δ = 7.57 – 7.54 (m, $2H^{3b}$ + $2H^{3b'}$), 7.34 – 7.31 (m, $2H^{3b}$ + $2H^{3b'}$), 7.22 – 7.18 (m, $1H^{3b}$ + $1H^{3b'}$), 5.54 (t, *J* = 1.7 Hz, $1H^{3b}$), 4.98 (t, *J* = 1.5 Hz, $1H^{3b'}$), 3.62 (d, *J* = 1.1 Hz, $1H^{3b'}$), 2.93 (dd, *J* = 16.2, 1.7 Hz, $1H^{3b}$), 2.76 (dd, *J* = 16.2, 1.8 Hz, $1H^{3b}$), 1.76 – 1.64 (m, $2H^{3b}$ + $2H^{3b'}$), 1.33 (s, $3H^{3b}$), 1.26 (s, $3H^{3b'}$), 0.97 (t, *J* = 7.5 Hz, $3H^{3b}$), 0.82 (t, *J* = 7.5 Hz, $3H^{3b'}$) ppm.

^{13}C NMR (151 MHz, $CDCl_3$): δ = 182.3 (3b´), 179.9 (3b), 153.7 (3b´), 146.5 (3b), 135.5 (3b´), 134.2 (3b), 129.2 (3b´), 128.8 (3b´), 127.1 (3b´), 128.6 (3b), 128.5 (3b), 126.8 (3b), 109.9 (3b´), 105.1 (3b), 49.6 (3b´), 43.4 (3b), 39.6 (3b), 34.8 (3b´), 31.1 (3b´), 30.9 (3b), 23.1 (3b´), 23.0 (3b), 9.4 (3b´), 8.9 (3b) ppm.

HRMS (ESIpos) m/z: Calcd for $C_{14}H_{17}O_2$ 217.1223, Found 217.1220.

IR (cm^{-1}): 2969, 1792, 1690, 1450, 1226, 1180 1071, 936, 750.

(*Z*)-5-Benzylidene-3-methyl-3-propyldihydrofuran-2(3H)-one

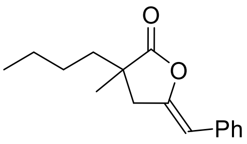

3c

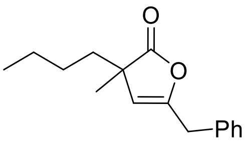

3c'

Following the general procedure **B**, using **1c** (28.8 mg) and pentane/Et_2O (98:2 v/v) as an eluent, the target compound was obtained as a colorless oil (23.0 mg, 94.1 µmol, 47%, **3c : 3c´** = 6:1).

^{1}H-NMR (600 MHz, CDCl$_3$): δ = 7.58 – 7.52 (m, $2H^{3c}$ + $2H^{3c'}$), 7.34 – 7.30 (m, $2H^{3c}$ + $2H^{3c'}$), 7.22 – 7.18 (m, $1H^{3c}$ + $1H^{3c'}$), 5.53 (t, *J* = 1.7 Hz, $1H^{3c}$), 5.00 (t, *J* = 1.4 Hz, $1H^{3c'}$), 3.62 (d, *J* = 1.4 Hz, $1H^{3c'}$), 2.94 (dd, *J* = 16.2, 1.7 Hz, $1H^{3c}$), 2.77 (dd, *J* = 16.2, 1.8 Hz, $1H^{3c}$), 1.68 – 1.60 (m, $2H^{3c}$ + $2H^{3c'}$), 1.44 – 1.35 (m, $1H^{3c}$ + $1H^{3c'}$), 1.28 – 1.21 (m, $3H^{3c}$ + $3H^{3c'}$), 1.33 (s, $3H^{3c}$), 0.91 (t, *J* = 7.2 Hz, $3H^{3c}$), 0.86 (t, *J* = 7.2 Hz, $1H^{3c'}$) ppm.

^{13}C-NMR (151 MHz, CDCl$_3$): δ = 182.4 (3c´), 180.0 (3c), 153.4 (3c´), 146.5 (3c), 135.6 (3c´), 134.2 (3c), 129.2 (3c´), 128.8 (3c´), 128.6 (3c), 128.5 (3c), 127.1 (3c´), 126.8 (3c), 110.3 (3c´), 105.1 (3c), 49.0 (3c´), 42.9 (3c), 40.0 (3c), 37.9 (3c´), 37.8 (3c), 34.8 (3c´), 27.3 (3c´), 26.6 (3c), 23.6 (3c´), 23.5 (3c), 23.0 (3c), 22.9 (3c´), 13.9 (3c), 14.0 (3c´) ppm.

HRMS (ESIpos) m/z: Calcd for $C_{16}H_{21}O_2$ 245.1536, Found 245.1533.

IR (cm^{-1}): 2931, 1797, 1687, 1449, 1224, 1177, 1038, 929, 831.

(*Z*)-5-Benzylidene-3-isobutyl-3-methyldihydrofuran-2(3H)-one

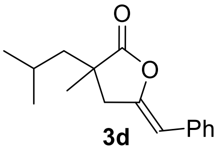

3d

Following the general procedure **B**, using **1d** (28.8 mg) and pentane/Et_2O (97:3 v/v) as an eluent, the target compound **3d** was obtained as a colorless oil (24.2 mg, 99.0 µmol, 50%).

^{1}H-NMR (600 MHz, CDCl$_3$): δ = 7.58 – 7.55 (m, 2H), 7.34 – 7.30 (m, 2H), 7.22 – 7.18 (m, 1H) 5.55 (t, *J* = 1.7 Hz, 1H), 3.03 (dd, *J* = 16.2, 1.8 Hz, 1H), 2.76 (dd, *J* = 16.2, 1.6 Hz, 1H), 1.84 – 1.76 (m, 1H), 1.69 (dd, *J* = 14.3, 4.6 Hz, 1H), 1.58 (dd, *J* = 14.3, 4.6 Hz, 1H), 1.33 (s, 3H), 0.97 (d, *J* = 6.6 Hz, 3H), 0.92 (d, *J* = 6.6 Hz, 3H) ppm.

^{13}C-NMR (151 MHz, $CDCl_3$): δ = 180.4, 146.5, 134.2, 128.6, 128.5, 126.8, 105.1, 46.1, 42.6, 40.1, 25.1, 24.8, 24.5, 23.1 ppm.

HRMS (ESIpos) m/z: Calcd for $C_{16}H_{21}O_2$ 245.1536 , Found 245.1533.

IR (cm^{-1}): 2959, 1789, 1687, 1449, 1227, 1176, 1088, 1039, 940, 831.

(*Z*)-5-Benzylidene-3-(cyclopentylmethyl)-3-methyldihydrofuran-2(3H)-one

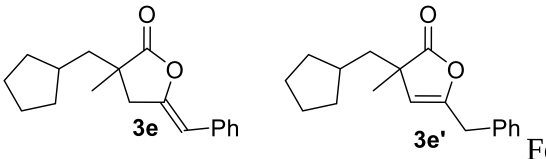

Following the general procedure **B**, using **1e** (34.1 mg) and pentane/Et_2O (98:2 v/v) as an eluent, the target compound was obtained as a colorless oil (25.2 mg, 93.2 µmol, 47%, **3e : 3e**´ = 7:1).

^{1}H-NMR (600 MHz, $CDCl_3$): δ =7.57 – 7.55 (m, $2H^{3e}$ + $2H^{3e'}$), 7.34 – 7.31 (m, $2H^{3e}$ + $2H^{3e'}$), 7.22 – 7.18 (m, $1H^{3e}$ + $1H^{3e'}$), 5.54 (t, *J* = 1.7 Hz, $1H^{3e}$), 5.05 (t, *J* = 1.4 Hz, $1H^{3e'}$), 3.62 (d, *J* = 1.2 Hz, $1H^{3e'}$), 3.02 (dd, *J* = 16.2, 1.7 Hz, $1H^{3e}$), 2.77 (dd, *J* = 16.2, 1.7 Hz, $1H^{3e}$), 1.89 – 1.76 (m, $4H^{3e}$ + $4H^{3e'}$), 1.72 (dd, *J* = 14.0, 7.5 Hz, $1H^{3e}$ + $1H^{3e'}$), 1.65 – 1.59 (m, $2H^{3e}$ + $2H^{3e'}$), 1.53 – 1.45 (m, $2H^{3e}$ + $2H^{3e'}$), 1.34 (s, 3H), 1.26 (s, $3H^{3e'}$), 1.17 – 1.07 (m, $2H^{3e}$ + $2H^{3e'}$) ppm.

^{13}C-NMR (151 MHz, $CDCl_3$): δ = 182.8 (3e´), 180.4 (3e), 152.9 (3e´), 146.6 (3e), 135.6 (3e´), 134.2 (3e), 129.1 (3e´), 128.7 (3e´), 127.1 (3e´), 128.6 (3e), 128.5 (3e), 126.8 (3e), 110.1 (3e´), 105.0 (3e), 49.0 (3e´), 44.4 (3e´), 43.9 (3e), 43.1 (3e), 40.3 (3e), 37.7 (3e´), 36.9 (3e), 34.9 (3e´), 34.6 (3e), 34.2 (3e´), 33.5 (3e), 33.0 (3e´), 25.4 (3e), 25.3 (3e´), 24.9 (3e), 24.8 (3e´), 24.4 (3e) ppm.

HRMS (ESIpos) m/z: Calcd for $C_{18}H_{23}O_2$ 271.1692, Found 271.1689.

IR (cm^{-1}): 2927, 1794, 1691, 1450, 1227, 1053, 951, 755.

(*Z*)-5-Benzylidene-3-(cyclohexylmethyl)-3-methyldihydrofuran-2(3H)-one

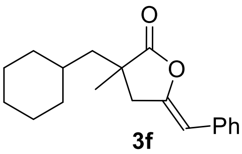

3f Following the general procedure **B**, using **1f** (36.9 mg) and pentane/Et_2O (98:2 v/v) as an eluent, the target compound **3f** was obtained as a colorless oil (29.5 mg, 0.103 mmol, 52%).

^{1}H-NMR (600 MHz, $CDCl_3$): δ = 7.58 – 7.53 (m, 2H), 7.34 – 7.30 (m, 2H), 7.22 – 7.18 (m, 1H), 5.55 (t, *J* = 1.7 Hz, 1H), 3.01 (dd, *J* = 16.1, 1.8 Hz, 1H), 2.74 (dd, *J* = 16.1, 1.6 Hz, 1H), 1.70 – 1.65 (m, 4H), 1.63 – 1.56 (m, 4H), 1.32 (s, 3H), 1.26 – 1.11 (m, 3H), 1.06 – 0.92 (m, 2H) ppm.

^{13}C-NMR (151 MHz, $CDCl_3$): δ = 180.5, 146.6, 134.2, 128.6, 128.5, 126.8, 105.1, 44.6, 42.5, 40.1, 35.1, 34.5, 33.8, 26.4, 26.3, 26.2, 24.5 ppm.

HRMS (ESIpos) m/z: Calcd for $C_{19}H_{25}O_2$ 285.1849 , Found 285.1845.

IR (cm^{-1}): 2917, 2846, 1790, 1682, 1447, 1223, 1147, 1068, 946, 844.

(*Z*)-5-Benzylidene-3-cyclohexyl-3-methyldihydrofuran-2(3H)-one

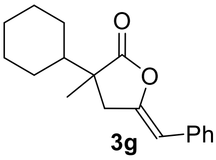

3g Following the general procedure **B**, using **1g** (34.1 mg) and pentane/Et_2O (98:2 v/v) as an eluent, the target compound **3g** was obtained as a colorless oil (37.8 mg, 0.139 mmol, 70%).

^{1}H-NMR (600 MHz, $CDCl_3$): δ = 7.57 – 7.54 (m, 2H), 7.34 – 7.30 (m, 2H), 7.21 – 7.16 (m, 1H), 5.51 (t, *J* = 1.8 Hz, 1H), 3.02 (dd, *J* = 16.7, 1.7 Hz, 1H), 2.63 (dd, *J* = 16.6, 1.8 Hz, 1H), 1.68 – 1.59 (m, 3H), 1.31 (s, 3H), 1.29 – 1.21 (m, 3H), 1.15 – 1.04 (m, 3H), 0.98 – 0.87 (m, 2H) ppm.

^{13}C-NMR (151 MHz, $CDCl_3$): δ = 180.0, 146.9, 134.3, 128.6, 128.4, 126.7, 104.5, 46.6, 44.1, 37.2, 28.1, 27.1, 26.3(4), 26.3(0), 26.4, 22.4 ppm.

HRMS (ESIpos) m/z: Calcd for $C_{18}H_{23}O_2$ 271.1692, Found 271.1690.

IR (cm^{-1}): 2926, 2853, 1731, 1450, 1275, 1260, 1182, 1062, 933, 755.

(*Z*)-5-Benzylidene-3-(4-fluorobutyl)-3-methyldihydrofuran-2(3H)-one

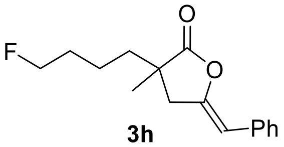

3h Following the general procedure **B**, using **1h** (32.4 mg) and pentane/Et_2O (98:2 v/v) as an eluent, the target compound **3h** was obtained as a colorless oil (29.2 mg, 0.111 mmol, 56%).

^{1}H NMR (600 MHz, $CDCl_3$): δ = 7.57 – 7.54 (m, 2H), 7.34 – 7.30 (m, 2H), 7.22 – 7.19 (m, 1H), 5.55 (t, *J* = 1.7 Hz, 1H), 4.50 – 4.46 (m, 1H), 4.42 – 4.39 (m, 1H), 2.95 (dd, *J* = 16.2, 1.7 Hz, 1H), 2.78 (dd, *J* = 16.2, 1.7 Hz, 1H), 1.75 – 1.66 (m, 4H), 1.61 – 1.54 (m, 1H), 1.43 – 1.37 (m, 1H), 1.35 (s, 3H) ppm.

^{13}C NMR (151 MHz, $CDCl_3$): δ = 179.7, 146.2, 134.1, 128.6, 128.5, 126.9, 105.3, 83.7 (d, *J* = 165.0 Hz), 42.9, 39.9, 37.5, 30.6 (d, *J* = 19.8 Hz), 23.4, 20.5 (d, *J* = 4.9 Hz) ppm.

^{19}F NMR (471 MHz, $CDCl_3$): δ = -219.25 (s) ppm.

HRMS (ESIpos) m/z: Calcd for $C_{16}H_{20}O_2F$ 263.1441, Found 263.1438.

IR (cm^{-1}): 2937, 1835, 1750, 1630, 1487, 1238, 1147, 1052, 913, 815.

(*Z*)-5-benzylidene-3-(4-chlorobutyl)-3-methyldihydrofuran-2(3H)-one

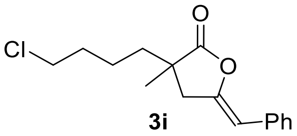

3i Following the general procedure **B**, using **1i** (35.7 mg) and pentane/Et_2O (98:2 v/v) as an eluent, the target compound **3i** was obtained as a colorless oil (24.5 mg, 87.8 µmol, 44%).

^{1}H NMR (600 MHz, $CDCl_3$): δ = 7.56 – 7.53 (m, 2H), 7.34 – 7.31 (m, 2H), 7.22 – 7.19 (m, 1H), 5.55 (t, *J* = 1.7 Hz, 1H), 3.55 – 3.52 (m, 2H), 2.96 (dd, *J* = 16.2, 1.7 Hz, 1H), 2.78 (dd, *J* = 16.1, 1.7 Hz, 1H), 1.82 – 1.77 (m, 2H), 1.68 – 1.61 (m, 3H), 1.46 – 1.38 (m, 1H), 1.35 (s, 3H).

^{13}C NMR (151 MHz, $CDCl_3$): δ = 179.7, 146.2, 134.0, 128.6, 128.5, 126.9, 105.4, 44.6, 42.9, 39.9, 37.0, 32.6, 23.4, 21.9 ppm.

HRMS (ESIpos) m/z: Calcd for $C_{16}H_{20}O_2Cl^{35}$ 279.1146, Found 279.1143.

IR (cm^{-1}): 2929, 1796, 1687, 1449, 1276, 1260, 1063, 944, 751.

(Z)-5-benzylidene-3-methyl-3-(4,4,4-trifluorobutyl)dihydrofuran-2(3H)-one

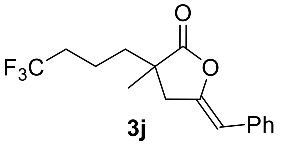

3j Following the general procedure **B**, using **1j** (39.6 mg) and pentane/Et_2O (99:1 v/v) as an eluent, the target compound **3j** was obtained as a colorless oil (24.9 mg, 83.4 µmol, 42%).

^{1}H NMR (600 MHz, $CDCl_3$): δ = 7.57 – 7.54 (m, 2H), 7.35 – 7.31 (m, 2H), 7.23 – 7.20 (m, 1H), 5.57 (t, *J* = 1.7 Hz, 1H), 2.94 (dd, *J* = 16.1, 1.7 Hz, 1H), 2.80 (dd, *J* = 16.1, 1.7 Hz, 1H), 2.15 – 2.06 (m, 2H), 1.74 – 1.71 (m, 3H), 1.56 – 1.51 (m, 1H) 1.36 (s, 3H).

^{13}C-NMR (126 MHz, $CDCl_3$): δ = 179.2, 145.8, 133.9, 128.6, 128.5, 127.0, 126.8 (q, *J* = 274.5 Hz), 105.7, 42.8, 40.0, 36.7, 33.9 (q, *J* = 28.9 Hz), 23.2, 17.4 ppm.

^{19}F NMR (471 MHz, $CDCl_3$): δ = -66.7 (s) ppm.

HRMS (ESIpos) m/z: Calcd for $C_{16}H_{18}O_2F_3$ 299.1253, Found 299.1248.

IR (cm^{-1}): 2963, 1794, 1691, 1460, 1363, 1074, 935, 825.

(Z)-(5-benzylidene-3-methyl-2-oxotetrahydrofuran-3-yl)methyl acetate

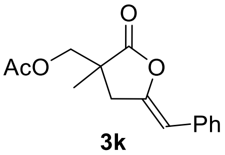

3k Following the general procedure **B**, using **1k** (32.0 mg) and pentane/Et_2O (85:15 v/v) as an eluent, the target compound **3k** was obtained as a colorless oil (17.9 mg, 68.7 µmol, 35%).

^{1}H-NMR (500 MHz, $CDCl_3$): δ = 7.57 – 7.53 (m, 2H), 7.35 – 7.31 (m, 2H), 7.24 – 7.20 (m, 1H), 5.57 (t, *J* = 1.7 Hz, 1H), 4.24 (d, *J* = 11.1 Hz, 1H), 4.19 (d, *J* = 11.1 Hz, 1H), 3.15 (dd, *J* = 16.3, 1.9 Hz, 1H), 2.79 (dd, *J* = 16.3, 1.6 Hz, 1H), 2.03 (s, 3H), 1.36 (s, 3H) ppm.

^{13}C-NMR (126 MHz, $CDCl_3$): δ = 177.4, 170.7, 145.5, 133.8, 128.6, 128.5, 127.0, 105.6, 67.2, 43.5, 37.4, 20.8, 20.6 ppm.

HRMS (ESIpos) m/z: Calcd for $C_{15}H_{17}O_4$ 261.1121, Found 261.1116

IR (cm^{-1}): 2927, 1741, 1456, 1375, 1228, 1040, 752, 699.

(*Z*)-5-benzylidene-3-methyl-3-phenethyldihydrofuran-2(3H)-one

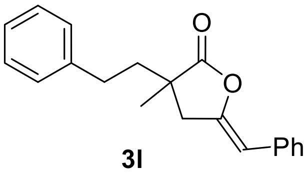
3l

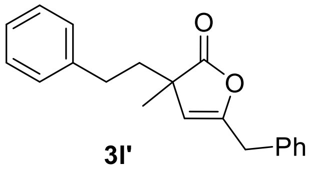
3l'

Following the general procedure **B**, using **1l** (38.4 mg) and pentane/Et_2O (98:2 v/v) as an eluent, the target compound was obtained as a colorless oil (26.7 mg, 91.3 µmol, 46%, **3l : 3l′** = 10:1).

^{1}H-NMR (500 MHz, $CDCl_3$): δ = δ 7.59 – 7.56 (m, $2H^{3l}$ + $2H^{3l'}$), 7.36 – 7.31 (m, $2H^{3l}$ + $2H^{3l'}$), 7.30 – 7.26 (m, $2H^{3l}$ + $2H^{3l'}$), 7.24 – 7.17 (m, $4H^{3l}$ + $4H^{3l'}$), 5.57 (t, *J* = 1.7 Hz, $1H^{3l}$), 5.04 (t, *J* = 1.5 Hz, $1H^{3l'}$), 3.64 (d, *J* = 1.4 Hz, $2H^{3l'}$), 3.01 (dd, *J* = 16.2, 1.7 Hz, $1H^{3l}$), 2.83 (dd, *J* = 16.2, 1.7 Hz, $1H^{3l}$), 2.80 – 2.72 (m, $1H^{3l}$), 2.65 – 2.54 (m, $1H^{3l}$ + $1H^{3l'}$), 2.47 – 2.39 (m, $1H^{3l'}$), 2.06 – 1.91 (m, $2H^{3l}$ + $1H^{3l'}$), 1.87 – 1.81 (m, $1H^{3l'}$), 1.41 (s, $3H^{3l}$), 1.32 (s, $3H^{3l'}$) ppm.

^{13}C-NMR (126 MHz, $CDCl_3$): δ = 181.9 (3l′), 179.5(3l), 153.9 (3l′), 146.2 (3l), 141.2 (3l′), 141.0 (3l), 135.4 (3l′), 134.0 (3l), 129.2 (3l′), 128.8 (3l′), 128.7 (3l), 128.6 (3l), 128.5 (3l), 128.4 (3l), 127.2 (3l′), 126.9 (3l), 126.4 (3l), 126.2 (3l′), 110.0 (3l′), 105.4 (3l), 49.0 (3l′), 43.0 (3l), 40.1 (3l), 39.9 (3l), 34.9 (3l′), 31.5 (3l′), 31.0 (3l), 29.8 (3l′), 23.6 (3l′), 23.4 (3l) ppm.

HRMS (EI) m/z: Calcd for $C_{20}H_{20}O_2$ 292.1463, Found 292.1463.

IR (cm^{-1}): 3025, 1794, 1689, 1494, 1453, 1275, 1260, 1055, 940, 750.

(*Z*)-5-benzylidene-3-(4-fluorophenethyl)-3-methyldihydrofuran-2(3H)-one

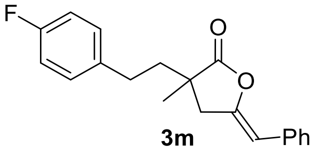
3m

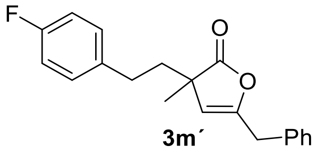
3m′

Following the general procedure **B**, using **1m** (42.0 mg) and pentane/Et_2O (98:2 v/v) as an eluent, the target compound was obtained as a colorless oil (37.5 mg, 0.120 mmol, 60%, **3m : 3m′** = 20:1).

^{1}H-NMR (500 MHz, $CDCl_3$): δ = 7.58 – 7.55 (m, $2H^{3m}$ + $2H^{3m'}$), 7.36 – 7.31 (m, $2H^{3m}$ + $2H^{3m'}$), 7.24 – 7.19 (m, $1H^{3m}$ + $1H^{3m'}$), 7.15 – 7.10 (m, $2H^{3m}$ + $2H^{3m'}$), 6.99 – 6.94 (m, $2H^{3m}$ + $2H^{3m'}$), 5.57 (t, *J* = 1.7 Hz, $1H^{3m}$), 5.02 (t, *J* = 1.5 Hz, $1H^{3m'}$), 3.63 (d, *J* = 1.3 Hz, $1H^{3m'}$), 3.00 (dd, *J* = 16.2, 1.7 Hz, $1H^{3m}$), 2.83 (dd, *J* = 16.2, 1.7 Hz, $1H^{3m}$), 2.77 – 2.69 (m, $1H^{3m}$ + $1H^{3m'}$), 2.61 – 2.53 (m, $1H^{3m}$ + $1H^{3m'}$),, 1.97 – 1.91 (m, $2H^{3m}$ + $2H^{3m'}$), 1.40 (s, $3H^{3m}$), 1.31 (s, $3H^{3m'}$) ppm.

^{13}C-NMR (151 MHz, CDCl$_3$): δ = 181.8 (3m´), 179.4 (3m), 161.6 (d, *J* = 244.1 Hz, 3m), 161.4 (d, *J* = 244.1 Hz, 3m´), 154.0 (3m´), 146.1 (3m), 136.8 (d, *J* = 26.2 Hz, 3m´), 136.7 (d, *J* = 26.2 Hz, 3m), 135.3 (3m´), 134.0 (3m), 129.8 (d, *J* = 7.8 Hz, 3m), 129.2 (3m´), 128.8 (3m´), 128.6 (3m), 128.5 (3m), 127.3 (3m´), 126.9 (3m), 115.5 (d, *J* = 21.2 Hz, 3m), 115.3 (d, *J* = 21.2 Hz, 3m´), 109.8 (3m´), 105.5 (3m), 48.9 (3m´), 43.0 (3m), 40.1 (3m), 40.0 (3m), 39.9 (3m´), 34.9 (3m´), 30.7 (3m´), 30.2 (3m), 23.7 (3m´), 23.5 (3m) ppm.

^{19}F NMR (471 MHz, CDCl$_3$): δ = -117.5 (s, 3k), -117.7 (s, 3k´) ppm.

HRMS (ESIpos) m/z: Calcd for $C_{12}H_{20}O_2F$ 311.1441, Found 313.1437.

IR (cm^{-1}): 2924, 1795, 1690, 1509, 1275, 1260, 1220, 1056, 941, 825, 750.

(*Z*)-3-benzyl-5-benzylidene-3-methyldihydrofuran-2(3H)-one

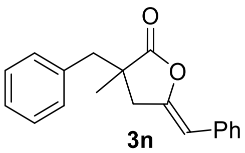

3n

Following the general procedure **B**, using **1n** (35.6 mg) and pentane/Et_2O (98:2 v/v) as an eluent, the target compound **3n** was obtained as a colorless oil (22.5 mg, 80.8 µmol, 40%).

^{1}H-NMR (600 MHz, CDCl$_3$): δ = 7.49 – 7.47 (m, 2H), 7.32 – 7.27 (m, 4H), 7.26 – 7.23 (m, 1H), 7.20 – 7.17 (m, 3H), 5.44 (t, *J* = 1.7 Hz, 1H), 3.10 (d, *J* = 13.7 Hz, 1H), 3.03 (dd, *J* = 16.0, 1.7 Hz, 1H), 2.83 (d, *J* = 13.7 Hz, 1H), 2.61 (dd, *J* = 16.0, 1.7 Hz, 1H), 1.37 (s, 3H) ppm.

^{13}C-NMR (126 MHz, CDCl$_3$): δ = 179.5, 146.1, 136.1, 134.0, 130.3, 128.7, 128.5, 128.5, 127.4, 126.8, 105.3, 44.5, 43.0, 38.8, 23.9 ppm.

HRMS (EI) m/z: Calcd for $C_{19}H_{18}O_2$ 278.1306, Found 278.1305.

IR (cm^{-1}): 3026, 1794, 1687, 1495, 1450,1225, 1180, 1046, 955, 753.

(*Z*)-methyl 4-((5-benzylidene-3-methyl-2-oxotetrahydrofuran-3-yl)methyl)benzoate

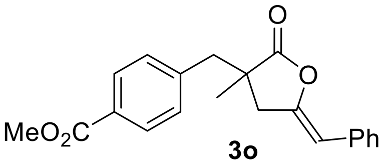

Following the general procedure **B**, using **1o** (47.2 mg) and pentane/Et_2O (88:12 v/v) as an eluent, the target compound **3o** was obtained as a colorless oil (27.4 mg, 81.5 µmol, 41%).

^{1}H-NMR (500 MHz, $CDCl_3$): δ = δ = 7.98 – 7.94 (m, 2H), 7.48 – 7.45 (m, 2H), 7.30 – 7.25 (m, 4H), 7.21 – 7.17 (m, 1H), 5.44 (t, *J* = 1.7 Hz, 1H), 3.89 (s, 3H), 3.14 (d, *J* = 13.5 Hz, 1H), 2.99 (dd, *J* = 16.0, 1.8 Hz, 1H), 2.89 (d, *J* = 13.5 Hz, 1H), 2.63 (dd, *J* = 16.0, 1.5 Hz, 1H), 1.37 (s, 3H) ppm.

^{13}C-NMR (126 MHz, $CDCl_3$): δ = 179.1, 166.9, 145.7, 141.4, 133.8, 130.3, 129.9, 129.4, 128.5, 128.5, 126.9, 105.7, 52.2, 44.5, 42.9, 38.9, 23.9 ppm.

HRMS (EI) m/z: Calcd for $C_{21}H_{20}O_4$ 336.1361, Found 336.1362.

IR (cm^{-1}): 2962, 1796, 1598, 1362, 1248, 1055, 931, 878.

(*Z*)-5-benzylidene-3-methyl-3-(4-nitrobenzyl)dihydrofuran-2(3H)-one

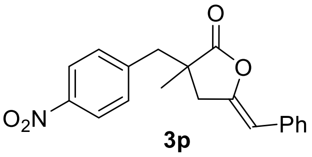

Following the general procedure **B**, using **1p** (44.6 mg) and pentane/Et_2O (88:12 v/v) as an eluent, the target compound **3p** was obtained as a colorless oil (27.8 mg, 85.9 µmol, 43%).

^{1}H-NMR (500 MHz, $CDCl_3$): δ = 8.16 – 8.12 (m, 2H), 7.48 – 7.42 (m, 2H), 7.39 – 7.35 (m, 2H), 7.31 – 7.27 (m, 2H), 7.22 – 7.17 (m, 1H), 5.46 (t, *J* = 1.7 Hz, 1H), 3.20 (d, *J* = 13.6 Hz, 1H), 2.99 – 2.91 (m, 2H), 2.70 (dd, *J* = 15.9, 1.5 Hz, 1H), 1.40 (s, 3H) ppm.

^{13}C-NMR (126 MHz, $CDCl_3$): δ = 178.6, 147.4, 145.2, 143.7, 133.6, 131.1, 128.6, 128.4, 127.1, 123.8, 106.0, 44.5, 42.8, 38.9, 24.0 ppm.

HRMS (EI) m/z: Calcd for $C_{19}H_{17}NO_4$ 323.1157, Found 323.1157.

IR (cm^{-1}): 2929, 1793, 1687, 1517, 1344, 1226, 1052, 852.

(*Z*)-5-benzylidene-3-methyl-3-(4-((trifluoromethyl)thio)benzyl)dihydrofuran-2(3H)-one

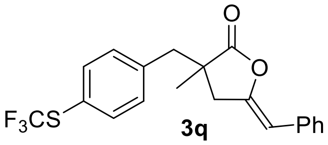

Following the general procedure **B**, using **1q** (35.6 mg) and pentane/Et_2O (98:2 v/v) as an eluent, the target compound **3q** was obtained as a colorless oil (33.4 mg, 88.2 µmol, 44%).

^{1}H-NMR (500 MHz, $CDCl_3$): δ = δ 7.60 – 7.56 (m, 2H), 7.48 – 7.44 (m, 2H), 7.32 – 7.28 (m, 2H), 7.27 – 7.23 (m, 2H), 7.21 – 7.17 (m, 1H), 5.45 (t, *J* = 1.7 Hz, 1H), 3.12 (d, *J* = 13.6 Hz, 1H), 2.98 (dd, *J* = 16.0, 1.8 Hz, 1H), 2.86 (d, *J* = 13.6 Hz, 1H), 2.66 (dd, *J* = 16.0, 1.6 Hz, 1H), 1.38 (s, 3H) ppm.

^{13}C-NMR (126 MHz, $CDCl_3$): δ = 179.0, 145.6, 139.4, 136.6, 133.7, 131.4, 130.2 (q, *J* = 308.0 Hz), 128.6, 128.5, 127.0, 123.5, 105.8, 44.5, 42.7, 38.9, 23.9 ppm.

HRMS (EI) m/z: Calcd for $C_{20}H_{17}F_3O_2S$ 378.0901, Found 378.0900.

IR (cm^{-1}): 2923, 1793, 1690, 1514, 1450, 1178, 1057, 754.

(*Z*)-5-benzylidene-3-(3,5-bis(trifluoromethyl)benzyl)-3-methyldihydrofuran-2(3H)-one

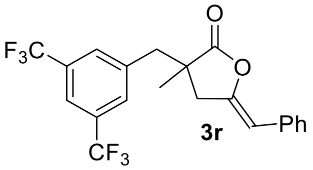

Following the general procedure **B**, using **1r** (62.8 mg) and pentane/Et_2O (96:4 v/v) as an eluent, the target compound **3r** was obtained as a colorless oil (28.1 mg, 89.8 µmol, 45%).

^{1}H-NMR (500 MHz, $CDCl_3$): δ = 7.74 (s, 1H), 7.66 (s, 2H), 7.45 – 7.41 (m, 2H), 7.31 – 7.27 (m, 2H), 7.21 – 7.17 (m, 1H), 5.45 (t, *J* = 1.6 Hz, 1H), 3.20 (d, *J* = 13.8 Hz, 1H), 2.98 (d, *J* = 13.8 Hz, 1H), 2.93 (dd, *J* = 15.8, 1.6 Hz, 1H), 2.74 (dd, *J* = 15.8, 1.6 Hz, 1H), 1.41 (s, 3H) ppm.

^{13}C-NMR (126 MHz, $CDCl_3$): δ = 178.3, 144.9, 138.5, 133.4, 132.0 (q, *J* = 33.2 Hz), 130.3, 128.6, 128.5, 127.1, 121.6, 106.2, 44.4, 42.8, 39.1, 23.8 ppm.

^{19}F NMR (471 MHz, $CDCl_3$): δ = -63.39 ppm.

HRMS (EI) m/z: Calcd for $C_{21}H_{16}F_6O_2$ 414.1054, Found 414.1054.

IR (cm^{-1}): 2928, 1796, 1692, 1376, 1275, 1169, 1126, 1054, 930, 897.

(*Z*)-5-benzylidene-3-methyl-3-phenyldihydrofuran-2(3H)-one

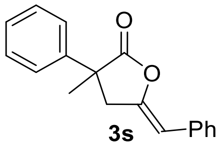

3s Following the general procedure **B**, using **1s** (32.8 mg) and pentane/Et_2O (98:2 v/v) as an eluent, the target compound **3s** was obtained as a colorless oil (18.1 mg, 68.5 µmol, 34%).

^{1}H-NMR (500 MHz, $CDCl_3$): δ = 7.60 – 7.56 (m, 2H), 7.47 – 7.43 (m, 2H), 7.38 – 7.29 (m, 5H), 7.24 – 7.20 (m, 1H), 5.61 (t, *J* = 1.5 Hz, 1H), 3.41 (dd, *J* = 16.0, 1.5 Hz, 2H), 3.17 (dd, *J* = 16.0, 1.8 Hz, 2H), 1.75 (s, 3H) ppm.

^{13}C-NMR (126 MHz, $CDCl_3$): δ = 178.0, 145.8, 141.0, 133.9, 129.9, 129.1, 128.6, 127.8, 127.0, 125.8, 105.5, 47.4, 43.4, 25.4 ppm.

HRMS (EI) m/z: Calcd for $C_{18}H_{16}O_2$ 264.1150, Found 264.1148.

IR (cm^{-1}): 2932, 1810, 1725, 1622, 1420, 1215, 1106, 1018, 954, 837.

(*Z*)-5-benzylidene-3,3-diethyldihydrofuran-2(3H)-one

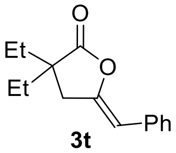

3t

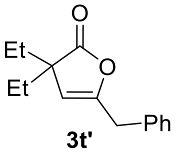

3t' Following the general procedure **B**, using **1t** (26.1 mg) and pentane/Et_2O (98:2 v/v) as an eluent, the target compound was obtained as a colorless oil (29.3 mg, 93.7 µmol, 47%, **3t : 3t´** = 11:1).

^{1}H-NMR (600 MHz, $CDCl_3$): δ = 7.57 – 7.54 (m, $2H^{3t}$ + $2H^{3t'}$), 7.34 – 7.30 (m, $2H^{3t}$ + $2H^{3t'}$), 7.22 – 7.18 (m, $1H^{3t}$ + $1H^{3t'}$), 5.53 (t, *J* = 1.7 Hz, $1H^{3t}$), 4.89 (t, *J* = 1.4 Hz, $1H^{3t'}$), 3.65 (d, *J* = 1.4 Hz, $2H^{3t'}$), 2.86 (d, *J* = 1.8 Hz, $2H^{3t}$), 1.77 – 1.59 (m, $4H^{3t}$ + $4H^{3t'}$) 0.96 (t, *J* = 7.5 Hz, $6H^{3t}$), 0.81 (t, *J* = 7.5 Hz, $6H^{3t'}$) ppm.

^{13}C-NMR (151 MHz, $CDCl_3$): δ = 181.8 (3t´), 179.3 (3t), 154.3 (3t´), 146.9 (3t), 135.7 (3t´), 134.3 (3t), 129.1 (3t´), 128.8 (3t´), 128.6 (3t), 128.4 (3t), 127.1 (3t´), 126.7 (3t), 107.8 (3t´), 104.7 (3t), 55.1 (3t´), 47.7 (3t), 36.7 (3t), 34.9 (3t´), 29.9 (3t´), 29.4 (3t), 9.3 (3t´), 8.8 (3t) ppm.

HRMS (EI) m/z: Calcd for $C_{15}H_{18}O_2$ 230.1306, Found 230.1306.

IR (cm^{-1}): 2967, 1790, 1687, 1458, 1224, 1178, 1074, 946, 754, 694.

(*Z*)-5-benzylidene-3-ethyl-3-isobutyldihydrofuran-2(3H)-one

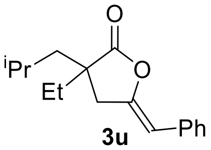

Following the general procedure **B**, using **1u** (31.6 mg) and pentane/Et_2O (98:2 v/v) as an eluent, the target compound **3u** was obtained as a colorless oil (25.7 mg, 99.5 µmol, 50%).

^{1}H-NMR (500 MHz, $CDCl_3$): δ =7.58 – 7.54 (m, 2H), 7.34 – 7.28 (m, 2H), 7.21 – 7.16 (m, 2H), 5.54 (t, *J* = 1.7 Hz, 1H), 2.99 (dd, *J* = 16.7, 1.9 Hz, 1H), 2.83 (dd, *J* = 16.7, 1.6 Hz, 1H), 1.81 – 1.55 (m, 5H), 0.98 – 0.88 (m, 9H) ppm.

^{13}C-NMR (126 MHz, $CDCl_3$): δ = 179.8, 146.9, 134.3, 128.6, 128.4, 126.7, 104.6, 46.7, 44.7, 37.1, 31.2, 25.0, 24.8, 23.0, 8.7 ppm.

HRMS (EI) m/z: Calcd for $C_{17}H_{22}O_2$ 258.1619, Found 258.1620.

IR (cm^{-1}): 2968, 1795, 1683, 1459, 1205, 1071, 935, 750.

(*Z*)-5-benzylidene-3-(3,5-bis(trifluoromethyl)benzyl)-3-ethyldihydrofuran-2(3H)-one

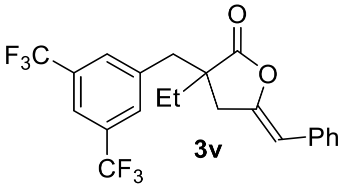

Following the general procedure **B**, using **1v** (65.6 mg) and pentane/Et_2O (98:2 v/v) as an eluent, the target compound **3v** was obtained as a colorless oil (33.8 mg, 78.9 µmol, 40%).

^{1}H-NMR (500 MHz, $CDCl_3$): δ = 7.72 (s, 1H), 7.65 (s, 2H), 7.42 – 7.38 (m, 2H), 7.30 – 7.25 (m, 2H), 7.20 – 7.15 (m, 1H), 5.39 (t, *J* = 1.7 Hz, 1H), 3.22 (d, *J* = 13.7 Hz, 1H), 2.97 (d, *J* = 13.7 Hz, 1H), 2.85 (d, *J* = 1.7 Hz, 2H), 1.90 – 1.81 (m, 1H), 1.79 – 1.68 (m, 1H) 1.05 (t, *J* = 7.4 Hz, 3H) ppm.

^{13}C-NMR (126 MHz, $CDCl_3$): δ = 177.8, 145.3, 138.6, 133.5, 132.0 (q, *J* = 33.2 Hz), 130.3, 128.5, 128.4, 127.0, 121.6, 105.6, 48.8, 41.5, 36.0, 30.5, 8.9 ppm.

^{19}F NMR (471 MHz, $CDCl_3$): δ = -63.4 (s) ppm.

HRMS (EI) m/z: Calcd for $C_{22}H_{18}F_6O_2$ 428.1211, Found 428.1208.

IR (cm^{-1}): 2973, 1794, 1692, 1377, 1275, 1126, 1070, 969.

(*Z*)-5-benzylidene-3-(3-(2,5-dimethylphenoxy)propyl)-3-methyldihydrofuran-2(3H)-one

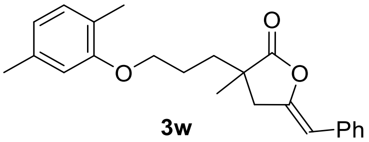

Following the general procedure **B**, using **1w** (65.6 mg) and pentane/Et_2O (98:2 v/v) as an eluent, the target compound **3w** was obtained as a colorless oil (37.0 mg, 0.105 mmol, 53%).

^{1}H-NMR (500 MHz, $CDCl_3$): δ = 7.59 – 7.55 (m, 2H), 7.37 – 7.30 (m, 2H), 7.23 – 7.19 (m, 1H), 7.01 – 6.99 (m, 1H), 6.69 – 6.64 (m, 1H), 6.60 (s, 1H), 5.57 (t, *J* = 1.7 Hz, 1H), 3.97 – 3.94 (m, 2H), 2.99 (dd, *J* = 16.2, 1.7 Hz, 1H), 2.83 (dd, *J* = 16.2, 1.7 Hz, 1H), 2.31 (s, 3H), 2.17 (s, 3H), 1.98 – 1.91 (m, 1H), 1.90 – 1.86 (m, 2H), 1.83 – 1.76 (m, 1H), 1.39 (s, 3H) ppm.

^{13}C-NMR (126 MHz, $CDCl_3$): δ = 179.5, 156.7, 146.1, 136.5, 133.9, 130.4, 128.5, 128.4, 126.7, 123.5, 120.9, 112.0, 105.2, 67.3, 42.6, 40.0, 34.5, 24.7, 23.2, 21.4, 15.8 ppm.

HRMS (ESIpos) m/z: Calcd for $C_{23}H_{27}O_3$ 351.1954, Found 351.1945.

IR (cm^{-1}): 2949, 1797, 1687, 1449, 1275, 1260, 1041, 941, 751.

(*Z*)-3-ethyl-5-(2-fluorobenzylidene)-3-methyldihydrofuran-2(3H)-one

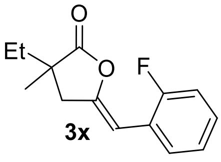

Following the general procedure **C**, using **2b** (61.1 mg) and pentane/Et_2O (98:2 v/v) as an eluent, the target compound **3x** was obtained as a colorless oil (24.3 mg, 0.103 mmol, 52%).

^{1}H NMR (500 MHz, $CDCl_3$): δ = 7.99 – 7.92 (m, 1H), 7.19 – 7.09 (m, 2H), 7.04 – 6.98 (m, 1H), 5.81 (t, *J* = 1.9 Hz, 1H), 2.96 (dd, *J* = 16.3, 1.7 Hz, 1H), 2.79 (dd, *J* = 16.4, 1.8 Hz, 1H), 1.75 – 1.65 (m, 2H), 1.33 (s, 3H), 0.98 (t, *J* = 7.5 Hz, 3H) ppm.

^{13}C NMR (126 MHz, $CDCl_3$): δ = 179.7, 159.3 (d, *J* = 248.2 Hz), 148.0, 130.2, 128.1 (d, *J* = 8.5 Hz), 124.3 (d, *J* = 3.7 Hz), 122.0 (d, *J* = 11.6 Hz), 115.1 (d, *J* = 22.1 Hz), 96.2 (d, *J* = 8.0 Hz), 43.4, 39.7, 30.9, 23.0, 8.9 ppm.

^{19}F NMR (471 MHz, $CDCl_3$): δ = -119.0 ppm.

HRMS (ESIpos) m/z: Calcd for $C_{14}H_{16}O_2F$ 235.1128 , Found 235.1126.

IR (cm^{-1}): 2970, 1797, 1685, 1581, 1255, 1149, 1069, 946, 876.

(*Z*)-5-(2-chlorobenzylidene)-3-ethyl-3-methyldihydrofuran-2(3H)-one

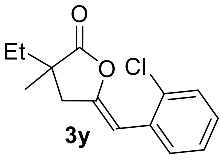

Following the general procedure **C**, using **2c** (69.3 mg) and pentane/Et_2O (98:2 v/v) as an eluent, the target compound **3y** was obtained as a colorless oil (26.2 mg, 0.104 mmol, 52%).

^{1}H NMR (500 MHz, $CDCl_3$): δ = 7.96 (dd, *J* = 7.9, 1.7 Hz, 1H), 7.35 (dd, *J* = 8.0, 1.4 Hz, 1H), 7.26 – 7.22 (m, 1H), 7.15 – 7.10 (m, 1H), 5.97 (t, *J* = 1.8 Hz, 1H), 2.99 (dd, *J* = 16.4, 1.7 Hz, 1H), 2.82 (dd, *J* = 16.4, 1.8 Hz, 1H), 1.77 – 1.64 (m, 2H), 1.34 (s, 3H), 0.98 (t, *J* = 7.5 Hz, 3H) ppm.

^{13}C NMR (126 MHz, $CDCl_3$): δ = 179.6, 148.2, 132.3, 131.9, 130.4, 129.4, 127.9, 127.0, 100.7, 43.4, 39.7, 30.9, 23.0, 8.9 ppm.

HRMS (ESIpos) m/z: Calcd for $C_{14}H_{16}O_2Cl$ 251.0833 , Found 251.0831.

IR (cm^{-1}): 2969, 1793, 1682, 1458, 1260, 1073, 764.

(*Z*)-3-ethyl-5-(3-fluorobenzylidene)-3-methyldihydrofuran-2(3H)-one

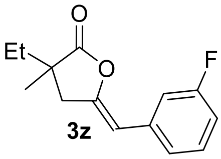

Following the general procedure **C**, using **2d** (61.1 mg) and pentane/Et_2O (98:2 v/v) as an eluent, the target compound **3z** was obtained as a colorless oil (24.3 mg, 0.103 mmol, 52%).

^{1}H NMR (500 MHz, $CDCl_3$): δ = 7.33 – 7.26 (m, 3H), 6.93 – 6.86 (m, 1H), 5.51 (t, *J* = 1.7 Hz, 1H), 2.93 (dd, *J* = 16.4, 1.7 Hz, 1H), 2.76 (dd, *J* = 16.3, 1.7 Hz, 1H), 1.75 – 1.65 (m, 2H), 1.33 (s, 3H), 0.97 (t, *J* = 7.5 Hz, 3H) ppm.

^{13}C NMR (126 MHz, $CDCl_3$): δ = 179.5, 163.0 (d, *J* = 244.6 Hz), 147.6, 136.3 (d, *J* = 8.4 Hz), 129.9 (d, *J* = 8.4 Hz), 124.2, 115.1 (d, *J* = 22.7 Hz), 113.6 (d, *J* = 21.2 Hz), 104.1, 43.3, 39.5, 30.9, 23.0, 8.9 ppm.

HRMS (ESIpos) m/z: Calcd for $C_{14}H_{16}O_2F$ 235.1128 , Found 235.1128.

IR (cm^{-1}): 2971, 1794, 1686, 1490, 1275, 1616, 1417, 1322, 1275, 1109, 1064, 934.

(*Z*)-5-(3-chlorobenzylidene)-3-ethyl-3-methyldihydrofuran-2(3H)-one

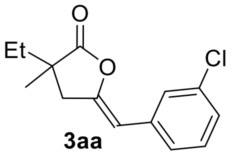

Following the general procedure **C**, using **2e** (69.3 mg) and pentane/Et_2O (98:2 v/v) as an eluent, the target compound **3aa** was obtained as a colorless oil (24.5 mg, 97.7 µmol, 49%).

^{1}H NMR (500 MHz, $CDCl_3$): δ = 7.53 – 7.51 (m, 1H), 7.44 – 7.41 (m, 1H),, 7.24 (t, *J* = 7.9 Hz, 1H), 7.18 – 7.15 (m, 1H), 5.47 (t, *J* = 1.7 Hz, 1H), 2.93 (dd, *J* = 16.4, 1.7 Hz, 1H), 2.76 (dd, *J* = 16.3, 1.7 Hz, 1H), 1.75 – 1.65 (m, 2H), 1.33 (s, 3H), 0.97 (t, *J* = 7.5 Hz, 3H) ppm.

^{13}C NMR (126 MHz, $CDCl_3$): δ = 179.5, 147.8, 135.9, 134.4, 129.8, 128.2, 126.8, 126.6, 103.8, 43.3, 39.6, 30.9, 23.0, 8.9 ppm.

HRMS (ESIpos) m/z: Calcd for $C_{14}H_{16}O_2Cl$ 251.0833 , Found 251.0830.

IR (cm^{-1}): 2970, 1791, 1680, 1457, 1275, 1260, 1075, 935, 750.

(*Z*)-3-ethyl-5-(4-fluorobenzylidene)-3-methyldihydrofuran-2(3H)-one

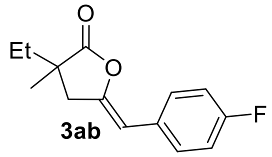

Following the general procedure **C**, using **2f** (61.1 mg) and pentane/Et_2O (98:2 v/v) as an eluent, the target compound **3ab** was obtained as a colorless oil (25.3 mg, 0.108 mmol, 54%).

^{1}H NMR (500 MHz, $CDCl_3$): δ = 7.54 – 7.49 (m, 2H), 7.03 – 6.97 (m, 2H), 5.50 (t, *J* = 1.7 Hz, 1H), 2.92 (dd, *J* = 16.2, 1.7 Hz, 1H), 2.75 (dd, *J* = 16.1, 1.8 Hz, 1H), 1.75 – 1.64 (m, 2H), 1.32 (s, 3H), 0.97 (t, *J* = 7.5 Hz, 3H) ppm.

^{13}C NMR (126 MHz, $CDCl_3$): δ = 179.7, 161.6 (d, *J* = 246.4 Hz), 146.1, 130.3, 130.0 (d, *J* = 7.8 Hz), 115.4 (d, *J* = 21.2 Hz), 104.0, 43.4, 39.5, 30.9, 23.0, 8.9 ppm.

HRMS (ESIpos) m/z: Calcd for $C_{14}H_{16}O_2F$ 235.1128 , Found 235.1128.

IR (cm^{-1}): 2972, 1796, 1691, 1603, 1508, 1459, 1275, 1260, 1229, 1071, 936, 849.

(*Z*)-5-(4-chlorobenzylidene)-3-ethyl-3-methyldihydrofuran-2(3H)-one

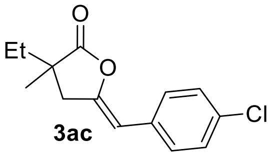

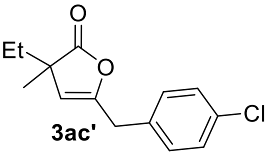

Following the general procedure **C**, using **2g** (69.3 mg) and pentane/Et_2O (98:2 v/v) as an eluent, the target compound was obtained as a colorless oil (26.2 mg, 0.104 mmol, 52%, **3ac : 3ac**´ = 12:1).

^{1}H NMR (500 MHz, $CDCl_3$): δ = 7.49 – 7.46 (m, $2H^{3ac}$), 7.29 – 7.26 (m, $2H^{3ac}$ + $2H^{3ac´}$), 7.20 – 7.17 (m, $2H^{3ac´}$), 5.49 (t, *J* = 1.7 Hz, $1H^{3ac}$), 4.99 (t, *J* = 1.4 Hz, $1H^{3ac´}$), 3.59 (d, *J* = 1.4 Hz, $2H^{3ac´}$), 2.92 (dd, *J* = 16.3, 1.7 Hz, 1 H^{3ac}), 2.75 (dd, *J* = 16.3, 1.8 Hz, 1 H^{3ac}), 1.75 – 1.64 (m, $2H^{3ac}$ + $2H^{3ac´}$), 1.32 (s, $3H^{3ac}$), 1.26 (s, $3H^{3ac´}$), 0.97 (t, *J* = 7.5 Hz, $3H^{3ac}$), 0.81 (t, *J* = 7.5 Hz, $3H^{3ac´}$) ppm.

^{13}C NMR (126 MHz, $CDCl_3$): δ = 179.6 (3ac), 153.1 (3ac´), 147.0 (3ac), 134.0 (3ac´), 132.7 (3ac), 132.3 (3ac), 130.5 (3ac´), 129.7 (3ac), 128.9 (3ac´), 128.7 (3ac), 110.1(3ac´), 103.9 (3ac), 49.6 (3ac´), 43.3 (3ac), 39.5 (3ac), 34.2 (3ac´), 31.1 (3ac´), 30.9 (3ac), 23.1 (3ac´), 23.0 (3ac), 9.4 (3ac´), 8.9 (3ac) ppm.

HRMS (ESIpos) m/z: Calcd for $C_{14}H_{16}O_2Cl$ 251.0833 , Found 251.0829.

IR (cm^{-1}): 2937, 1765, 1654, 1415, 1215, 1260, 1178, 1054, 912, 837.

(*Z*)-5-(4-bromobenzylidene)-3-ethyl-3-methyldihydrofuran-2(3H)-one

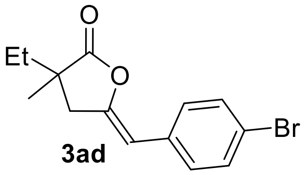

3ad

Following the general procedure **C**, using **2h** (91.5 mg) and pentane/Et_2O (98:2 v/v) as an eluent, the target compound **3ad** was obtained as a colorless oil (18.7 mg, 63.3 µmol, 32%).

^{1}H NMR (500 MHz, $CDCl_3$): δ = 7.44 – 7.40 (m, 4H), 5.47 (t, *J* = 1.7 Hz, 1H), 2.91 (dd, *J* = 16.3, 1.7 Hz, 1H), 2.74 (dd, *J* = 16.3, 1.8 Hz, 1H), 1.74 – 1.63 (m, 2H), 1.32 (s, 3H), 0.97 (t, *J* = 7.5 Hz, 3H) ppm.

^{13}C NMR (126 MHz, $CDCl_3$): δ = 179.6, 147.2, 133.1, 131.7, 130.0, 120.5, 104.0, 43.3, 39.6, 30.9, 23.0, 8.9 ppm.

HRMS (ESIpos) m/z: Calcd for $C_{14}H_{16}O_2Br$ 295.0328 , Found 295.0325.

IR (cm^{-1}): 2922, 1794, 1689, 1450, 1260, 1050, 801.

(*Z*)-3-ethyl-3-methyl-5-(4-(trifluoromethyl)benzylidene)dihydrofuran-2(3H)-one

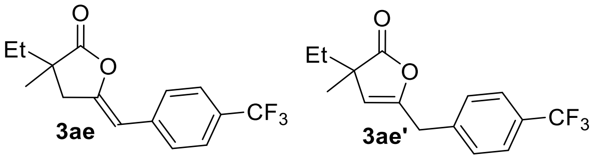

3ae **3ae'**

Following the general procedure **C**, using **2i** (86.1 mg) and pentane/Et_2O (96:4 v/v) as an eluent, the target compound was obtained as a colorless oil (34.5 mg, 0.121 mmol, 61%, **3ae : 3ae´** = 3:1).

^{1}H NMR (500 MHz, $CDCl_3$): δ = 7.64 (d, *J* = 8.2 Hz, $2H^{3ae}$), 7.59 (d, *J* = 8.5 Hz, $2H^{3ae´}$), 7.55 (d, *J* = 8.7 Hz, $2H^{3ae}$), 7.38 (d, *J* = 7.9 Hz, $2H^{3ae´}$), 5.57 (t, *J* = 1.7 Hz, $1H^{3ae}$), 5.04 (t, *J* = 1.4 Hz, $1H^{3ae´}$), 3.68 (s, $2H^{3ae´}$), 2.96 (dd, *J* = 16.5, 1.7 Hz, $1H^{3ae}$), 2.79 (dd, *J* = 16.5, 1.8 Hz, $1H^{3ae}$), 1.73 – 1.67 (m, $2H^{3ae}$), 1.61 – 1.54 (m, $2H^{3ae´}$), 1.34 (s, $3H^{3ae}$), 1.27 (s, $3H^{3ae´}$), 0.98 (t, *J* = 7.5 Hz, $3H^{3ae}$), 0.82 (t, *J* = 7.5 Hz, $3H^{3ae´}$) ppm.

^{13}C NMR (126 MHz, $CDCl_3$): δ = 181.9 (3ae´), 179.4 (3ae), 152.6 (3ae´), 148.7 (3ae), 139.7 (3ae´), 137.7 (3ae), 129.5 (3ae´), 128.9 (q, *J* = 271.6 Hz, 3ae´), 128.7 (q, *J* = 271.6 Hz, 3ae), 128.5 (3ae), 125.7 (d, *J* = 3.9 Hz, 3ae´), 125.4 (d, *J* = 3.9 Hz, 3ae), 110.5 (3ae´), 103.8 (3ae), 49.6 (3ae´), 43.3 (3ae), 39.6 (3ae), 34.7 (3ae´), 31.1 (3ae´), 31.0 (3ae), 23.1 (3ae´), 23.0 (3ae), 9.4 (3ae´), 8.9 (3ae) ppm.

HRMS (ESIpos) m/z: Calcd for $C_{15}H_{16}O_2F_3$ 285.1096, Found 285.1094.

IR (cm^{-1}): 2973, 1795, 1686, 1616, 1322, 1163, 1064, 934, 749.

(*Z*)-methyl 4-((4-ethyl-4-methyl-5-oxodihydrofuran-2(3H)-ylidene)methyl)benzoate

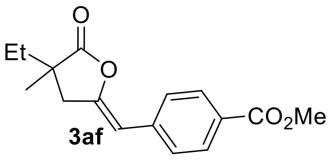

Following the general procedure **C**, using **2j** (81.1 mg) and pentane/Et_2O (85:15 v/v) as an eluent, the target compound **3af** (26.5 mg, 94.0 µmol, 47%) and **3af**′(8.5 mg, 30.0 µmol, 15%) were obtained as colorless oil.

^{1}H NMR (500 MHz, $CDCl_3$): δ = 7.99 – 7.96 (m, 2H), 7.62 – 7.59 (m, 2H), 5.57 (t, *J* = 1.7 Hz, 2H), 3.90 (s, 3H), 2.96 (dd, *J* = 16.5, 1.7 Hz, 1H), 2.78 (dd, *J* = 16.5, 1.7 Hz, 1H), 1.76 – 1.74 (m, 2H), 1.33 (s, 3H), 0.97 (t, *J* = 7.5 Hz, 3H) ppm.

^{13}C NMR (126 MHz, $CDCl_3$): δ = 179.5, 167.1, 148.7, 138.8, 129.9, 128.3, 128.0, 104.3, 52.2, 43.3, 39.7, 31.0, 23.0, 8.9 ppm.

HRMS (ESIpos) m/z: Calcd for $C_{16}H_{19}O_4$ 275.1277, Found 275.1273.

IR (cm^{-1}): 2970, 1799, 1716, 1677, 1606, 1434, 1275, 1181, 1068, 937, 867.

methyl 4-((4-ethyl-4-methyl-5-oxo-4,5-dihydrofuran-2-yl)methyl)benzoate

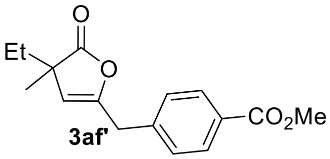

^{1}H NMR (500 MHz, $CDCl_3$): δ = 8.02 – 7.98 (m, 2H), 7.34 – 7.32 (m, 2H), 3.92 (s, 3H), 3.68 (s, 2H), 1.77 – 1.68 (m, 1H), 1.60 – 1.56 (m, 1H), 1.26 (s, 3H), 0.81 (t, *J* = 7.5 Hz, 3H) ppm.

^{13}C NMR (126 MHz, $CDCl_3$): δ = 181.9, 167.0, 152.7, 140.9, 130.1, 129.2, 128.6, 110.4, 52.3, 49.6, 34.8, 31.1, 23.1, 9.4 ppm.

HRMS (ESIpos) m/z: Calcd for $C_{16}H_{19}O_4$ 275.1277, Found 275.1273.

IR (cm^{-1}): 2971, 1797, 1718, 1606, 1435, 1275, 1180, 1105, 1069, 1018, 935, 867.

(*Z*)-3-ethyl-3-methyl-5-(4-nitrobenzylidene)dihydrofuran-2(3H)-one

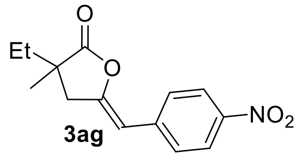

Following the general procedure **C** [using **L6** (5.4 mg, 20 µmmol, 20 mol%) instead of **L10**], using **2k** (74.6 mg) and pentane/Et_2O (92:8 v/v) as an eluent, **3ag** (26.8 mg, 0.102 mmol, 51%) and **3ag´**(5.4 mg, 20.0 µmol, 10%) were obtained as colorless oil.

^{1}H NMR (600 MHz, $CDCl_3$): δ = 8.16 – 8.12 (m, 2H), 7.69 – 7.66 (m, 2H), 5.61 (t, *J* = 1.8 Hz, 1H), 2.99 (dd, *J* = 16.7, 1.7 Hz, 1H), 2.82 (dd, *J* = 16.7, 1.8 Hz, 1H), 1.78 – 1.65 (m, 2H), 1.35 (s, 3H), 0.98 (t, *J* = 7.5 Hz, 3H) ppm.

^{13}C NMR (151 MHz, $CDCl_3$): δ = 179.1, 150.6, 145.9, 140.9, 128.8, 123.9, 103.3, 43.2, 39.8, 31.0, 23.0, 8.9 ppm.

HRMS (ESIpos) m/z: Calcd for $C_{14}H_{16}O_4N$ 262.1073, Found 262.1072.

IR (cm^{-1}): 2972, 1794, 1520, 1347, 1275, 1260, 1080, 749.

3-ethyl-3-methyl-5-(4-nitrobenzyl)furan-2(3H)-one

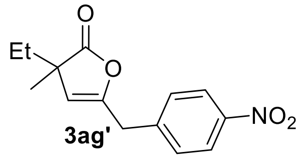

^{1}H NMR (600 MHz, $CDCl_3$): δ = 8.20 (d, *J* = 8.7 Hz, 2H), 7.44 (d, *J* = 8.9 Hz, 2H), 5.10 (t, *J* = 1.3 Hz, 1H), 3.74 (s, 2H), 1.78 – 1.69 (m, 1H), 1.62 – 1.58 (m, 1H), 1.28 (s, 3H), 0.82 (t, *J* = 7.5 Hz, 3H) ppm.

^{13}C NMR (151 MHz, $CDCl_3$): δ = 181.6, 151.8, 147.4, 143.2, 130.0, 124.1, 111.0, 49.7, 34.7, 31.0, 23.1, 9.4 ppm.

HRMS (ESIpos) m/z: Calcd for $C_{14}H_{16}O_4N$ 262.1073, Found 262.1072.

IR (cm^{-1}): 2950, 1785, 1505, 1418, 1315, 1225, 1120, 934, 742.

(Z)-N-(4-((4-ethyl-4-methyl-5-oxodihydrofuran-2(3H)-ylidene)methyl)phenyl)-2,2,2-trifluoroacetamide

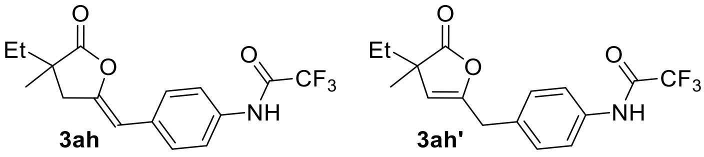

Following the general procedure **C**, using **2l** (107.6 mg) and pentane/Et_2O (85:15 v/v) as an eluent, the target compound was obtained as a colorless solid (40.1 mg, 0.123 mmol, 61%, **3ah : 3ah´** = 4:1).

^{1}H NMR (600 MHz, $(CD_3)_2CO$): δ = 7.72 – 7.69 (m, $2H^{3ah}$ + $2H^{3ah´}$), 7.60 – 7.57 (m, $2H^{3ah}$ + $2H^{3ah´}$), 5.65 (t, *J* = 1.7 Hz, $1H^{3ah}$), 5.25 (t, *J* = 1.3 Hz, $1H^{3ah´}$), 3.69 (s, $2H^{3ah´}$), 3.04 (dd, *J* = 16.4, 1.7 Hz, $1H^{3ah}$), 2.89 (dd, *J* = 16.4, 1.8 Hz, $1H^{3ah}$), 1.72 – 1.60 (m, $2H^{3ah}$ + $2H^{3ah´}$), 1.31 (s, $3H^{3ah}$), 1.23 (s, $1H^{3ah´}$), 0.95 (t, *J* = 7.5 Hz, $3H^{3ah}$), 0.78 (t, *J* = 7.5 Hz, $1H^{3ah´}$) ppm.

^{13}C NMR (600 MHz, $(CD_3)_2CO$): δ = 182.1 (3ah´), 179.9 (3ah), 155.6 (q, *J* = 37.0 Hz, 3ah´), 155.5 (q, *J* = 37.0 Hz, 3ah), 154.2 (3ah´), 148.4 (3ah), 136.2 (3ah´), 135.6 (3ah), 134.6 (3ah´), 133.2 (3ah), 130.4 (3ah´), 129.6 (3ah), 121.8 (3ah´), 121.6 (3ah), 117.0 (q, *J* = 288.0 Hz, 3ah´), 117.2 (q, *J* = 288.0 Hz, 3ah), 110.7 (3ah´), 104.1 (3ah), 50.0 (3ah´), 43.8 (3ah), 39.8 (3ah), 34.4 (3ah´), 31.5 (3ah´), 31.4 (3ah), 23.3 (3ah´), 22.8 (3ah), 9.5 (3ah´), 8.9 (3ah) ppm.

HRMS (ESIpos) m/z: Calcd for $C_{16}H_{16}F_3NO_3$ 327.1082, Found 327.1083.

IR (cm^{-1}): 3312, 2971, 1788, 1723, 1541, 1514, 1418, 1245, 1150, 1075, 950, 735.

(Z)-3-((4-ethyl-4-methyl-5-oxodihydrofuran-2(3H)-ylidene)methyl)benzaldehyde

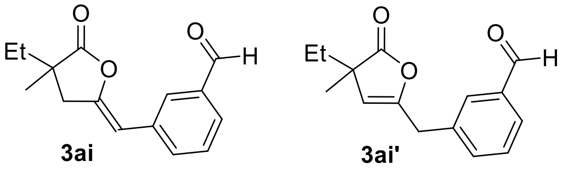

Following the general procedure **C** using **2m** (66 mg) and pentane/Et_2O (90:10 v/v) as an eluent, the target compound obtained as a colorless oil (25.9 mg, 0.106 mmol, 53%, **3ai : 3ai´** = 1.6:1).

^{1}H NMR (500 MHz, $CDCl_3$): δ = 10.02 (s, $1H^{3ai}$), 10.01 (s, $1H^{3ai´}$), 7.97 – 7.94 (m, $1H^{3ai}$ + $1H^{3ai´}$), 7.88 – 7.85 (m, $1H^{3ai}$ + $1H^{3ai´}$), 7.81 – 7.76 (m, $1H^{3ai}$ + $1H^{3ai´}$), 7.73 – 7.69 (m, $1H^{3ai}$ + $1H^{3ai´}$), 5.60 (t, *J* = 1.7 Hz, $1H^{3ai}$), 5.05 (t, *J* = 1.4 Hz, $1H^{3ai´}$), 3.71 (d, *J* = 1.3 Hz, $1H^{3ai´}$), 2.97 (dd, *J* = 16.4, 1.7 Hz, $1H^{3ai}$), 2.80 (dd, *J* = 16.4, 1.7 Hz, $1H^{3ai}$), 1.76 – 1.66 (m, $2H^{3ai}$), 1.60 – 1.53 (m, $2H^{3ai´}$), 1.34 (s, $3H^{3ai}$), 1.27 (s, $3H^{3ai´}$), 0.98 (t, *J* = 7.5 Hz, $3H^{3ai}$), 0.81 (t, *J* = 7.5 Hz, $3H^{3ai´}$) ppm.

^{13}C NMR (126 MHz, CDCl$_3$): δ = 192.7 (3ai), 192.2 (3ai´), 181.9 (3ai´), 179.5 (3ai), 152.7 (3ai), 148.1 (3ai´), 136.9 (3ai´), 136.8 (3ai´), 136.7 (3ai), 135.2 (3ai), 134.2 (3ai), 130.0 (3ai), 129.9 (3ai´), 129.5 (3ai´), 129.3 (3ai), 129.0 (3ai´), 127.5 (3ai), 110.5 (3ai´), 103.8 (3ai), 49.6 (3ai´), 43.3 (3ai), 39.6 (3ai), 34.6 (3ai´), 31.1 (3ai´), 30.9 (3ai), 23.1 (3ai´), 23.0 (3ai), 9.4 (3ai´), 8.9 (3ai) ppm.

HRMS (ESIpos) m/z: Calcd for $C_{15}H_{17}O_3$ 245.1172, Found 245.1170.

IR (cm^{-1}): 2969, 2931, 1792, 1693, 1603, 1457, 1385, 1192, 1073, 952, 792.

(*Z*)-3-ethyl-5-(3-methoxybenzylidene)-3-methyldihydrofuran-2(3H)-one

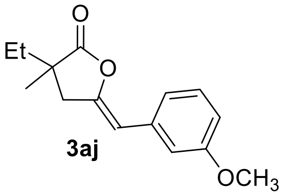

3aj

Following the general procedure **C** using **2n** (67.1 mg) and pentane/Et_2O (95:5 v/v) as an eluent, the target compound **3aj** was obtained as a colorless oil (21.3 mg, 86.5 µmol, 43%).

^{1}H NMR (500 MHz, CDCl$_3$): δ = 7.25 – 7.21 (m, 1H), 7.15 – 7.12 (m, 2H), 6.78 – 6.74 (m, 1H), 5.51 (t, *J* = 1.7 Hz, 1H), 3.82 (s, 3H), 2.92 (dd, *J* = 16.2, 1.7 Hz, 1H), 2.75 (dd, *J* = 16.2, 1.7 Hz, 1H), 1.74 – 1.65 (m, 2H), 1.32 (s, 3H), 0.97 (t, *J* = 7.5 Hz, 3H) ppm.

^{13}C NMR (126 MHz, CDCl$_3$): δ = 179.7, 159.8, 146.8, 135.5, 129.5, 121.1, 113.8, 112.6, 105.0, 55.4, 43.3, 39.6, 30.9, 23.0, 8.9 ppm.

HRMS (ESIpos) m/z: Calcd for $C_{15}H_{19}O_3$ 247.1328, Found 247.1325.

IR (cm^{-1}): 2967, 1793, 1683, 1598, 1455, 1256, 1155, 1071, 949, 866.

(*Z*)-3-ethyl-3-methyl-5-(2-methylbenzylidene)dihydrofuran-2(3H)-one

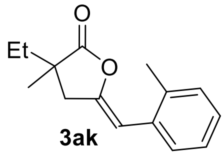

3ak Following the general procedure **C** using **2o** (59.1 mg) and pentane/Et_2O (98:2 v/v) as an eluent, the target compound **3ak** was obtained as a colorless oil (23.8 mg, 0.103 mmol, 52%).

^{1}H NMR (500 MHz, $CDCl_3$): δ = 7.79 – 7.75 (m, 1H), 7.21 – 7.10 (m, 3H), 5.68 (t, *J* = 1.8 Hz, 1H), 2.96 (dd, *J* = 16.1, 1.7 Hz, 1H), 2.79 (dd, *J* = 16.1, 1.8 Hz, 1H), 2.32 (s, 3H), 1.75 – 1.67 (m, 2H), 1.33 (s, 3H), 0.99 (t, *J* = 7.5 Hz, 3H) ppm.

^{13}C NMR (126 MHz, $CDCl_3$): δ = 179.9, 146.6, 135.2, 132.6, 130.1, 129.2, 126.9, 126.2, 102.3, 43.5, 39.6, 30.9, 22.9, 20.4, 8.9 ppm.

HRMS (ESIpos) m/z: Calcd for $C_{15}H_{19}O_2$ 231.1379, Found 231.1377.

IR (cm^{-1}): 2963, 1797, 1688, 1449, 1275, 1055, 949, 751.

6. Preparative Scale Applications and Derivatizations

Large-scale reactions

(*Z*)-5-Benzylidene-3-cyclohexyl-3-methyldihydrofuran-2(3H)-one

Reaction on 1.0 mmol Scale

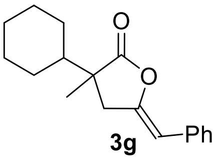

3g

An oven dried 150 mL Schlenk tube was charged with $Pd(OAc)_2$ (22.5 mg, 0.100 mmol, 10 mol%), **L10** (38.2 mg, 0.100 mmol, 10 mol%), K_2CO_3 (69 mg, 0.50 mmol, 0.5 equiv.), AgOAc (334 mg, 2.00 mmol, 2 equiv.), MnO_2 (17.4 mg, 0.2 mmol, 20 mol%), styrene **2a** (285 µL, 2.50 mmol, 2.5 equiv.), carboxylic acid **1g** (170 mg, 1.00 mmol, 1.0 equiv.), and HFIP (10.0 mL). The reaction mixture was stirred at 70 °C (recorded by a Thermometer) for 21 h in a preheated metal block. After the mixture was allowed to cool to room temperature, formic acid (0.5 mL) was added to the reaction mixture and it was filtered over a pad of Celite® using CH_2Cl_2 (40 mL) to complete the elution. All volatiles were removed under reduced pressure, and the residue was purified by silica gel column chromatography to afford the target compound **3g** as a colorless oil (158 mg, 0.584 mmol, 58%).

(*Z*)-5-Benzylidene-3-cyclohexyl-3-methyldihydrofuran-2(3H)-one

Reaction on 5.0 mmol scale

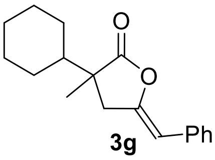

3g

An oven dried 150 mL Schlenk tube was charged with $Pd(OAc)_2$ (112 mg, 0.500 mmol, 10 mol%), **L10** (191 mg, 0.500 mmol, 10 mol%), K_2CO_3 (345 mg, 2.50 mmol, 0.5 equiv.), AgOAc (1.7 g, 10 mmol, 2 equiv.), MnO_2 (87 mg, 1.0 mmol, 20 mol%), styrene **2a** (1.40 mL, 12.5 mmol, 2.5 equiv.), carboxylic acid **1g** (851 mg, 5.00 mmol, 1.0 equiv.), and HFIP (50.0 mL). The reaction mixture was stirred at 70 °C (recorded by a Thermometer) for 21 h in a preheated metal block. After the mixture was allowed to cool to room temperature, formic acid (2.0 mL) was added to the reaction mixture and it was filtered over a pad of Celite® using CH_2Cl_2 (60 mL) to complete the elution. All volatiles were removed under reduced pressure, and the residue was purified by silica gel column chromatography to afford the target compound **3g** as a colorless oil (610 mg, 2.26 mmol, 45%).

(*Z*)-5-benzylidene-3-(3-(2,5-dimethylphenoxy)propyl)-3-methyldihydrofuran-2(3H)-one

Reaction on 1.0 mmol

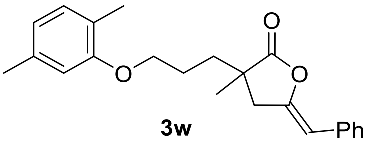

3w

An oven dried 150 mL Schlenk tube was charged with $Pd(OAc)_2$ (22.5 mg, 0.100 mmol, 10 mol%), **L10** (38.2 mg, 0.100 mmol, 10 mol%), K_2CO_3 (69 mg, 0.50 mmol, 0.5 equiv.), AgOAc (334 mg, 2.00 mmol, 2 equiv.), MnO_2 (17.4 mg, 0.2 mmol, 20 mol%), styrene **2a** (285 µL, 2.50 mmol, 2.5 equiv.), carboxylic acid **1w** (250 mg, 1.00 mmol, 1.0 equiv.), and HFIP (10.0 mL). The reaction mixture was stirred at 70 °C (recorded by a Thermometer) for 21 h in a preheated metal block. After the mixture was allowed to cool to room temperature, formic acid (0.5 mL) was added to the reaction mixture and it was filtered over a pad of Celite® using CH_2Cl_2 (40 mL) to complete the elution. All volatiles were removed under reduced pressure, and the residue was purified by silica gel column chromatography to afford the target compound **3w** as a colorless oil (168 mg, 0.480 mmol, 48%).

Diversification of the products

2-(3-(2,5-dimethylphenoxy)propyl)-2-methyl-4-oxo-5-phenylpentanal

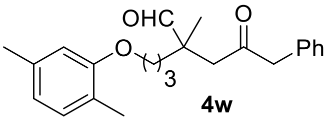

4w

Following a modified literature procedure,[2] (*Z*)-5-benzylidene-3-(3-(2,5-dimethylphenoxy)propyl)-3-methyldihydrofuran-2(3H)-one **3w** (35 mg, 0.10 mmol, 1 equiv.) was dissolved in dry THF (2.0 mL) and the mixture was cooled to – 50 °C. DIBAL-H (0.15 mL, 0.15 mmol, 1.5 equiv., 1M in toluene) was added slowly to the mixture under N_2 atmosphere. The reaction mixture was stirred at – 50 °C for 2 h. After the completion of the reaction (monitored by TLC), the cooling bath was removed and the reaction was quenched with water (5 mL). The reaction mixture was concentrated under reduced pressure. Water (15 mL) and NH_4Cl (5 mL) were added and the aqueous phase was extracted with CH_2Cl_2 (2× 15 mL). The combined organic phases were dried over $MgSO_4$, filtered, and the solvent was removed under reduced pressure. The crude product was purified by silica gel column chromatography using pentane: Et_2O (85:15) to obtain the target compound **4w** as a colorless oil (24.8 mg, 70.4 µmol, 70%).

^{1}H NMR (500 MHz, $CDCl_3$): δ = 9.58 (s, 1H), 7.35 – 7.30 (m, 2H), 7.21 – 7.17 (m, 1H), 7.20 – 7.17 (m, 2H), 7.00 (d, *J* = 7.5 Hz, 1H), 6.66 (d, *J* = 7.5 Hz, 1H), 6.57 (s, 1H), 3.86 – 3.82 (m, 2H), 3.69 (s, 2H), 2.84 (d, *J* = 17.8 Hz, 1H), 2.71 (d, *J* = 17.8 Hz, 1H), 2.30 (s, 3H), 2.15 (s, 3H), 1.72 – 1.58 (m, 4H), 1.13 (s, 3H) ppm.

^{13}C NMR (126 MHz, $CDCl_3$): δ = 206.1, 205.0, 156.9, 136.7, 133.8, 130.5, 129.5, 129.0, 127.4, 123.7, 121.0, 112.0, 67.7, 50.8, 48.0, 47.1, 32.3, 24.1, 21.5, 19.4, 15.9 ppm.

HRMS (ESIpos) m/z: Calcd for $C_{23}H_{29}O_3$ 353.2111, Found 353.2109.

IR (cm^{-1}): 2922, 1798, 1691, 1508, 1261, 1129, 803, 750.

Methyl 2-(3-(2,5-dimethylphenoxy)propyl)-2-methyl-4-oxo-5-phenylpentanoate

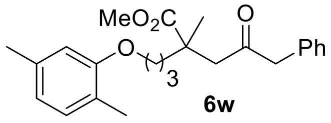

Following a literature procedure,[3] K_2CO_3 was added (98 mg, 0.713 mmol, 5 equiv.) to **3w (** 50.0 mg, 0.143 mmol, 1.0 equiv.) in dry MeOH (2 mL). The solution was stirred at rt for 6 h. The reaction was quenched with H_2O (10 mL). The reaction mixture was concentrated under reduced pressure. Water (15 mL) was added and the aqueous phase was extracted with CH_2Cl_2 (2× 10 mL). The combined organic phases were dried over $MgSO_4$, filtered, and the solvent was removed under reduced pressure. The crude product was purified by silica gel column chromatography using pentane: Et_2O (85:15) to obtain the target compound **6w** as a colorless oil (43.1 mg, 0.113 mmol, 79%).

^{1}H NMR (600 MHz, $CDCl_3$): δ = 7.35 – 7.31 (m, 2H), 7.31 – 7.26 (m, 1H), 7.21 – 7.18 (m, 2H), 7.00 (d, *J* = 7.5 Hz, 1H), 6.66 (d, *J* = 7.5 Hz, 1H), 6.57 (s, 1H), 3.88 – 3.83 (m, 2H), 3.67 (s, 2H), 3.65 (s, 3H), 2.96 (d, *J* = 17.8 Hz, 1H), 2.63 (d, *J* = 17.8 Hz, 1H), 2.30 (s, 3H), 2.15 (s, 3H), 1.77 – 1.62 (m, 4H), 1.22 (s, 3H) ppm.

^{13}C NMR (151 MHz, $CDCl_3$): δ = 206.0, 177.0, 156.9, 136.6, 134.1, 130.4, 129.5, 128.9, 127.2, 123.7, 120.9, 112.0, 67.7, 52.0, 50.7, 50.1, 43.5, 36.0, 24.5, 21.8, 21.5, 15.9 ppm.

HRMS (ESIpos) m/z: Calcd for $C_{24}H_{31}O_4$ 383.2216, Found 383.2213.

IR (cm^{-1}): 2921, 1720, 1508, 1261, 1129, 1033, 803, 749.

5-benzyl-3-(3-(2,5-dimethylphenoxy)propyl)-3-methyldihydrofuran-2(3H)-one

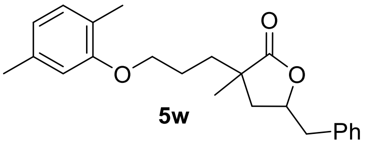

5w

Following the literature procedure,[4] a 10 mL Schlenk tube was charged with (*Z*)-5-benzylidene-3-cyclohexyl-3-methyldihydrofuran-2(3H)-one **3w** (35.0 mg, 0.100 mmol, 1 equiv.) and methanol (0.3 mL). The reaction mixture was cooled to 0 °C and powdered $NaBH_4$ (7.5 mg, 20 µmol, 2 equiv.) was added in one portion with constant stirring. After the full consumption of the starting material (monitored by TLC), The reaction was quenched by the addition of excess methanol (5 mL). The reaction mixture was concentrated under reducted pressure. Water (15 mL) and NH_4Cl (5 mL) were added and the aqueous phase was extracted with CH_2Cl_2 (2× 15 mL). The combined organic phases were dried over $MgSO_4$, filtered, and the solvent was removed under reduced pressure. The crude product was purified by silica gel column chromatography using pentane: Et_2O (80:20) to obtain the major distereomer of the target compound **5w** as a colorless oil (19.5 mg, 55.3 µmol, 55%). The minor diastereomer of **5w** was obtained as a colorless oil (3 mg, 8.8 µmol, 8%)

The analytical data of major diastereomer are given below:

^{1}H NMR (500 MHz, $CDCl_3$): δ = 7.34 – 7.29 (m, 2H), 7.28 – 7.22 (m, 3H), 7.01 (d, *J* = 7.5 Hz, 1H), 6.67 (d, *J* = 7.5 Hz, 1H), 6.60 (s, 1H), 4.71 – 4.64 (m, 1H), 3.95 – 3.87 (m, 2H), 3.11 (dd, *J* = 14.0, 6.2 Hz, 1H), 2.93 (dd, *J* = 14.0, 6.2 Hz, 1H), 2.32 (s, 3H), 2.17 (s, 3H), 2.03 – 1.91 (m, 2H), 1.84 – 1.70 (m, 3H), 1.67 – 1.58 (m, 1H), 1.26 (s, 3H) ppm.

^{13}C NMR (126 MHz, $CDCl_3$): δ = 181.2, 156.9, 136.7, 136.1, 130.5, 129.6, 128.8, 127.1, 123.7, 121.0, 112.1, 77.4, 67.7, 44.0, 41.6, 39.9, 34.2, 24.7, 22.9, 21.5, 15.9 ppm.

HRMS (EI) m/z: Calcd for $C_{23}H_{28}O_3$ 352.2038, Found 352.2038.

IR (cm^{-1}): 2923, 1764, 1508, 1454, 1260, 1129, 1018, 804, 750.

2-cyclohexyl-2-methyl-5-phenylpentanoic acid

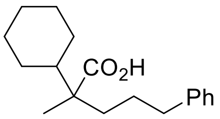

7g

Following a modified literature procedure,[5] (*Z*)-5-benzylidene-3-cyclohexyl-3-methyldihydrofuran-2(3H)-one **3g** (27 mg, 0.10 mmol, 1 equiv.) was dissolved in dry THF (2.0 mL). Pd/C (10 mg, 0.01 mmol, 10 mol%) was added to the mixture. Next, an atmosphere of hydrogen was introduced by briefly evacuating the flask, then flushing with pure hydrogen (1 atm, hydrogen balloon). The mixture was stirred at room temperature for 4 h under an H_2 atmosphere. The hydrogen was removed under vacuum, and the flask was refilled with nitrogen. The mixture was filtered through a pad of celite using CH_2Cl_2 (25 mL) to complete the elution and concentrated under reduced pressure. The crude product was purified by silica gel column chromatography using pentane: Et_2O (60:40) to obtain target compound **7g** as a colorless oil (26.7 mg, 97.3 µmol, 97%).

^{1}H NMR (500 MHz, $CDCl_3$): δ = 7.29 – 7.24 (m, 2H), 7.19 – 7.15 (m, 3H), 2.65 – 2.54 (m, 2H), 1.80 – 1.58 (m, 7H), 1.55 – 1.51 (m, 3H), 1.25 – 1.19 (m, 2H), 1.16 – 1.06 (m, 2H), 1.03 (s, 3H), 1.00 – 0.91 (m, 1H) ppm.

^{13}C NMR (126 MHz, $CDCl_3$): δ = 183.1, 142.2, 128.3, 128.3, 125.7, 49.4, 45.3, 37.0, 36.4, 28.5, 27.0, 26.8, 26.7, 26.6, 26.5, 16.5 ppm.

HRMS (ESIneg) m/z: Calcd for $C_{18}H_{25}O_2$ 273.1860, Found 273.1862.

IR (cm^{-1}): 2929, 1691, 1450, 1270, 1203, 939, 746.

7. Preliminary Mechanistic Investigations

Synthesis of the Proposed Reaction Intermediates

(E)-2-ethyl-2-methyl-5-phenylpent-4-enoic acid

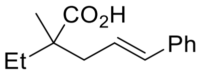

Int-1 Following the literature procedure,[1a] using methyl 2-methylbutanoate (1.0 g, 8.6 mmol, 1.0 equiv.), *i*Pr_2NH (1.7 mL, 9.5 mmol, 1.1 equiv.), *n*-BuLi (6.0 mL, 9.5 mmol, 1.1 equiv.) and (E)-(3-bromoprop-1-en-1-yl)benzene (2.20 g, 11.2 mmol, 1.3 equiv.), the target compound **Int-1** was obtained as a colorless solid (465 mg, 2.10 mmol, 25%).

^{1}H NMR (600 MHz, $CDCl_3$): δ = 7.36 – 7.33 (m, 2H), 7.31 – 7.27 (m, 2H), 7.22 – 7.18 (m, 1H), 6.44 (d, *J* = 15.7 Hz, 1H), 6.17 (ddd, *J* = 15.7, 7.8, 7.2 Hz, 1H), 2.56 (ddd, *J* = 13.8, 7.2, 1.4 Hz, 1H), 2.38 (ddd, *J* = 13.8, 7.8, 1.3 Hz, 1H), 1.78 – 1.70 (m, 1H), 1.62 – 1.52 (m, 1H), 1.19 (s, 3H), 0.92 (t, *J* = 7.5 Hz, 3H) ppm.

^{13}C NMR (151 MHz, $CDCl_3$): δ = 183.4, 137.6, 133.4, 128.6, 127.3, 126.3, 125.8, 46.8, 41.8, 31.6, 21.1, 9.1 ppm.

HRMS (ESIneg) m/z: Calcd for $C_{14}H_{17}O_2$ 217.1223, Found 217.1228.

(Z)-2-ethyl-2-methyl-5-phenylpent-4-enoic acid

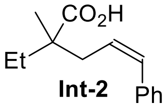

Int-2 Following the literature procedure,[6] using 2-ethyl-2-methyl-5-phenylpent-4-ynoate (0.50 g, 2.1 mmol, 1 equiv.), $Ni(OAc)_2{\cdot}4H_2O$ (0.5 g, 2.1 mmol), $NaBH_4$ (77 mg, 2.1 mmol, 1 equiv.), and ethylenediamine (370 mg, 6.14 mmol, 3 equiv.) the target compound **Int-2** was obtained as a colorless oil (300 mg, 1.39 mmol, 68%).

^{1}H NMR (600 MHz, $CDCl_3$): δ = 7.35 – 7.31 (m, 2H), 7.27 – 7.25 (m, 2H), 7.25 – 7.21 (m, 1H), 6.55 (dd, *J* = 11.8, 2.0 Hz, 1H), 5.65 (ddd, *J* = 11.8, 7.8, 6.6 Hz, 1H), 2.67 (ddd, *J* = 15.2, 6.6, 2.1 Hz, 1H), 2.56 (ddd, *J* = 15.2, 7.8, 1.8 Hz, 1H), 1.75 –1.67 (m, 1H), 1.57 –1.48 (m, 1H), 1.15 (s, 3H), 0.83 (t, *J* = 7.5 Hz, 3H) ppm.

^{13}C NMR (151 MHz, $CDCl_3$): δ = 182.7, 137.5, 131.5, 128.9, 128.3, 127.7, 126.8, 46.5, 36.7, 31.5, 21.0, 9.0 ppm.

HRMS (ESIneg) m/z: Calcd for $C_{14}H_{17}O_2$ 217.1223, Found 217.1228.

Investigation of the cyclization step leading to the product formation

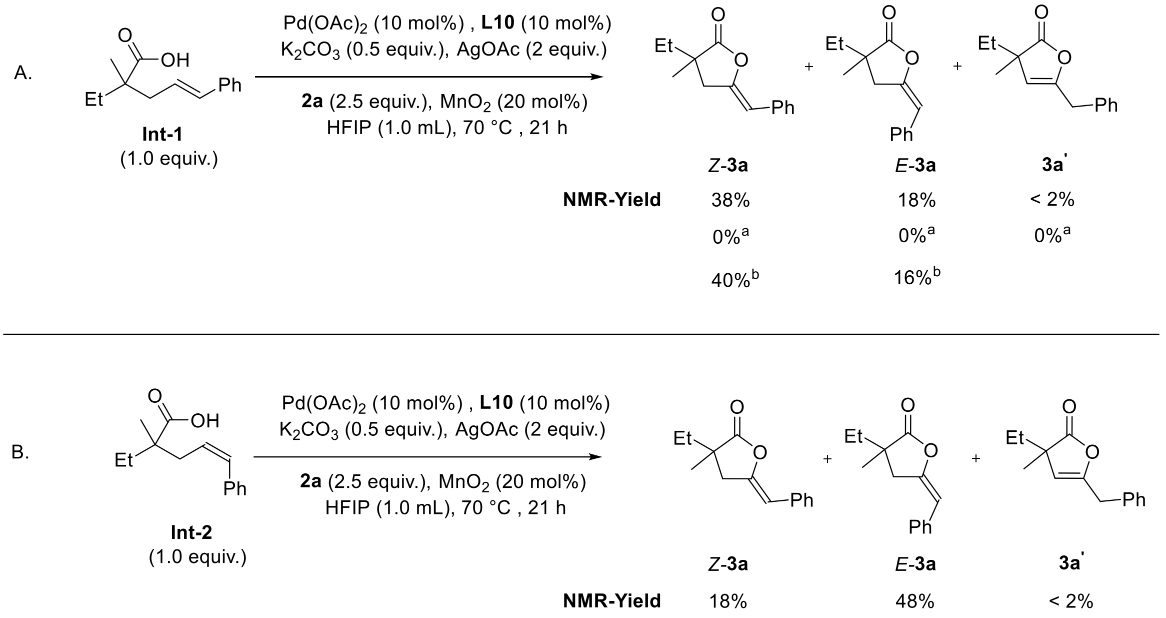

Scheme S19: Control experiments with **Int-1** and **Int-2**. [a] without $Pd(OAc)_2$. [b] without ligand

In order to gain detailed insights on the final cyclization step leading to product formation, several control experiments were conducted (Scheme S19). When **Int-1** was subjected to the standard olefination conditions, *Z*-**3a** (38%, Scheme S19A) was obtained as the major product, along with *E*-**3a** (18%) and **3a´** (< 2%). As expected, no product formed in the absence of Pd, indicating its essential role in the cyclization step. The omission of ligand (**L10**) led to a virtually identical reaction outcome as judged by the ^{1}H NMR-yield, suggesting the ligand has minimal to no influence on the cyclization step. Furthermore, treating **Int-2** under the same conditions yielded *E*-**3a** (48%, Scheme S19B) as major product, alongside *Z*-**3a** (18%) and **3a´**(< 2%). These results confirm that **Int-1** is a viable reaction intermediate in our reaction.

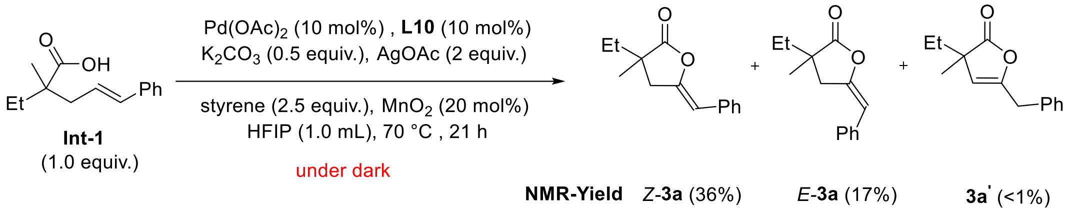

Scheme S20: Control experiments with **Int-1** under dark

To determine whether *E* and *Z* isomers originate from a photochemical *E*/*Z* isomerization of the product, the **Int-1** was subjected to the standard olefination conditions in the dark. However, the product (*Z* and *E*) distribution remained unaltered in **3a** (Scheme S20), indicating *E*-**3a** does not result from a photochemical isomerization of the initially formed *Z*-**3a**. Another possibility considered was that the intermediate (**Int-1** or **Int-2**) may undergo *E*/*Z* isomerization to generate an equilibrium mixture of **Int-1** and **Int-2**, which would then cyclize to form *Z*-**3a** and *E*-**3a** respectively. However, crude ^{1}H NMR analysis of the control experiments shown in Scheme S19 provided no evidence of such isomerization occurring within the remaining **Int-1** or **Int-2**. Therefore, *E*/*Z* isomerization in **3a** likely proceeds through another mechanism.[7]

Although the control experiments with the intermediates (**Int-1** and **Int-2**) led to the mixture of *Z*- and *E*-**3a**, it is noteworthy that the *E*-isomer of **3** was not observed for any substrate during the scope studies presumably due to the substantial change in the effective concentration of reagent and/or pH required for *E*/*Z* isomerization in the one-pot protocol comprising two catalytic cycles, compared to the the control experiments with Intermediates comprising only the final cyclization step.

Comment on the formation of 3´

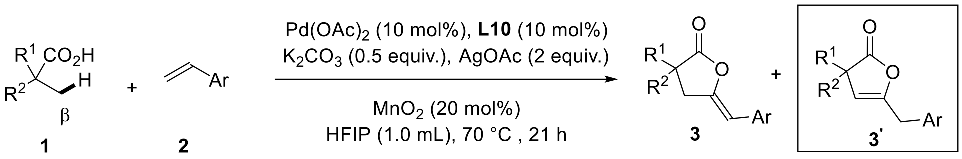

During the scope studies, it was observed that some substrates yielded a mixture of **3** and **3´**, likely due to the isomerization tendency of exocyclic double bond, as previously described in the literature.[8] While no general trend was found with different carboxylic acid substrates, styrenes with strongly electron-withdrawing groups (such as -CF_3, -NO_2, -CHO) typically exhibited a higher **3´** to **3** ratio. To determine whether **3´** originates from **3**, we conducted a series of control experiments. First, lactone **3z** was subjected to the standard conditions with pivalic acid (**1a**) as substrate. While **1a** underwent smooth product formation to afford **3a** (50%), no formation of **3z´** was detected. Instead, more than 80% of **3z** were recovered, alongside partial decomposition to **4z** (12%, Scheme S21). This substrate yielded substantial **3z´** in the scope studies and in this case is was used as starting material in pure form. Notably, if the reaction conditions would generate a thermodynamic equilibrium, the same ratio as in the scope entry should be observed in this experiment.

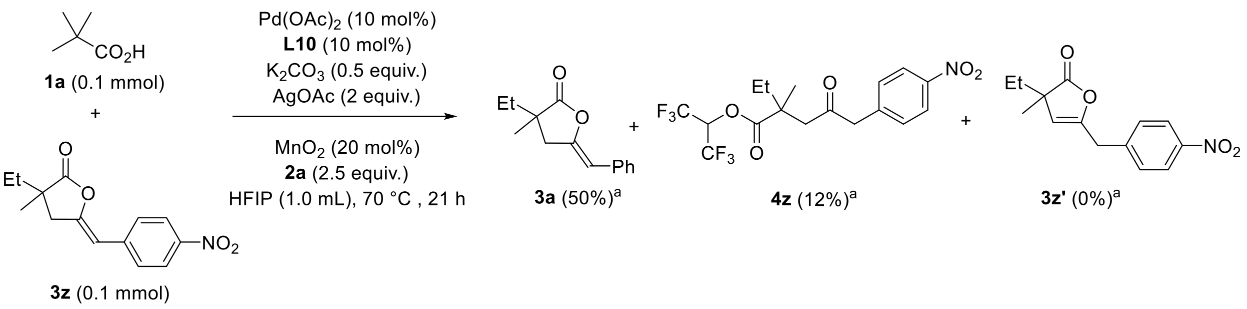

Scheme S21: Evaluation of the isomerization of **3z** under the standard reaction conditions. [a]Yields and conversions were calculated using ^{1}H NMR analysis of the crude reaction mixture using CH_2Br_2 as an internal standard.

Moreover, time-course analysis of the reaction bewteen **1b** and **2z** revealed that the ratio of **3z** to **3z´** remained nearly unaltered over time (Scheme S22), despite substantial accumulation of **3z**. The same trend was noted in the experiments shown in Scheme S14. Therefore, considering the results described in these experiments, it can be concluded that **3z´** (or **3´**) does not arise from the initially formed **3z** (or **3**).

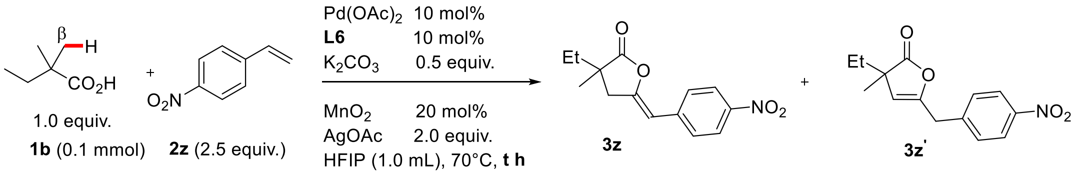

Entry	t (h)	3z	3z'	Ratio (3z/3z')
1.	6	38%	12%	3.16
2.	12	42%	13%	3.23
3.	18	51%	16%	3.18
4.	21	52%	16%	3.25
5.	30	55%	17%	3.23
6.	36	53%	16%	3.31

Scheme S22: Timely monitoring the formation of 3z and 3z'

Building upon the conclusions of the control experiments, we propose the following mechanism: **Int-1** first isomerizes to form **Int-3**,[9] a β,γ-unsaturated acid. Due to the well-known propensity of such species to undergo Pd-catalyzed 5-*endo*-*trig*-type cyclization,[10] **Int-3** undergoes a sequence of oxypalladation and β-H elimination to afford **3´**. Notably, the distribution between these intermediates will be strongly influenced by the electronic property of aryl (-Ar) group, as reflected in the scope studies.

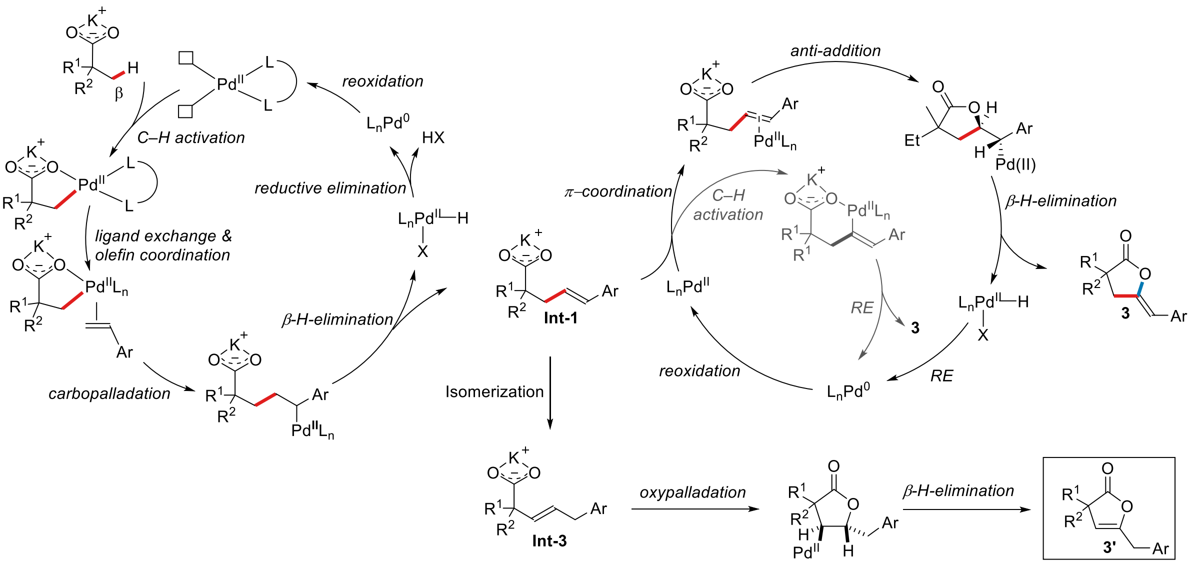

Scheme S23: Plausible mechanism for the formation of 3´

Investigations on the role of MnO_2

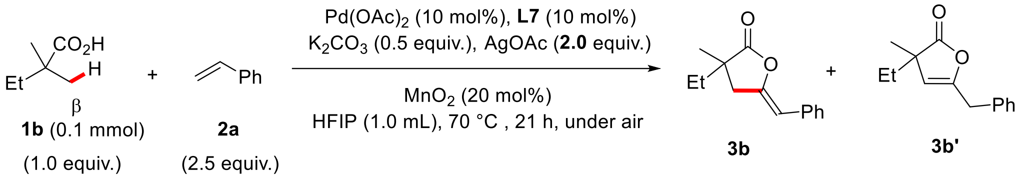

Entry	Conditions	3b	3b'	Overall yield
1.	As above	66%	7%	73%
2.	no AgOAc	12%	6%	18%
3.	no AgOAc and under N_2	9%	5%	14%
4.	1.0 eq. MnO_2 instead of AgOAc	21%	9%	30%
5.	2.0 eq. MnO_2 instead of AgOAc	22%	9%	31%
6.	1.0 eq. MnO_2 instead of AgOAc and under N_2	8%	4%	12%
7.	1.0 eq. MnO_2 instead of AgOAc and under O_2	23%	9%	32%

Scheme S24: Control experiments under the fully optimized conditions

To investigate the role of MnO_2, we conducted a series of control experiments. In the absence of AgOAc, the reaction afforded **3b** and **3b'** with a combined yield of 18% (Entry 2, Scheme S24). Additionally, performing the reaction under N_2 atmosphere resulted in a slight decrease in the overall yield to 14% (Entry 3), indicating both air and MnO_2 contribute to the oxidation process, with MnO_2 having a more significant effect. Since the final product forms through two catalytic cycles — both requiring the re-oxidation of Pd^0 to Pd^{II} — a total of 4-electrons need to be removed from Pd by oxidation. Therefore, either 4 equiv. of Ag(I) or a combination of silver salt, MnO_2, and air are required, with the latter showing superior efficiency in our system. Furthermore, replacing AgOAc with stoichiometric MnO_2 afforded 30-31% overall yield (Entry 4-5) whereas performing the reaction under N_2 led to drastic decrease in the yield (Entry 6). However, conducting the reaction under O_2 atmosphere did not improve the yield (Entry 7). Overall, these results indicate that all three oxidizing components are involved in at least one of the oxidation steps.

8. References

[1] a) F. Ghiringhelli, A. Uttry, K. K. Ghosh, M. van Gemmeren, *Angew. Chem. Int. Ed.* **2020**, *59*, 23127-23131; b) Y. Zhang, J. Struwe, L. Ackermann, *Angew. Chem. Int. Ed.* **2020**, *59*, 15076-15080; c) L. Hu, G. Meng, X. Chen, J. S. Yoon, J.-R. Shan, N. Chekshin, D. A. Strassfeld, T. Sheng, Z. Zhuang, R. Jazzar, G. Bertrand, K. N. Houk, J.-Q. Yu, *J. Am. Chem. Soc.* **2023**, *145*, 16297-16304.

[2] M. Yoshida, H. Otaka, T. Doi, *Eur. J. Org. Chem.* **2014**, *2014*, 6010-6016.

[3] F. Zhang, S. Dutta, A. Petti, D. Rana, C. G. Daniliuc, F. Glorius, *Angew. Chem. Int. Ed.* **2025**, *64*, e202418239.

[4] P. C. P, E. Joseph, A. A, N. D. S, I. Ibnusaud, J. Raskatov, B. Singaram, *J. Org. Chem.* **2018**, *83*, 1431-1440.

[5] J. Mitra, X. Zhou, T. Rauchfuss, *Green Chem.* **2015**, *17*, 307-313.

[6] K. Shen, Q. Wang, *J. Am. Chem. Soc.* **2017**, *139*, 13110-13116.

[7] R. Matsuura, M. K. Karunananda, M. Liu, N. Nguyen, D. G. Blackmond, K. M. Engle, *ACS Catal.* **2021**, *11*, 4239-4246.

[8] a) H. Rudler, P. Harris, A. Parlier, F. Cantagrel, B. Denise, M. Bellassoued, J. Vaissermann, *Journal of Organometallic Chemistry* **2001**, *624*, 186-202; b) C. Pimpasri, X. Luo, S. Yang, S. Díez-González, *Adv. Synth. Catal.* **2024**, *366*, 806-812; c) R. M. Trend, Y. K. Ramtohul, B. M. Stoltz, *J. Am. Chem. Soc.* **2005**, *127*, 17778-17788.

[9] A. Sen, T. W. Lai, *Inorganic Chemistry* **1984**, *23*, 3257-3258.

[10] a) G. B. Bajracharya, P. S. Koranne, R. N. Nadaf, R. K. M. Gabr, K. Takenaka, S. Takizawa, H. Sasai, *Chem. Commun.* **2010**, *46*, 9064-9066; b) K. Kiyokawa, K. Takemoto, S. Yahata, T. Kojima, S. Minakata, *Synthesis* **2017**, *49*, 2907-2912.

9. NMR Data

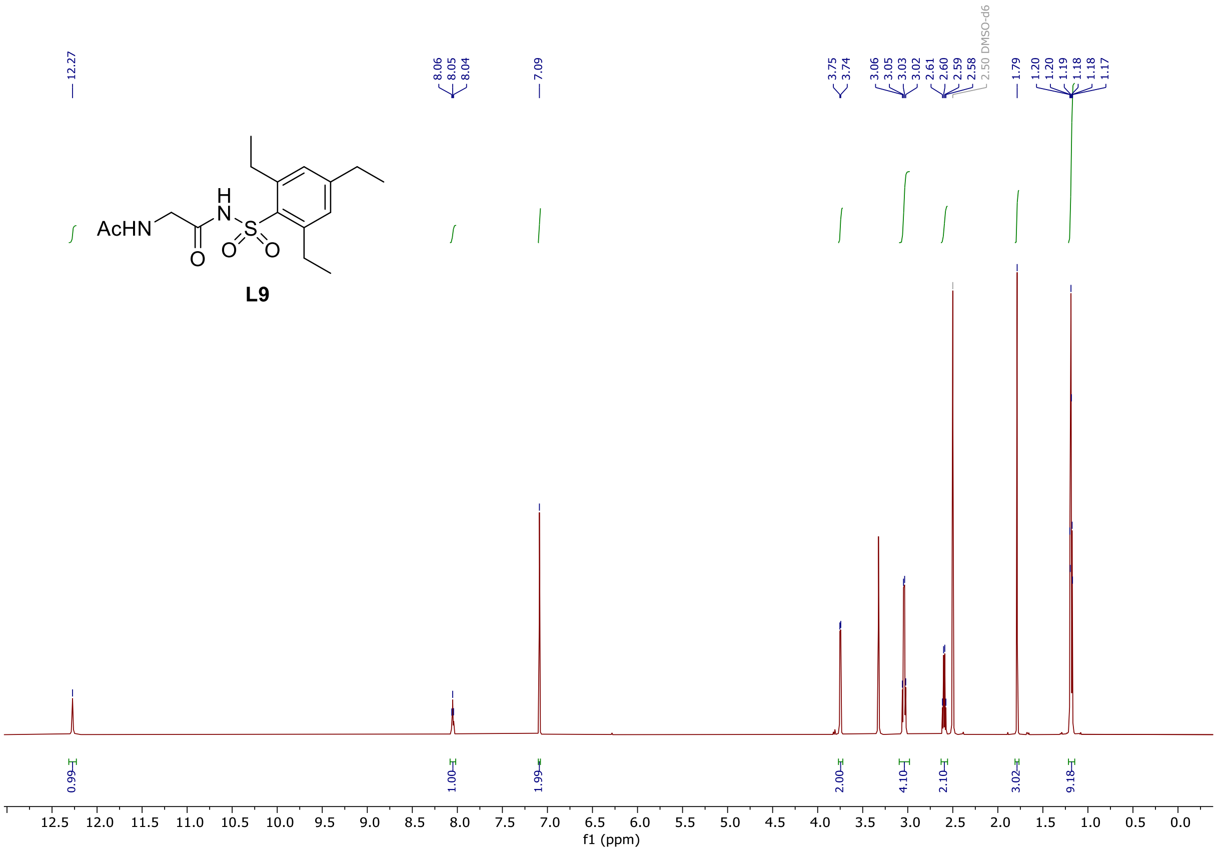

12.27
8.06
8.05
8.04
7.09
3.75
3.74
3.06
3.05
3.03
3.02
2.61
2.60
2.59
2.58
2.50 DMSO-d6
1.79
1.20
1.20
1.19
1.18
1.18
1.17
AcHN
H
N
S
O
O
O
L9
0.99
1.00
1.99
2.00
4.10
2.10
3.02
9.18
12.5 12.0 11.5 11.0 10.5 10.0 9.5 9.0 8.5 8.0 7.5 7.0 6.5 6.0 5.5 5.0 4.5 4.0 3.5 3.0 2.5 2.0 1.5 1.0 0.5 0.0
f1 (ppm)

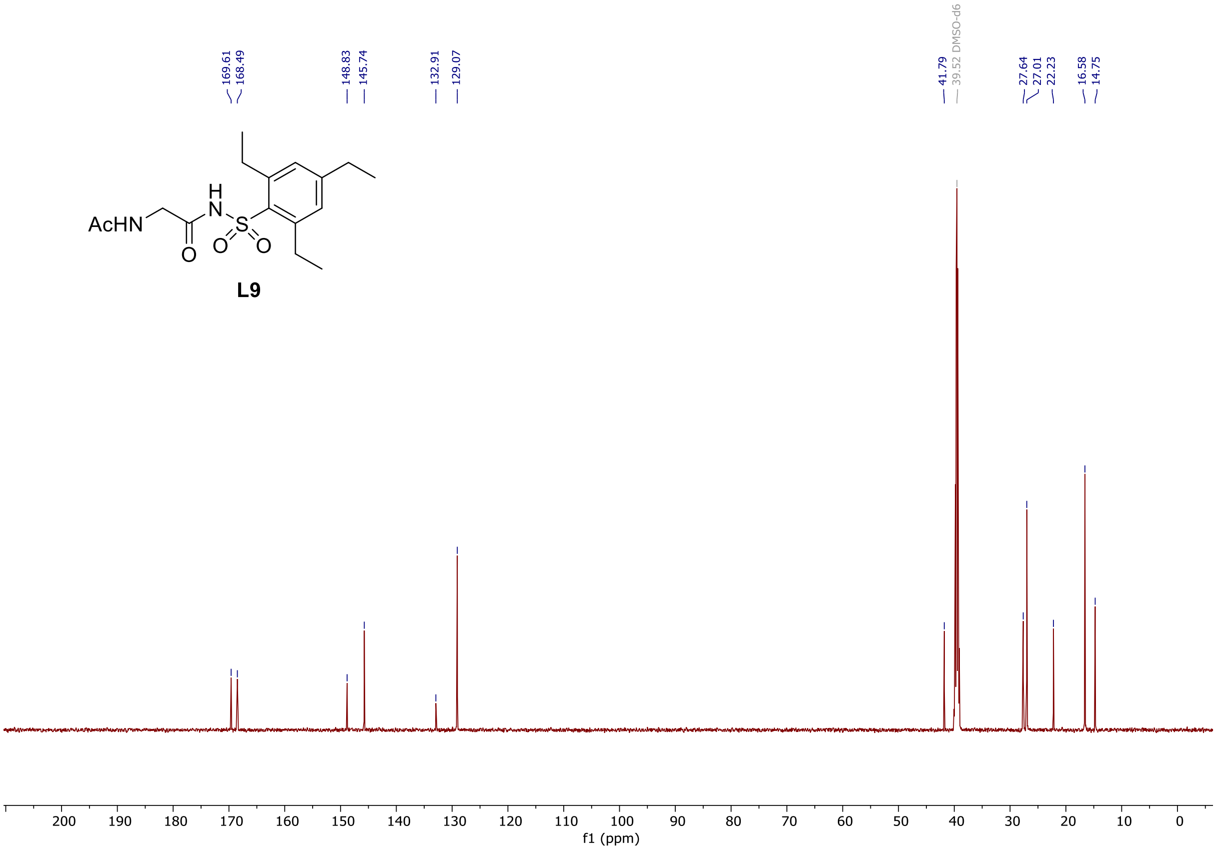

169.61
168.49
148.83
145.74
132.91
129.07
41.79
39.52 DMSO-d6
27.64
27.01
22.23
16.58
14.75
AcHN
H
N
S
O
O
O
L9
200 190 180 170 160 150 140 130 120 110 100 90 80 70 60 50 40 30 20 10 0
f1 (ppm)

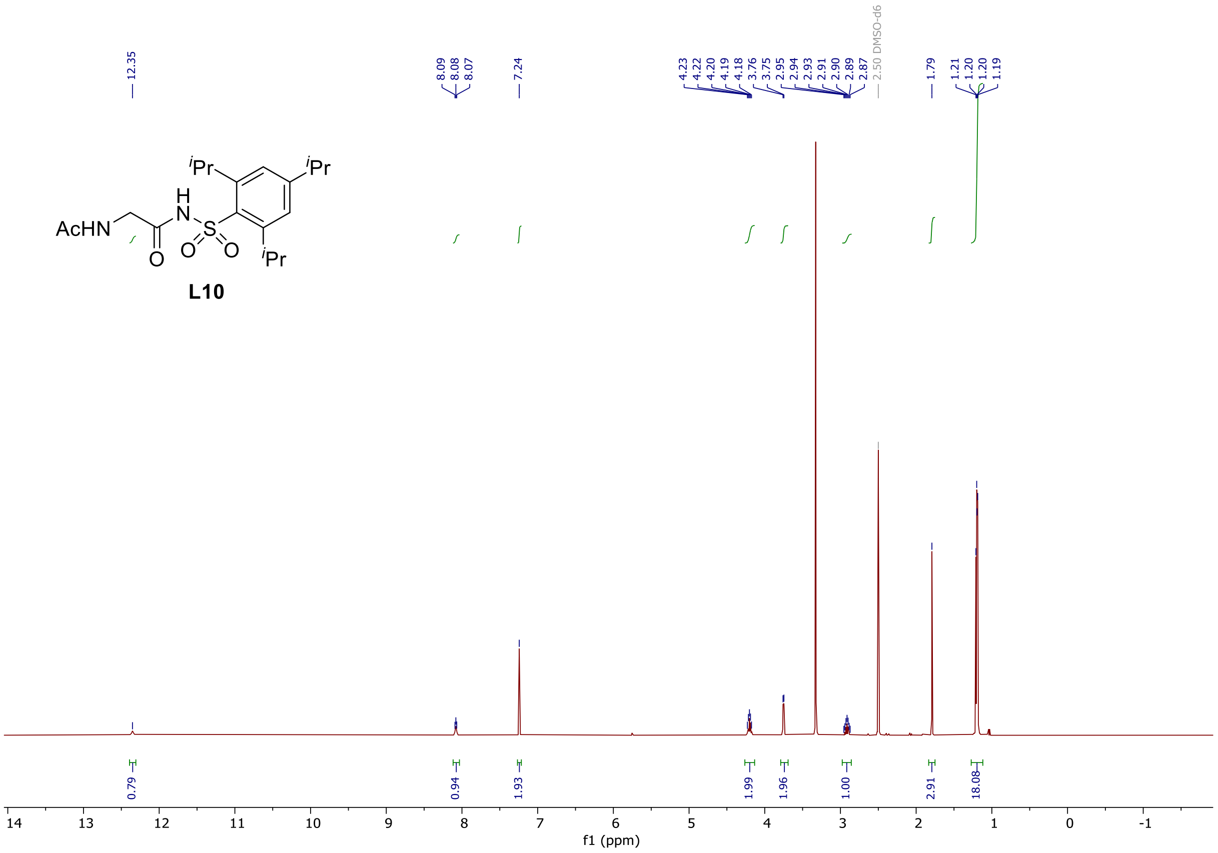
12.35
8.09
8.08
8.07
7.24
4.23
4.22
4.20
4.19
4.18
3.76
3.75
2.95
2.94
2.93
2.91
2.90
2.89
2.87
2.50 DMSO-d6
1.79
1.21
1.20
1.20
1.19
AcHN
L10
0.79
0.94
1.93
1.99
1.96
1.00
2.91
18.08
f1 (ppm)

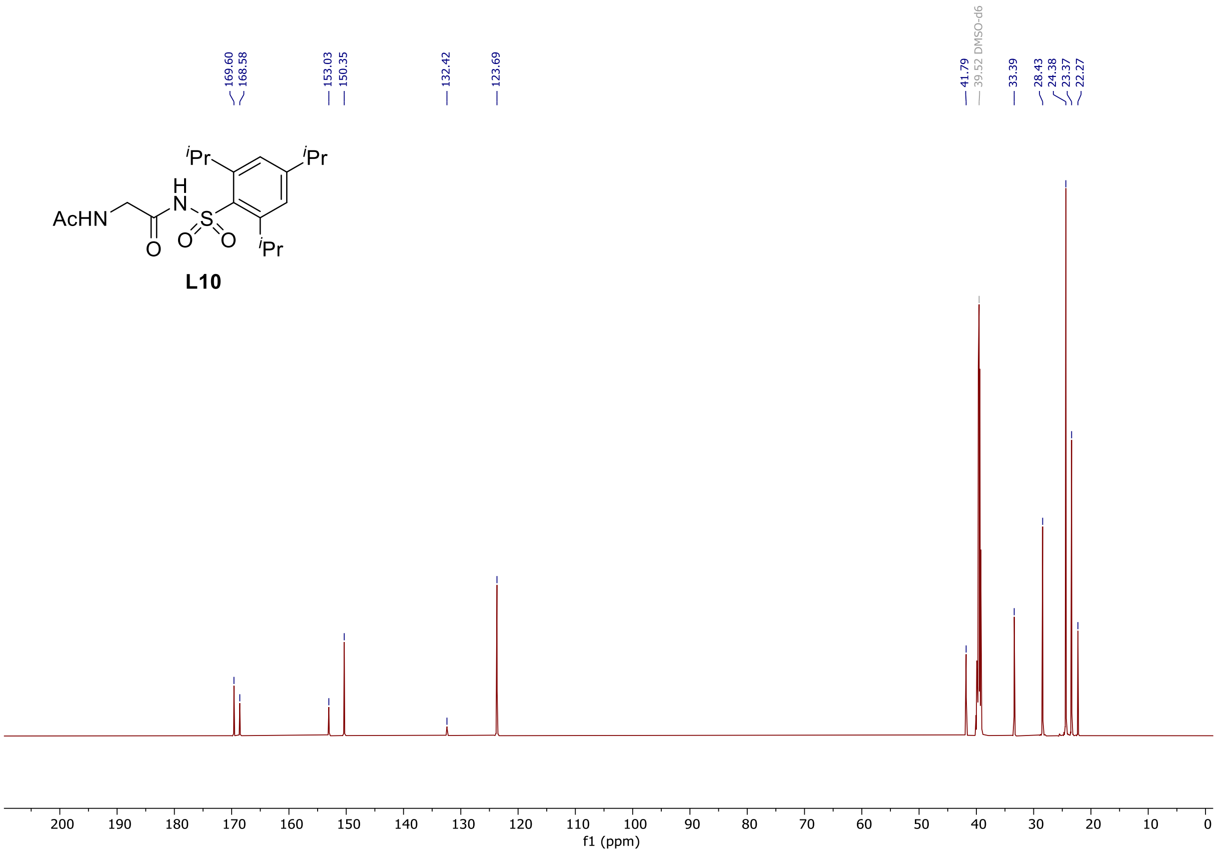
169.60
168.58
153.03
150.35
132.42
123.69
41.79
39.52 DMSO-d6
33.39
28.43
24.38
23.37
22.27
AcHN
L10
f1 (ppm)

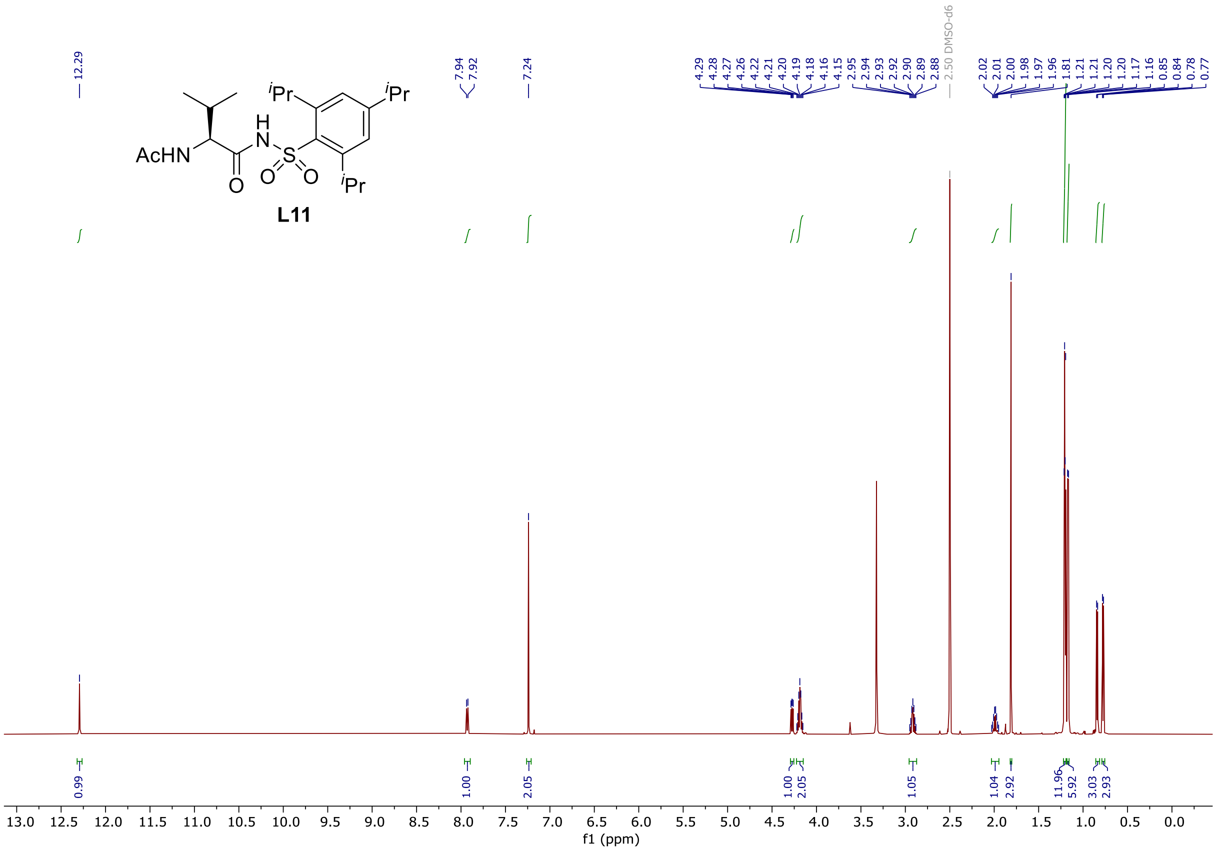

AcHN
H
N
S
O
O
O
iPr
iPr
iPr
L11
12.29
7.94
7.92
7.24
4.29 4.28 4.27 4.26 4.22 4.21 4.20 4.19 4.18 4.16 4.15
2.95 2.94 2.93 2.92 2.90 2.89 2.88
2.50 DMSO-d6
2.02 2.01 2.00 1.98 1.97 1.96 1.81 1.21 1.21 1.21 1.20 1.20 1.17 1.16 0.85 0.84 0.78 0.77
0.99
1.00
2.05
1.00
2.05
1.05
1.04
2.92
11.96
5.92
3.03
2.93
f1 (ppm)

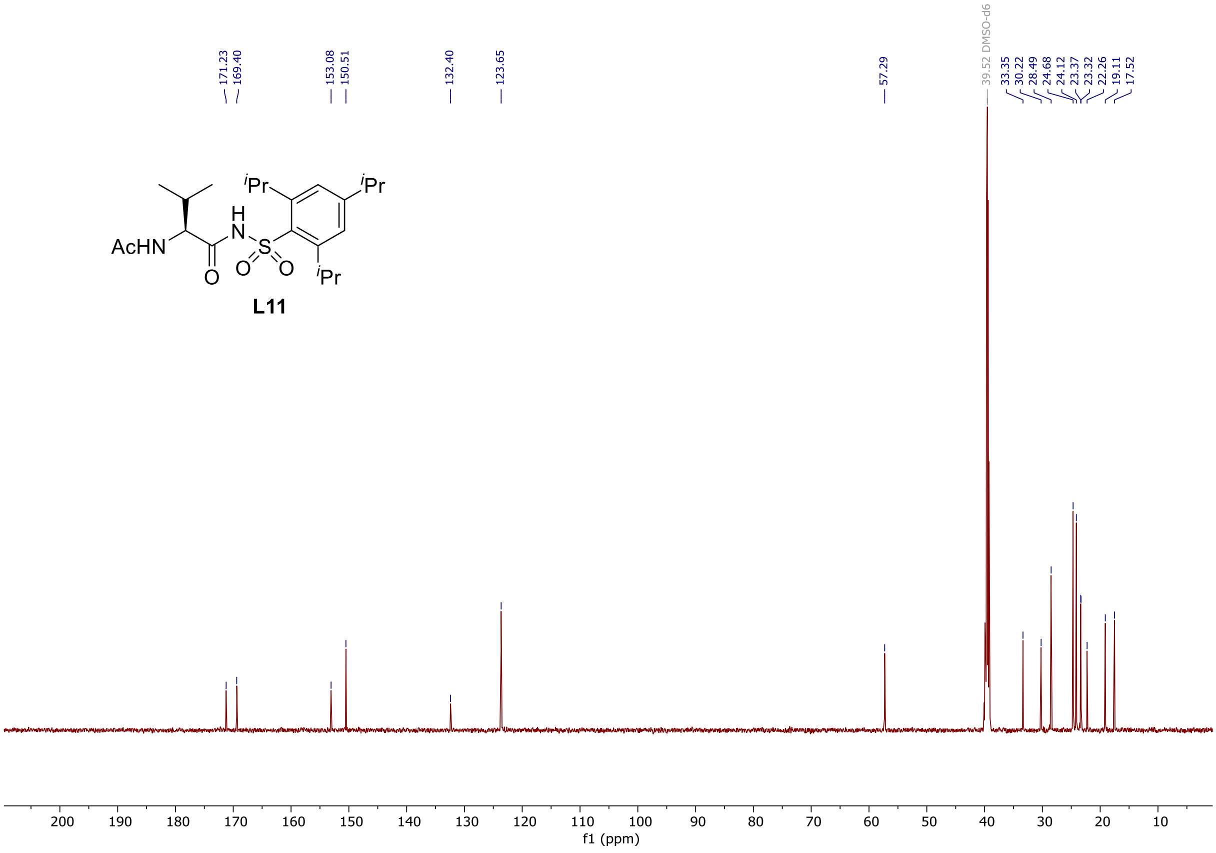

AcHN
H
N
S
O
O
O
iPr
iPr
iPr
L11
171.23
169.40
153.08
150.51
132.40
123.65
57.29
39.52 DMSO-d6
33.35 30.22 28.49 24.68 24.12 23.37 23.32 22.26 19.11 17.52
f1 (ppm)

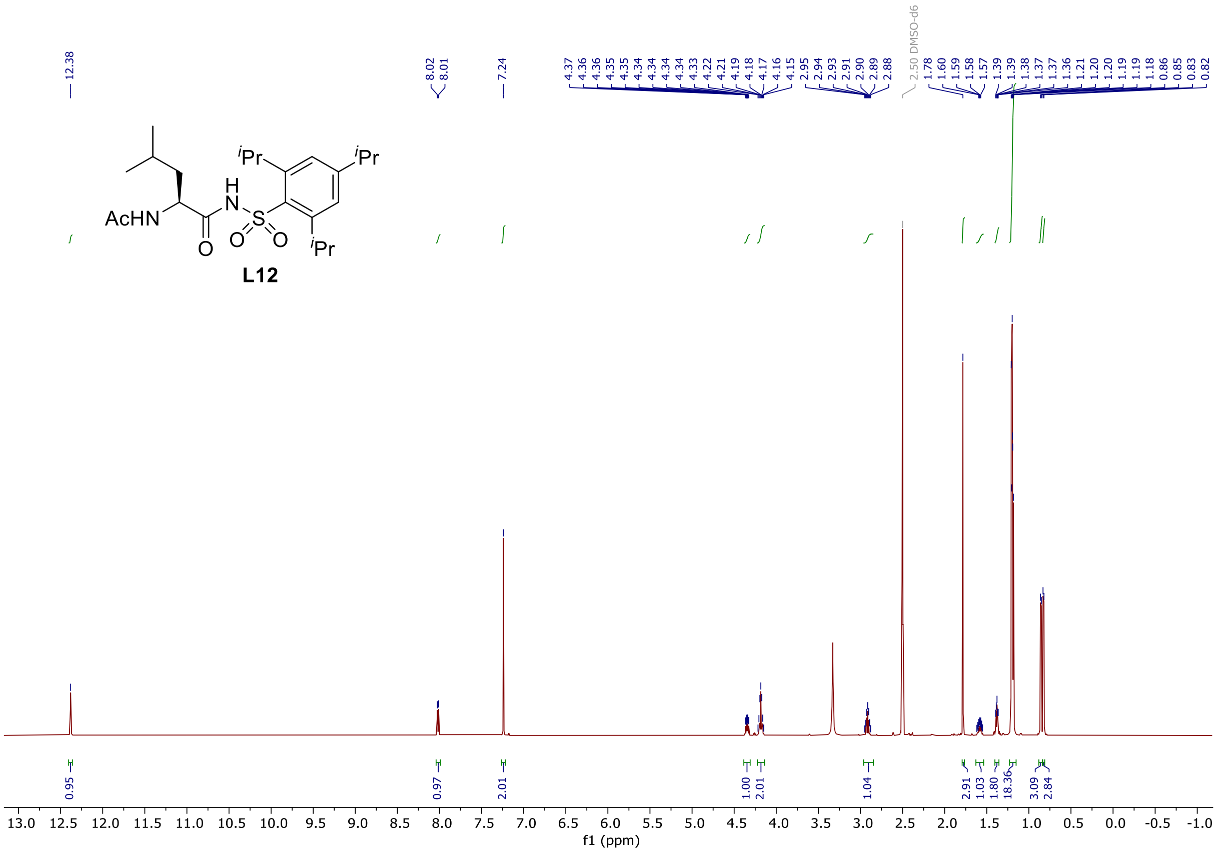
12.38
8.02
8.01
7.24
4.37 4.36 4.35 4.35 4.34 4.34 4.34 4.34 4.33 4.22 4.21 4.19 4.18 4.17 4.16 4.15
2.95 2.94 2.93 2.91 2.90 2.89 2.88
2.50 DMSO-d6
1.78 1.60 1.59 1.58 1.57 1.39 1.39 1.38 1.37 1.37 1.36 1.21 1.20 1.20 1.19 1.19 1.18 0.86 0.85 0.83 0.82
AcHN
L12
0.95
0.97
2.01
1.00
2.01
1.04
2.91
1.03
1.80
18.36
3.09
2.84
f1 (ppm)

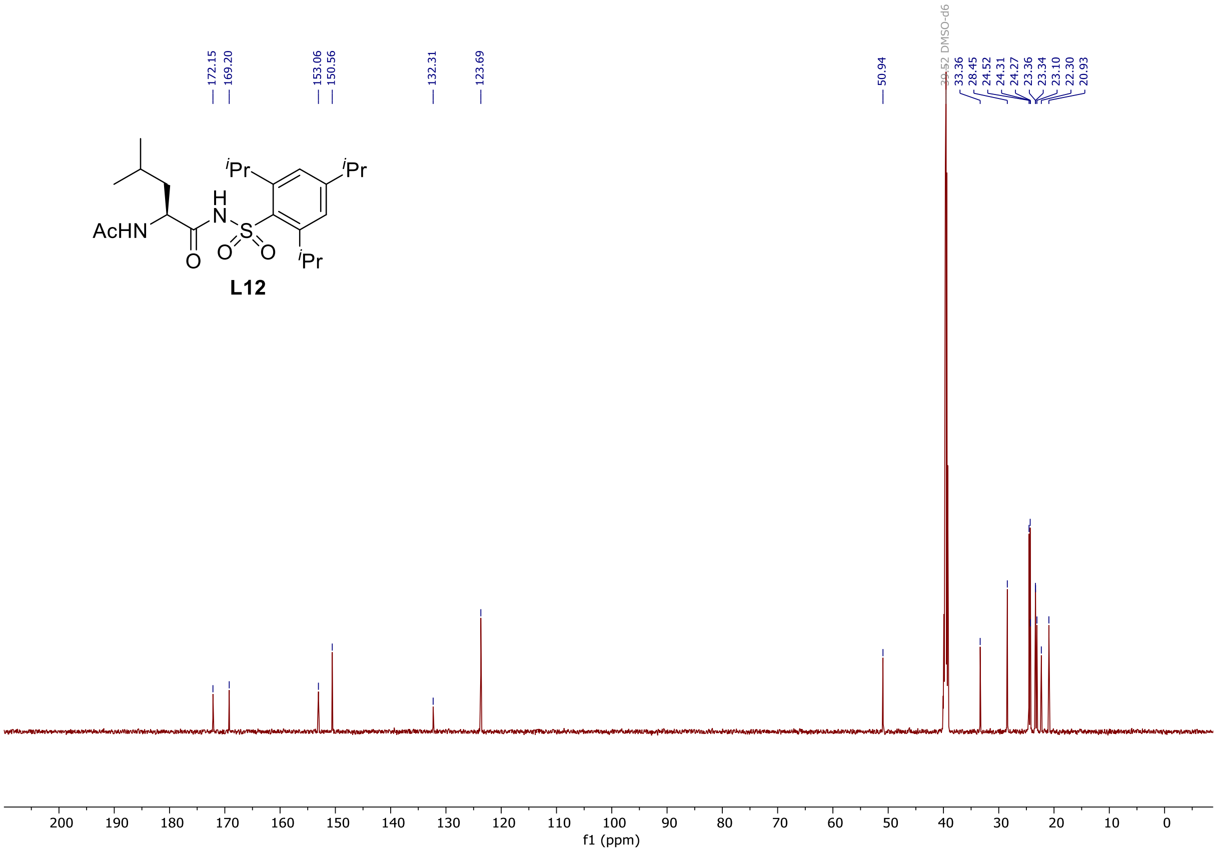
172.15
169.20
153.06
150.56
132.31
123.69
50.94
39.52 DMSO-d6
33.36 28.45 24.52 24.31 24.27 23.36 23.34 23.10 22.30 20.93
AcHN
L12
f1 (ppm)

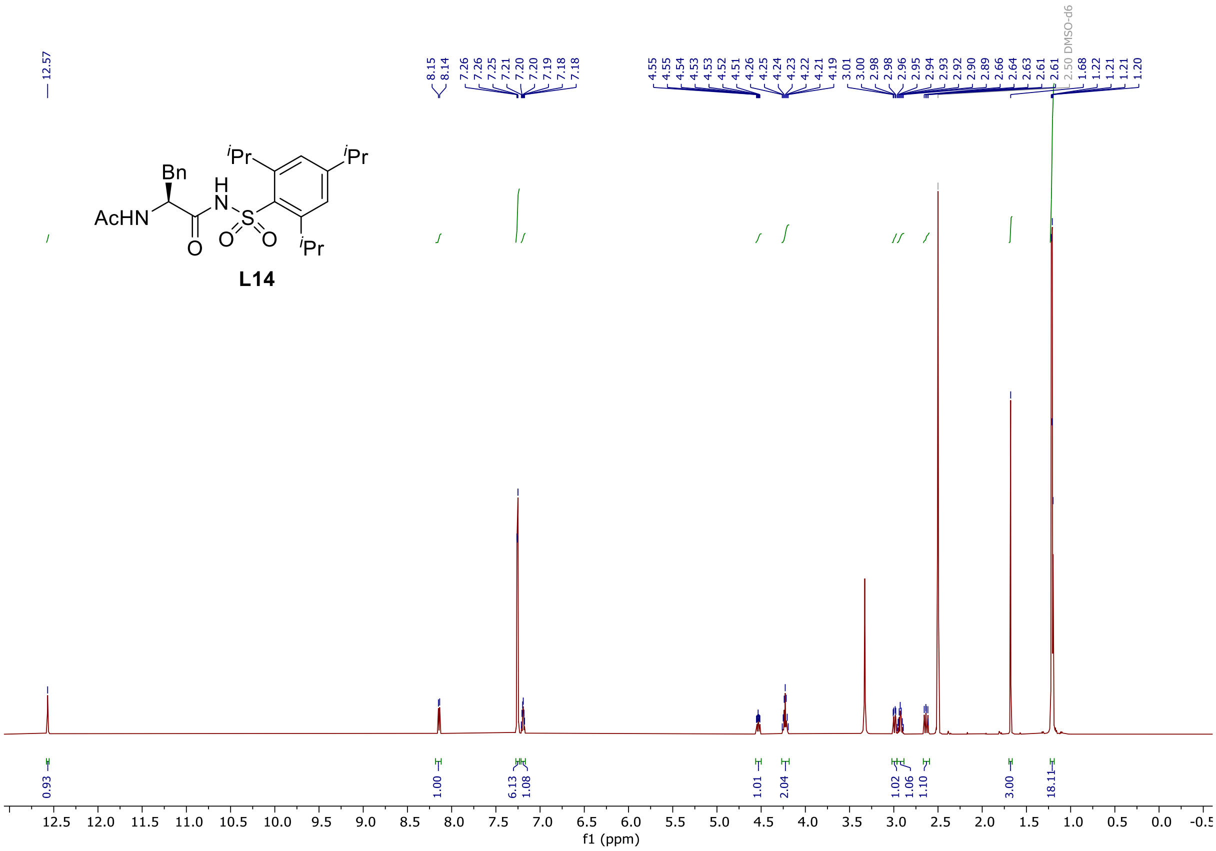

12.57
8.15
8.14
7.26
7.26
7.25
7.21
7.20
7.20
7.19
7.18
7.18
4.55
4.55
4.54
4.53
4.53
4.52
4.51
4.26
4.25
4.24
4.23
4.22
4.21
4.19
3.01
3.00
2.98
2.98
2.96
2.95
2.94
2.93
2.92
2.90
2.89
2.66
2.64
2.63
2.61
2.61
2.50 DMSO-d6
1.68
1.22
1.21
1.21
1.20
Bn
AcHN
H
N
S
O
O
O
iPr
iPr
iPr
L14
0.93
1.00
6.13
1.08
1.01
2.04
1.02
1.06
1.10
3.00
18.11
12.5
12.0
11.5
11.0
10.5
10.0
9.5
9.0
8.5
8.0
7.5
7.0
6.5
6.0
5.5
5.0
4.5
4.0
3.5
3.0
2.5
2.0
1.5
1.0
0.5
0.0
-0.5
f1 (ppm)

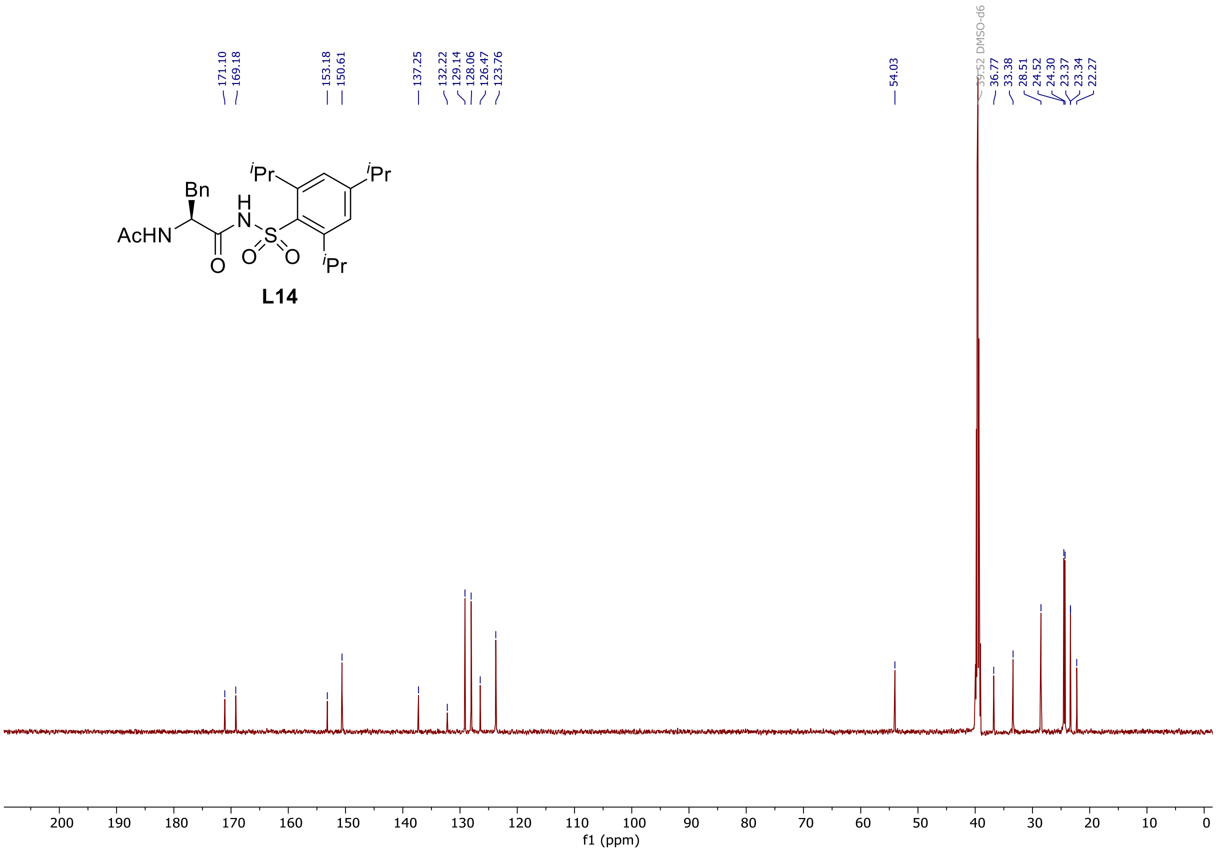

171.10
169.18
153.18
150.61
137.25
132.22
129.14
128.06
126.47
123.76
54.03
39.52 DMSO-d6
36.77
33.38
28.51
24.52
24.30
23.37
23.34
22.27
Bn
AcHN
H
N
S
O
O
O
iPr
iPr
iPr
L14
200
190
180
170
160
150
140
130
120
110
100
90
80
70
60
50
40
30
20
10
0
f1 (ppm)

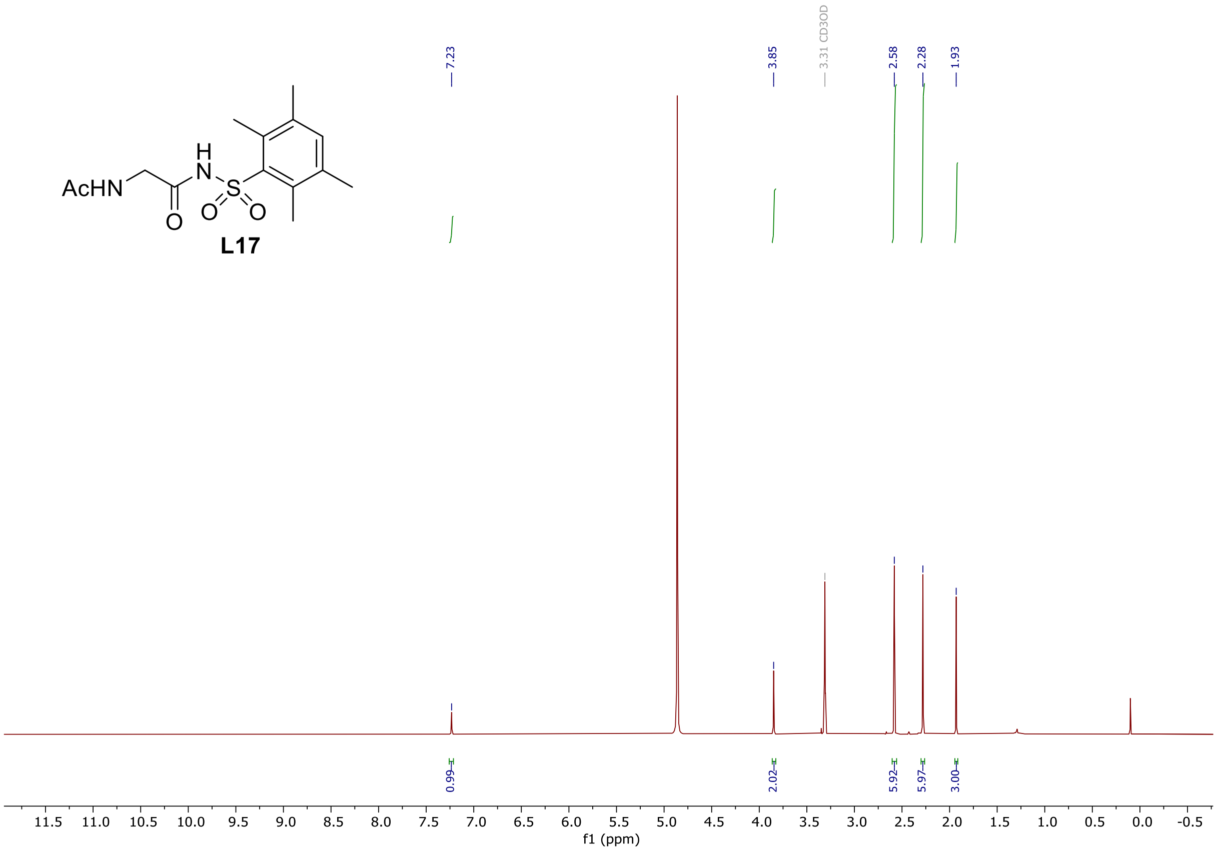
7.23
3.85
3.31 CD3OD
2.58
2.28
1.93
AcHN
H
N
S
O
O
O
L17
0.99
2.02
5.92
5.97
3.00
f1 (ppm)

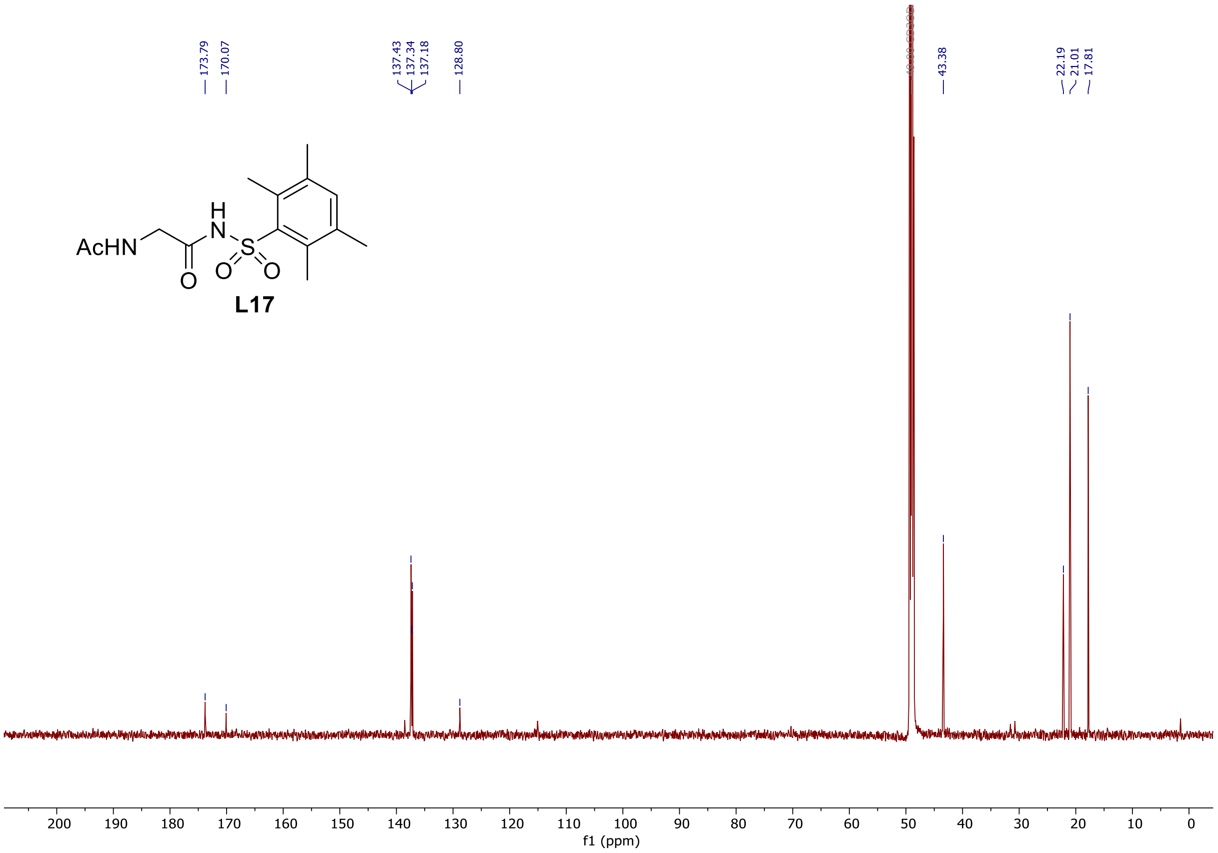
173.79
170.07
137.43
137.34
137.18
128.80
43.38
22.19
21.01
17.81
AcHN
H
N
S
O
O
O
L17
f1 (ppm)

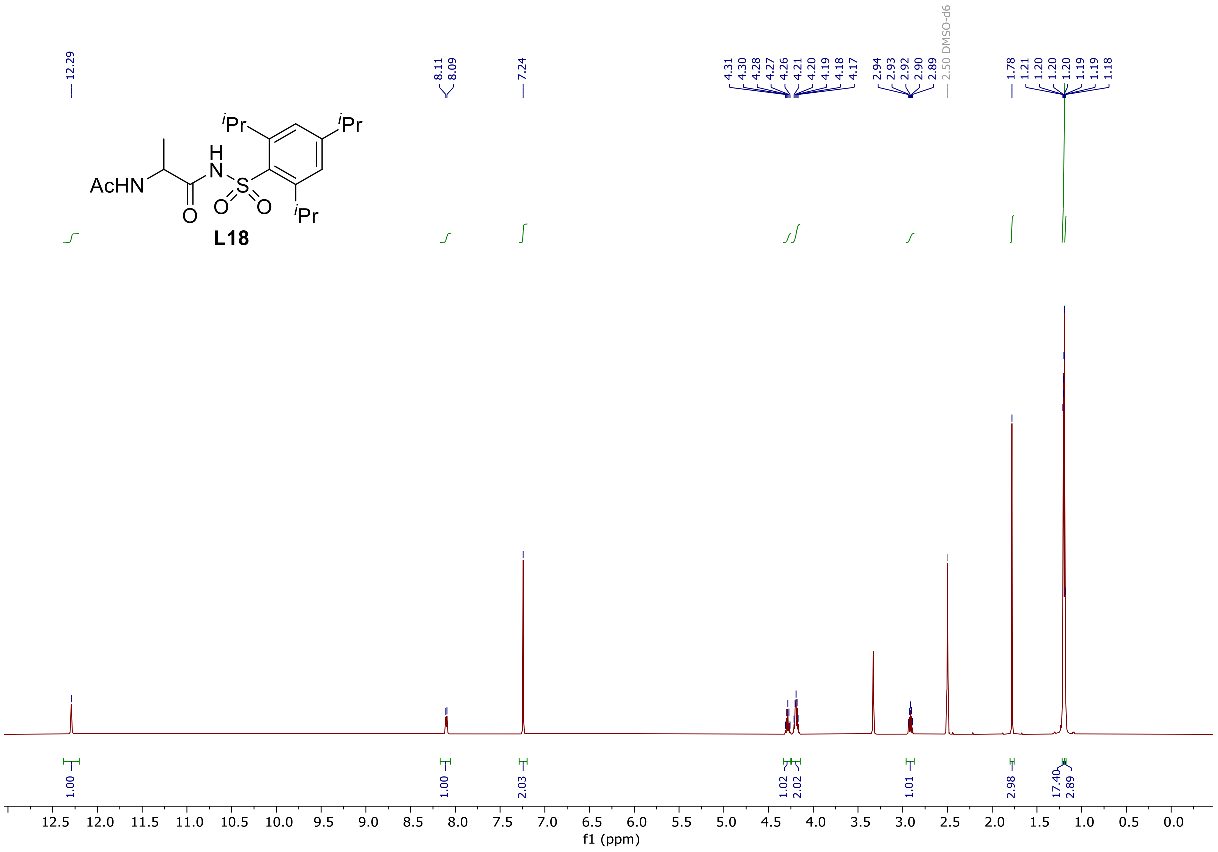
12.29
8.11
8.09
7.24
4.31
4.30
4.28
4.27
4.26
4.21
4.20
4.19
4.18
4.17
2.94
2.93
2.92
2.90
2.89
2.50 DMSO-d6
1.78
1.21
1.20
1.20
1.20
1.19
1.19
1.18
iPr
iPr
H
N
AcHN
O
S
O
O
iPr
L18
1.00
1.00
2.03
1.02
2.02
1.01
2.98
17.40
2.89
f1 (ppm)

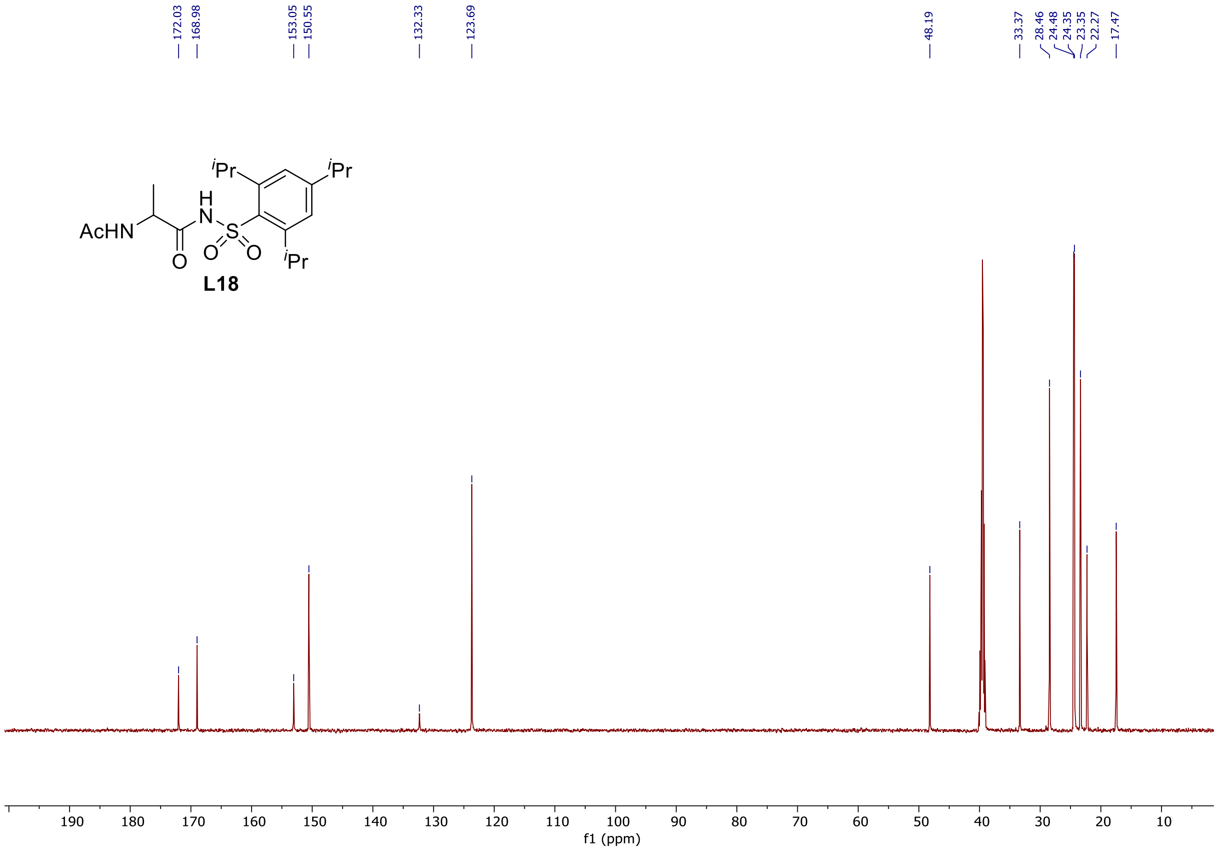
172.03
168.98
153.05
150.55
132.33
123.69
48.19
33.37
28.46
24.48
24.35
23.35
22.27
17.47
iPr
iPr
H
N
AcHN
O
S
O
O
iPr
L18
f1 (ppm)

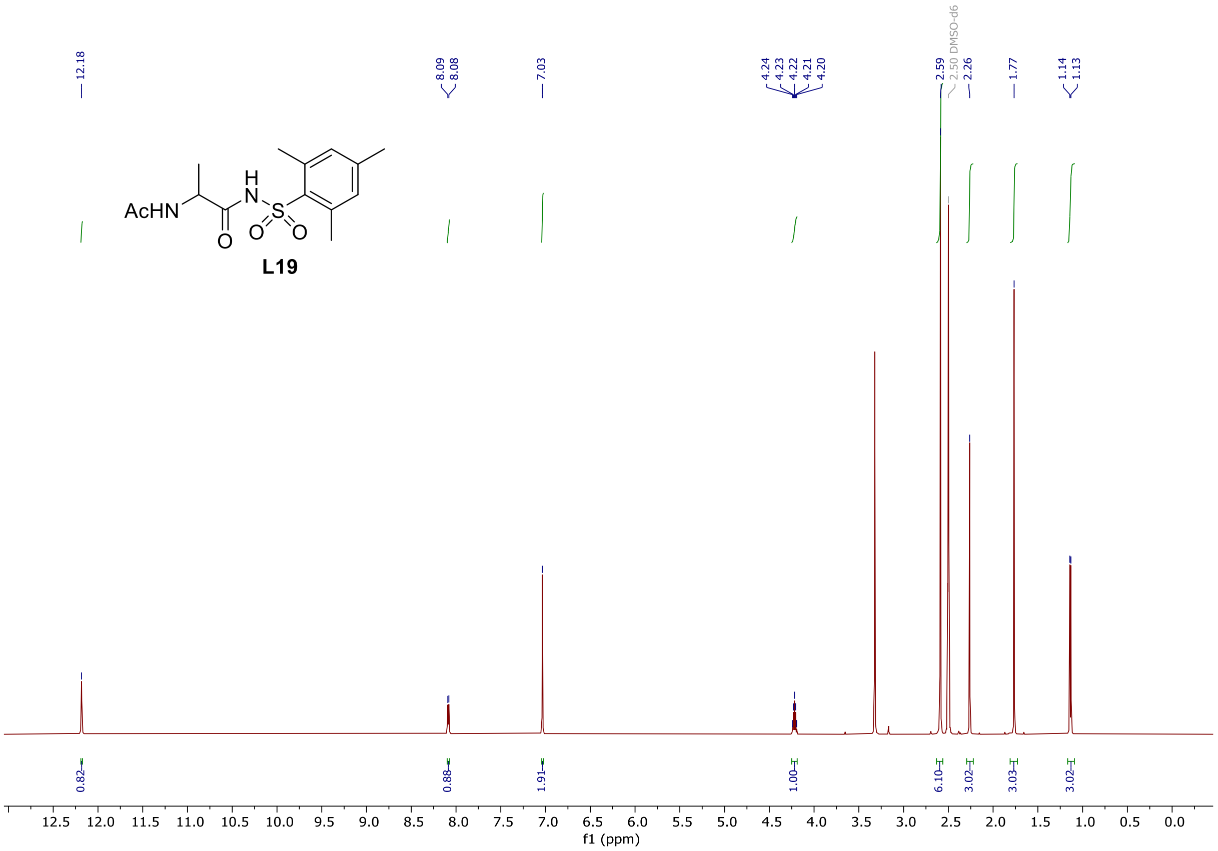
12.18
8.09
8.08
7.03
4.24
4.23
4.22
4.21
4.20
2.59
2.50 DMSO-d6
2.26
1.77
1.14
1.13
AcHN
H
N
S
O
O
O
L19
0.82
0.88
1.91
1.00
6.10
3.02
3.03
3.02
f1 (ppm)

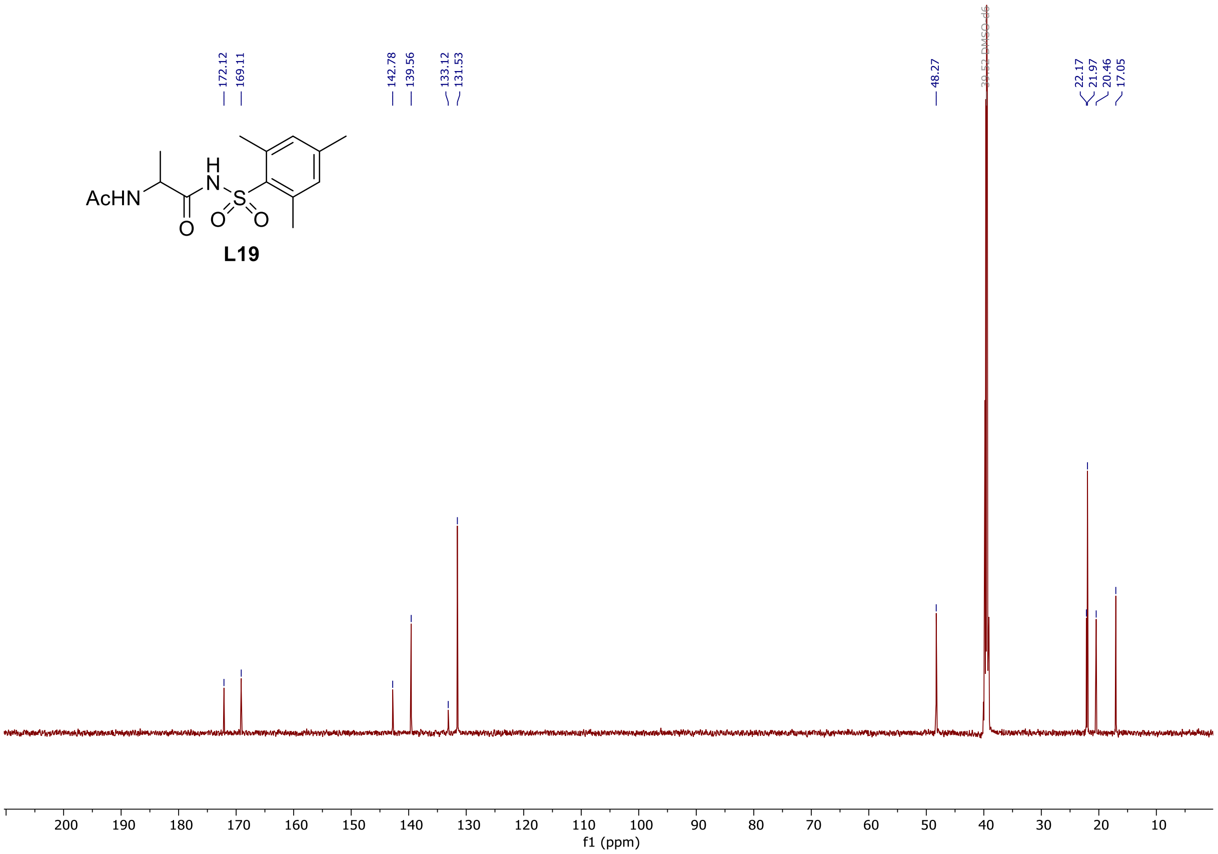
172.12
169.11
142.78
139.56
133.12
131.53
48.27
39.52 DMSO-d6
22.17
21.97
20.46
17.05
AcHN
H
N
S
O
O
O
L19
f1 (ppm)

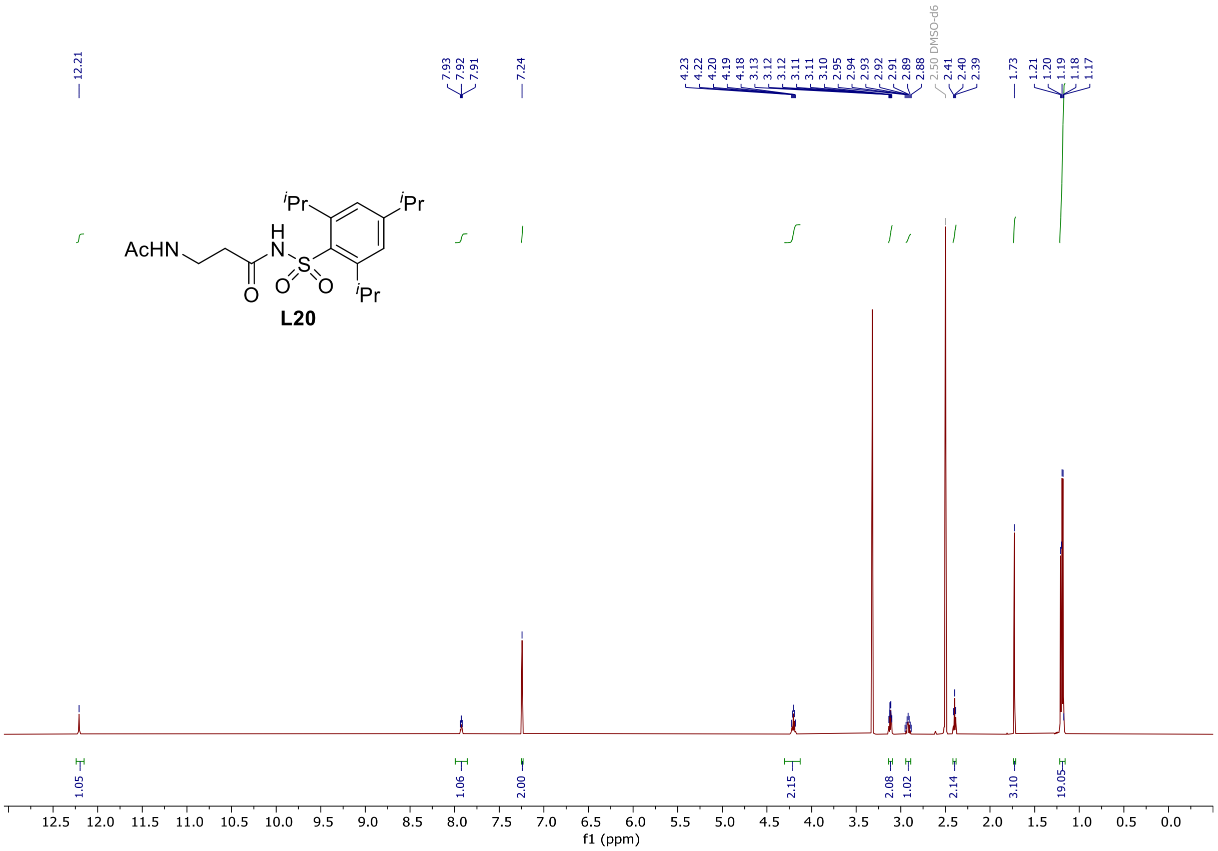
12.21
7.93 7.92 7.91
7.24
4.23 4.22 4.20 4.19 4.18 3.13 3.12 3.12 3.11 3.11 3.10 2.95 2.94 2.93 2.92 2.91 2.89 2.88
2.50 DMSO-d6
2.41 2.40 2.39
1.73
1.21 1.20 1.19 1.18 1.17
AcHN
H
N
S
O O
O
^{i}Pr
^{i}Pr
^{i}Pr
L20
1.05
1.06
2.00
2.15
2.08
1.02
2.14
3.10
19.05
12.5 12.0 11.5 11.0 10.5 10.0 9.5 9.0 8.5 8.0 7.5 7.0 6.5 6.0 5.5 5.0 4.5 4.0 3.5 3.0 2.5 2.0 1.5 1.0 0.5 0.0
f1 (ppm)

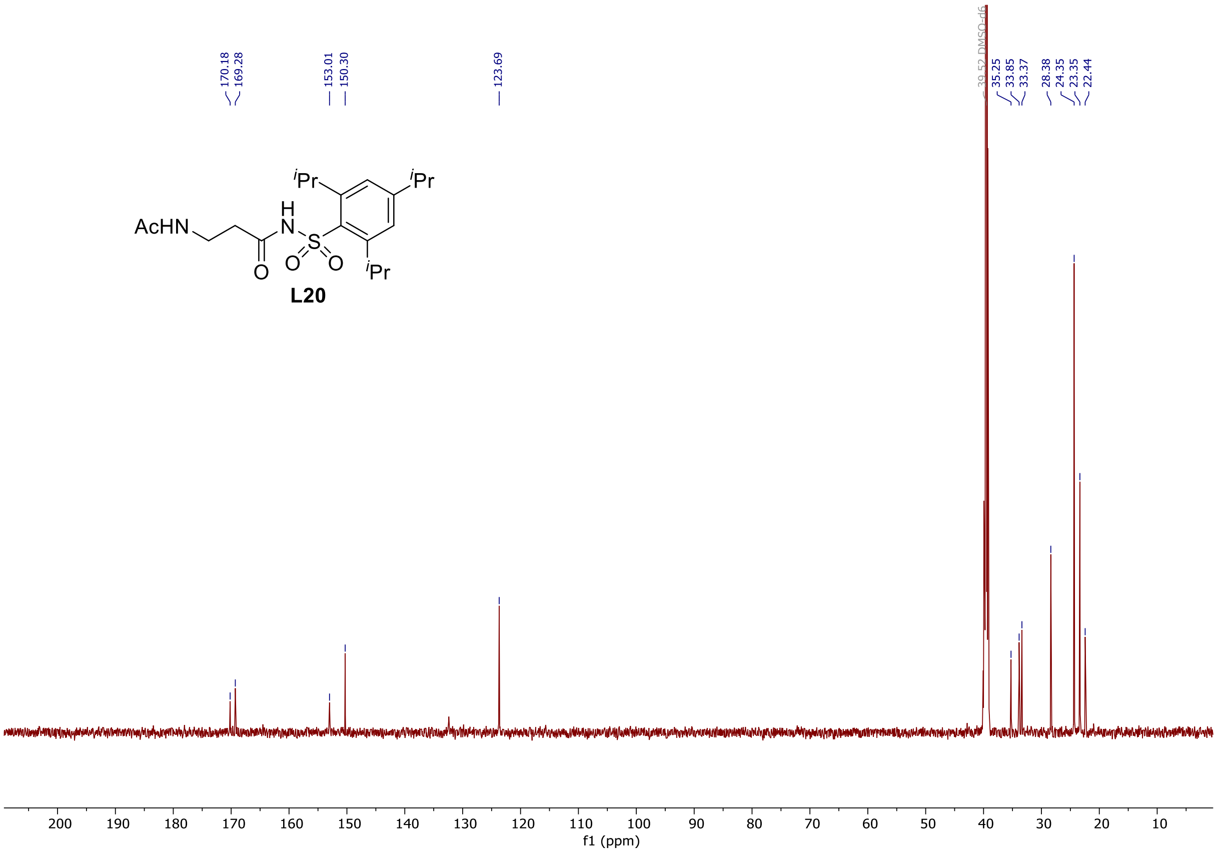
170.18
169.28
153.01
150.30
123.69
39.52 DMSO-d6
35.25
33.85
33.37
28.38
24.35
23.35
22.44
AcHN
H
N
S
O O
O
^{i}Pr
^{i}Pr
^{i}Pr
L20
200 190 180 170 160 150 140 130 120 110 100 90 80 70 60 50 40 30 20 10
f1 (ppm)

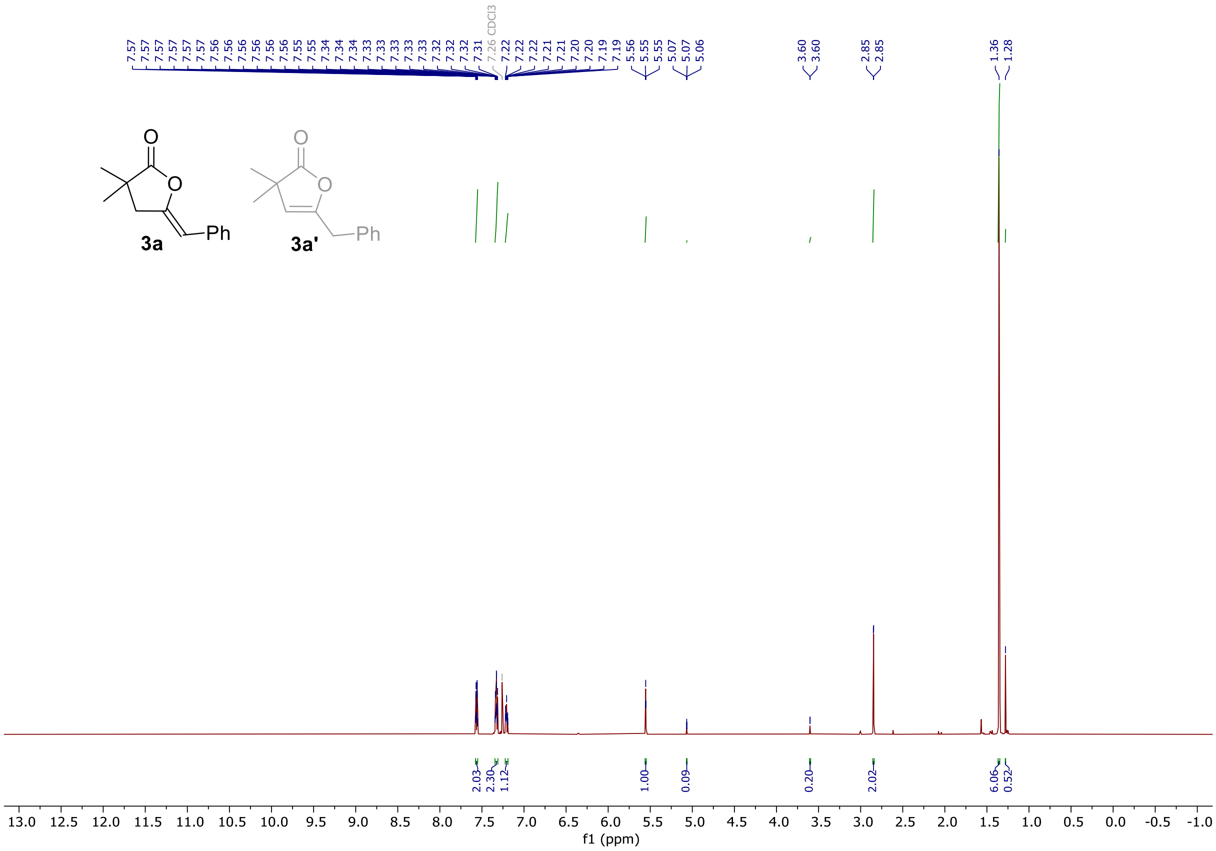

7.57
7.57
7.57
7.57
7.57
7.57
7.56
7.56
7.56
7.56
7.56
7.56
7.55
7.55
7.34
7.34
7.34
7.33
7.33
7.33
7.33
7.32
7.32
7.32
7.32
7.31
7.26 CDCl3
7.22
7.22
7.22
7.22
7.21
7.21
7.20
7.20
7.19
5.56
5.55
5.55
5.07
5.07
5.06
3.60
3.60
2.85
2.85
1.36
1.28
3a
3a'
Ph
2.03
2.30
1.12
1.00
0.09
0.20
2.02
6.06
0.52
f1 (ppm)

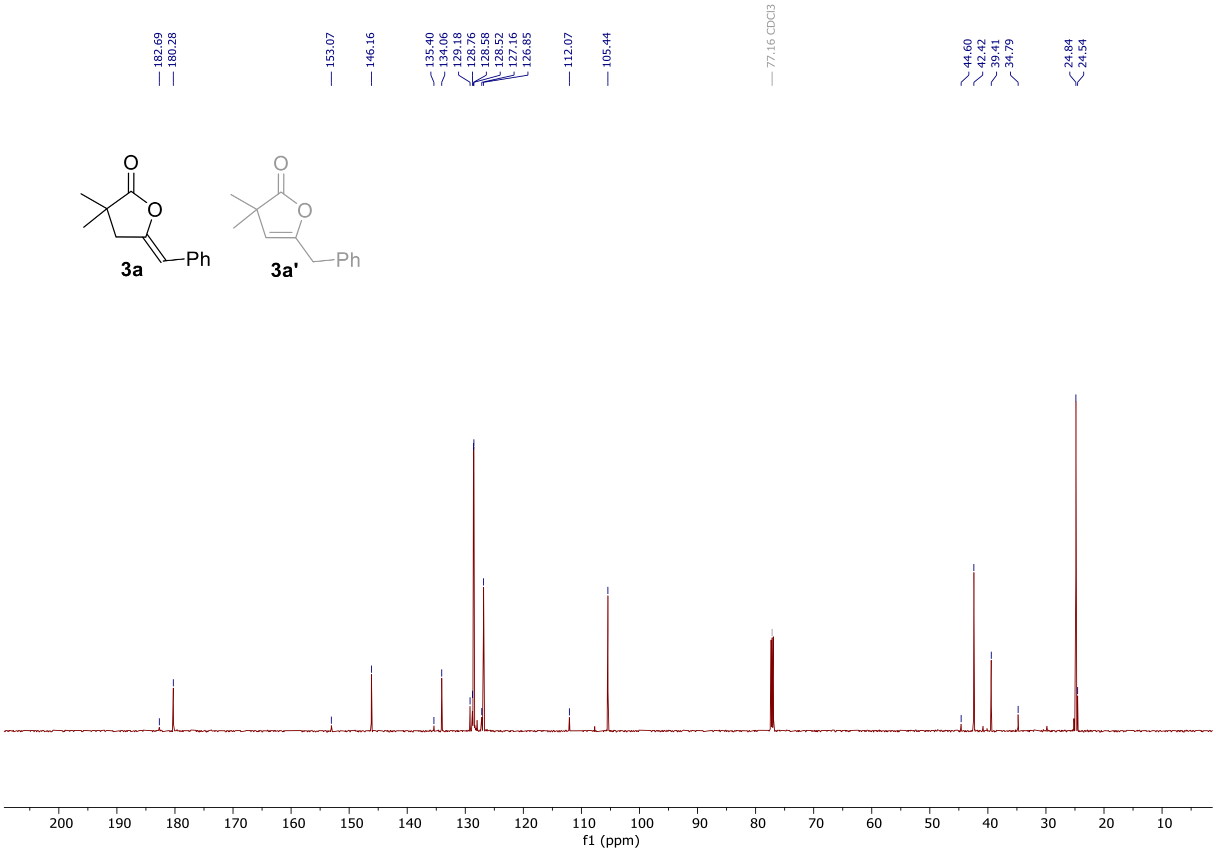

182.69
180.28
153.07
146.16
135.40
134.06
129.18
128.76
128.58
128.52
127.16
126.85
112.07
105.44
77.16 CDCl3
44.60
42.42
39.41
34.79
24.84
24.54
3a
3a'
Ph
f1 (ppm)

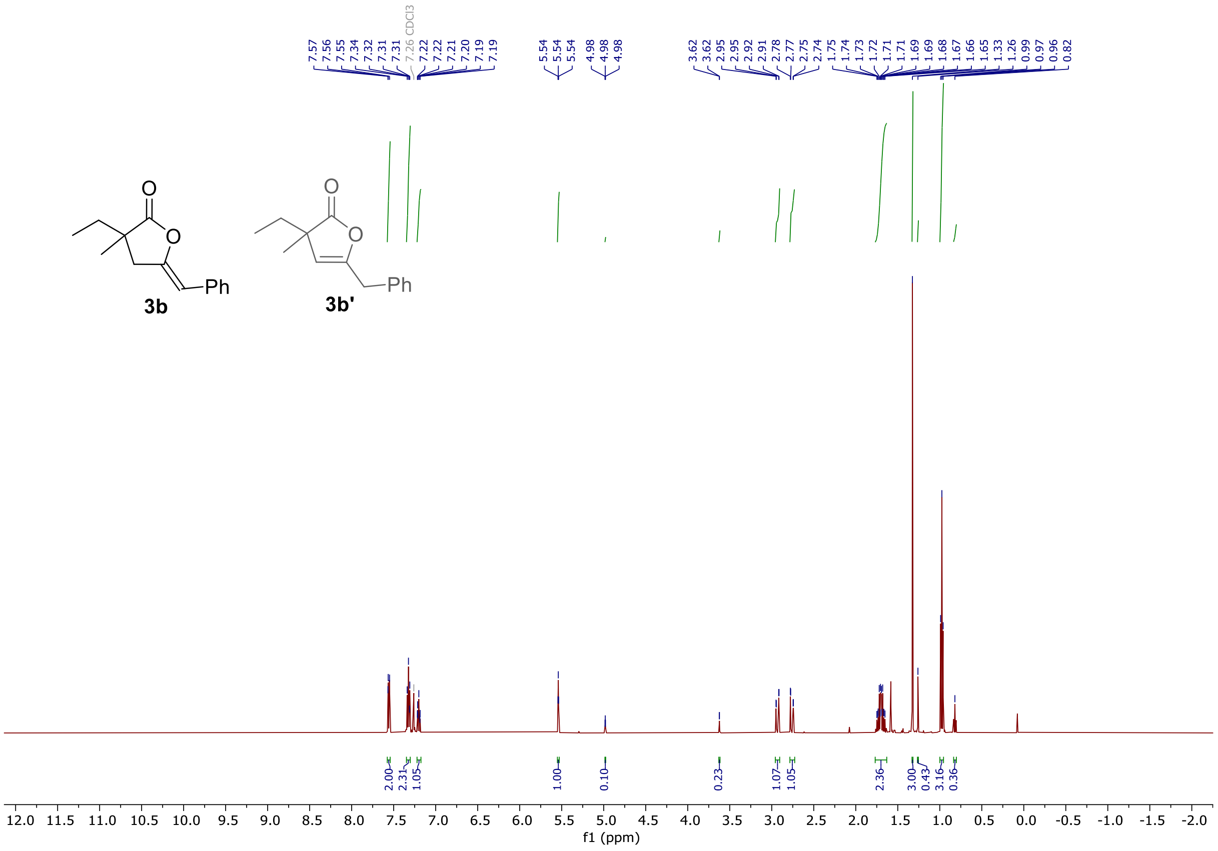
7.57 7.56 7.55 7.34 7.32 7.31 7.26 CDCl3 7.22 7.22 7.21 7.20 7.19 7.19 5.54 5.54 5.54 4.98 4.98 4.98 3.62 3.62 2.95 2.95 2.92 2.91 2.78 2.77 2.75 2.74 1.75 1.74 1.73 1.72 1.71 1.71 1.69 1.69 1.68 1.67 1.66 1.65 1.33 1.26 0.99 0.97 0.96 0.82
O
O
Ph
3b
O
O
Ph
3b'
2.00 2.31 1.05 1.00 0.10 0.23 1.07 1.05 2.36 3.00 0.43 3.16 0.36
12.0 11.5 11.0 10.5 10.0 9.5 9.0 8.5 8.0 7.5 7.0 6.5 6.0 5.5 5.0 4.5 4.0 3.5 3.0 2.5 2.0 1.5 1.0 0.5 0.0 -0.5 -1.0 -1.5 -2.0
f1 (ppm)

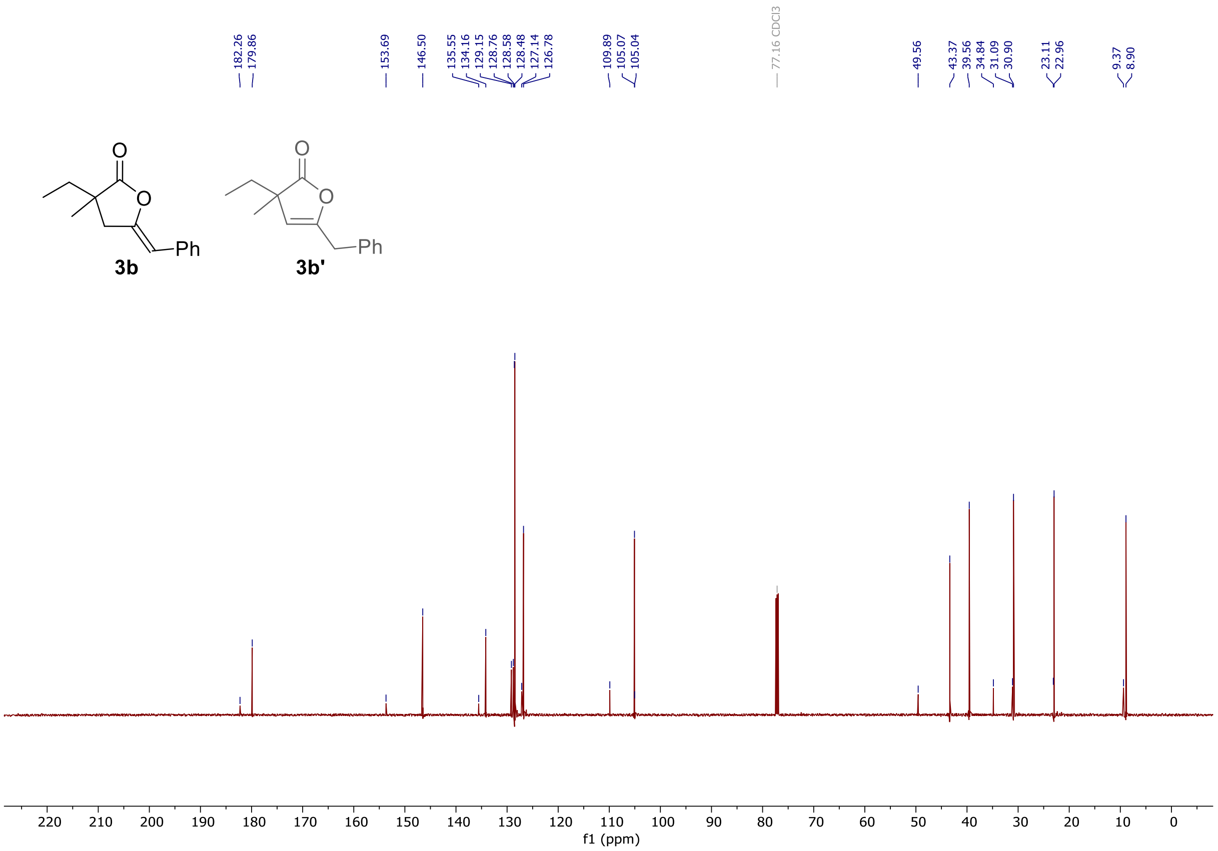
182.26 179.86 153.69 146.50 135.55 134.16 129.15 128.76 128.58 128.48 127.14 126.78 109.89 105.07 105.04 77.16 CDCl3 49.56 43.37 39.56 34.84 31.09 30.90 23.11 22.96 9.37 8.90
O
O
Ph
3b
O
O
Ph
3b'
220 210 200 190 180 170 160 150 140 130 120 110 100 90 80 70 60 50 40 30 20 10 0
f1 (ppm)

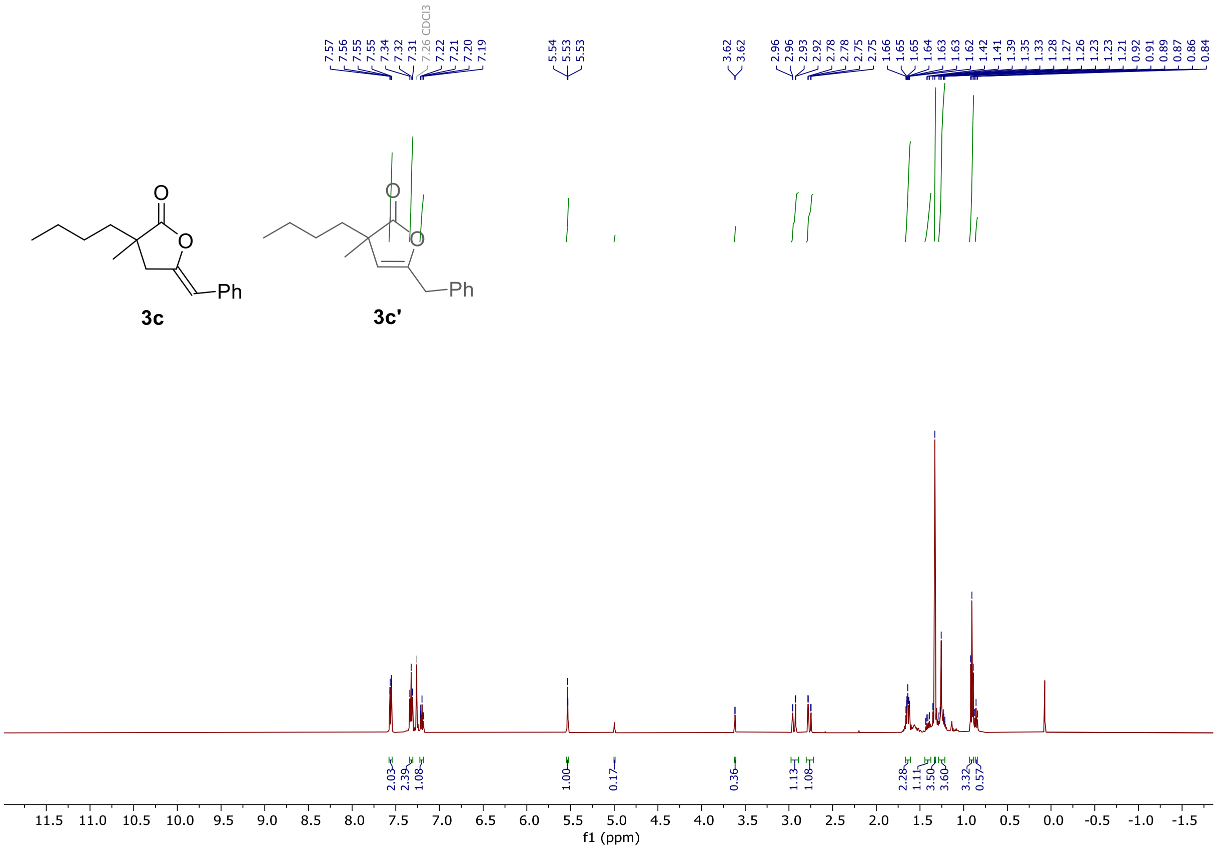
7.57 7.56 7.55 7.55 7.34 7.32 7.31 7.26 CDCl3 7.22 7.21 7.20 7.19
5.54 5.53 5.53
3.62 3.62
2.96 2.96 2.93 2.92 2.78 2.78 2.75 2.75 1.66 1.65 1.64 1.63 1.63 1.62 1.42 1.41 1.39 1.35 1.33 1.28 1.27 1.26 1.23 1.23 1.21 0.92 0.91 0.89 0.87 0.86 0.84
O
O
Ph
3c
O
O
Ph
3c'
2.03 2.39 1.08 1.00 0.17 0.36 1.13 1.08 2.28 1.11 3.50 3.60 3.32 0.57
11.5 11.0 10.5 10.0 9.5 9.0 8.5 8.0 7.5 7.0 6.5 6.0 5.5 5.0 4.5 4.0 3.5 3.0 2.5 2.0 1.5 1.0 0.5 0.0 -0.5 -1.0 -1.5
f1 (ppm)

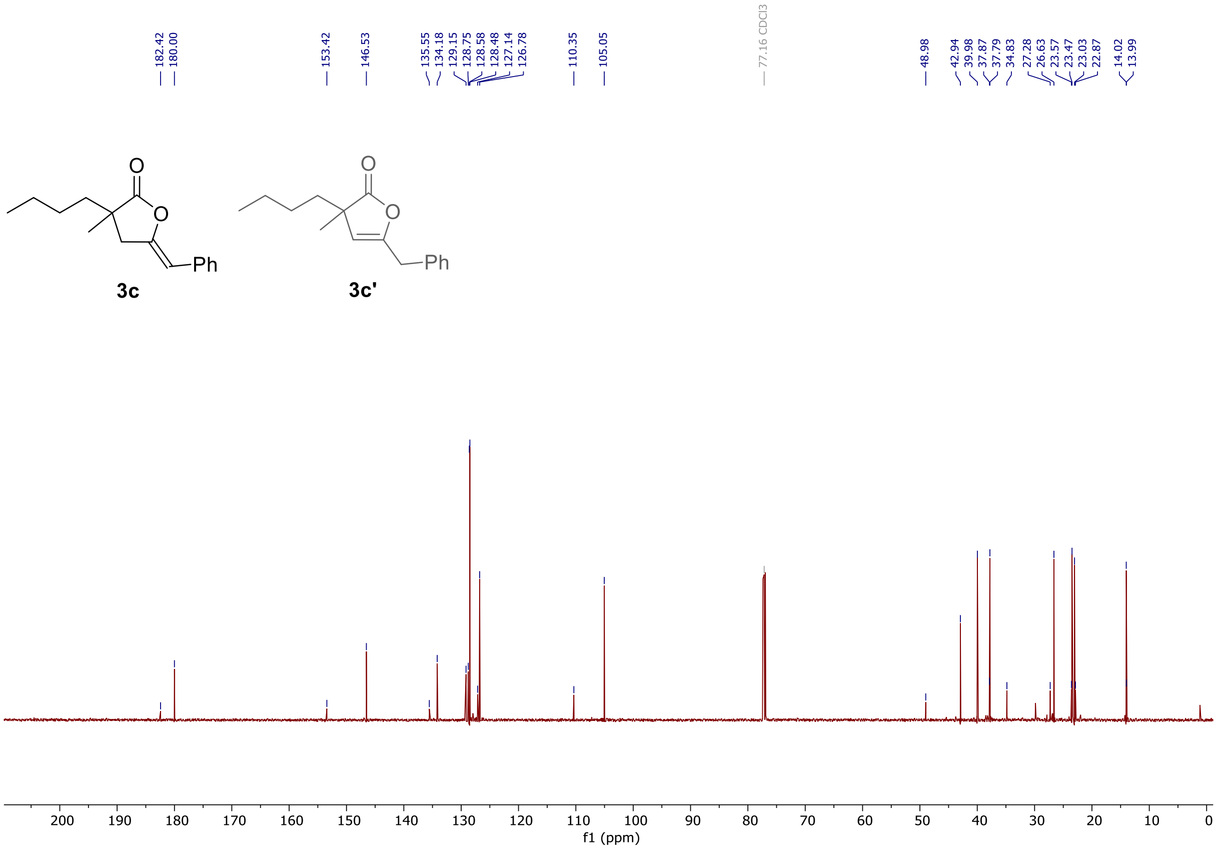
182.42 180.00
153.42 146.53
135.55 134.18 129.15 128.75 128.58 128.48 127.14 126.78
110.35 105.05
77.16 CDCl3
48.98
42.94 39.98 37.87 37.79 34.83 27.28 26.63 23.57 23.47 23.03 22.87 14.02 13.99
O
O
Ph
3c
O
O
Ph
3c'
200 190 180 170 160 150 140 130 120 110 100 90 80 70 60 50 40 30 20 10 0
f1 (ppm)

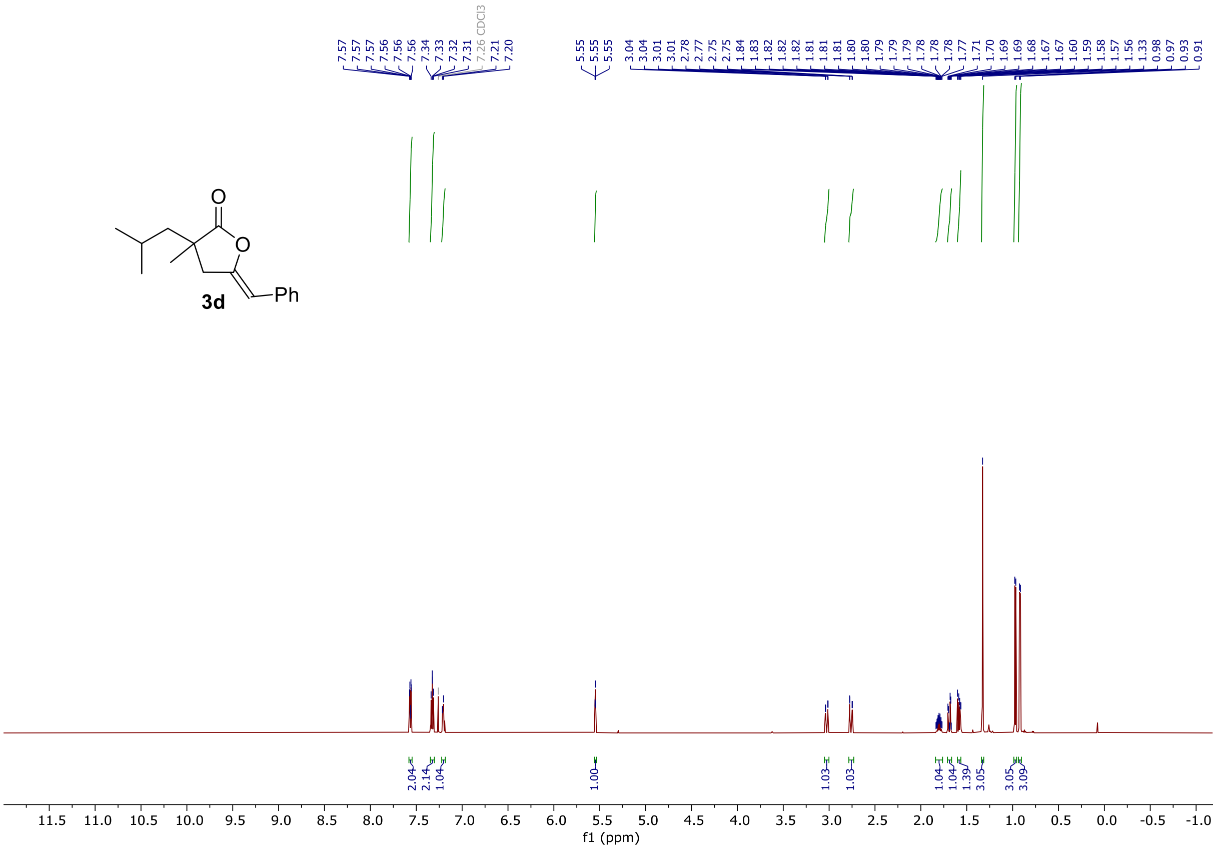

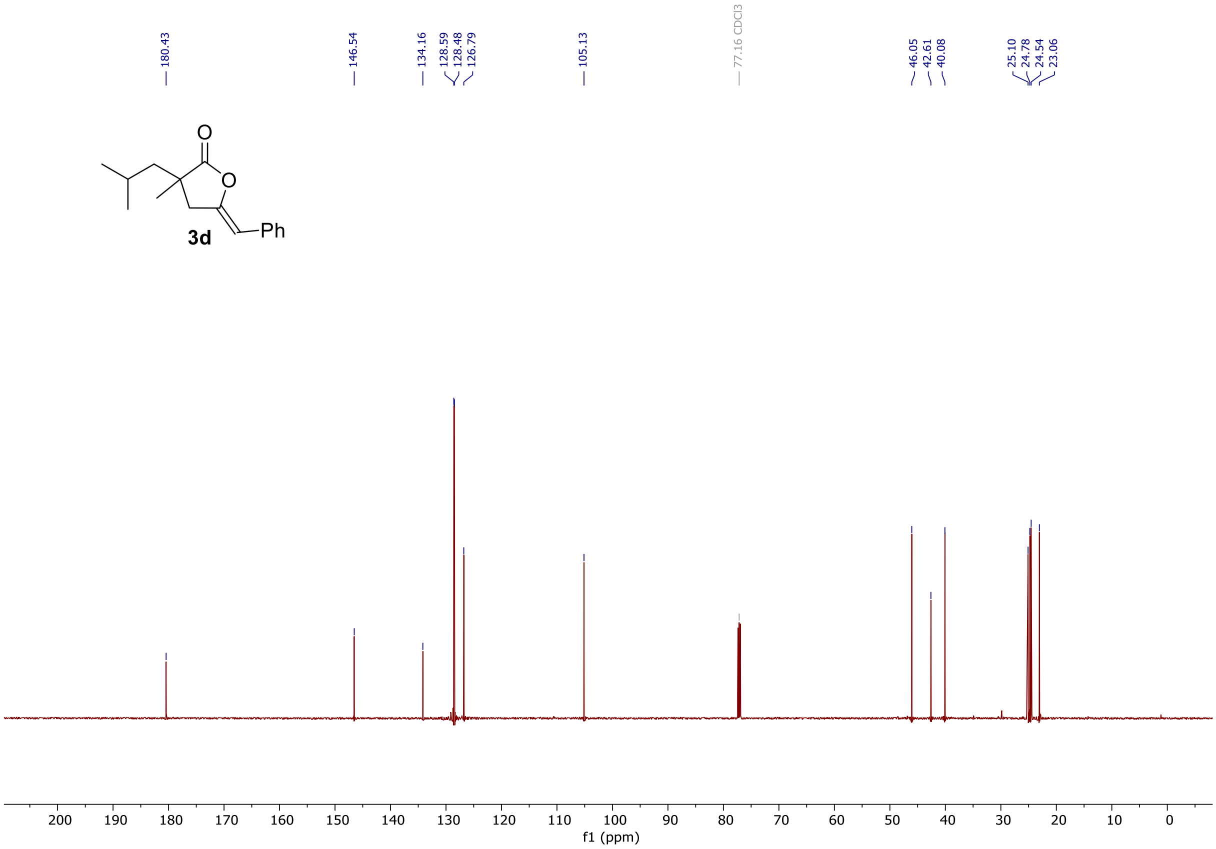

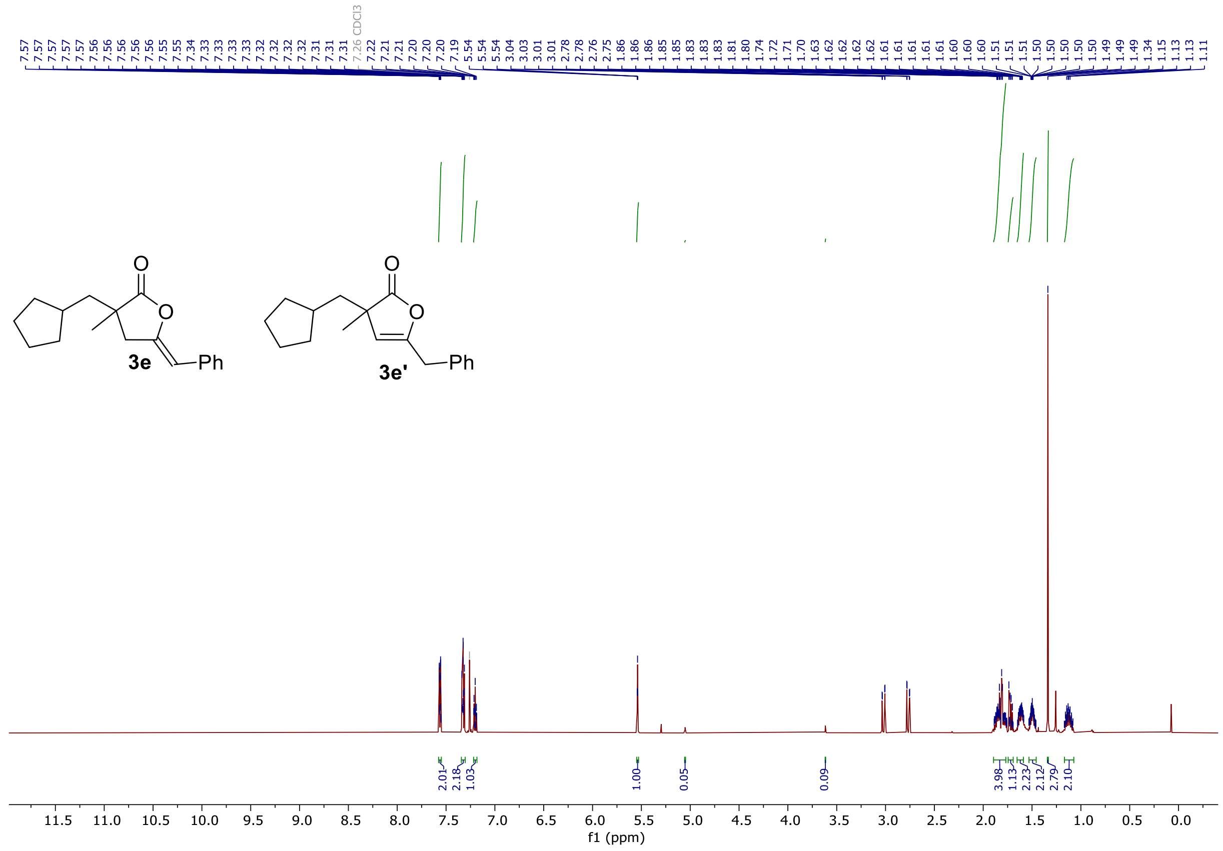

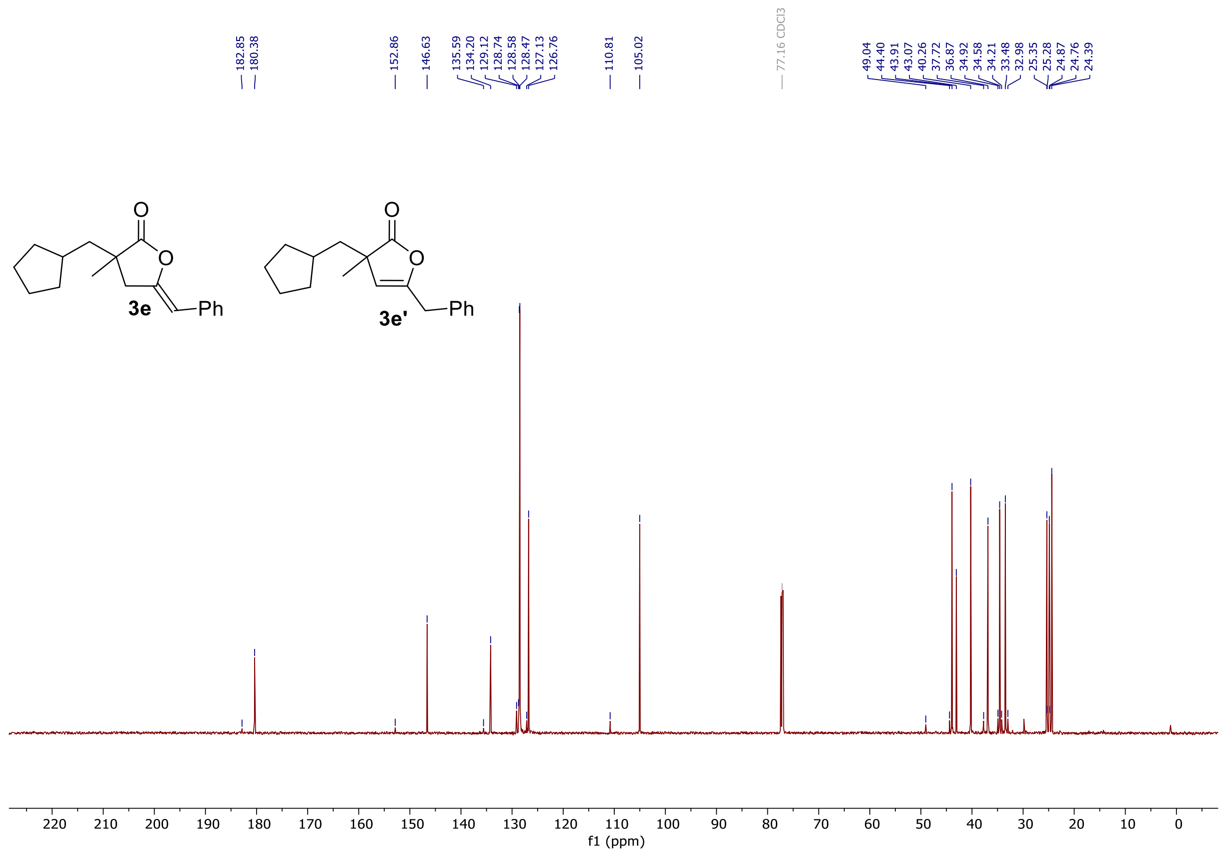

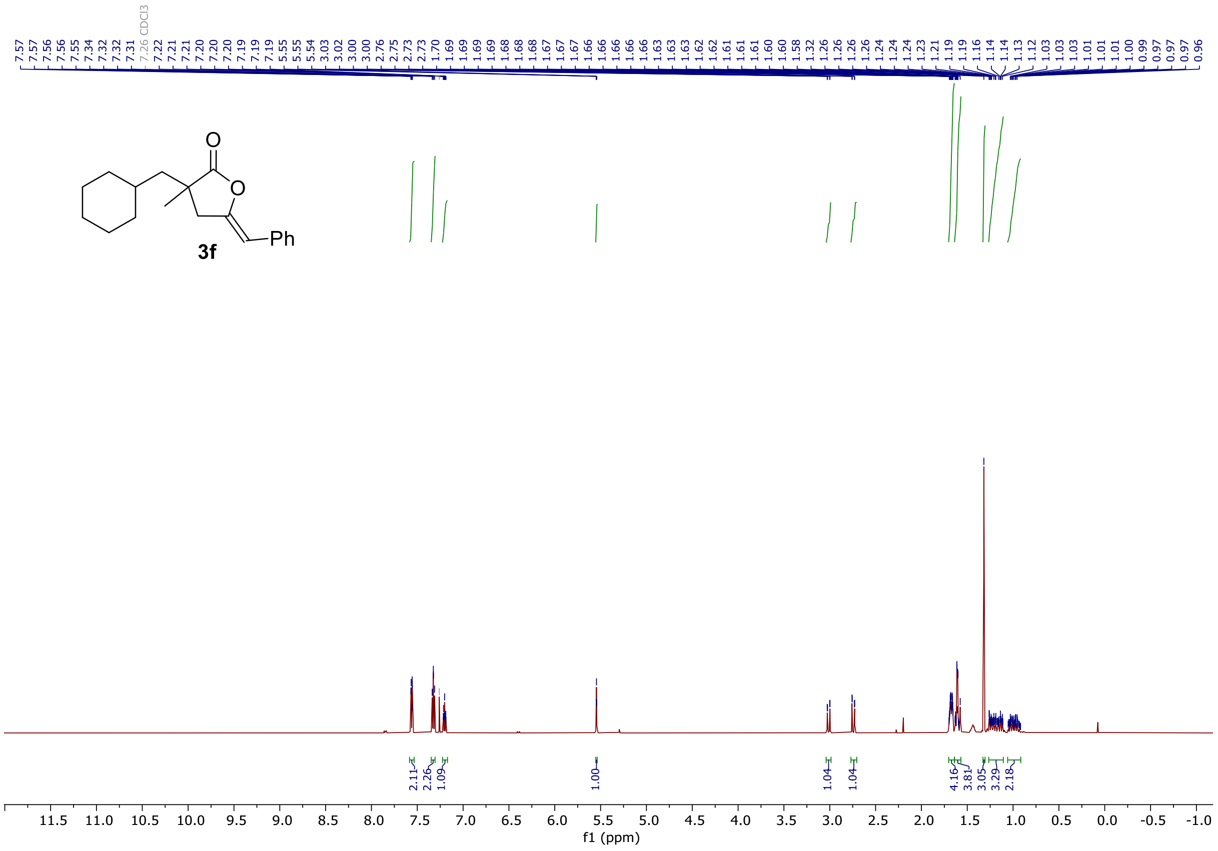
7.57 7.57 7.56 7.55 7.34 7.32 7.32 7.31 7.26 CDCl3 7.22 7.21 7.21 7.20 7.20 7.20 7.19 7.19 7.19 5.55 5.55 5.55 5.54 3.03 3.02 3.00 3.00 2.76 2.75 2.73 2.73 1.70 1.69 1.69 1.69 1.68 1.68 1.67 1.67 1.66 1.66 1.66 1.66 1.63 1.63 1.62 1.61 1.61 1.61 1.60 1.58 1.32 1.26 1.26 1.26 1.26 1.24 1.24 1.23 1.21 1.19 1.19 1.16 1.14 1.14 1.13 1.12 1.03 1.03 1.03 1.01 1.01 1.01 1.00 0.99 0.97 0.97 0.97 0.96
O
O
Ph
3f
2.11 2.26 1.09 1.00 1.04 1.04 4.16 3.81 3.05 3.29 2.18
11.5 11.0 10.5 10.0 9.5 9.0 8.5 8.0 7.5 7.0 6.5 6.0 5.5 5.0 4.5 4.0 3.5 3.0 2.5 2.0 1.5 1.0 0.5 0.0 -0.5 -1.0
f1 (ppm)

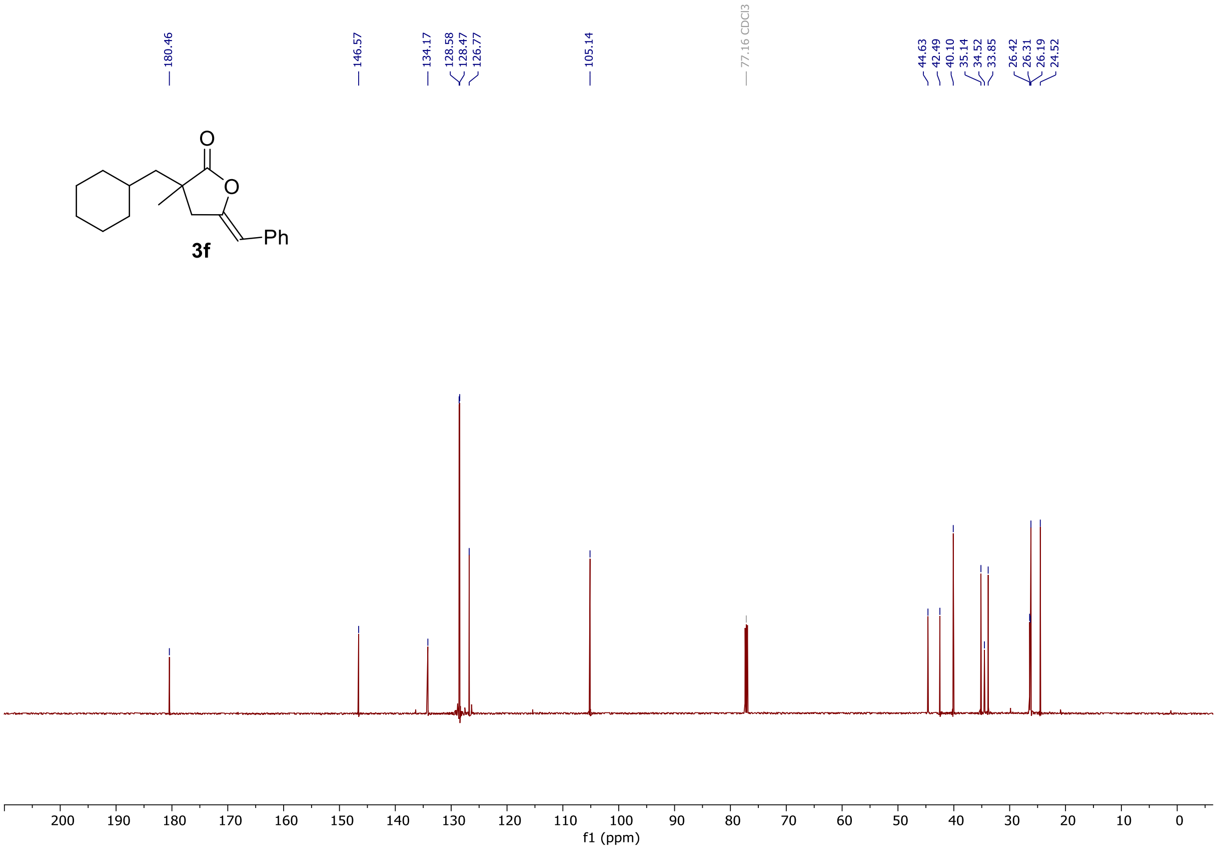
180.46 146.57 134.17 128.58 128.47 126.77 105.14 77.16 CDCl3 44.63 42.49 40.10 35.14 34.52 33.85 26.42 26.31 26.19 24.52
O
O
Ph
3f
200 190 180 170 160 150 140 130 120 110 100 90 80 70 60 50 40 30 20 10 0
f1 (ppm)

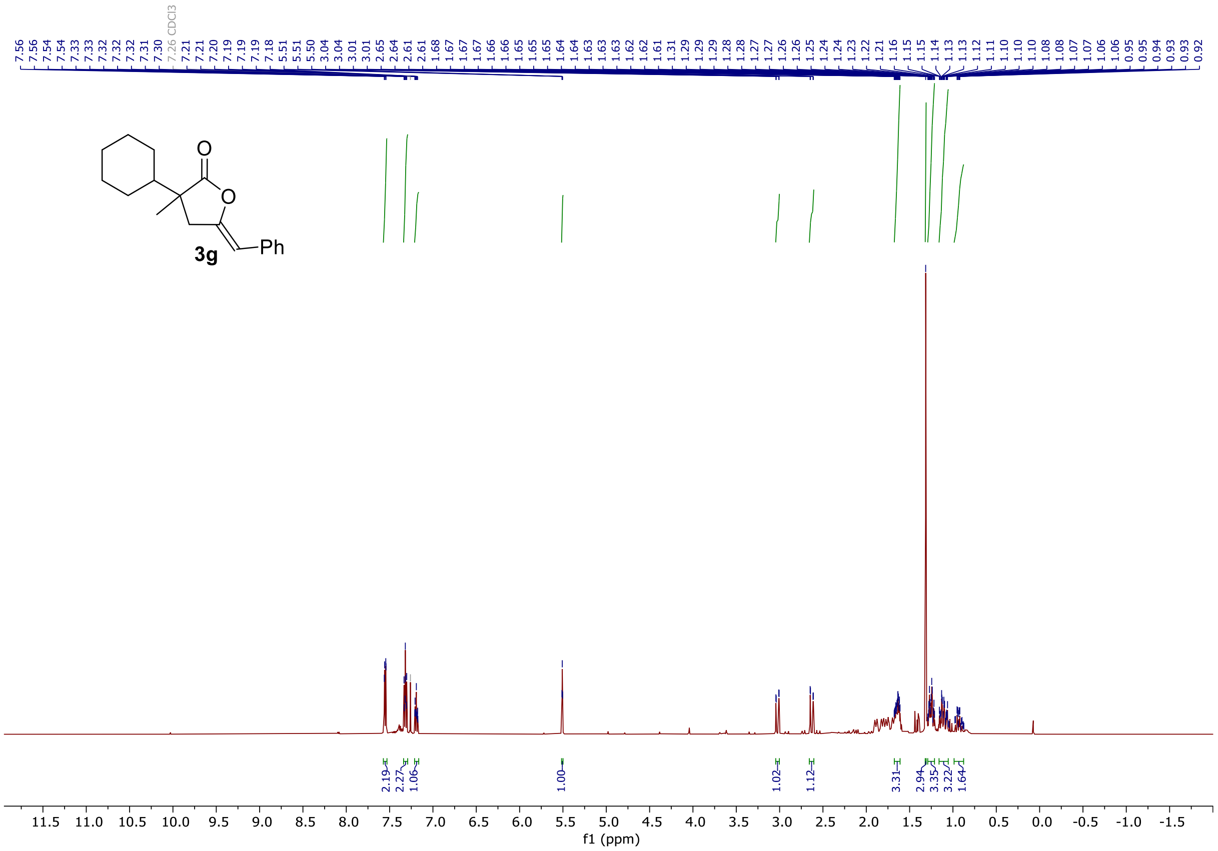
7.56 7.56 7.54 7.54 7.33 7.33 7.32 7.32 7.32 7.31 7.30 7.26 CDCl3 7.21 7.21 7.20 7.19 7.19 7.19 7.18 5.51 5.51 5.50 3.04 3.04 3.01 3.01 2.65 2.64 2.61 2.61 1.68 1.67 1.67 1.67 1.66 1.66 1.65 1.65 1.65 1.64 1.64 1.63 1.63 1.63 1.62 1.61 1.31 1.29 1.29 1.28 1.28 1.27 1.26 1.26 1.25 1.24 1.24 1.23 1.22 1.21 1.16 1.15 1.15 1.14 1.13 1.13 1.12 1.11 1.10 1.10 1.10 1.08 1.08 1.07 1.07 1.06 1.06 0.95 0.95 0.94 0.93 0.93 0.92
O
O
3g
Ph
2.19
2.27
1.06
1.00
1.02
1.12
3.31
2.94
3.35
3.22
1.64
11.5 11.0 10.5 10.0 9.5 9.0 8.5 8.0 7.5 7.0 6.5 6.0 5.5 5.0 4.5 4.0 3.5 3.0 2.5 2.0 1.5 1.0 0.5 0.0 -0.5 -1.0 -1.5
f1 (ppm)

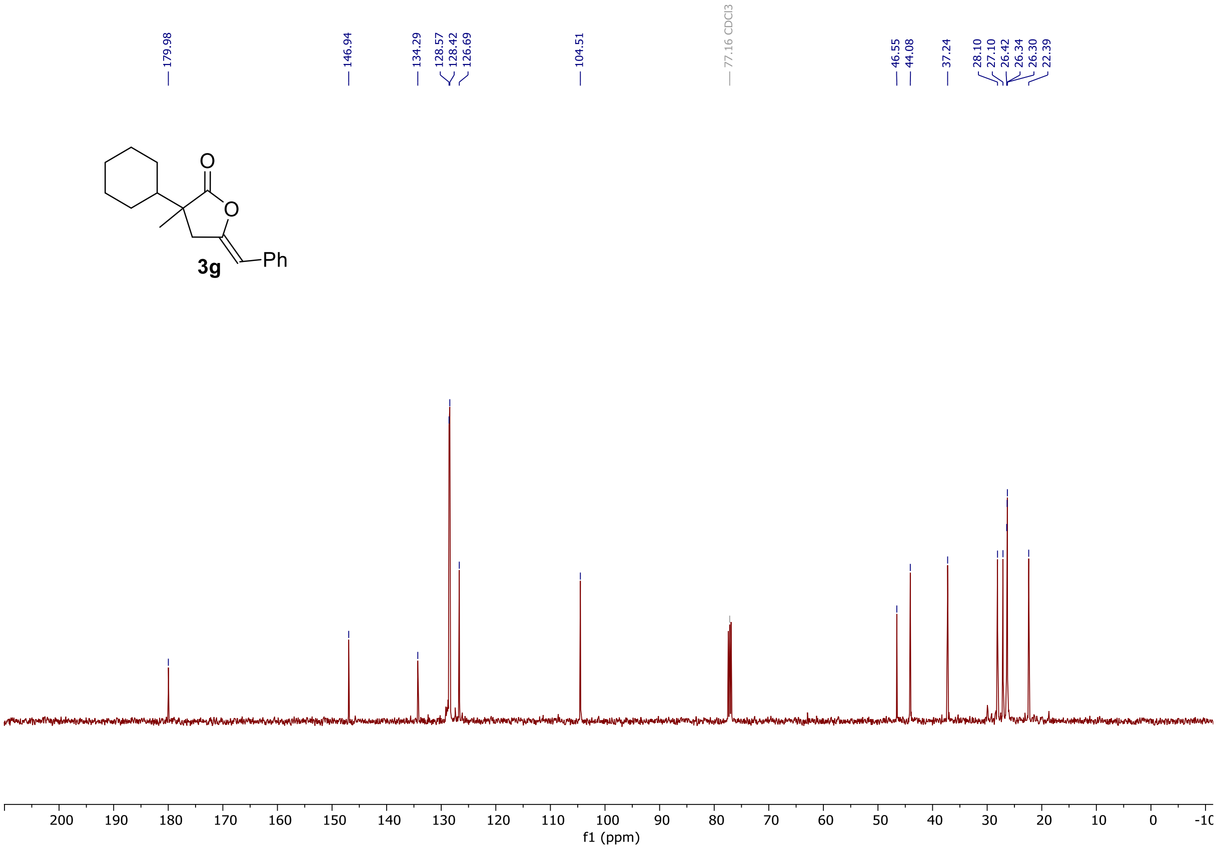
179.98
146.94
134.29 128.57 128.42 126.69
104.51
77.16 CDCl3
46.55 44.08
37.24
28.10 27.10 26.42 26.34 26.30 22.39
O
O
3g
Ph
200 190 180 170 160 150 140 130 120 110 100 90 80 70 60 50 40 30 20 10 0 -10
f1 (ppm)

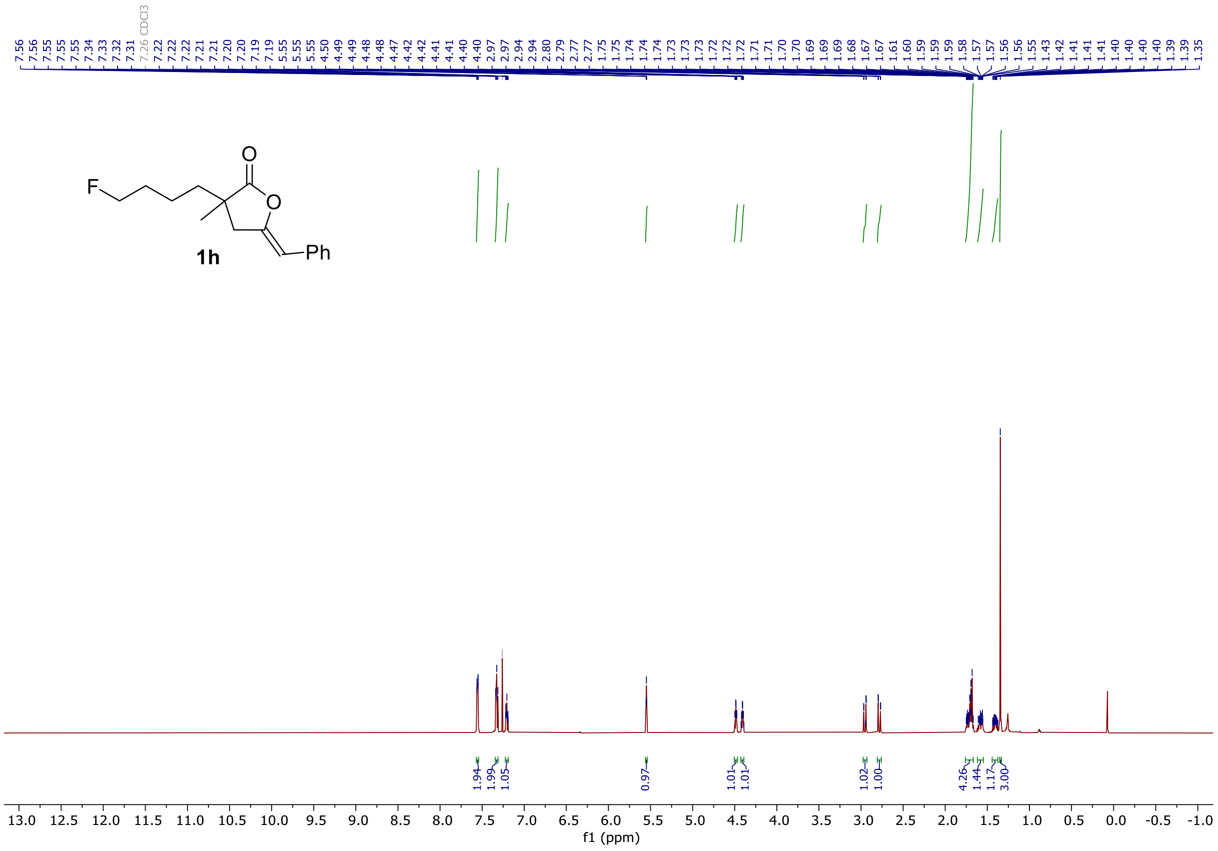
7.56 7.56 7.55 7.55 7.55 7.34 7.33 7.32 7.31 7.26 CDCl3 7.22 7.22 7.22 7.21 7.21 7.20 7.20 7.19 7.19 5.55 5.55 5.55 5.55 4.50 4.49 4.49 4.48 4.48 4.47 4.42 4.42 4.41 4.41 4.40 4.40 2.97 2.97 2.94 2.94 2.80 2.79 2.77 2.77 1.75 1.75 1.74 1.74 1.74 1.73 1.73 1.73 1.72 1.72 1.72 1.71 1.70 1.70 1.69 1.69 1.69 1.68 1.67 1.67 1.61 1.60 1.59 1.59 1.59 1.58 1.57 1.57 1.57 1.56 1.55 1.43 1.42 1.41 1.41 1.40 1.40 1.40 1.40 1.39 1.39 1.35
F
O
O
Ph
1h
1.94 1.99 1.05 0.97 1.01 1.01 1.02 1.00 4.26 1.44 1.17 3.00
13.0 12.5 12.0 11.5 11.0 10.5 10.0 9.5 9.0 8.5 8.0 7.5 7.0 6.5 6.0 5.5 5.0 4.5 4.0 3.5 3.0 2.5 2.0 1.5 1.0 0.5 0.0 -0.5 -1.0
f1 (ppm)

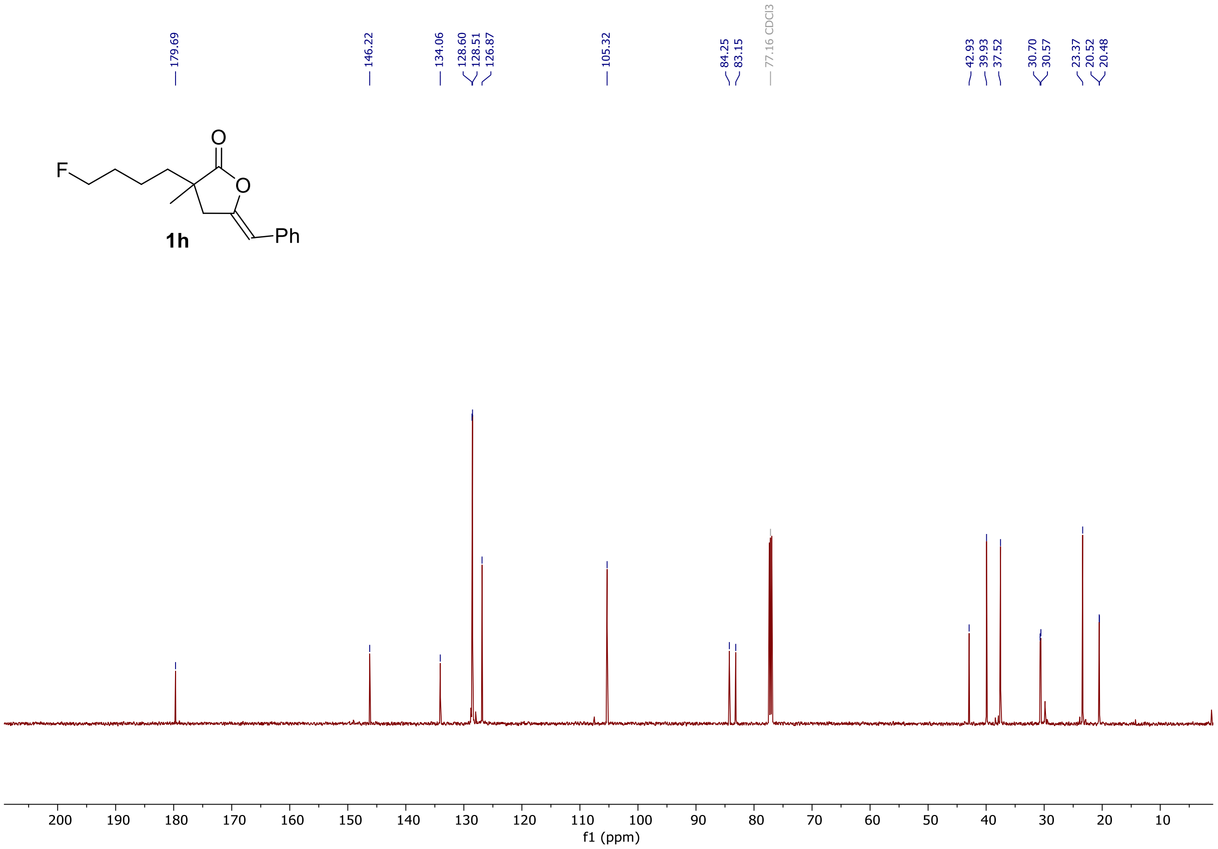
179.69 146.22 134.06 128.60 128.51 126.87 105.32 84.25 83.15 77.16 CDCl3 42.93 39.93 37.52 30.70 30.57 23.37 20.52 20.48
F
O
O
Ph
1h
200 190 180 170 160 150 140 130 120 110 100 90 80 70 60 50 40 30 20 10
f1 (ppm)

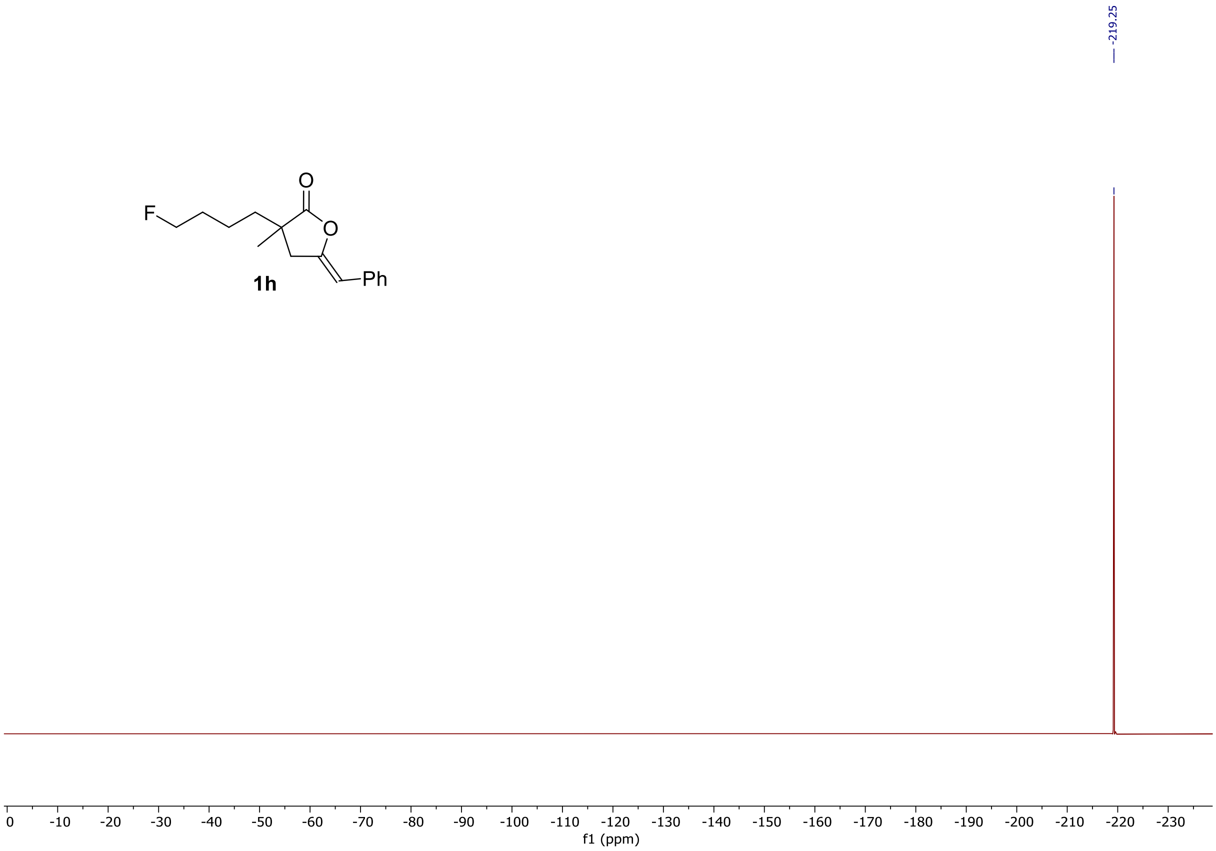
F
O
O
Ph
1h
-219.25
0 -10 -20 -30 -40 -50 -60 -70 -80 -90 -100 -110 -120 -130 -140 -150 -160 -170 -180 -190 -200 -210 -220 -230
f1 (ppm)

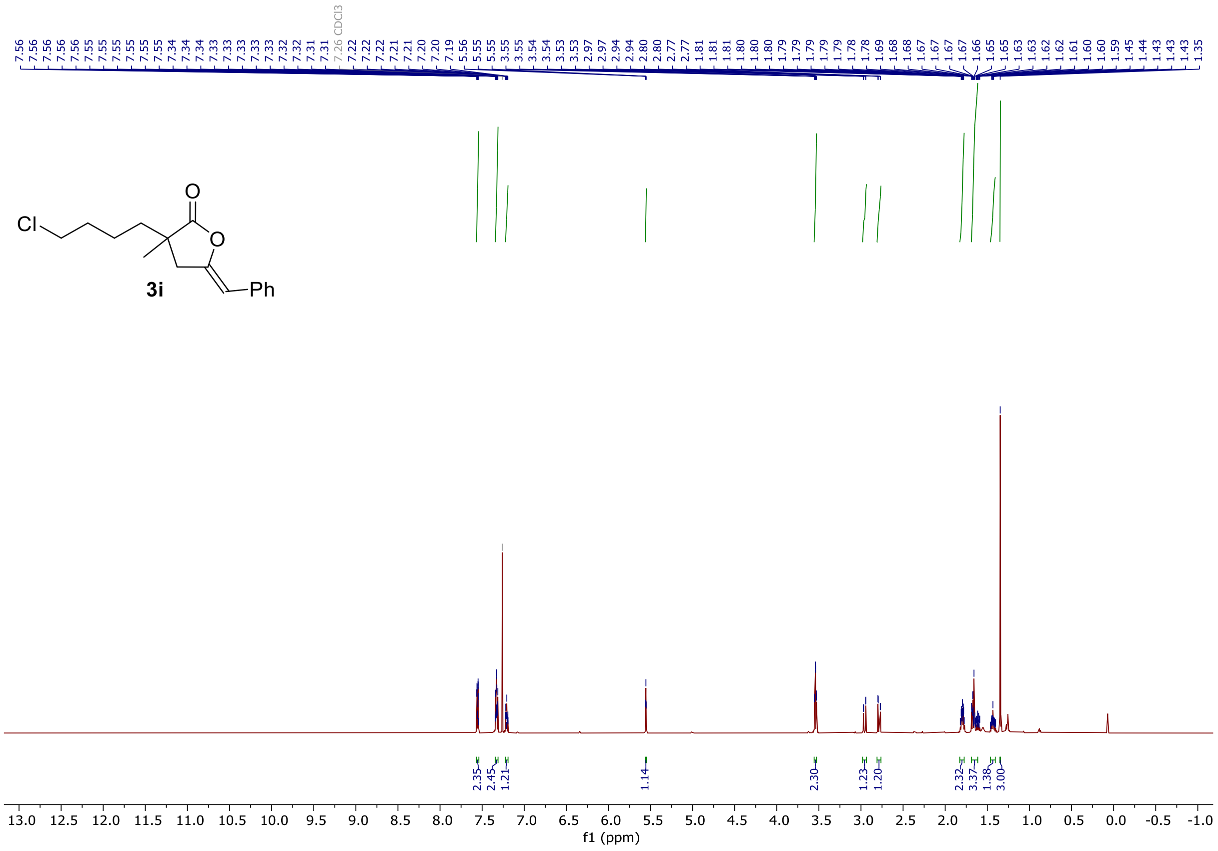

7.56 7.56 7.56 7.56 7.55 7.55 7.55 7.55 7.55 7.55 7.34 7.34 7.34 7.33 7.33 7.33 7.33 7.32 7.32 7.31 7.26 CDCl3 7.22 7.22 7.22 7.21 7.21 7.20 7.20 7.19 5.56 5.55 5.55 3.55 3.55 3.54 3.54 3.53 3.53 2.97 2.97 2.94 2.94 2.80 2.80 2.77 1.81 1.81 1.80 1.80 1.80 1.79 1.79 1.79 1.79 1.79 1.78 1.78 1.69 1.68 1.68 1.67 1.67 1.67 1.66 1.65 1.65 1.63 1.63 1.62 1.62 1.61 1.60 1.60 1.59 1.45 1.44 1.43 1.43 1.43 1.35
Cl
O
O
3i
Ph
2.35
2.45
1.21
1.14
2.30
1.23
1.20
2.32
3.37
1.38
3.00
13.0 12.5 12.0 11.5 11.0 10.5 10.0 9.5 9.0 8.5 8.0 7.5 7.0 6.5 6.0 5.5 5.0 4.5 4.0 3.5 3.0 2.5 2.0 1.5 1.0 0.5 0.0 -0.5 -1.0
f1 (ppm)

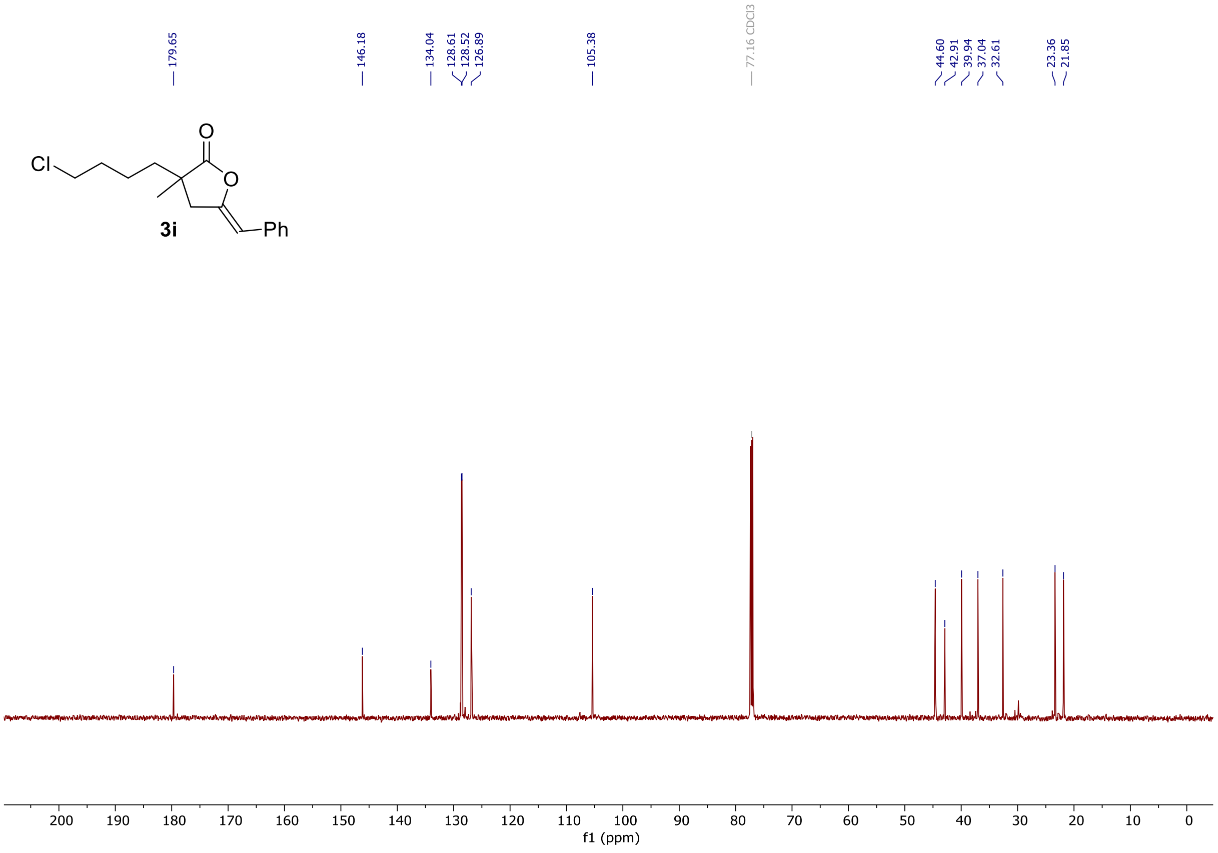

179.65
146.18
134.04
128.61
128.52
126.89
105.38
77.16 CDCl3
44.60
42.91
39.94
37.04
32.61
23.36
21.85
Cl
O
O
3i
Ph
200 190 180 170 160 150 140 130 120 110 100 90 80 70 60 50 40 30 20 10 0
f1 (ppm)

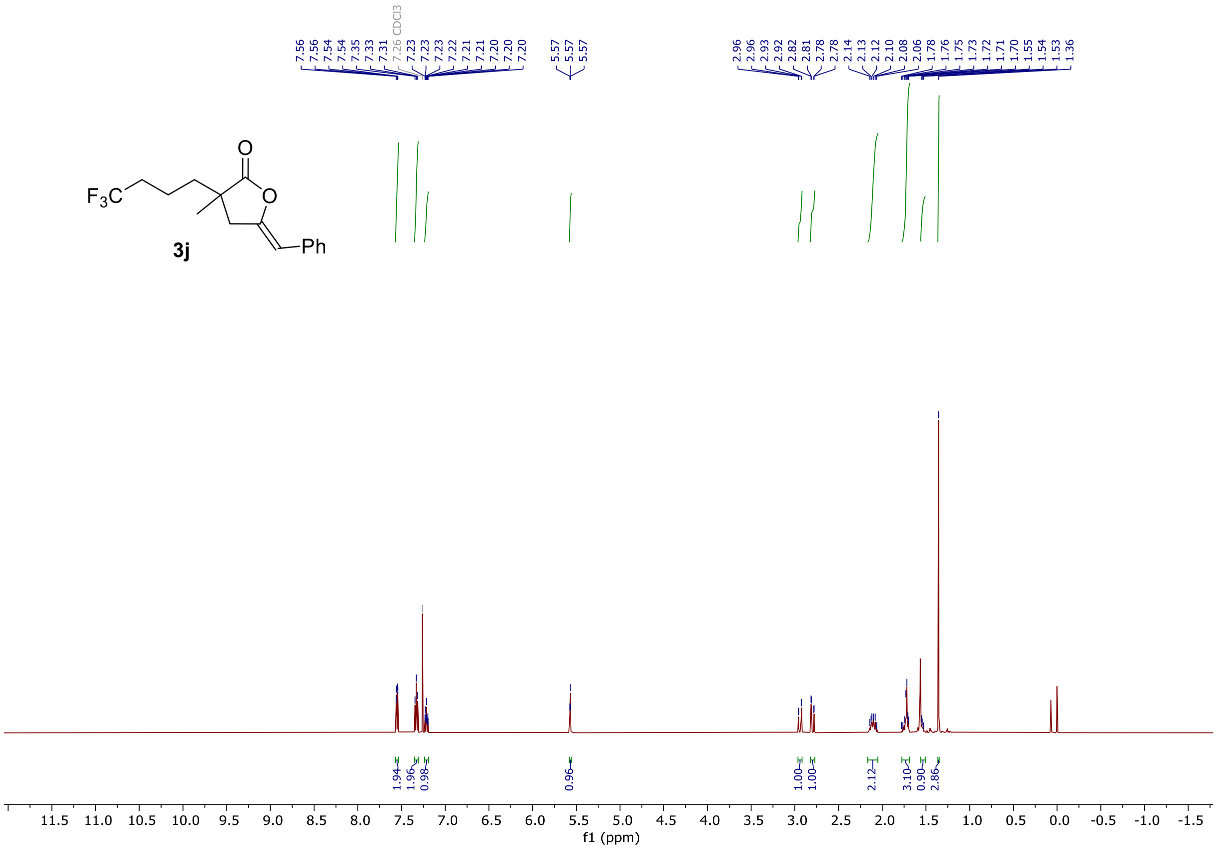
7.56 7.56 7.54 7.54 7.35 7.33 7.31 7.26 CDCl3 7.23 7.23 7.23 7.22 7.21 7.21 7.20 7.20 7.20
5.57 5.57 5.57
2.96 2.96 2.93 2.92 2.82 2.81 2.78 2.78 2.14 2.13 2.12 2.10 2.08 2.06 1.78 1.76 1.75 1.73 1.72 1.71 1.70 1.55 1.54 1.53 1.36
F_3C
O
3j
Ph
1.94 1.96 0.98 0.96 1.00 1.00 2.12 3.10 0.90 2.86
f1 (ppm)

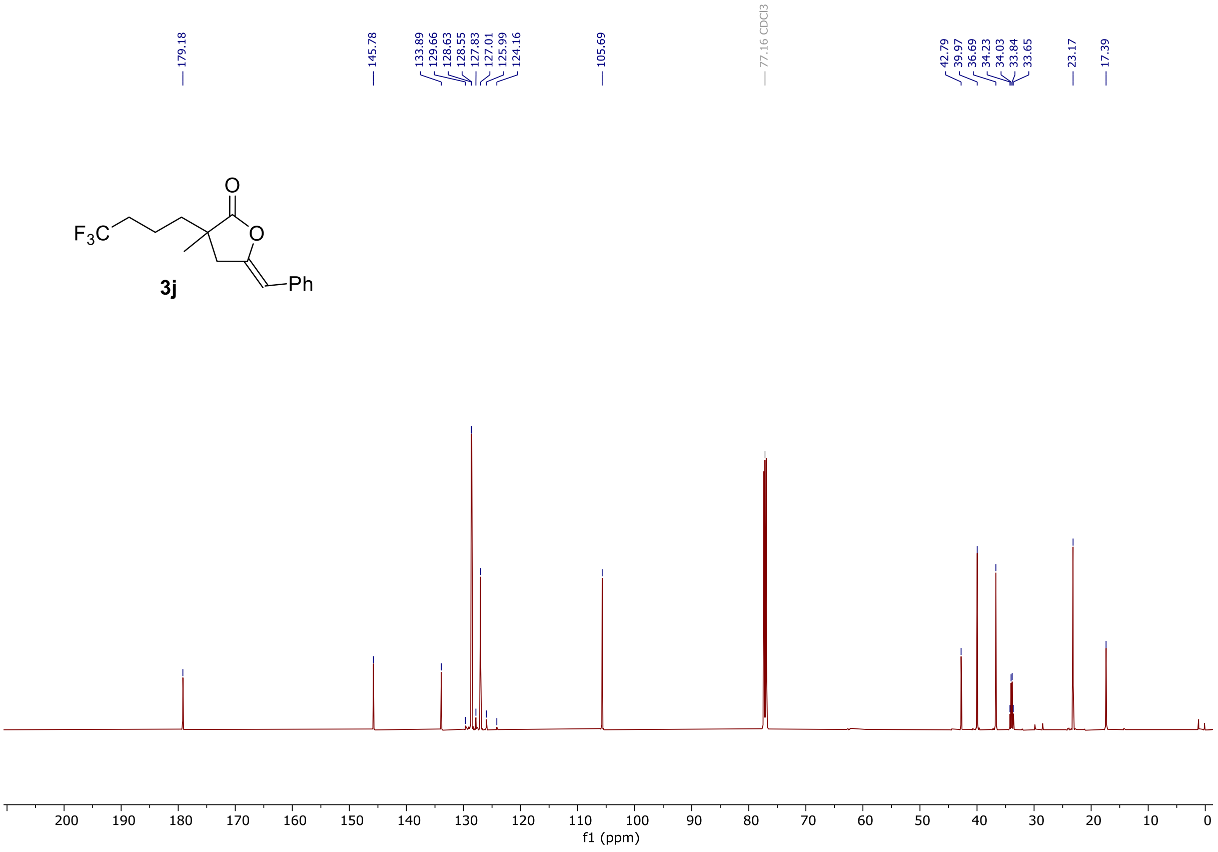
179.18
145.78
133.89 129.66 128.63 128.55 127.83 127.01 125.99 124.16
105.69
77.16 CDCl3
42.79 39.97 36.69 34.23 34.03 33.84 33.65
23.17
17.39
F_3C
O
3j
Ph
f1 (ppm)

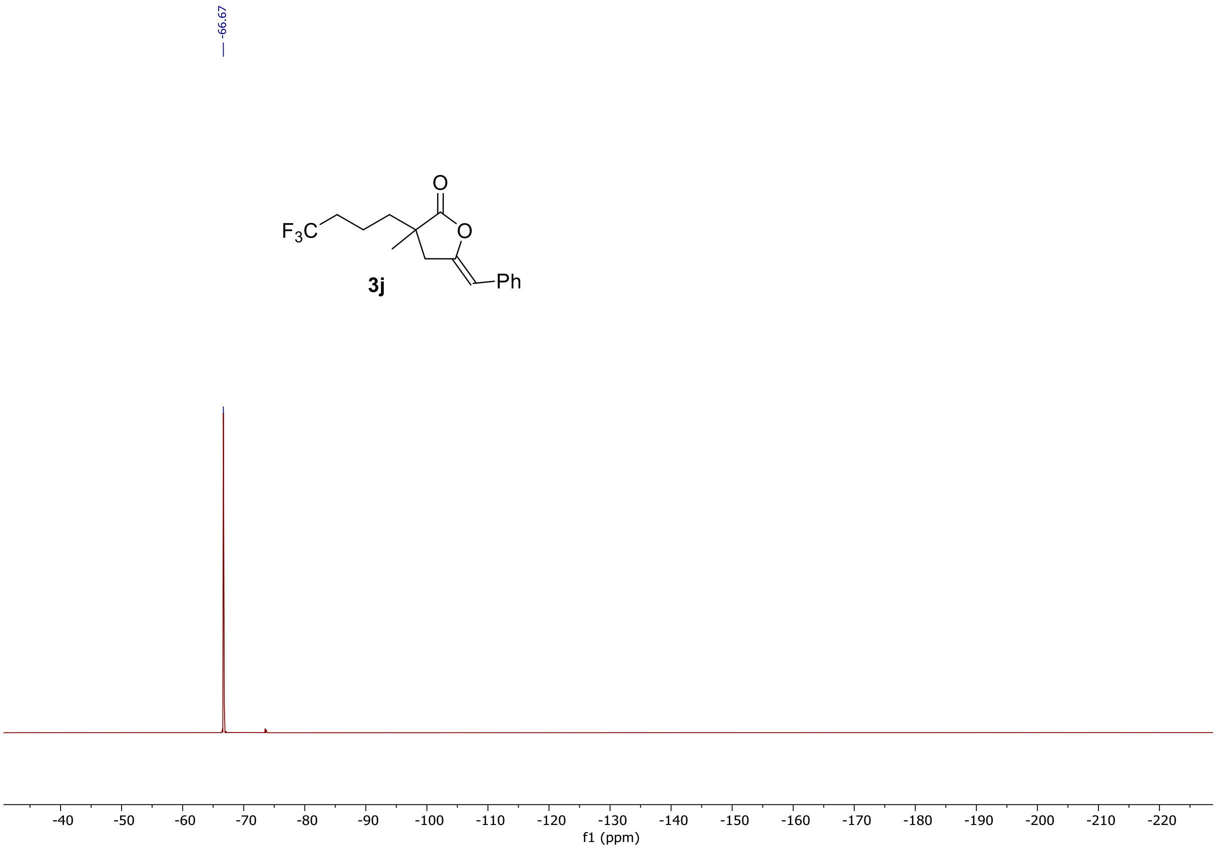
-66.67
O
F3C
O
Ph
3j
-40
-50
-60
-70
-80
-90
-100
-110
-120
-130
-140
-150
-160
-170
-180
-190
-200
-210
-220
f1 (ppm)

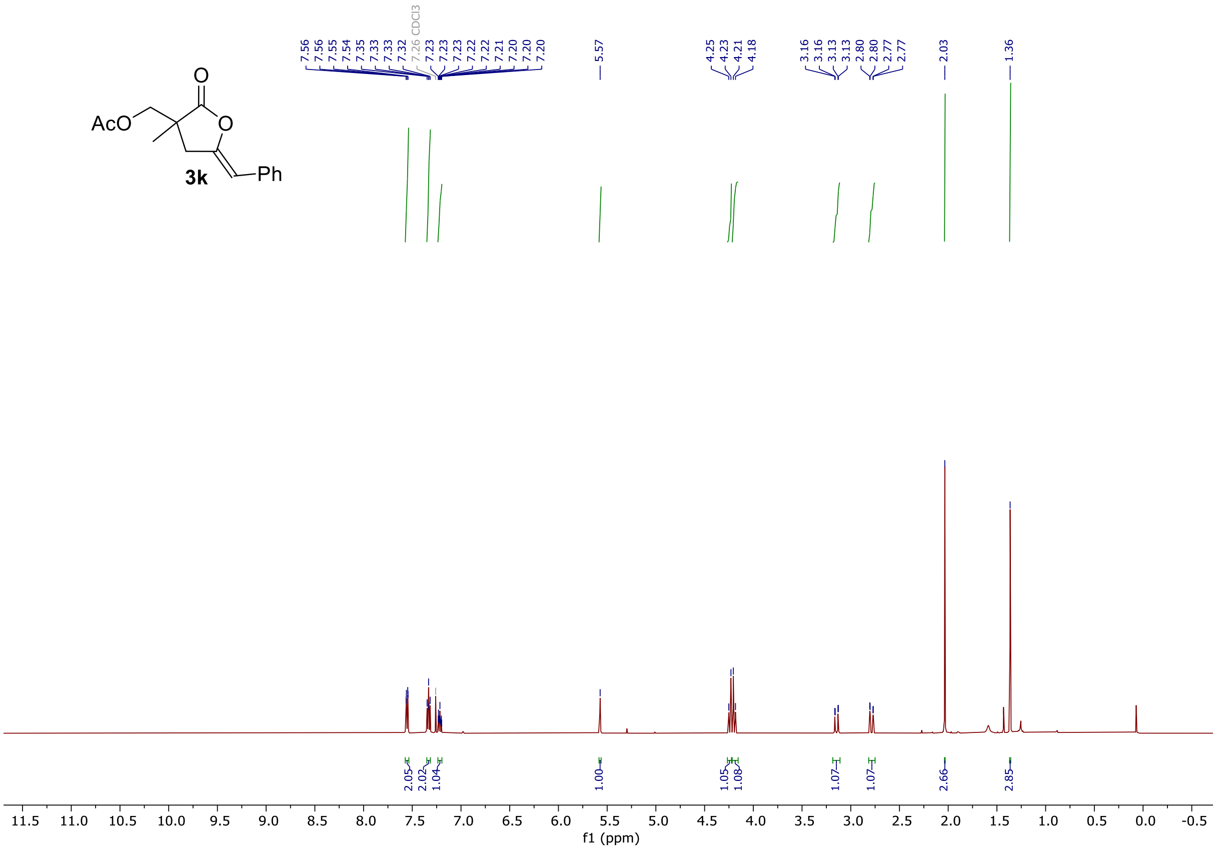
AcO
O
O
3k
Ph
7.56
7.56
7.55
7.54
7.35
7.33
7.33
7.32
7.26 CDCl3
7.23
7.23
7.23
7.22
7.22
7.21
7.20
7.20
7.20
5.57
4.25
4.23
4.21
4.18
3.16
3.16
3.13
3.13
2.80
2.80
2.77
2.77
2.03
1.36
2.05
2.02
1.04
1.00
1.05
1.08
1.07
1.07
2.66
2.85
11.5 11.0 10.5 10.0 9.5 9.0 8.5 8.0 7.5 7.0 6.5 6.0 5.5 5.0 4.5 4.0 3.5 3.0 2.5 2.0 1.5 1.0 0.5 0.0 -0.5
f1 (ppm)

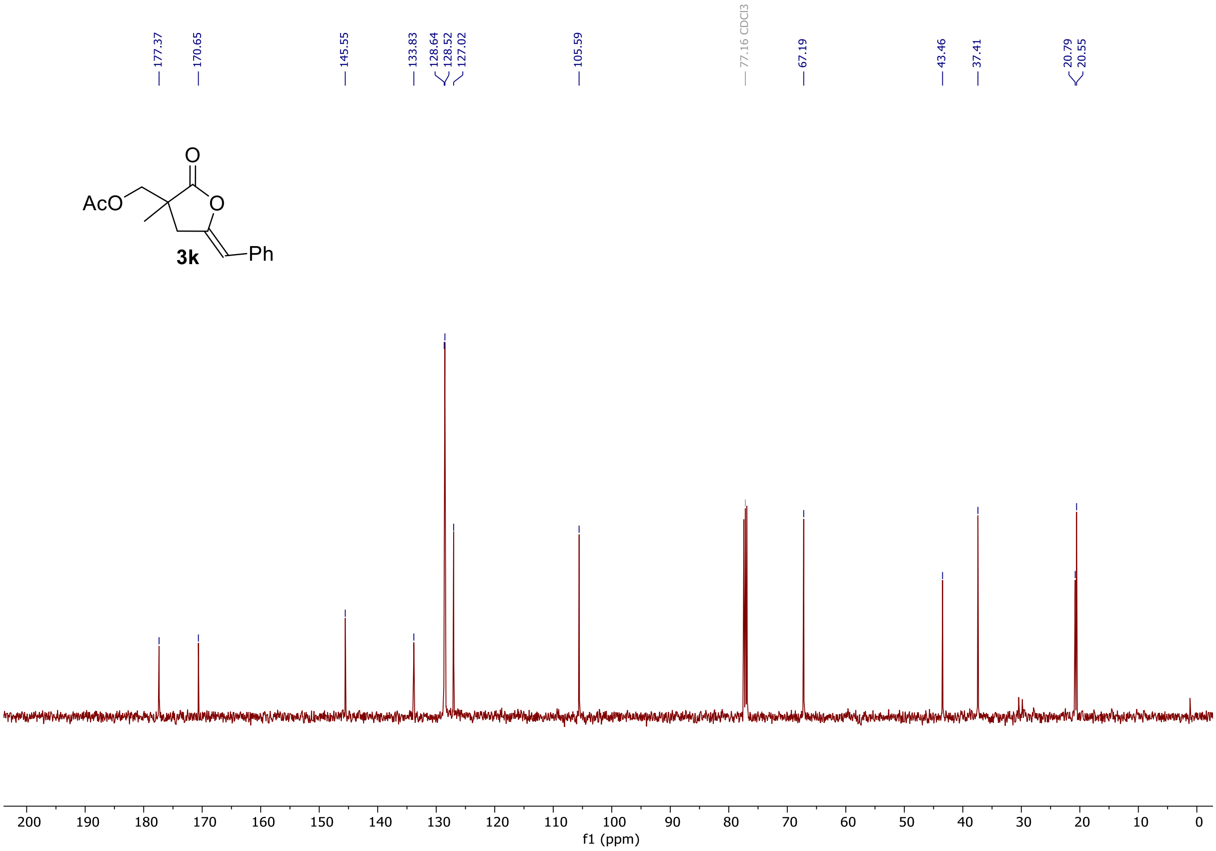
177.37
170.65
145.55
133.83
128.64
128.52
127.02
105.59
77.16 CDCl3
67.19
43.46
37.41
20.79
20.55
AcO
O
O
3k
Ph
200 190 180 170 160 150 140 130 120 110 100 90 80 70 60 50 40 30 20 10 0
f1 (ppm)

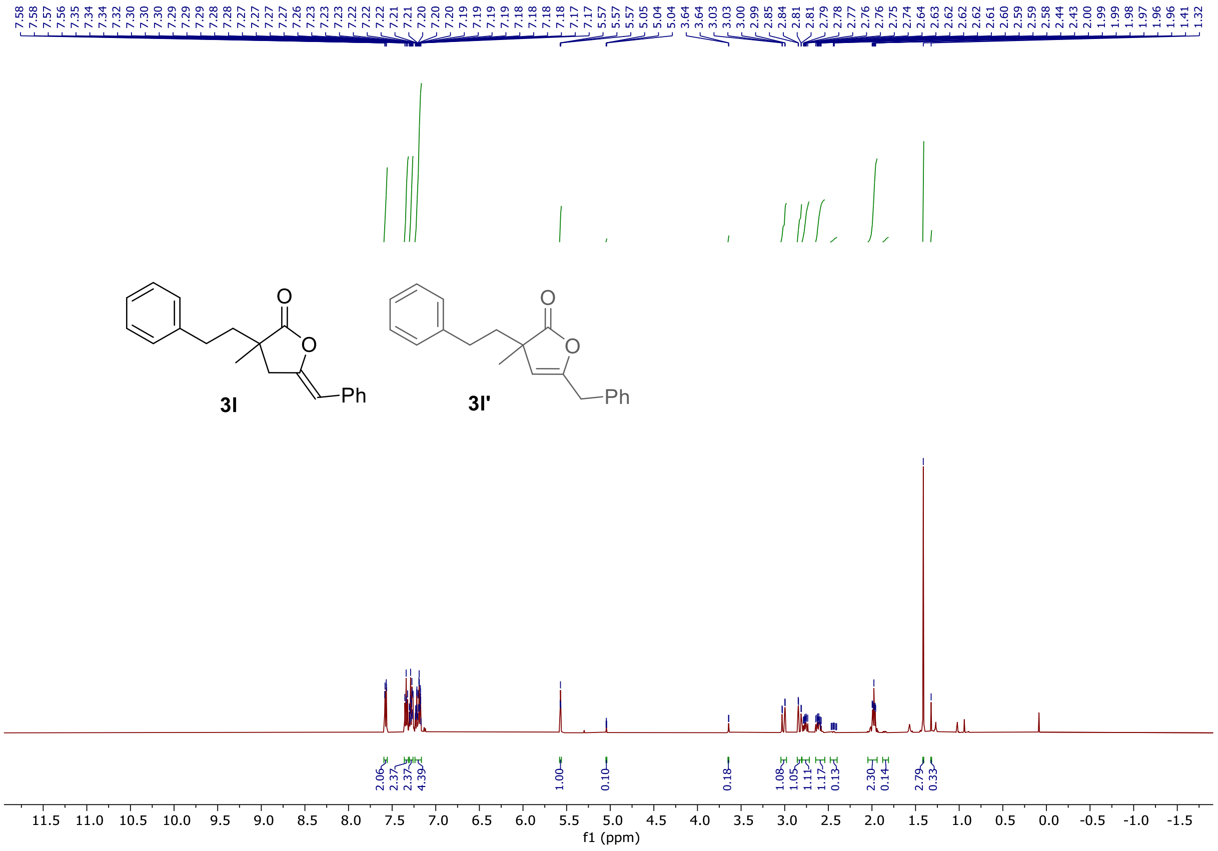
7.58 7.58 7.57 7.56 7.35 7.34 7.34 7.32 7.30 7.30 7.30 7.29 7.29 7.29 7.28 7.28 7.27 7.27 7.27 7.27 7.26 7.23 7.23 7.23 7.22 7.22 7.22 7.22 7.21 7.21 7.20 7.20 7.20 7.19 7.19 7.19 7.19 7.18 7.18 7.18 7.18 7.17 7.17 5.57 5.57 5.57 5.05 5.04 5.04 3.64 3.64 3.03 3.03 3.00 2.99 2.85 2.84 2.81 2.81 2.79 2.78 2.77 2.76 2.76 2.75 2.74 2.64 2.63 2.62 2.62 2.61 2.60 2.59 2.59 2.58 2.44 2.43 2.00 1.99 1.99 1.98 1.97 1.96 1.41 1.32
O
O
Ph
3l
O
O
Ph
3l'
2.06 2.37 2.37 4.39 1.00 0.10 0.18 1.08 1.05 1.11 1.17 0.13 2.30 0.14 2.79 0.33
11.5 11.0 10.5 10.0 9.5 9.0 8.5 8.0 7.5 7.0 6.5 6.0 5.5 5.0 4.5 4.0 3.5 3.0 2.5 2.0 1.5 1.0 0.5 0.0 -0.5 -1.0 -1.5
f1 (ppm)

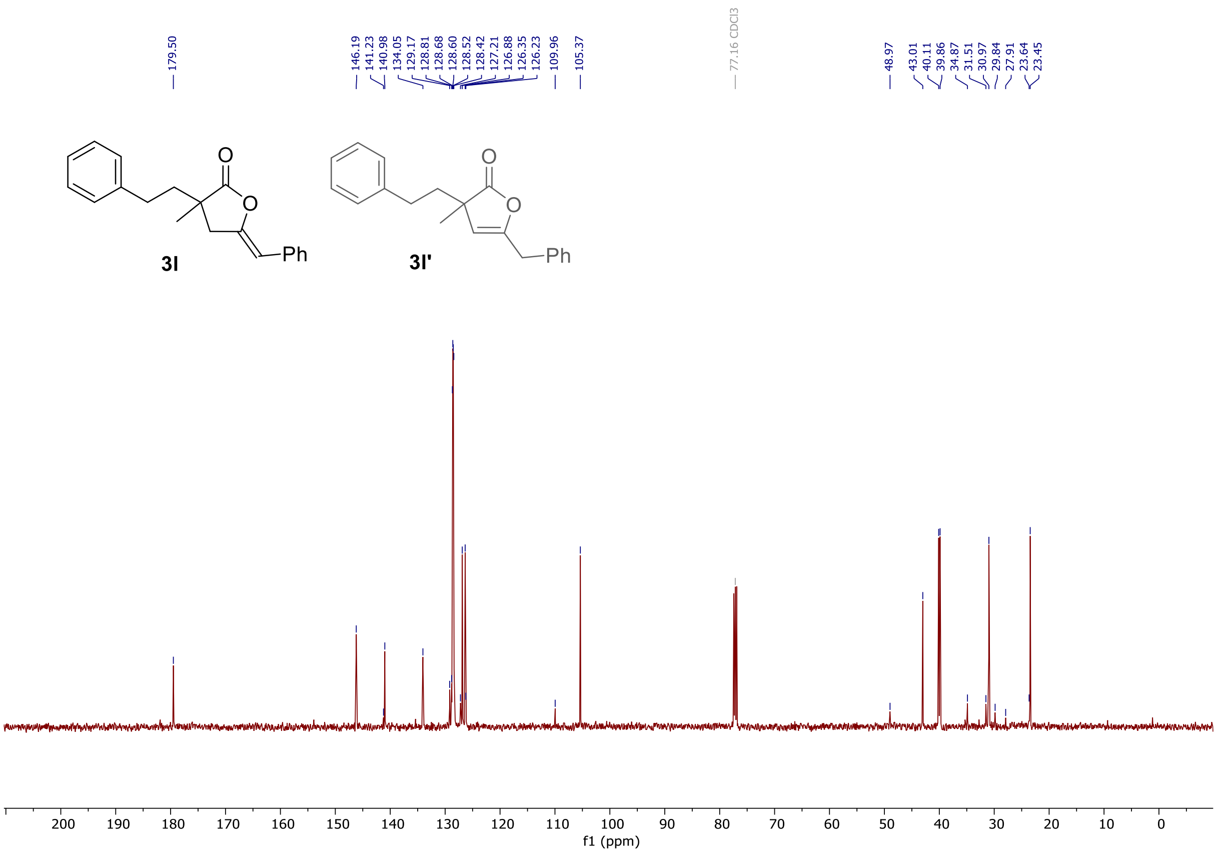
179.50
146.19 141.23 140.98 134.05 129.17 128.81 128.68 128.60 128.52 128.42 127.21 126.88 126.35 126.23 109.96 105.37
77.16 CDCl3
48.97 43.01 40.11 39.86 34.87 31.51 30.97 29.84 27.91 23.64 23.45
O
O
Ph
3l
O
O
Ph
3l'
200 190 180 170 160 150 140 130 120 110 100 90 80 70 60 50 40 30 20 10 0
f1 (ppm)

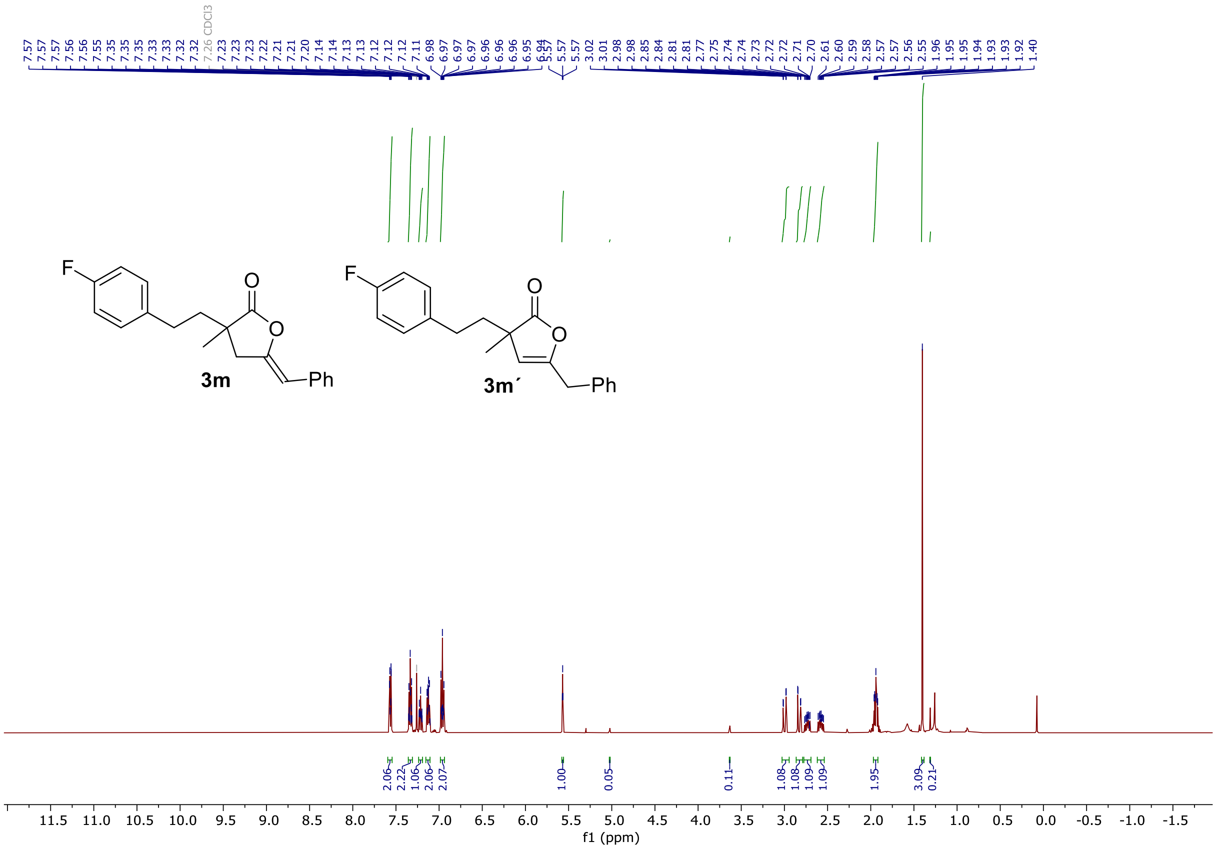
7.57 7.57 7.56 7.56 7.55 7.35 7.35 7.35 7.33 7.33 7.32 7.32 7.26 CDCl3 7.23 7.23 7.23 7.22 7.21 7.21 7.20 7.14 7.14 7.13 7.13 7.12 7.12 7.12 7.11 6.98 6.97 6.97 6.97 6.96 6.96 6.96 6.95 5.57 5.57 5.57 3.02 3.01 2.98 2.98 2.85 2.84 2.81 2.81 2.77 2.75 2.74 2.74 2.73 2.72 2.72 2.71 2.70 2.61 2.60 2.59 2.58 2.57 2.57 2.56 2.55 1.96 1.95 1.95 1.94 1.93 1.93 1.92 1.40
F
O
O
3m
Ph
F
O
O
3m´
Ph
2.06 2.22 1.06 2.06 2.07 1.00 0.05 0.11 1.08 1.08 1.09 1.09 1.95 3.09 0.21
11.5 11.0 10.5 10.0 9.5 9.0 8.5 8.0 7.5 7.0 6.5 6.0 5.5 5.0 4.5 4.0 3.5 3.0 2.5 2.0 1.5 1.0 0.5 0.0 -0.5 -1.0 -1.5
f1 (ppm)

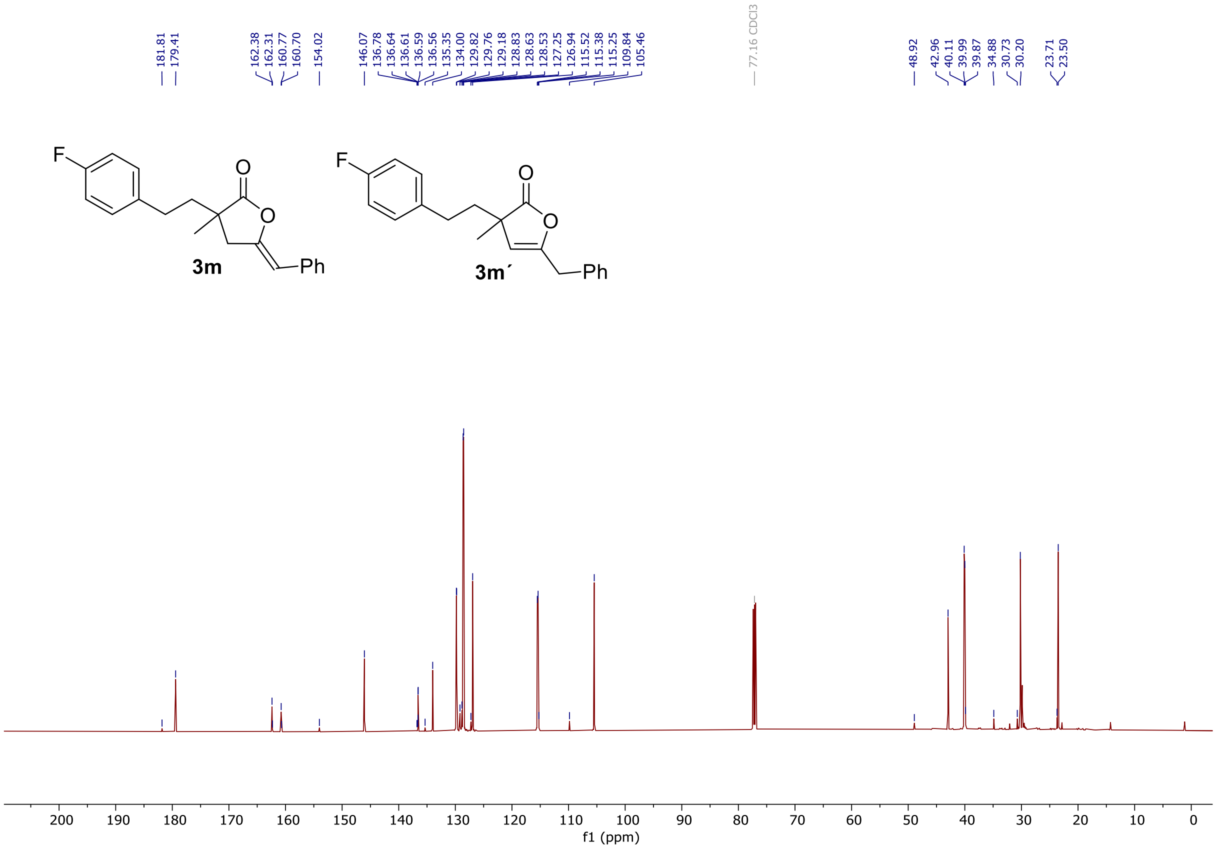
181.81 179.41 162.38 162.31 160.77 160.70 154.02 146.07 136.78 136.64 136.61 136.59 136.56 135.35 134.00 129.82 129.76 129.18 128.83 128.63 128.53 127.25 126.94 115.52 115.38 115.25 109.84 105.46 77.16 CDCl3 48.92 42.96 40.11 39.99 39.87 34.88 30.73 30.20 23.71 23.50
F
O
O
3m
Ph
F
O
O
3m´
Ph
200 190 180 170 160 150 140 130 120 110 100 90 80 70 60 50 40 30 20 10 0
f1 (ppm)

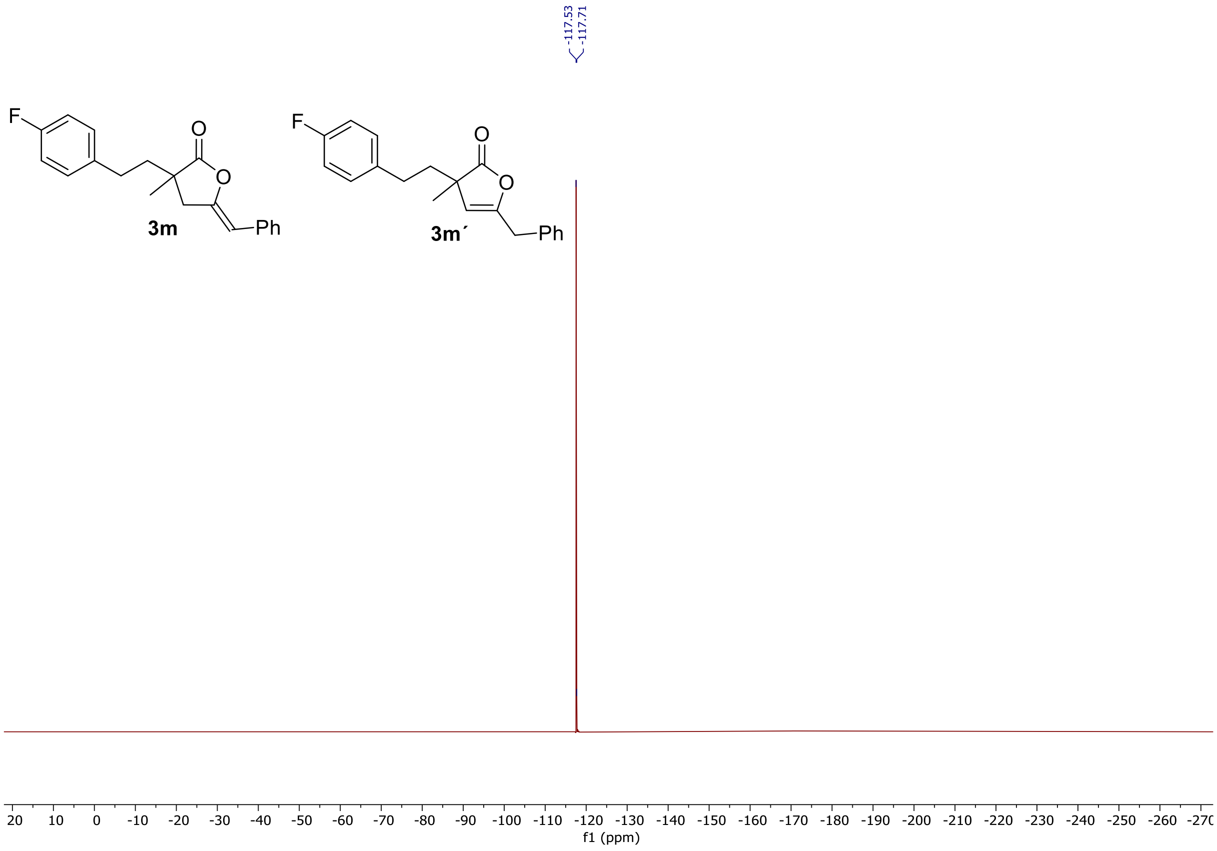
-117.53
-117.71
3m
3m´
f1 (ppm)

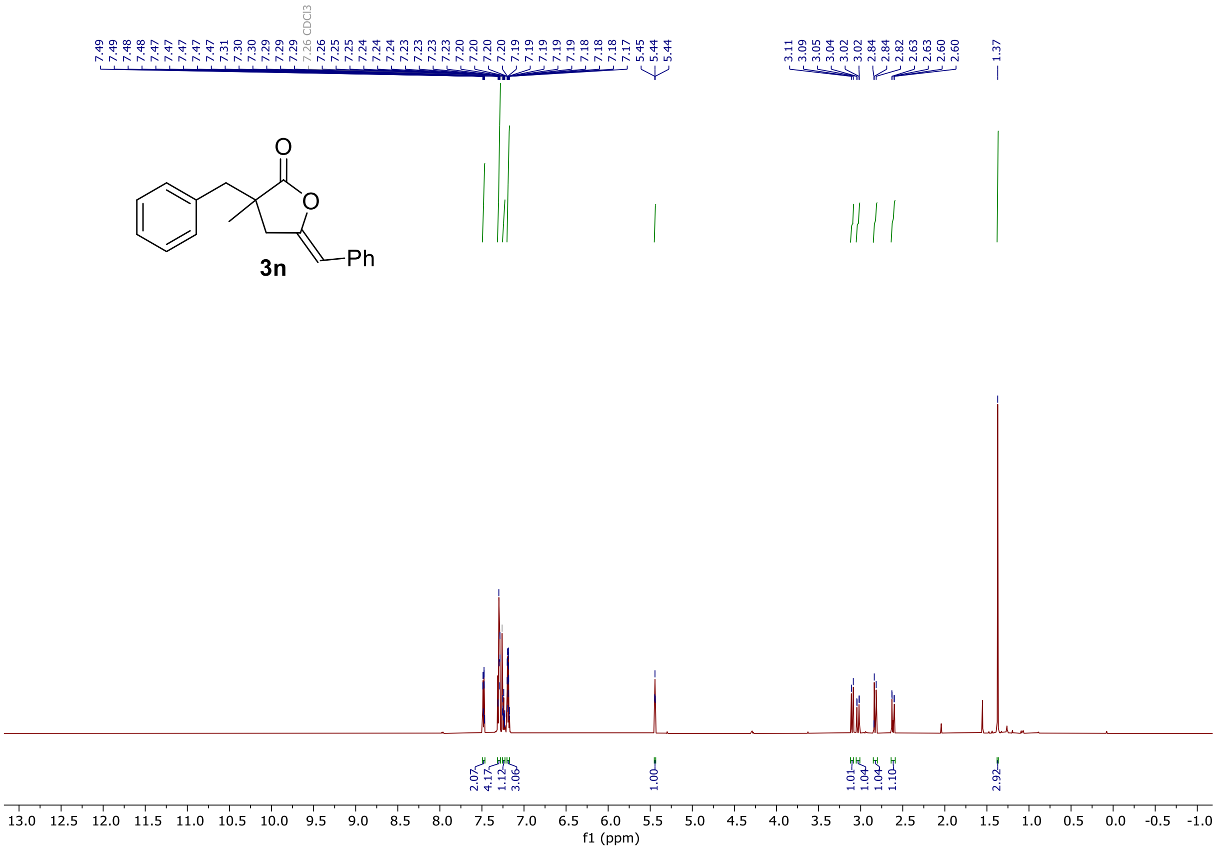
7.49
7.49
7.48
7.47
7.47
7.47
7.47
7.31
7.30
7.29
7.29
7.29
7.26 CDCl3
7.26
7.25
7.25
7.24
7.24
7.24
7.23
7.23
7.23
7.23
7.20
7.20
7.20
7.19
7.19
7.19
7.19
7.19
7.18
7.18
7.17
5.45
5.44
5.44
3.11
3.09
3.05
3.04
3.02
3.02
2.84
2.84
2.82
2.63
2.63
2.60
2.60
1.37
O
O
Ph
3n
2.07
4.17
1.12
3.06
1.00
1.01
1.04
1.04
1.10
2.92
f1 (ppm)

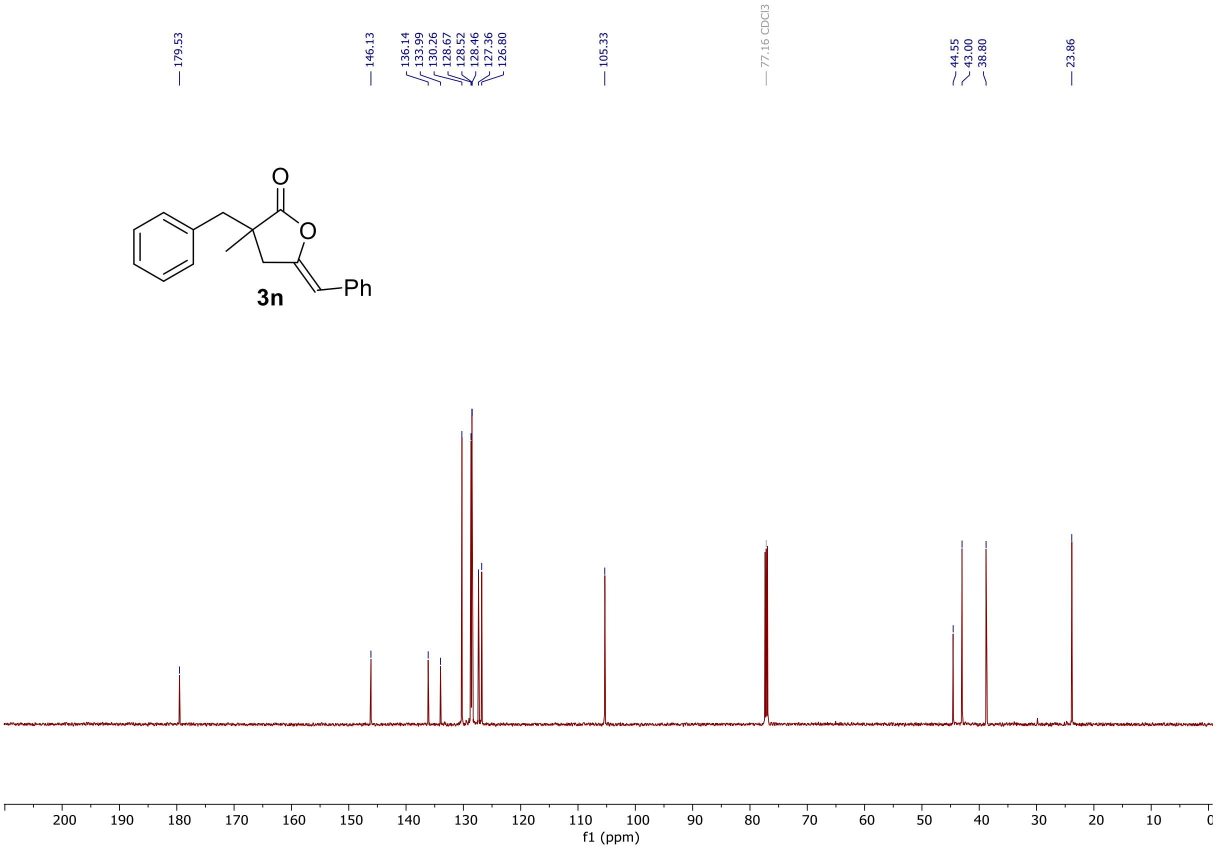
179.53
146.13
136.14
133.99
130.26
128.67
128.52
128.46
127.36
126.80
105.33
77.16 CDCl3
44.55
43.00
38.80
23.86
O
O
Ph
3n
f1 (ppm)

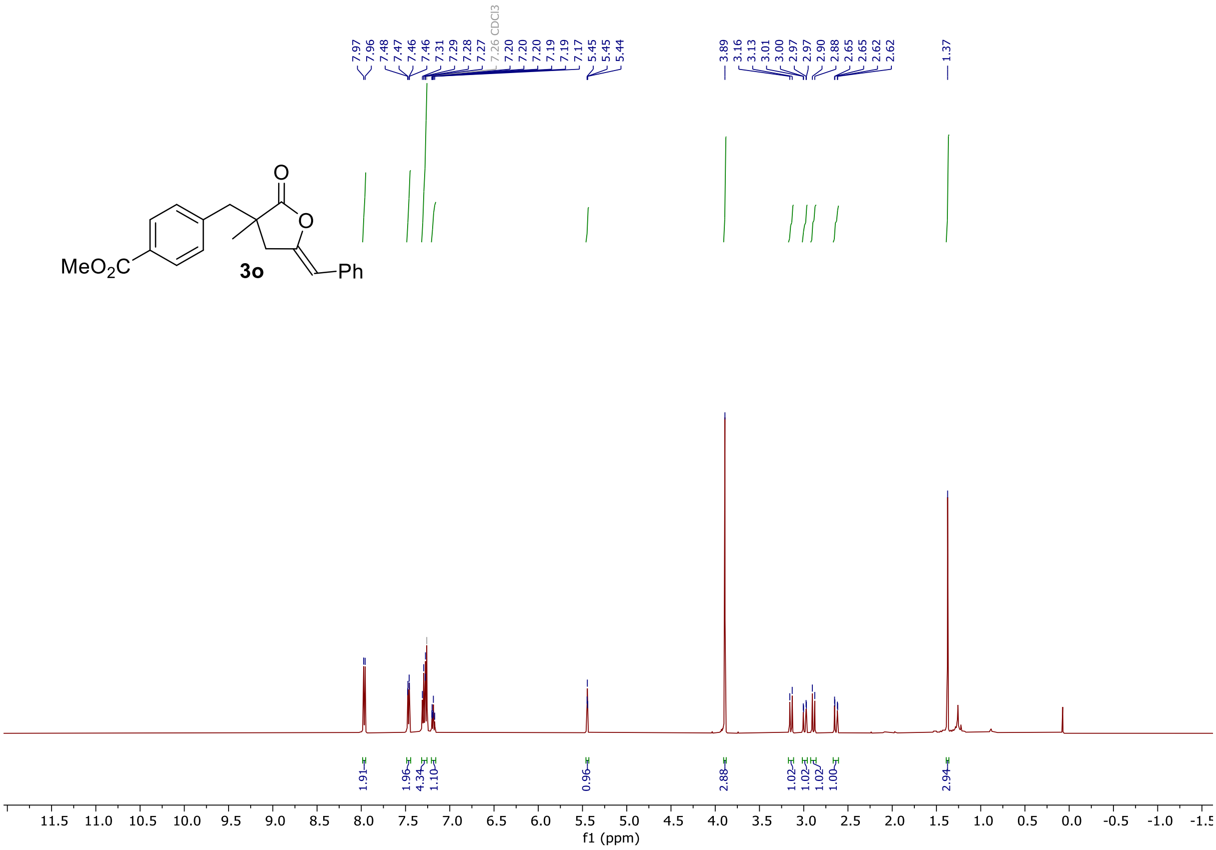
7.97
7.96
7.48
7.47
7.46
7.46
7.31
7.29
7.28
7.27
7.26 CDCl3
7.20
7.20
7.20
7.19
7.17
5.45
5.45
5.44
3.89
3.16
3.13
3.01
3.00
2.97
2.97
2.90
2.88
2.65
2.65
2.62
2.62
1.37
O
O
MeO2C
3o
Ph
1.91
1.96
4.34
1.10
0.96
2.88
1.02
1.02
1.02
1.00
2.94
f1 (ppm)

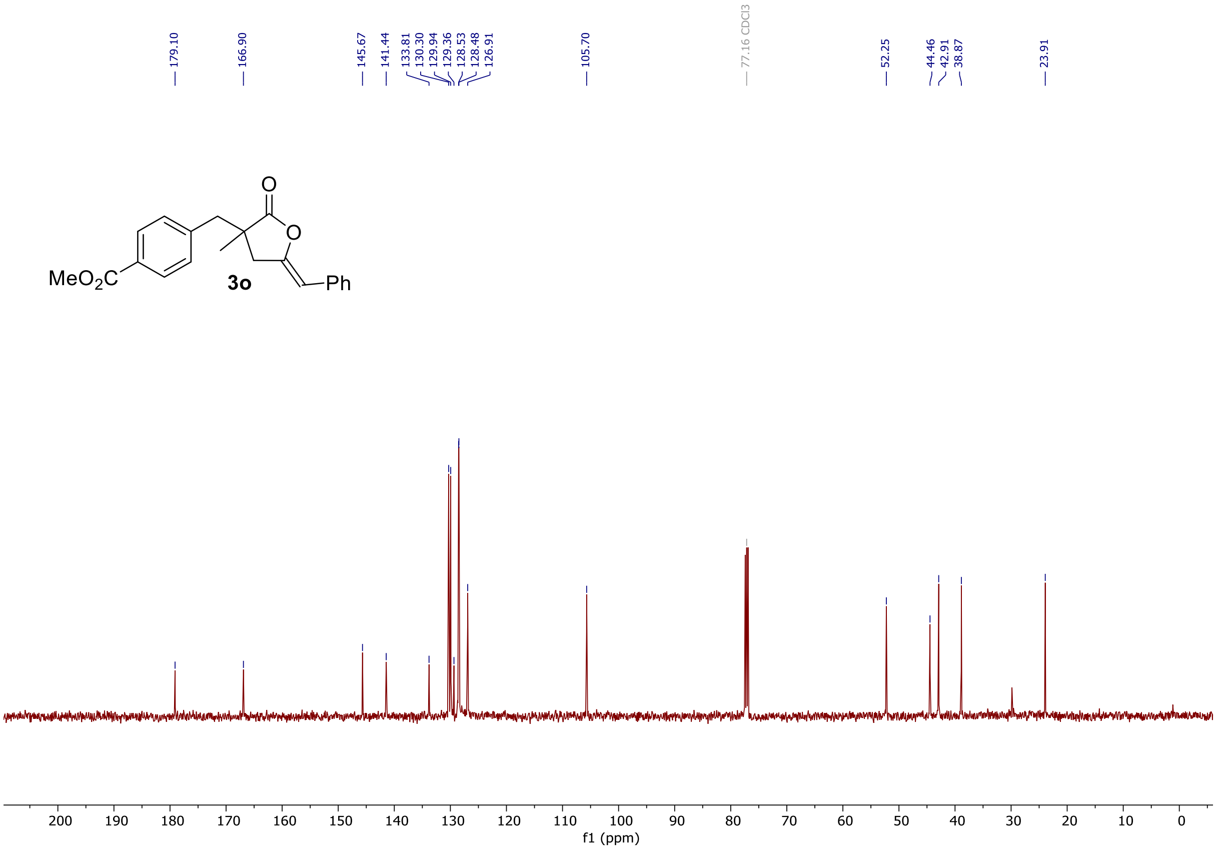
179.10
166.90
145.67
141.44
133.81
130.30
129.94
129.36
128.53
128.48
126.91
105.70
77.16 CDCl3
52.25
44.46
42.91
38.87
23.91
O
O
MeO2C
3o
Ph
f1 (ppm)

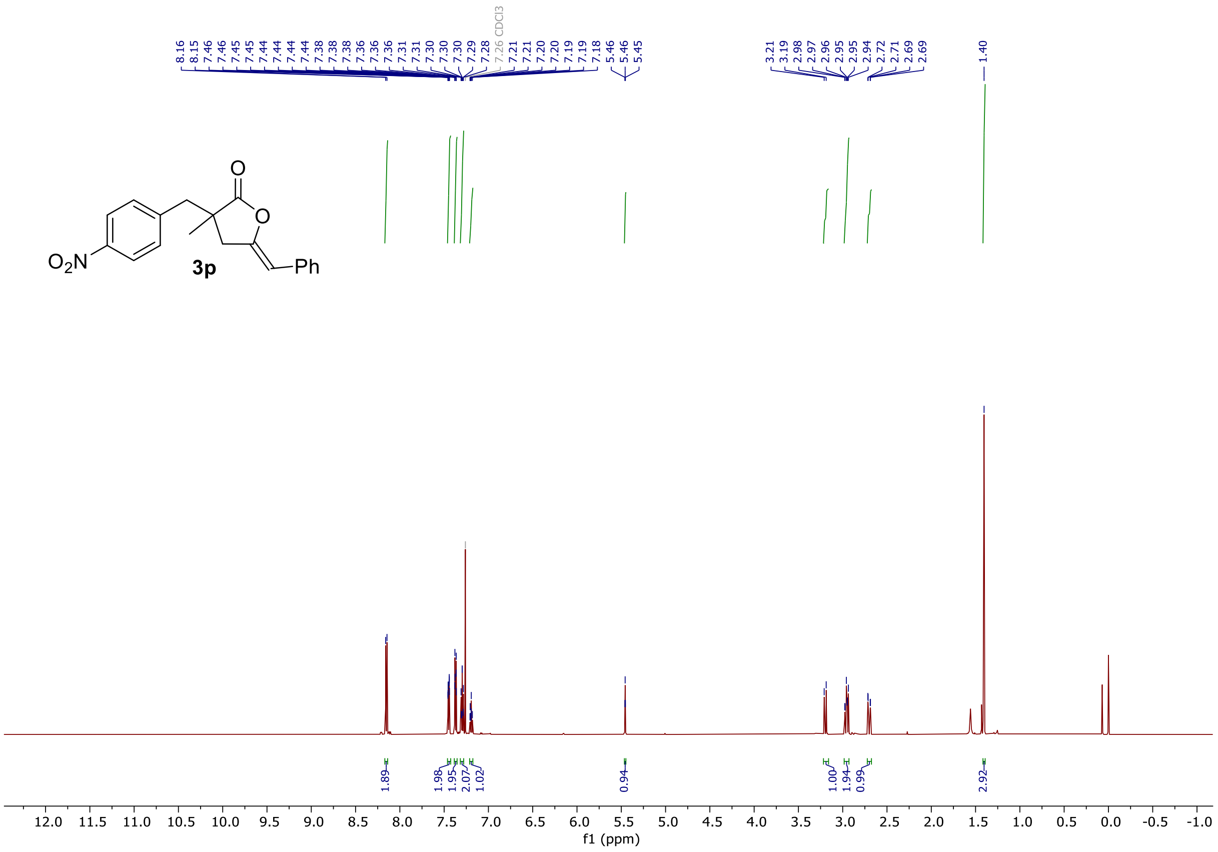
8.16
8.15
7.46
7.45
7.45
7.44
7.44
7.44
7.38
7.38
7.38
7.36
7.36
7.36
7.31
7.31
7.30
7.30
7.30
7.29
7.28
7.26 CDCl3
7.21
7.21
7.20
7.20
7.19
7.19
7.18
5.46
5.46
5.45
3.21
3.19
2.98
2.97
2.96
2.95
2.95
2.95
2.94
2.72
2.71
2.69
2.69
1.40
O
O
O_2N
3p
Ph
1.89
1.98
1.95
2.07
1.02
0.94
1.00
1.94
0.99
2.92
f1 (ppm)

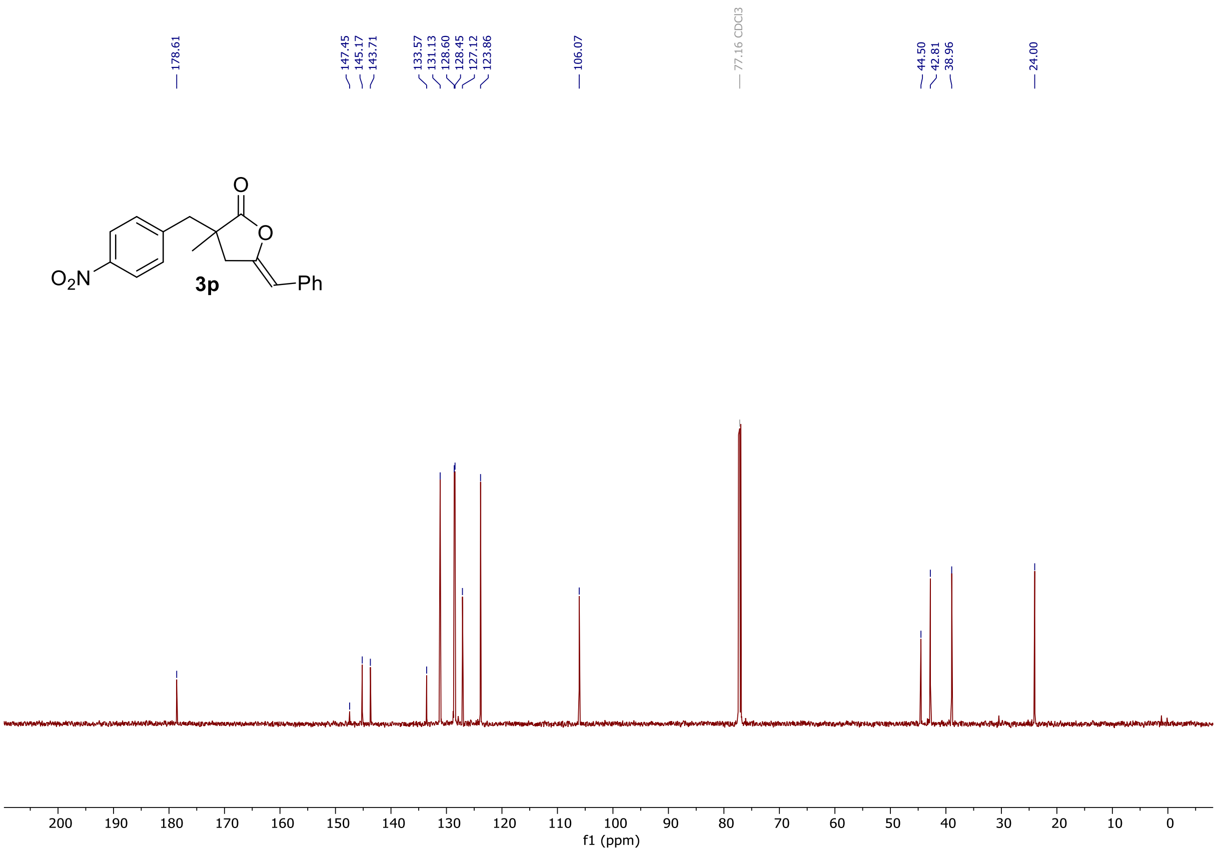
178.61
147.45
145.17
143.71
133.57
131.13
128.60
128.45
127.12
123.86
106.07
77.16 CDCl3
44.50
42.81
38.96
24.00
O
O
O_2N
3p
Ph
f1 (ppm)

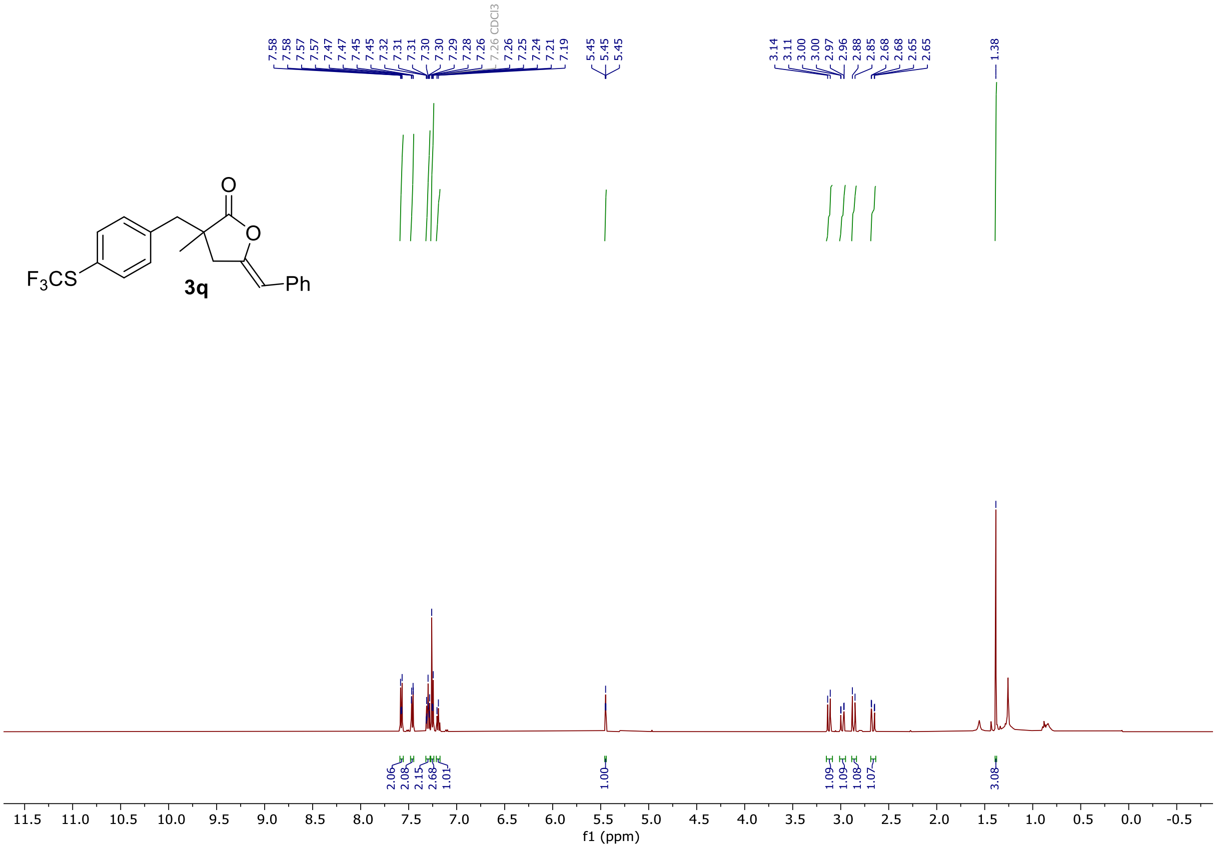
7.58
7.58
7.57
7.57
7.47
7.47
7.45
7.45
7.32
7.31
7.31
7.30
7.30
7.29
7.28
7.26 CDCl3
7.26
7.26
7.25
7.24
7.21
7.19
5.45
5.45
5.45
3.14
3.11
3.00
3.00
2.97
2.96
2.88
2.85
2.68
2.68
2.65
2.65
1.38
F3CS
3q
Ph
2.06
2.08
2.15
2.68
1.01
1.00
1.09
1.09
1.08
1.07
3.08
f1 (ppm)

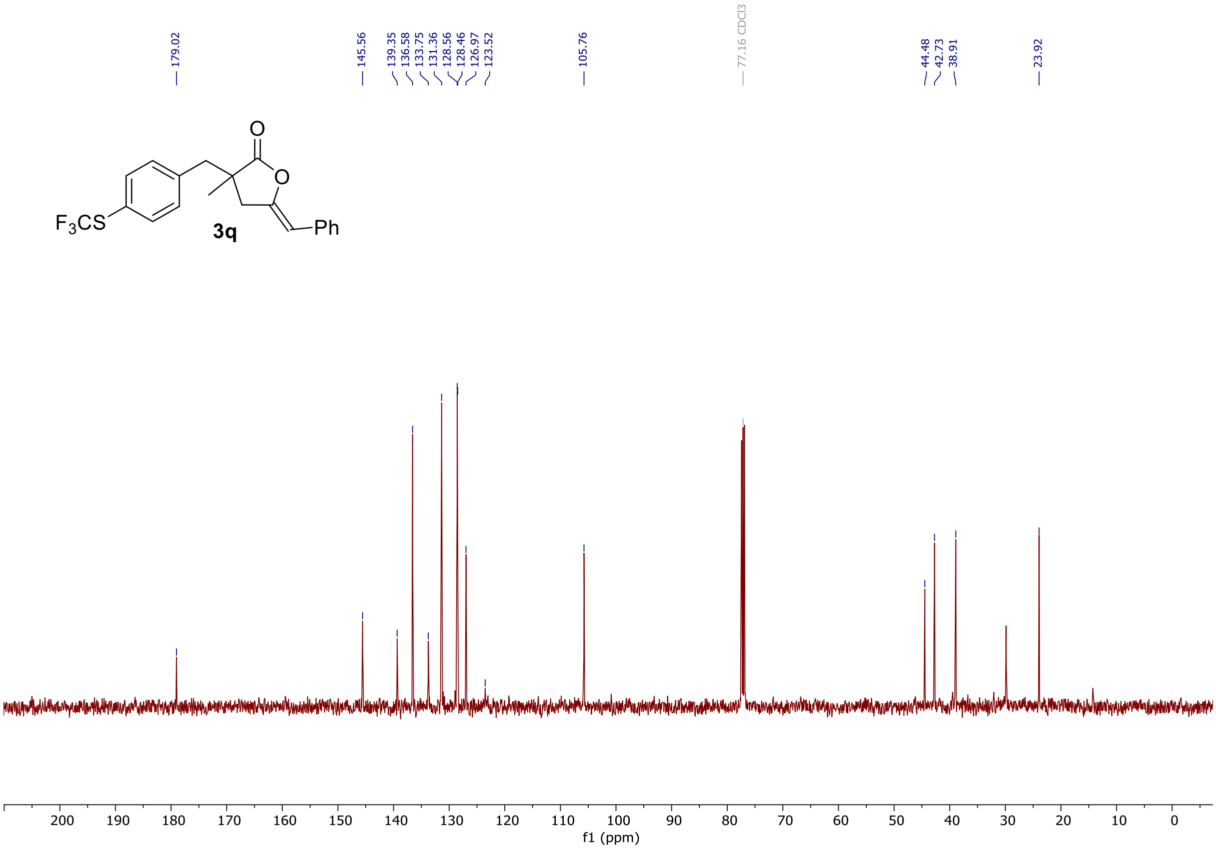
179.02
145.56
139.35
136.58
133.75
131.36
128.56
128.46
126.97
123.52
105.76
77.16 CDCl3
44.48
42.73
38.91
23.92
F3CS
3q
Ph
f1 (ppm)

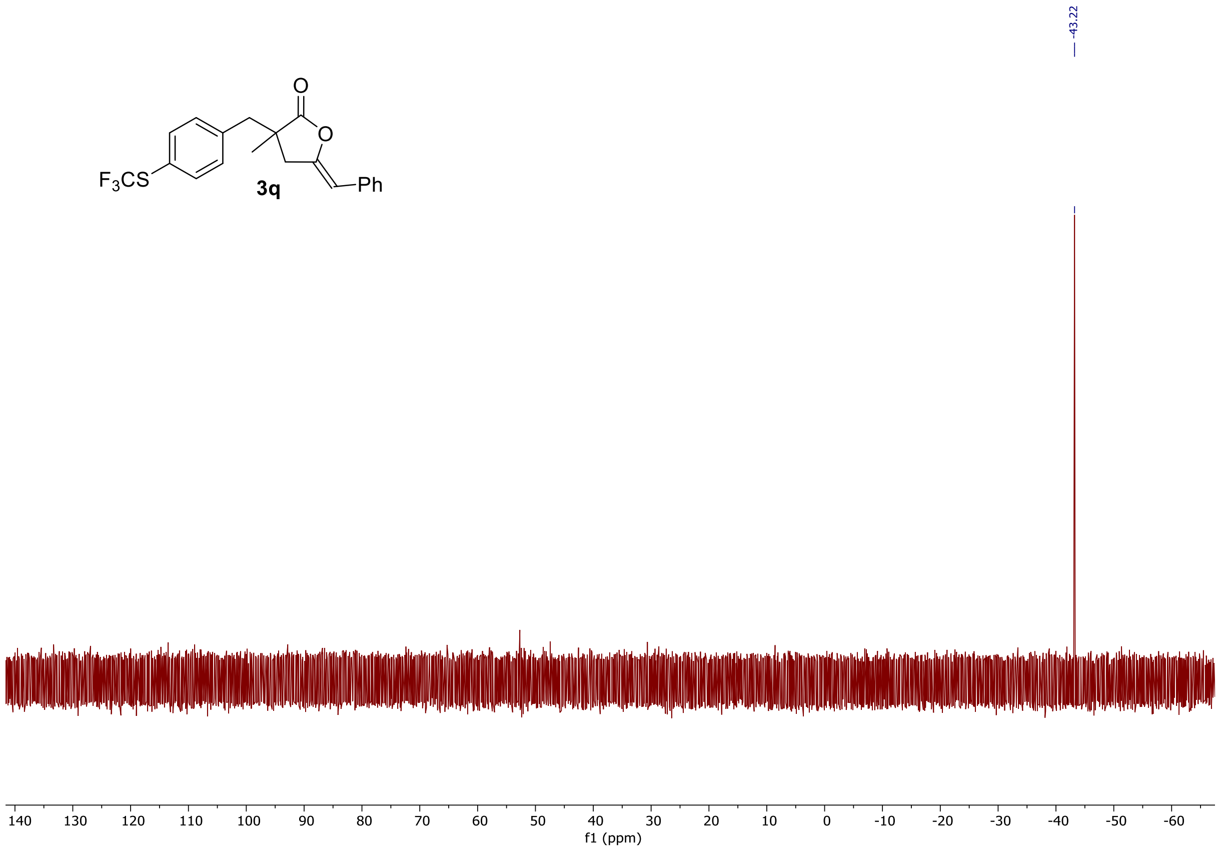
O
O
F3CS
3q
Ph
-43.22
140 130 120 110 100 90 80 70 60 50 40 30 20 10 0 -10 -20 -30 -40 -50 -60
f1 (ppm)

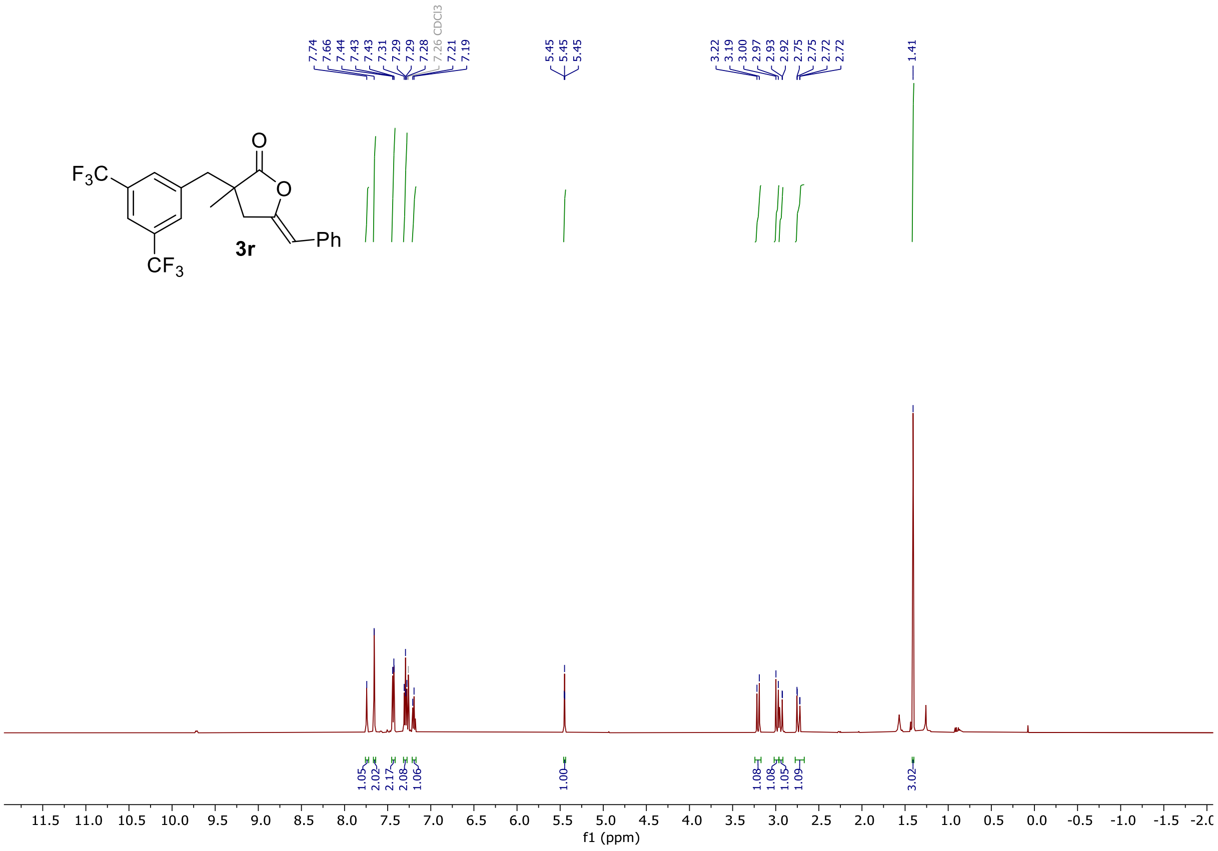
7.74
7.66
7.44
7.43
7.43
7.31
7.29
7.29
7.28
7.26 CDCl3
7.21
7.19
5.45
5.45
5.45
3.22
3.19
3.00
2.97
2.93
2.92
2.75
2.75
2.72
2.72
1.41
F3C
O
O
Ph
CF3
3r
1.05
2.02
2.17
2.08
1.06
1.00
1.08
1.08
1.05
1.09
3.02
f1 (ppm)

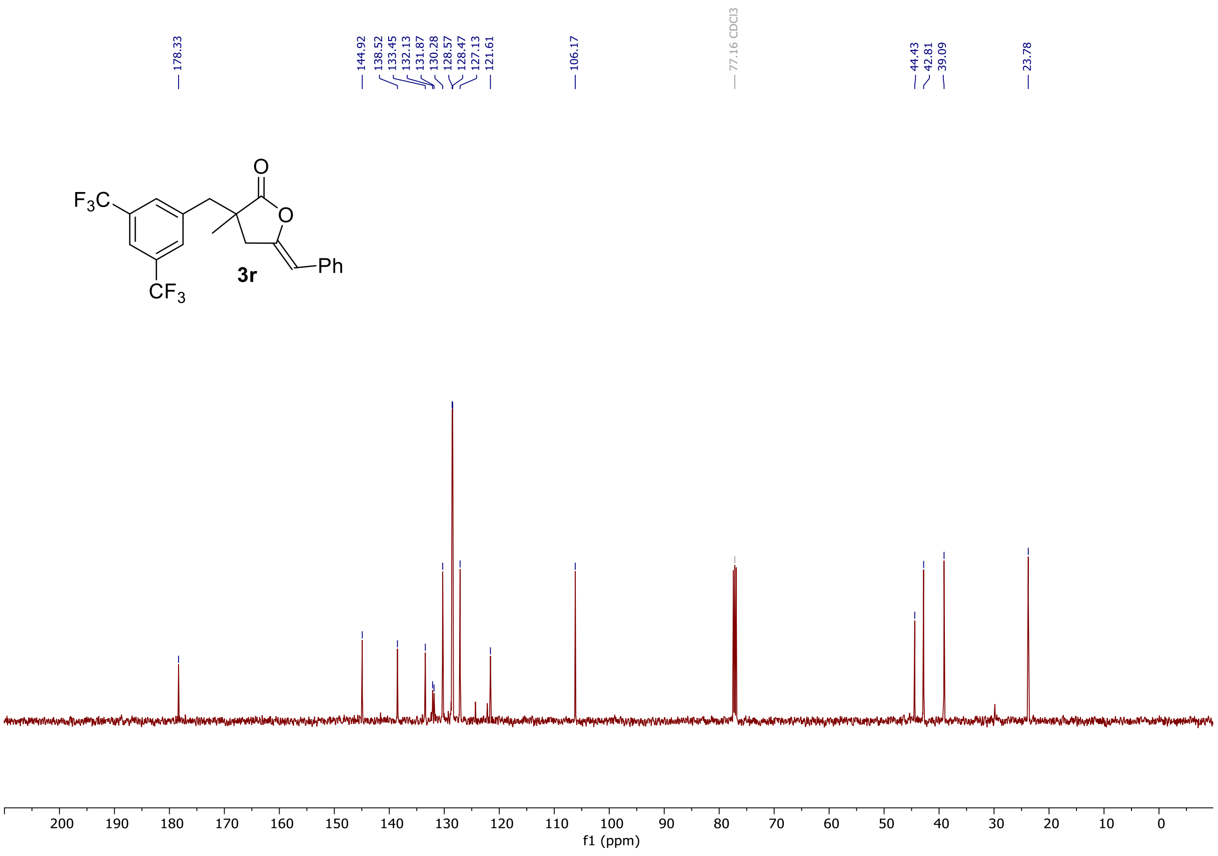
178.33
144.92
138.52
133.45
132.13
131.87
130.28
128.57
128.47
127.13
121.61
106.17
77.16 CDCl3
44.43
42.81
39.09
23.78
F3C
O
O
Ph
CF3
3r
f1 (ppm)

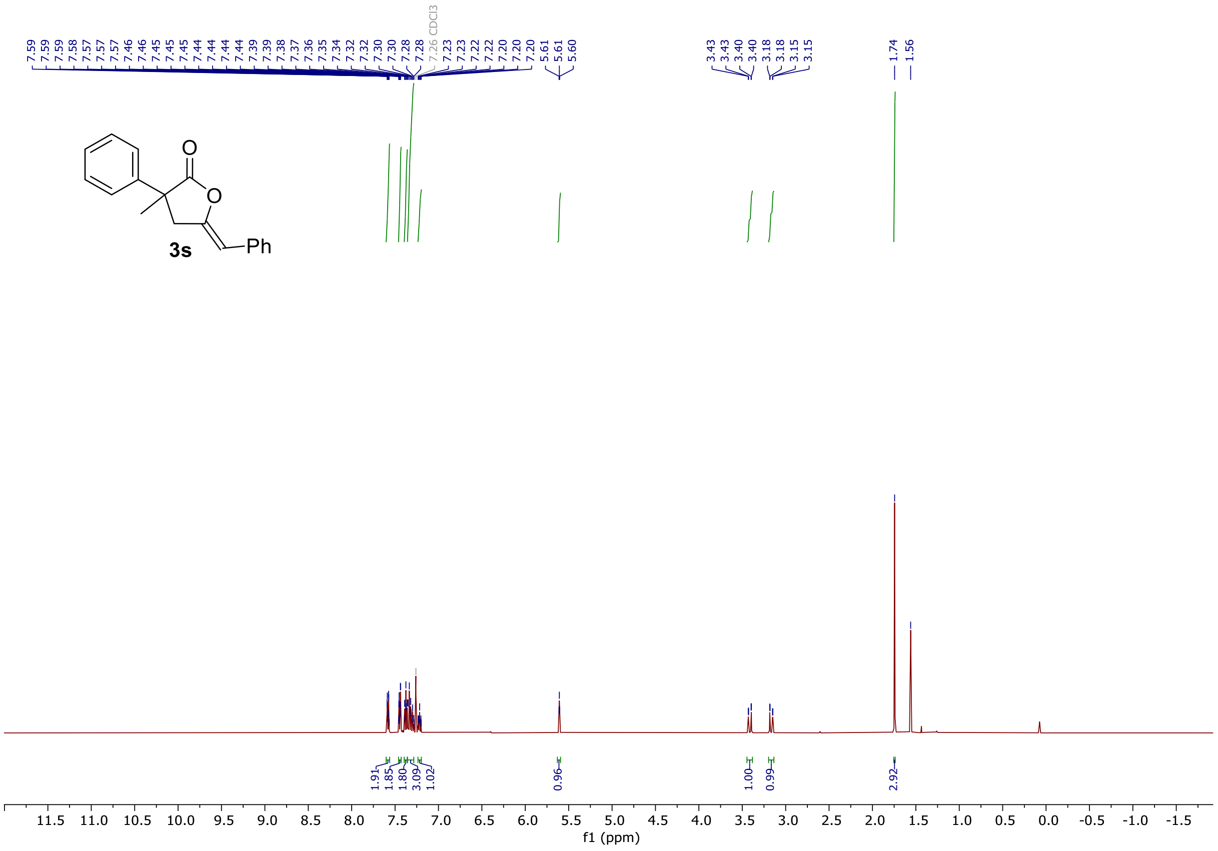
7.59 7.59 7.58 7.57 7.57 7.57 7.46 7.45 7.45 7.45 7.44 7.44 7.44 7.44 7.39 7.39 7.38 7.37 7.36 7.35 7.34 7.32 7.32 7.30 7.30 7.28 7.28 7.26 CDCl3 7.23 7.23 7.23 7.22 7.22 7.20 7.20 7.20 5.61 5.61 5.60
3.43 3.43 3.40 3.40 3.18 3.18 3.15 3.15
1.74 1.56
O
O
3s
Ph
1.91 1.85 1.80 3.09 1.02
0.96
1.00 0.99
2.92
11.5 11.0 10.5 10.0 9.5 9.0 8.5 8.0 7.5 7.0 6.5 6.0 5.5 5.0 4.5 4.0 3.5 3.0 2.5 2.0 1.5 1.0 0.5 0.0 -0.5 -1.0 -1.5
f1 (ppm)

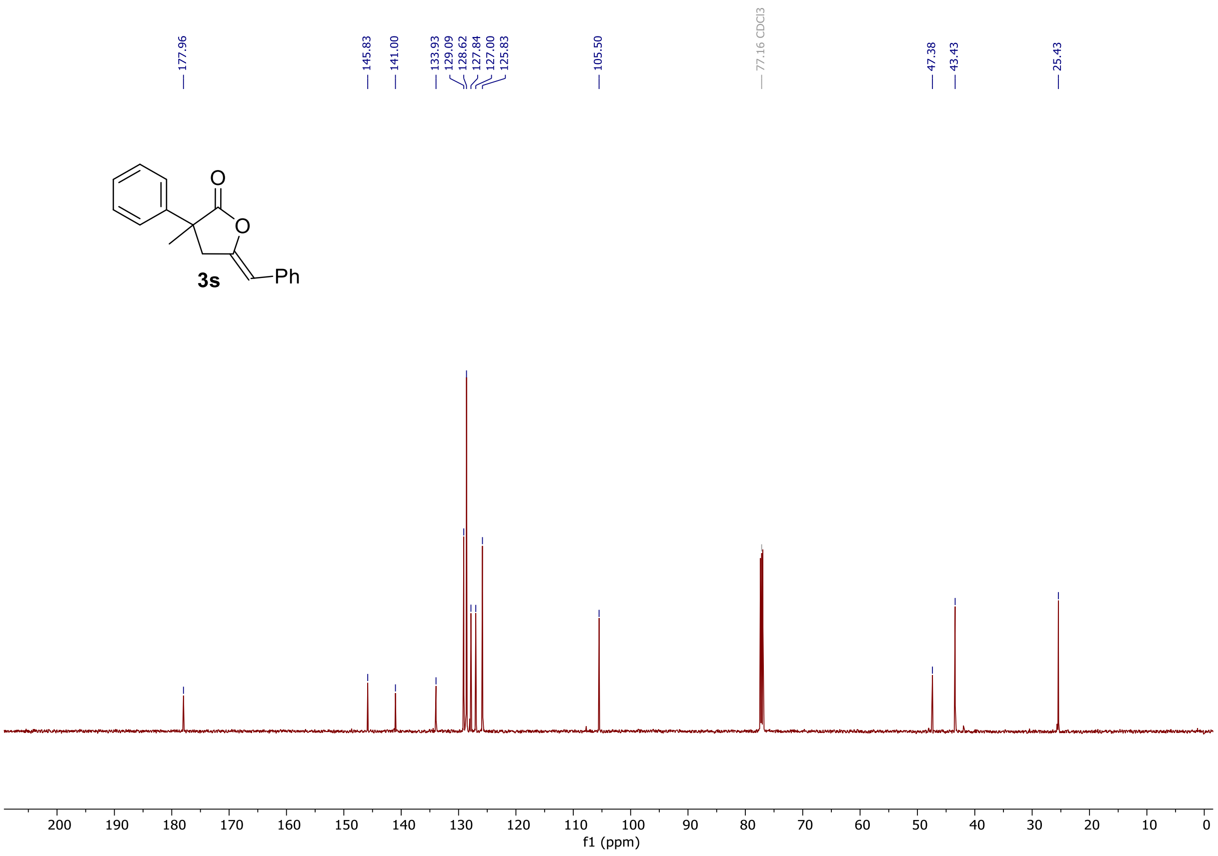
177.96
145.83 141.00
133.93 129.09 128.62 127.84 127.00 125.83
105.50
77.16 CDCl3
47.38 43.43
25.43
O
O
3s
Ph
200 190 180 170 160 150 140 130 120 110 100 90 80 70 60 50 40 30 20 10 0
f1 (ppm)

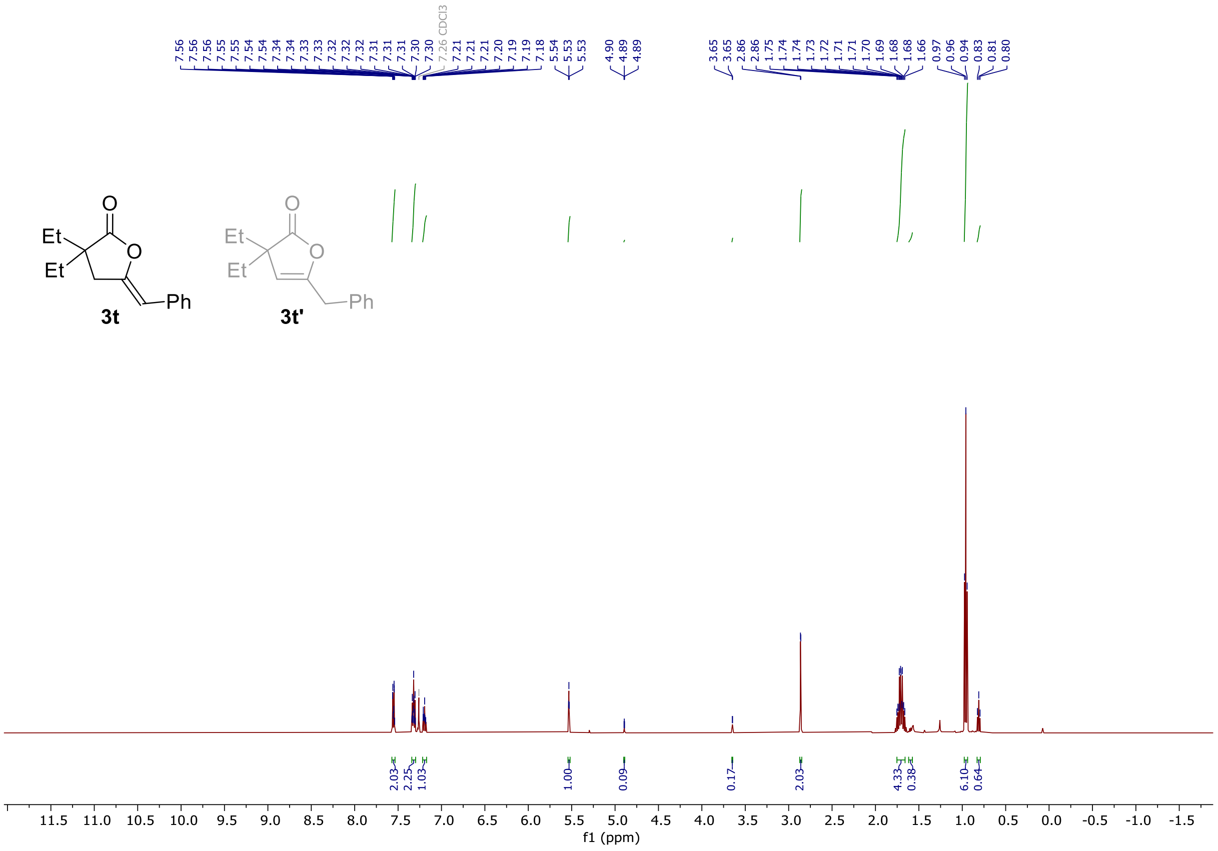
7.56
7.56
7.56
7.55
7.55
7.54
7.34
7.34
7.33
7.33
7.32
7.32
7.31
7.31
7.30
7.30
7.26 CDCl3
7.21
7.21
7.21
7.20
7.19
7.19
7.18
5.54
5.53
5.53
4.90
4.89
4.89
3.65
3.65
2.86
1.75
1.74
1.74
1.73
1.72
1.71
1.70
1.69
1.68
1.68
1.66
0.97
0.96
0.94
0.83
0.81
0.80
Et
Et
O
O
Ph
3t
3t'
2.03
2.25
1.03
1.00
0.09
0.17
2.03
4.33
0.38
6.10
0.64
f1 (ppm)

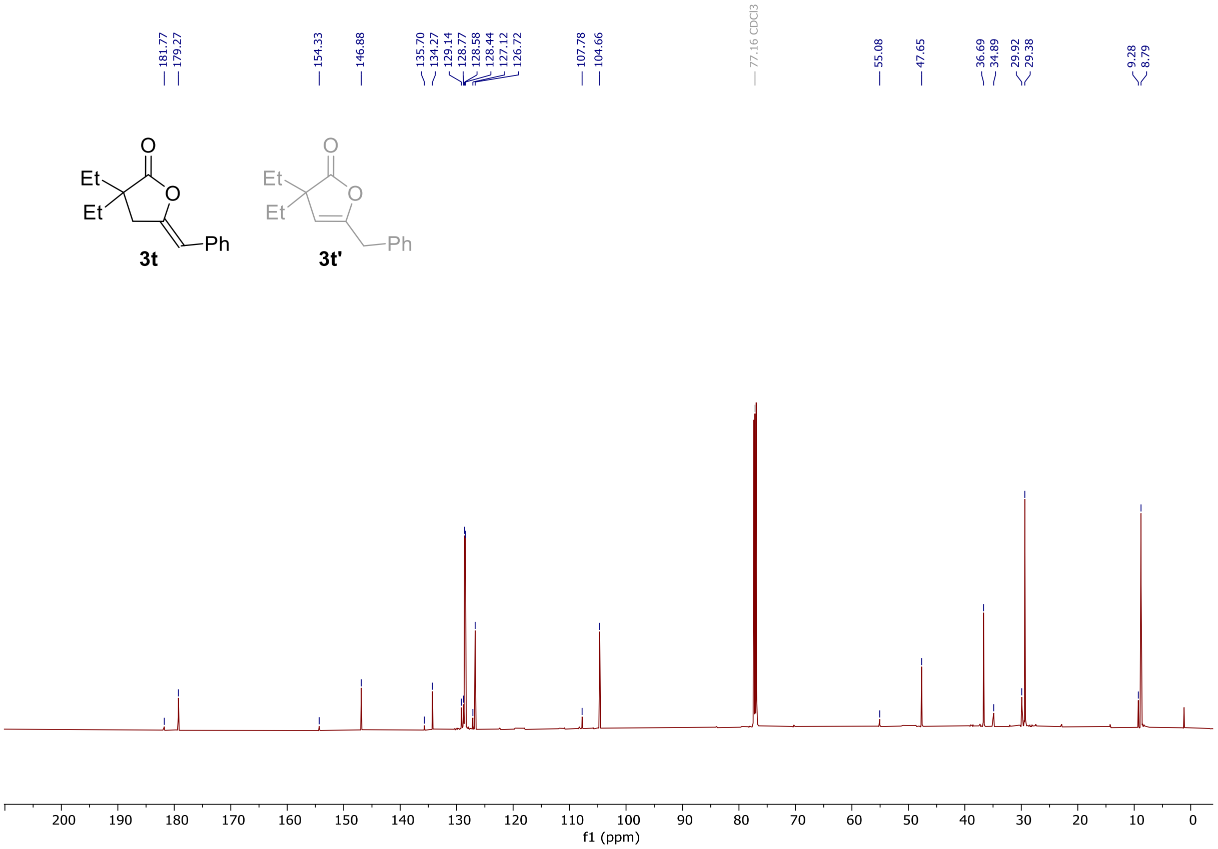
181.77
179.27
154.33
146.88
135.70
134.27
129.14
128.77
128.58
128.44
127.12
126.72
107.78
104.66
77.16 CDCl3
55.08
47.65
36.69
34.89
29.92
29.38
9.28
8.79
Et
Et
O
O
Ph
3t
3t'
f1 (ppm)

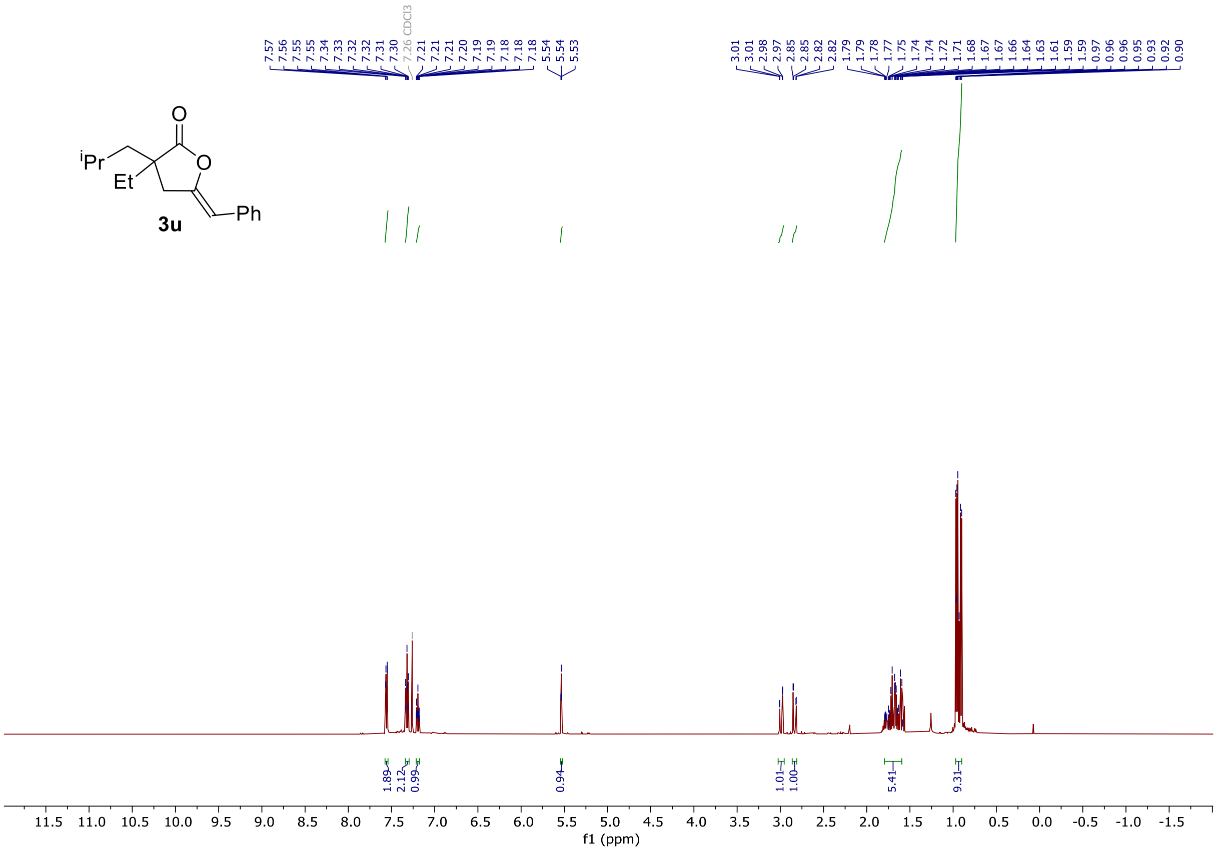

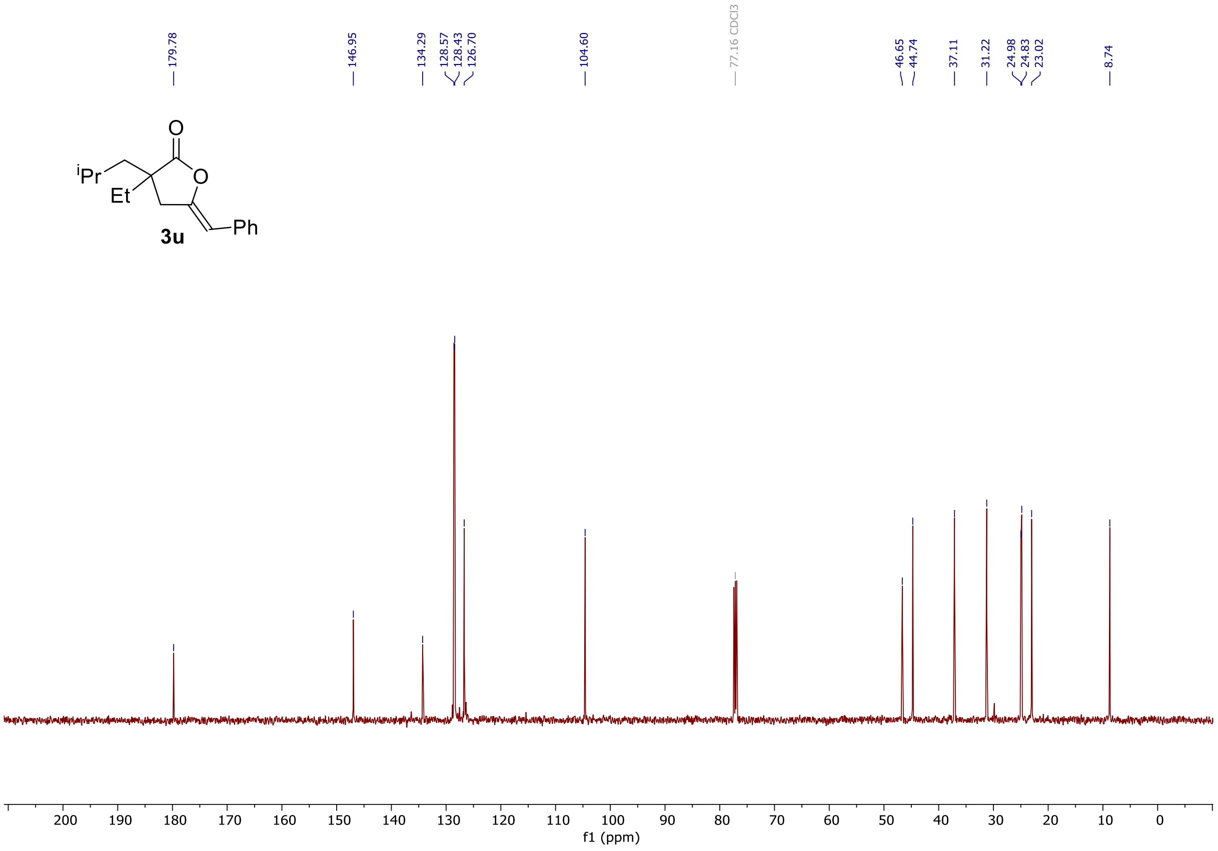

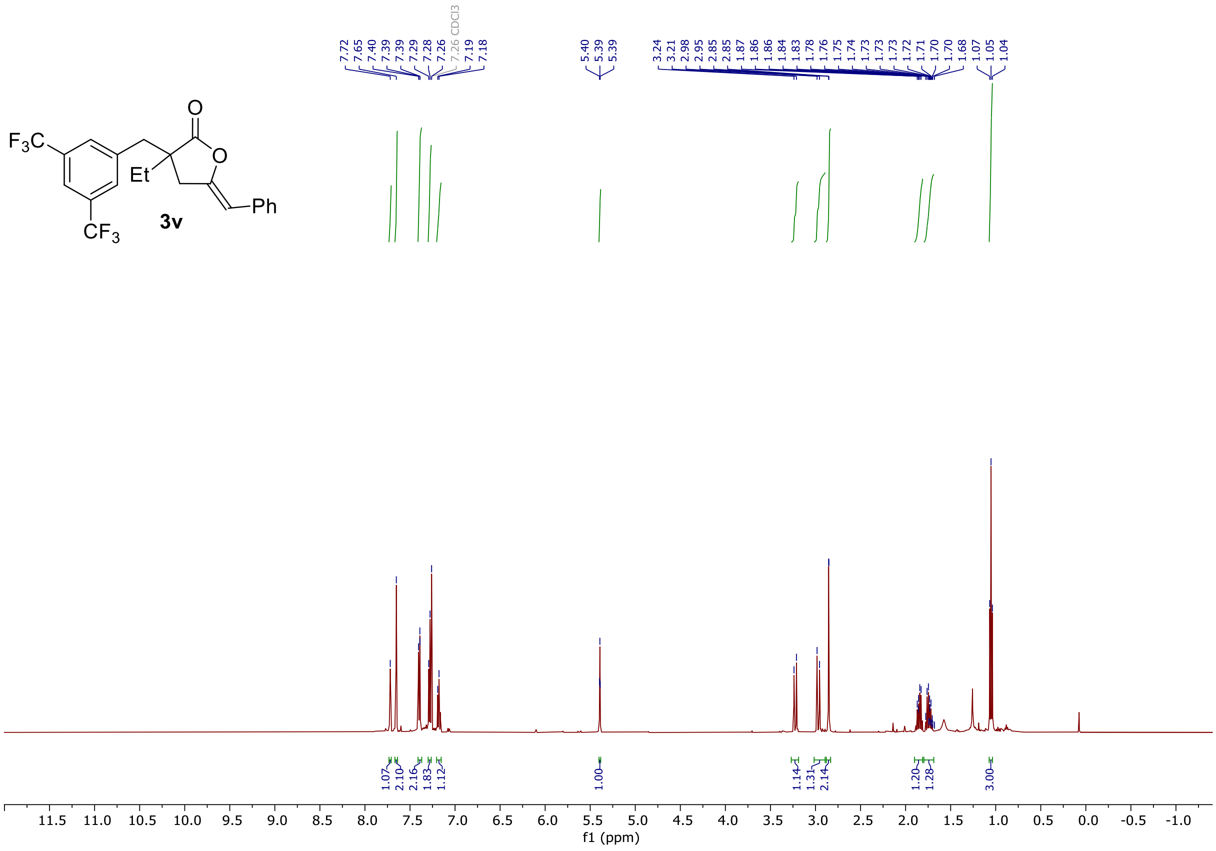
7.72 7.65 7.40 7.39 7.39 7.29 7.28 7.26 CDCl3 7.19 7.18
5.40 5.39 5.39
3.24 3.21 2.98 2.95 2.85 2.85 1.87 1.86 1.86 1.84 1.83 1.78 1.76 1.75 1.74 1.73 1.73 1.72 1.71 1.70 1.70 1.68 1.07 1.05 1.04
F3C
CF3
Et
O
Ph
3v
1.07 2.10 2.16 1.83 1.12
1.00
1.14 1.31 2.14
1.20 1.28
3.00
11.5 11.0 10.5 10.0 9.5 9.0 8.5 8.0 7.5 7.0 6.5 6.0 5.5 5.0 4.5 4.0 3.5 3.0 2.5 2.0 1.5 1.0 0.5 0.0 -0.5 -1.0
f1 (ppm)

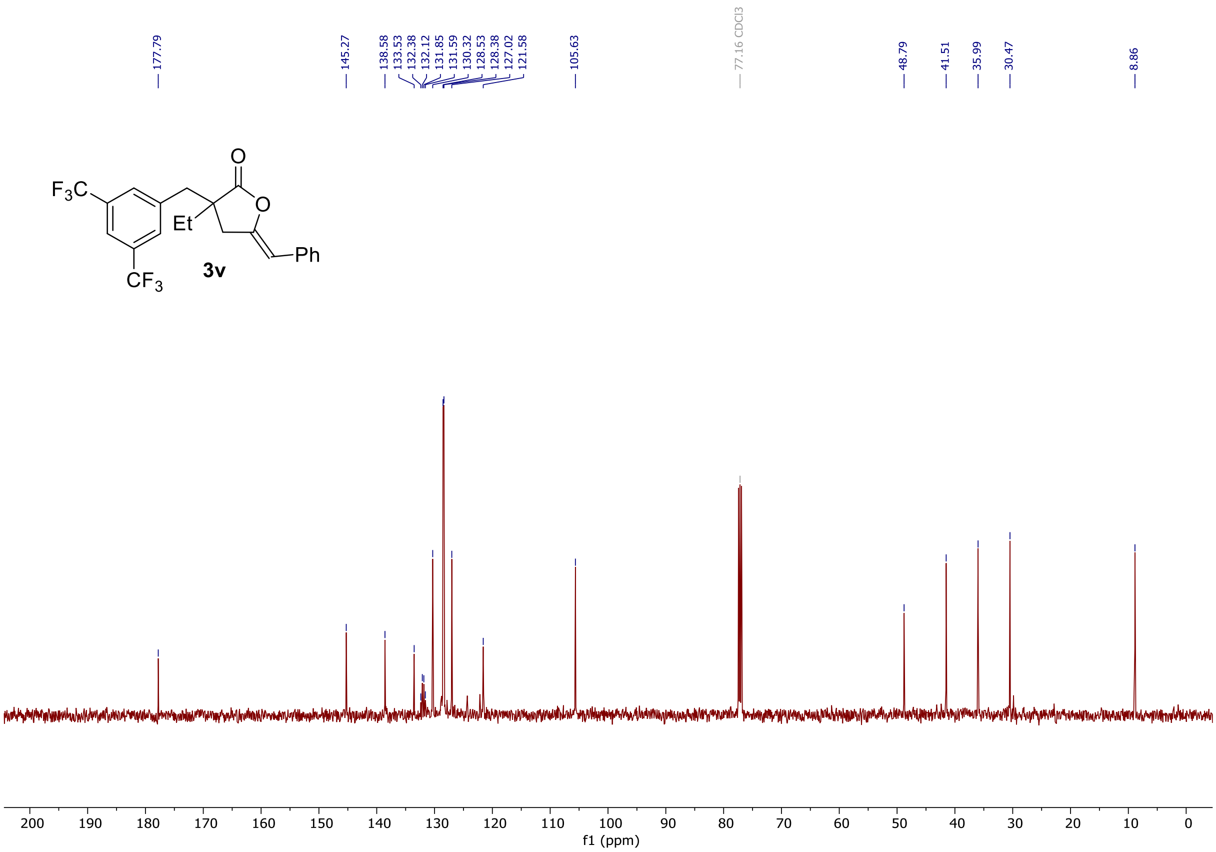
177.79
145.27
138.58 133.53 132.38 132.12 131.85 131.59 130.32 128.53 128.38 127.02 121.58
105.63
77.16 CDCl3
48.79
41.51
35.99
30.47
8.86
F3C
CF3
Et
O
Ph
3v
200 190 180 170 160 150 140 130 120 110 100 90 80 70 60 50 40 30 20 10 0
f1 (ppm)

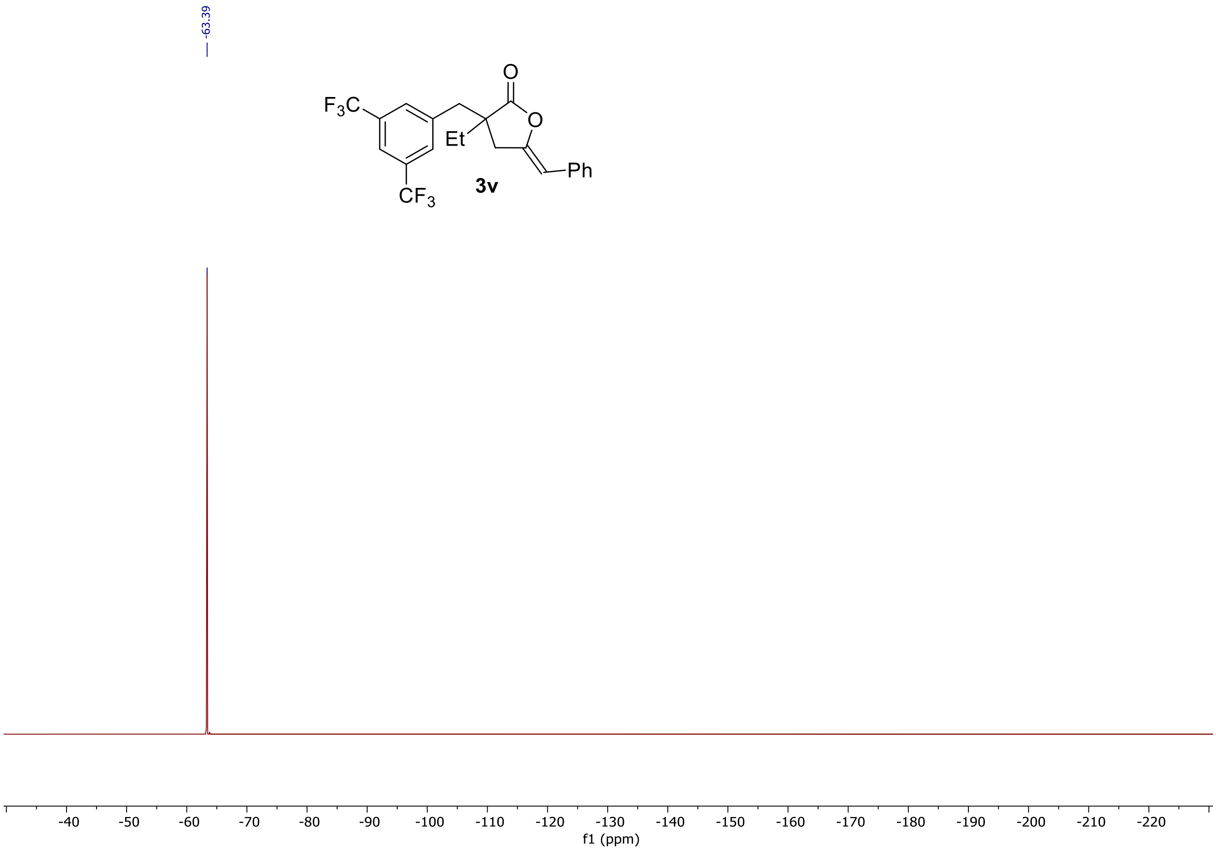
-63.39
F3C
CF3
Et
O
O
Ph
3v
-40
-50
-60
-70
-80
-90
-100
-110
-120
-130
-140
-150
-160
-170
-180
-190
-200
-210
-220
f1 (ppm)

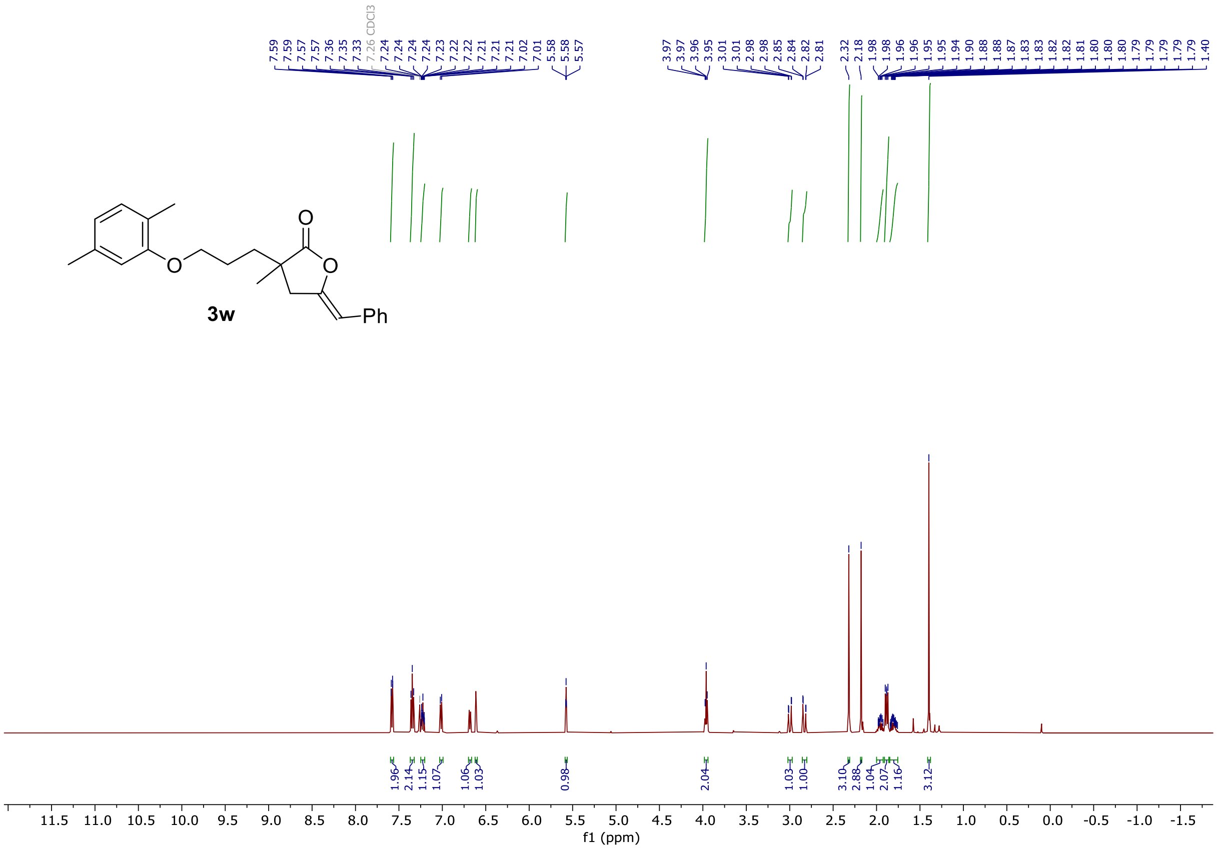
7.59 7.59 7.57 7.36 7.35 7.33 7.26 CDCl3 7.24 7.24 7.24 7.24 7.23 7.22 7.22 7.21 7.21 7.21 7.02 7.01 5.58 5.58 5.57
3.97 3.97 3.96 3.95 3.01 3.01 2.98 2.98 2.85 2.84 2.82 2.81
2.32 2.18 1.98 1.98 1.96 1.96 1.95 1.95 1.94 1.90 1.88 1.88 1.87 1.83 1.83 1.82 1.82 1.81 1.80 1.80 1.79 1.79 1.79 1.79 1.40
O
O
Ph
3w
1.96 2.14 1.15 1.07 1.06 1.03 0.98 2.04 1.03 1.00 3.10 2.88 1.04 2.07 1.16 3.12
11.5 11.0 10.5 10.0 9.5 9.0 8.5 8.0 7.5 7.0 6.5 6.0 5.5 5.0 4.5 4.0 3.5 3.0 2.5 2.0 1.5 1.0 0.5 0.0 -0.5 -1.0 -1.5
f1 (ppm)

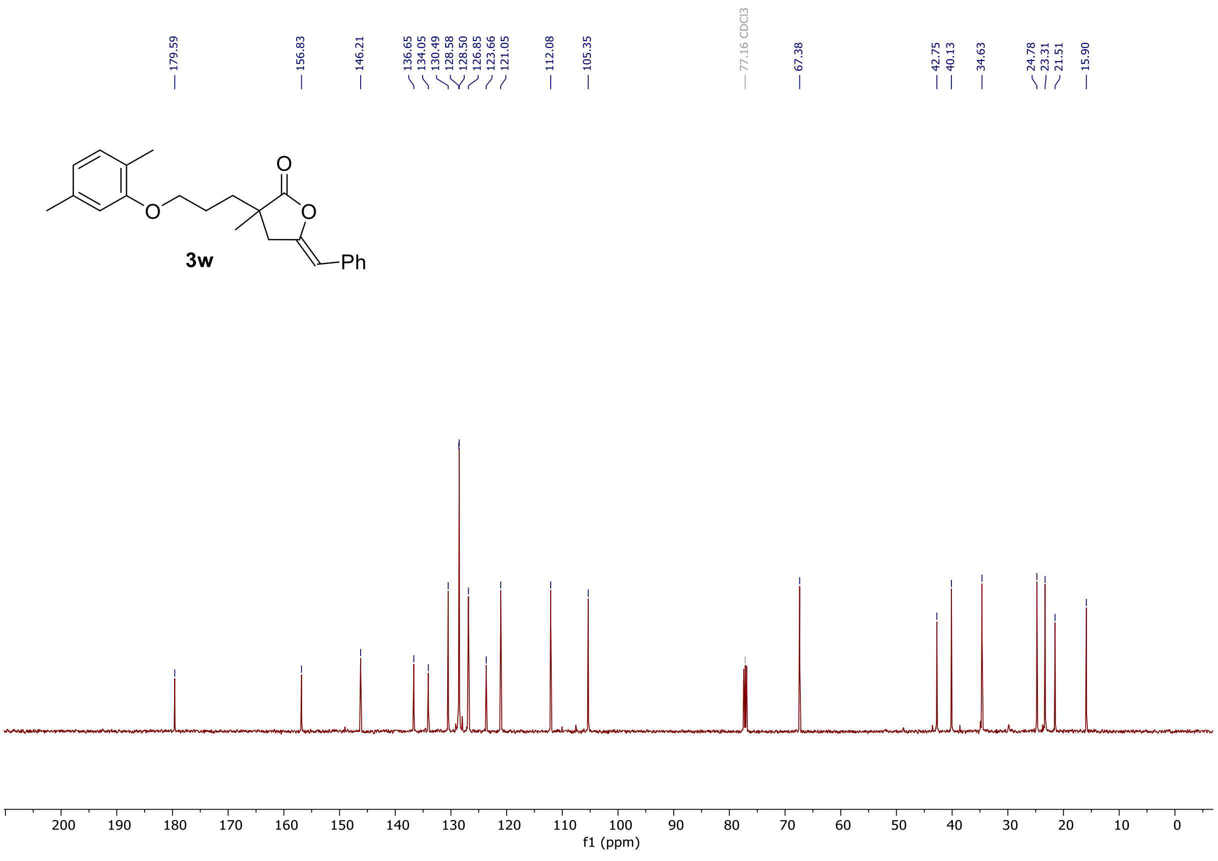
179.59
156.83
146.21
136.65 134.05 130.49 128.58 128.50 126.85 123.66 121.05
112.08
105.35
77.16 CDCl3
67.38
42.75 40.13
34.63
24.78 23.31 21.51
15.90
O
O
Ph
3w
200 190 180 170 160 150 140 130 120 110 100 90 80 70 60 50 40 30 20 10 0
f1 (ppm)

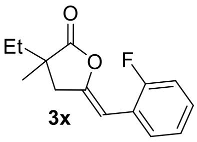
O
Et
O
F

3x

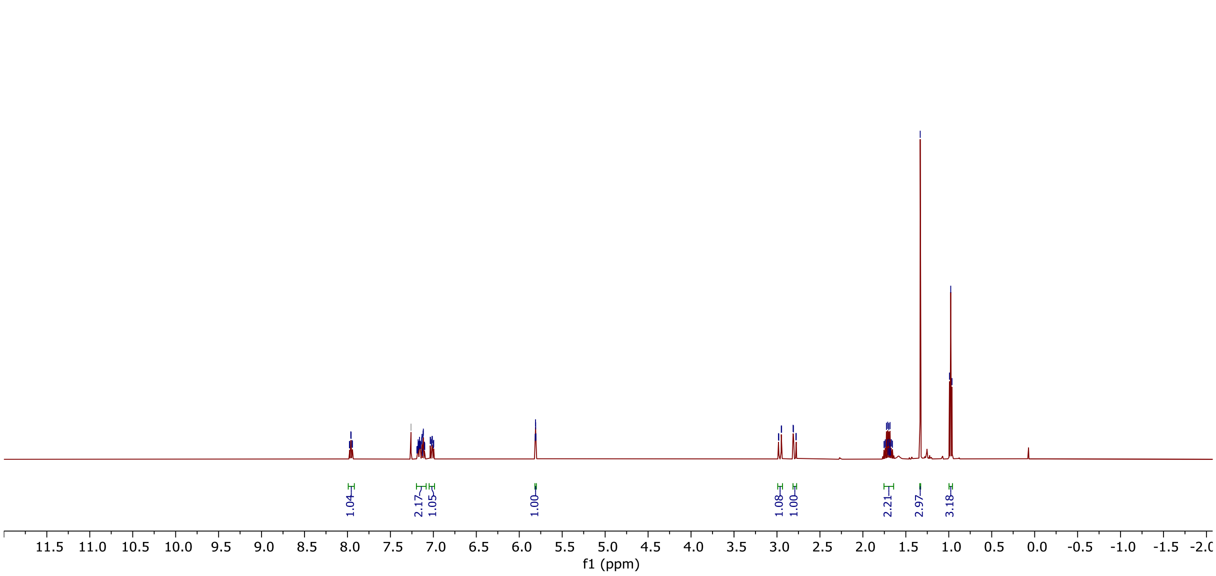
7.98
7.97
7.96
7.96
7.95
7.94
7.26 CDCl3
7.19
7.18
7.18
7.17
7.17
7.16
7.16
7.15
7.15
7.14
7.13
7.13
7.13
7.12
7.12
7.10
7.10
7.10
7.04
7.03
7.02
7.02
7.01
7.01
7.00
7.00
7.00
5.81
5.81
5.81
5.81
2.98
2.98
2.95
2.95
2.81
2.81
2.78
2.78
1.75
1.74
1.74
1.73
1.71
1.71
1.70
1.69
1.68
1.67
1.67
1.66
1.33
0.99
0.98
0.96
1.04
2.17
1.05
1.00
1.08
1.00
2.21
2.97
3.18
11.5
11.0
10.5
10.0
9.5
9.0
8.5
8.0
7.5
7.0
6.5
6.0
5.5
5.0
4.5
4.0
3.5
3.0
2.5
2.0
1.5
1.0
0.5
0.0
-0.5
-1.0
-1.5
-2.0
f1 (ppm)

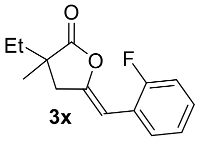
O
Et
O
F

3x

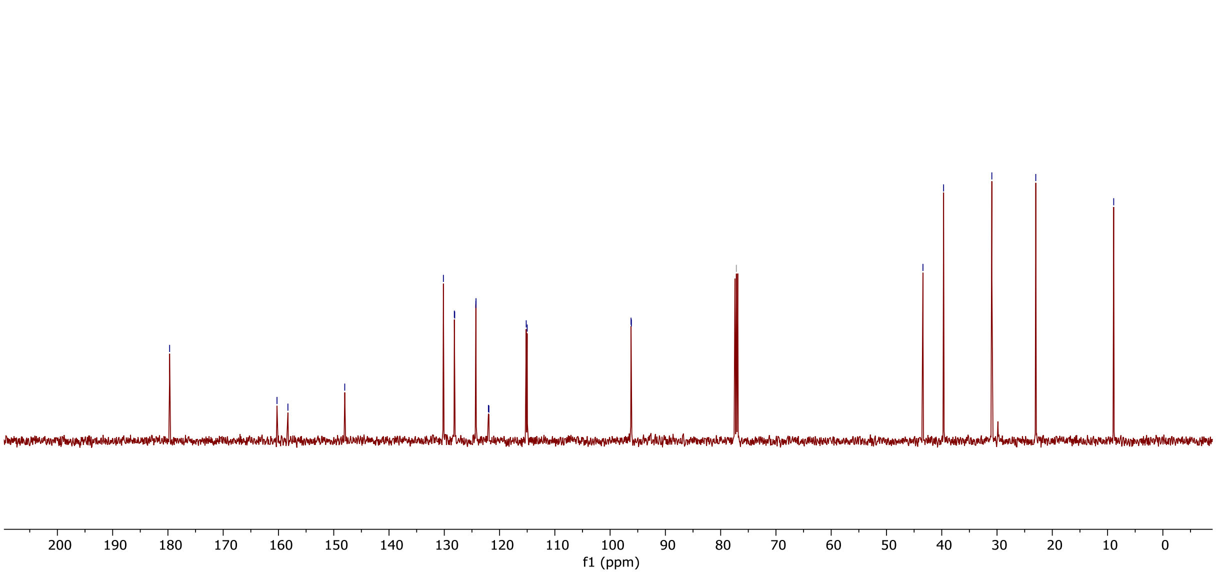
179.69
160.27
158.29
148.01
130.16
128.18
128.11
124.29
124.26
122.02
121.93
115.18
115.00
96.22
96.15
77.16 CDCl3
43.40
39.67
30.94
22.99
8.89
200
190
180
170
160
150
140
130
120
110
100
90
80
70
60
50
40
30
20
10
0
f1 (ppm)

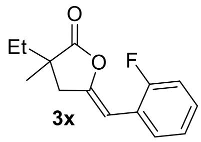

3x

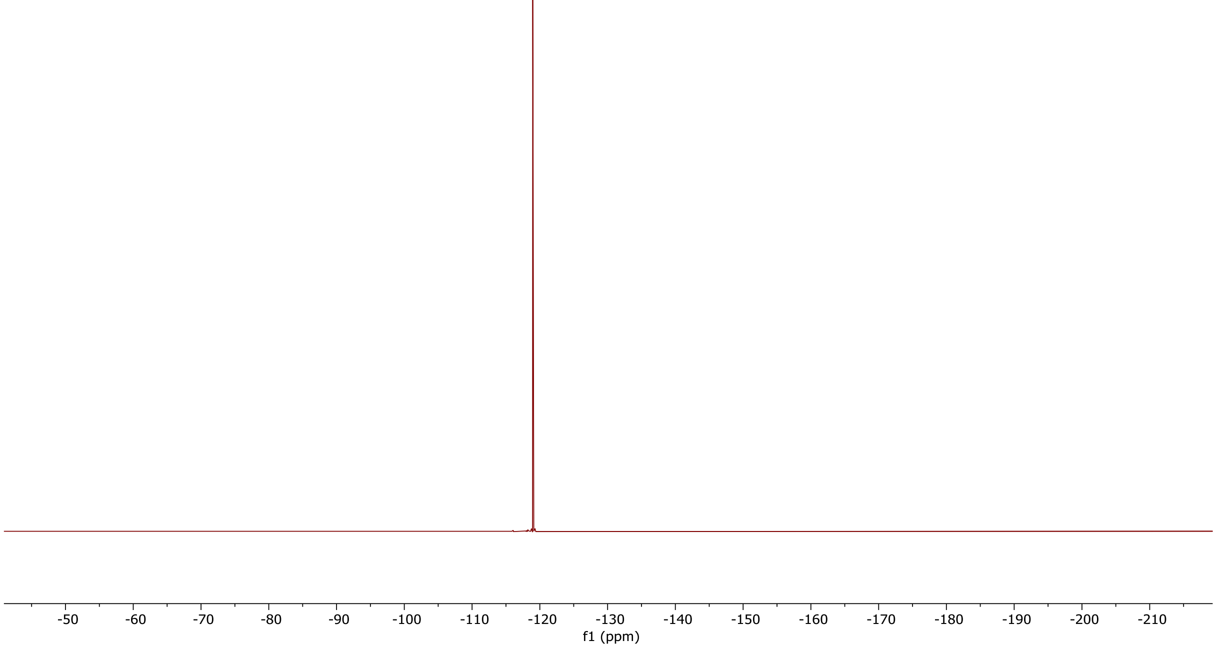
-118.95
-50
-60
-70
-80
-90
-100
-110
-120
-130
-140
-150
-160
-170
-180
-190
-200
-210
f1 (ppm)

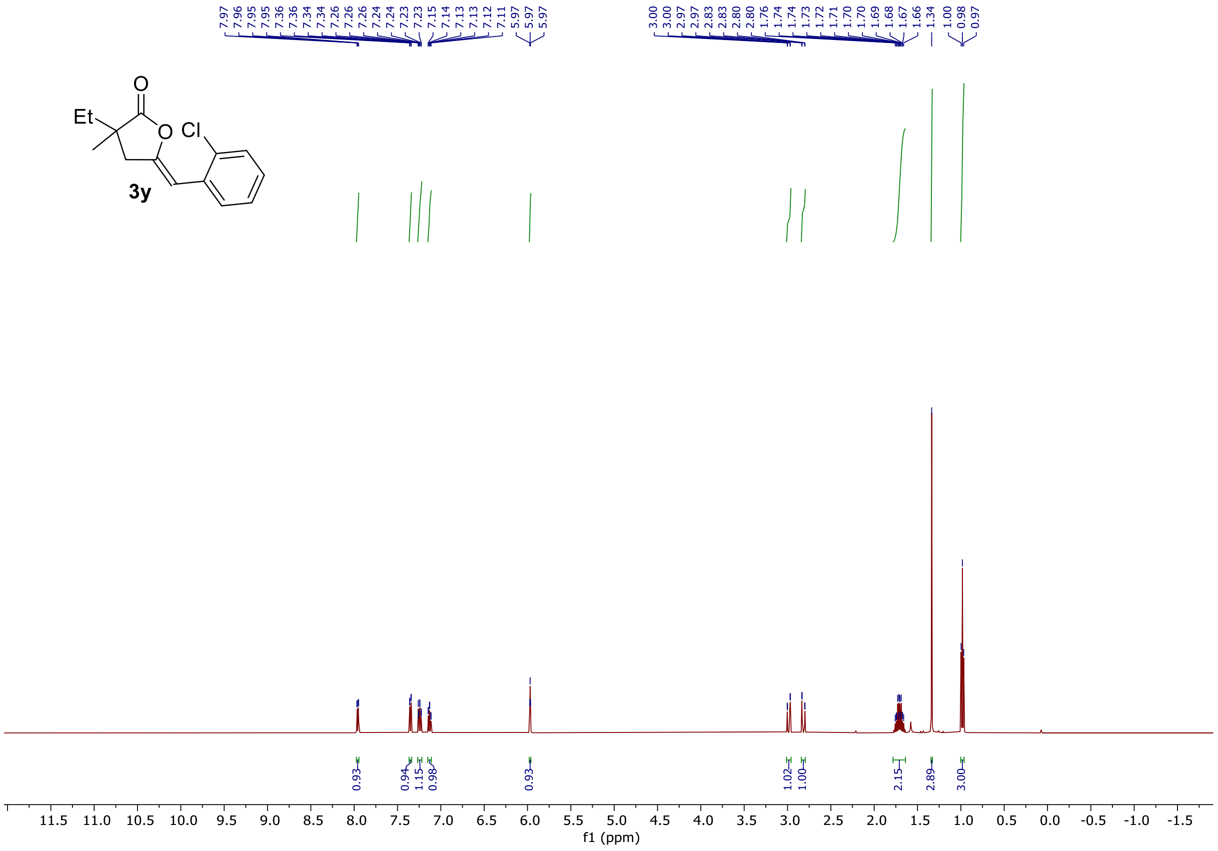
7.97 7.96 7.95 7.36 7.36 7.34 7.34 7.26 7.26 7.24 7.24 7.23 7.23 7.15 7.14 7.13 7.13 7.12 7.11 5.97 5.97 5.97
3.00 3.00 2.97 2.97 2.83 2.83 2.80 2.80 1.76 1.74 1.74 1.73 1.72 1.71 1.70 1.70 1.69 1.68 1.67 1.66 1.34 1.00 0.98 0.97
Et
O
O
Cl
3y
0.93 0.94 1.15 0.98 0.93 1.02 1.00 2.15 2.89 3.00
f1 (ppm)

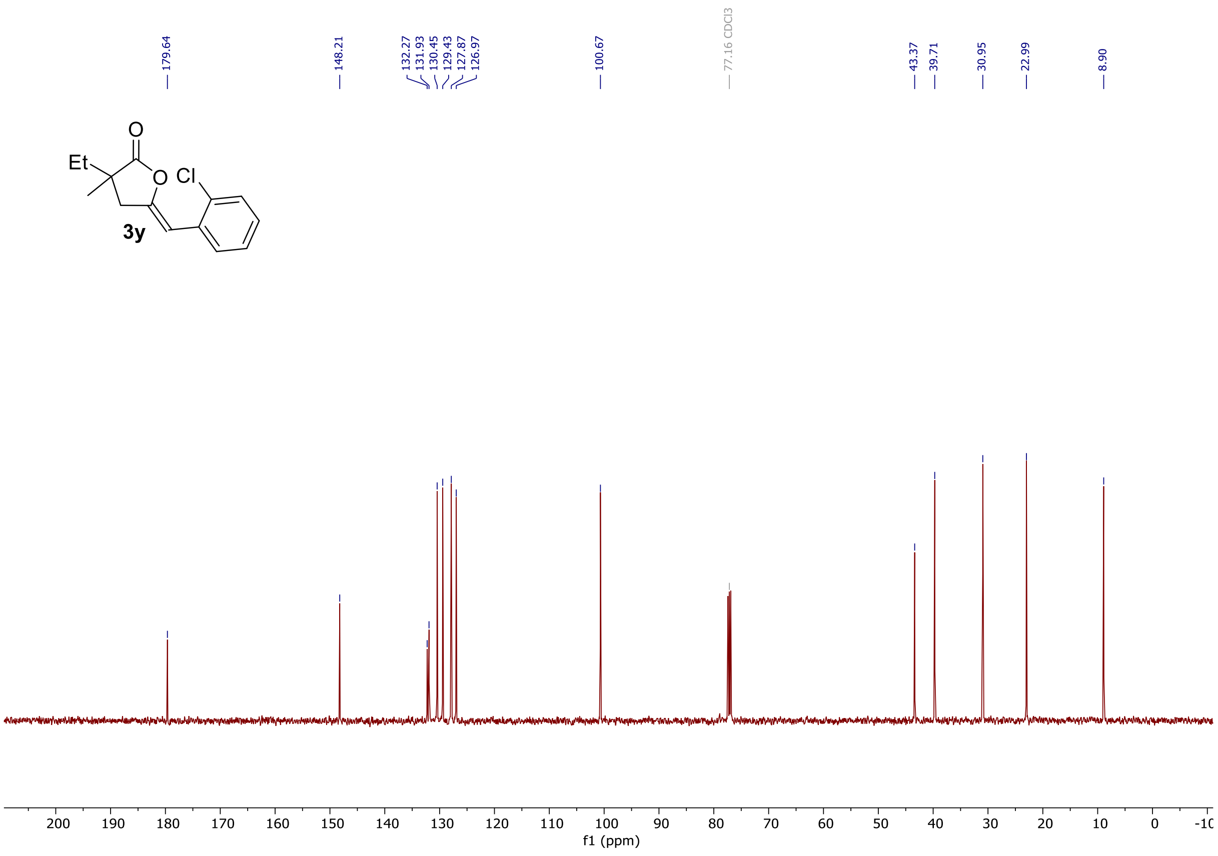
179.64
148.21
132.27 131.93 130.45 129.43 127.87 126.97
100.67
77.16 CDCl3
43.37 39.71 30.95 22.99 8.90
Et
O
O
Cl
3y
f1 (ppm)

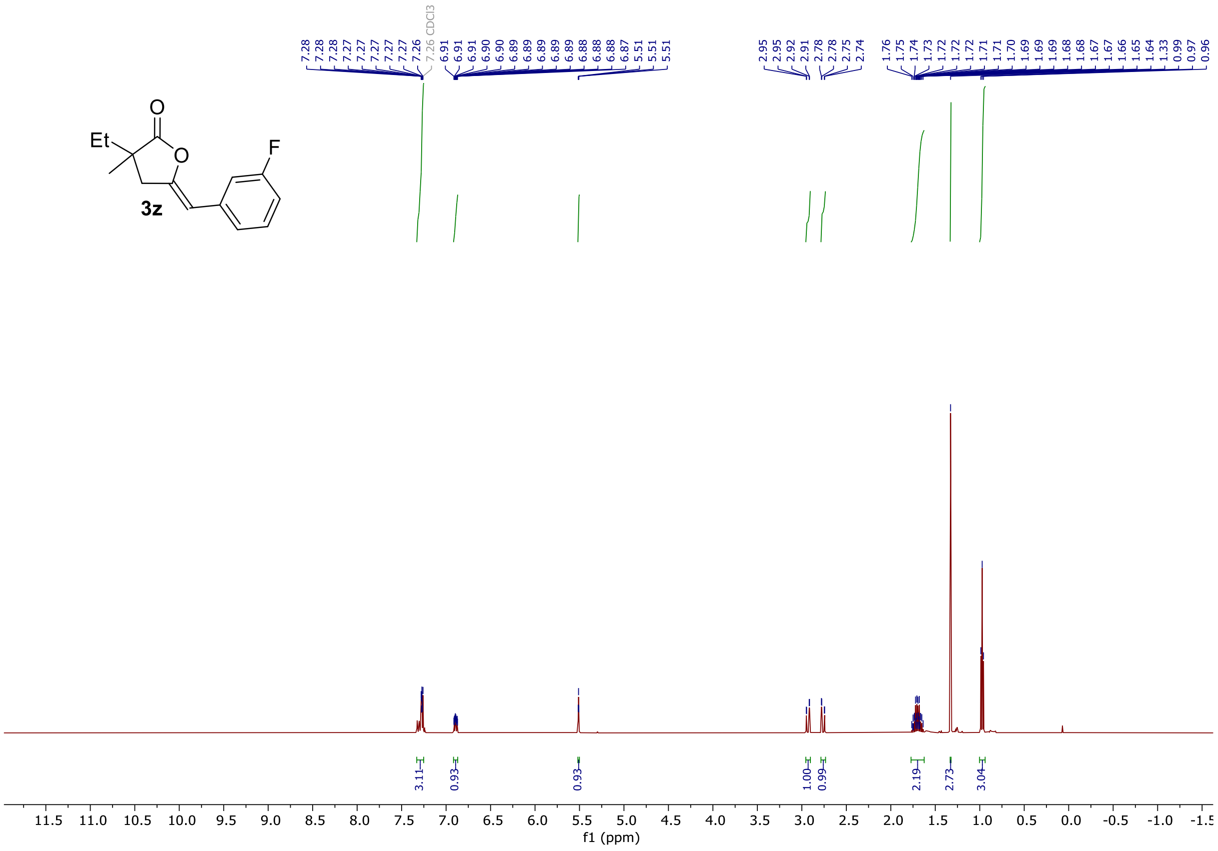
Et
O
O
F
3z
7.28 7.28 7.28 7.27 7.27 7.27 7.27 7.27 7.26 7.26 CDCl3
6.91 6.91 6.91 6.91 6.90 6.90 6.89 6.89 6.89 6.89 6.89 6.88 6.88 6.88 6.87 5.51 5.51 5.51 5.51
2.95 2.95 2.92 2.91 2.78 2.78 2.75 2.74
1.76 1.75 1.74 1.73 1.72 1.72 1.72 1.72 1.71 1.70 1.69 1.69 1.69 1.68 1.68 1.67 1.67 1.66 1.65 1.64 1.33 0.99 0.97 0.96
3.11 0.93 0.93 1.00 0.99 2.19 2.73 3.04
11.5 11.0 10.5 10.0 9.5 9.0 8.5 8.0 7.5 7.0 6.5 6.0 5.5 5.0 4.5 4.0 3.5 3.0 2.5 2.0 1.5 1.0 0.5 0.0 -0.5 -1.0
f1 (ppm)

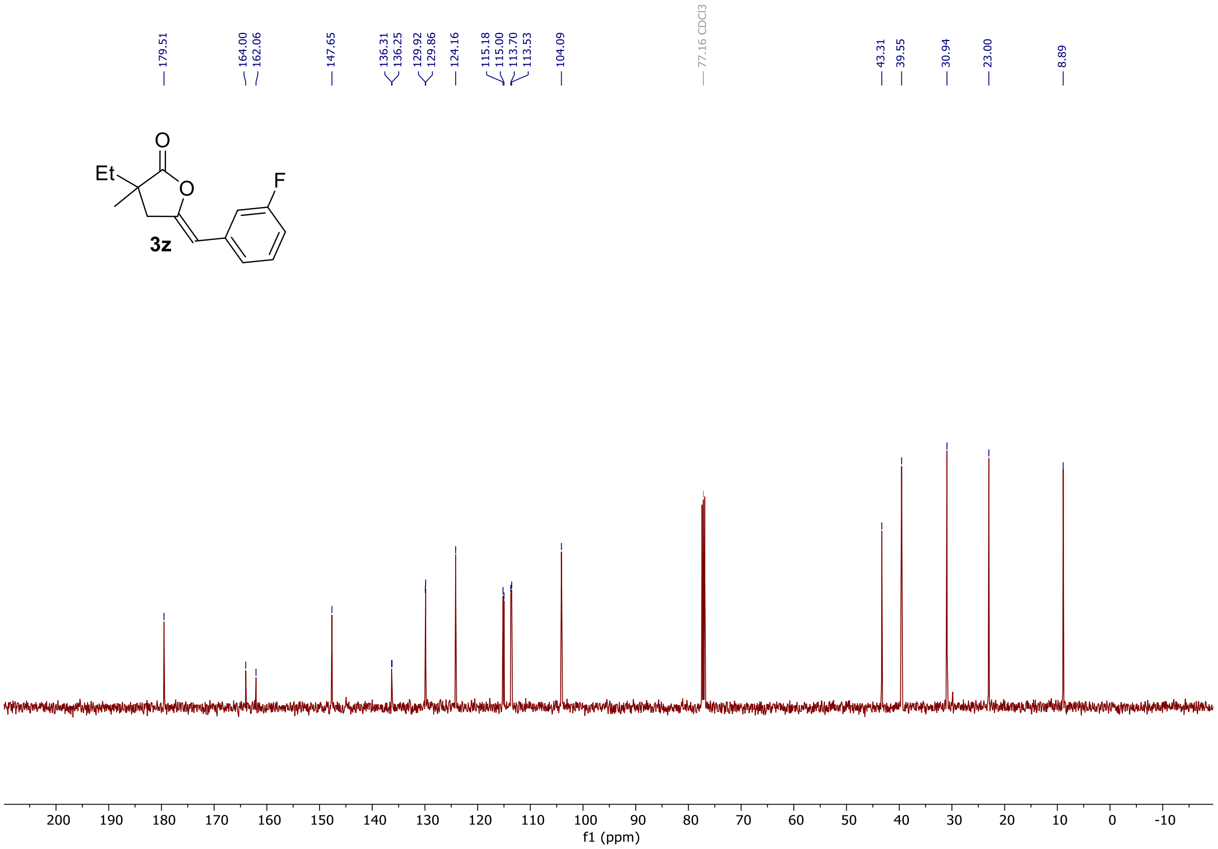
Et
O
O
F
3z
179.51
164.00 162.06
147.65
136.31 136.25 129.92 129.86
124.16
115.18 115.00 113.70 113.53
104.09
77.16 CDCl3
43.31 39.55 30.94 23.00 8.89
200 190 180 170 160 150 140 130 120 110 100 90 80 70 60 50 40 30 20 10 0 -10
f1 (ppm)

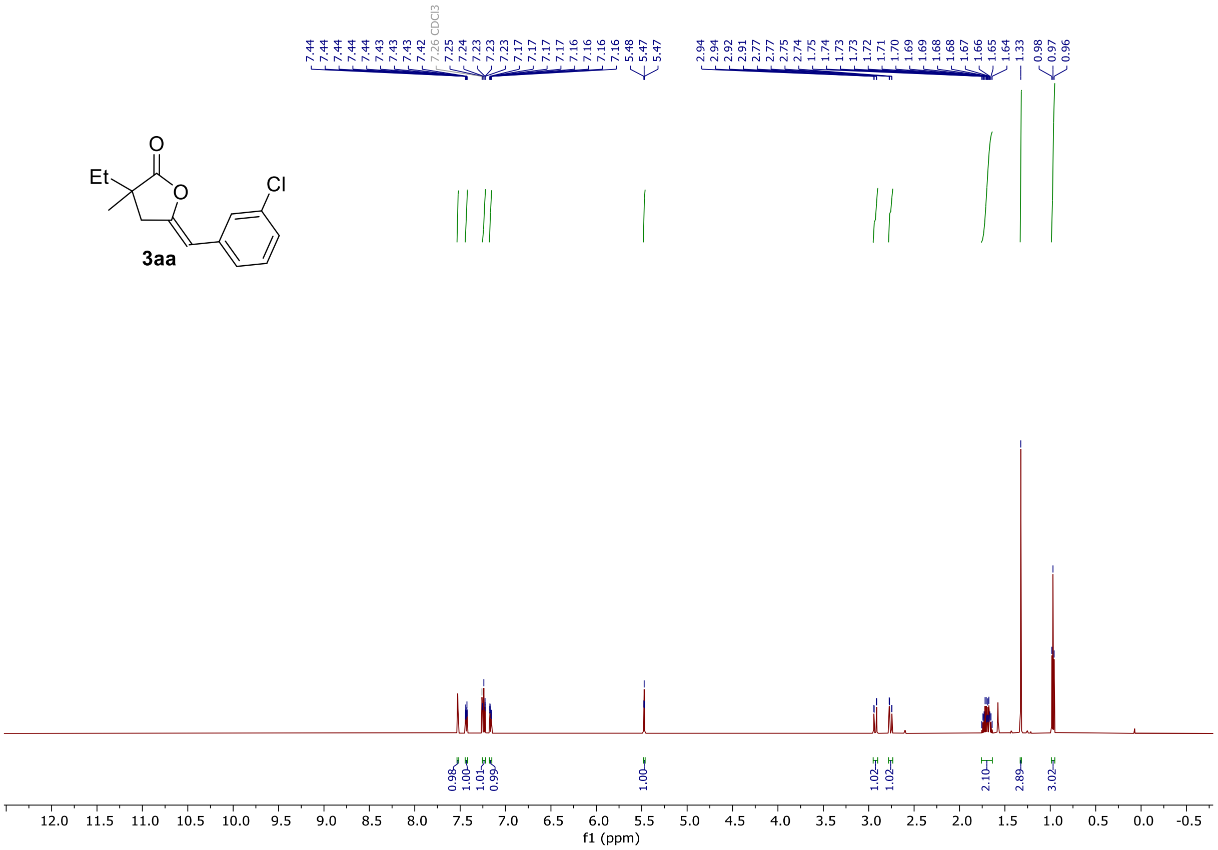

7.44 7.44 7.44 7.44 7.44 7.43 7.43 7.43 7.42 7.26 CDCl3 7.25 7.24 7.23 7.23 7.23 7.23 7.17 7.17 7.17 7.17 7.16 7.16 7.16 7.16 5.48 5.47 5.47
2.94 2.94 2.92 2.91 2.77 2.77 2.75 2.74 1.75 1.75 1.74 1.73 1.73 1.72 1.71 1.70 1.69 1.69 1.68 1.68 1.67 1.66 1.65 1.64 1.33 0.98 0.97 0.96
Et
O
O
Cl
3aa
0.98 1.00 1.01 0.99 1.00 1.02 1.02 2.10 2.89 3.02
12.0 11.5 11.0 10.5 10.0 9.5 9.0 8.5 8.0 7.5 7.0 6.5 6.0 5.5 5.0 4.5 4.0 3.5 3.0 2.5 2.0 1.5 1.0 0.5 0.0 -0.5
f1 (ppm)

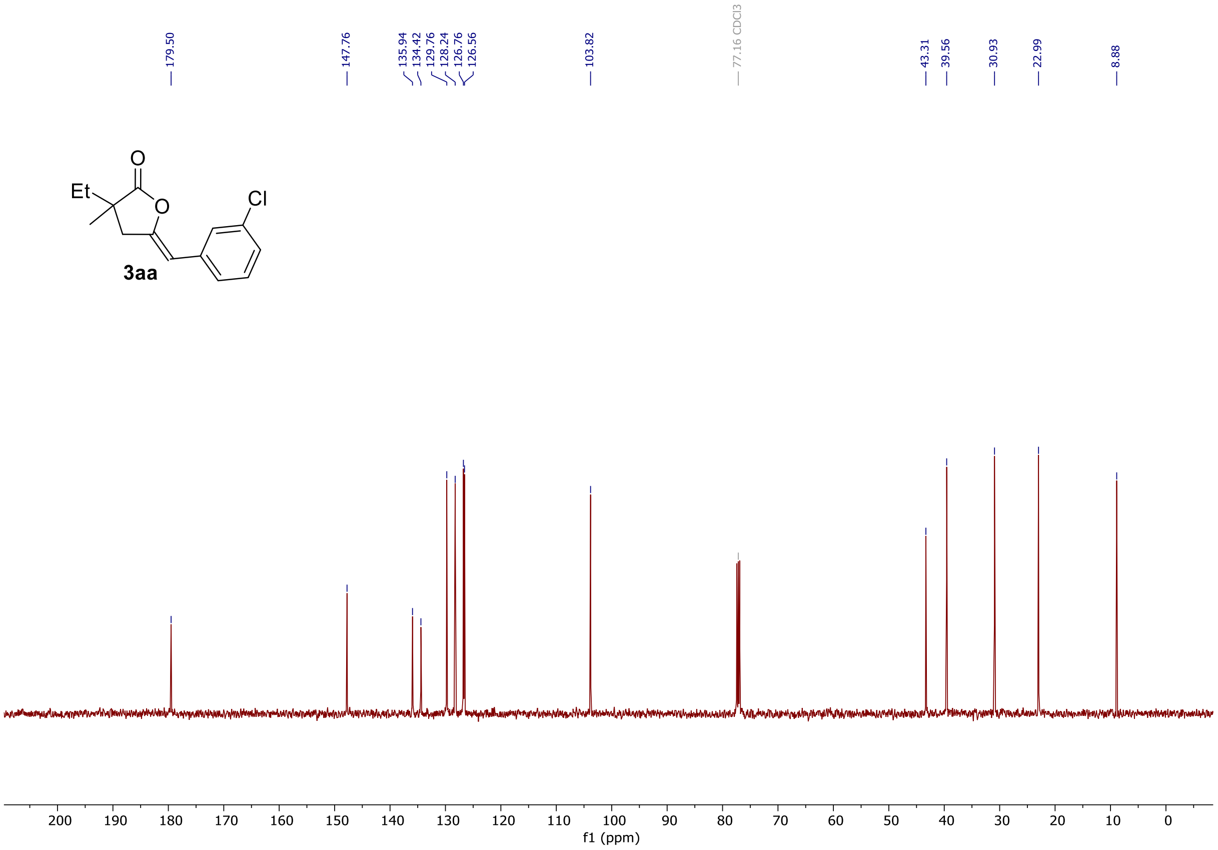

179.50 147.76 135.94 134.42 129.76 128.24 126.76 126.56 103.82 77.16 CDCl3 43.31 39.56 30.93 22.99 8.88
Et
O
O
Cl
3aa
200 190 180 170 160 150 140 130 120 110 100 90 80 70 60 50 40 30 20 10 0
f1 (ppm)

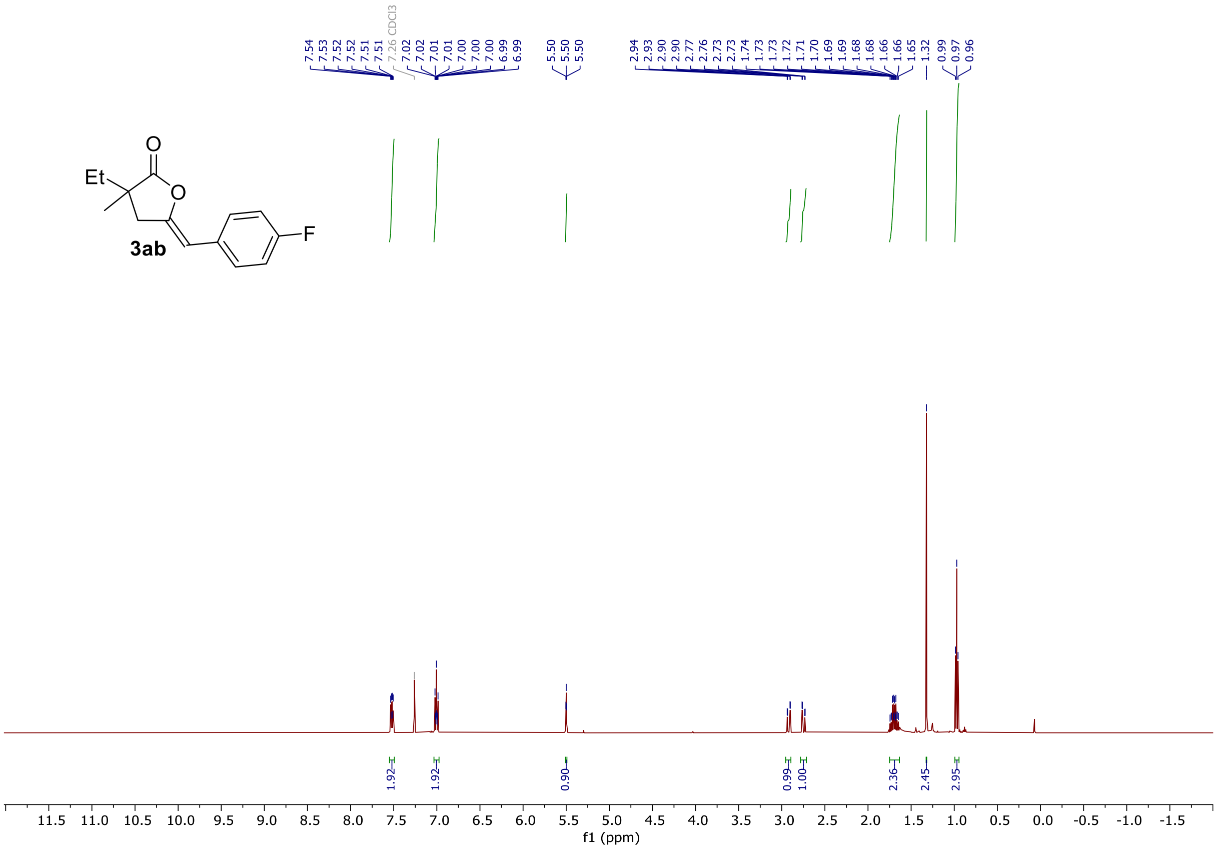
7.54
7.53
7.52
7.52
7.51
7.51
7.26 CDCl3
7.02
7.02
7.01
7.01
7.00
7.00
7.00
6.99
6.99
5.50
5.50
5.50
2.94
2.93
2.90
2.90
2.77
2.76
2.73
2.73
1.74
1.73
1.73
1.72
1.71
1.70
1.69
1.69
1.68
1.68
1.66
1.66
1.65
1.32
0.99
0.97
0.96
Et
O
O
F
3ab
1.92
1.92
0.90
0.99
1.00
2.36
2.45
2.95
f1 (ppm)

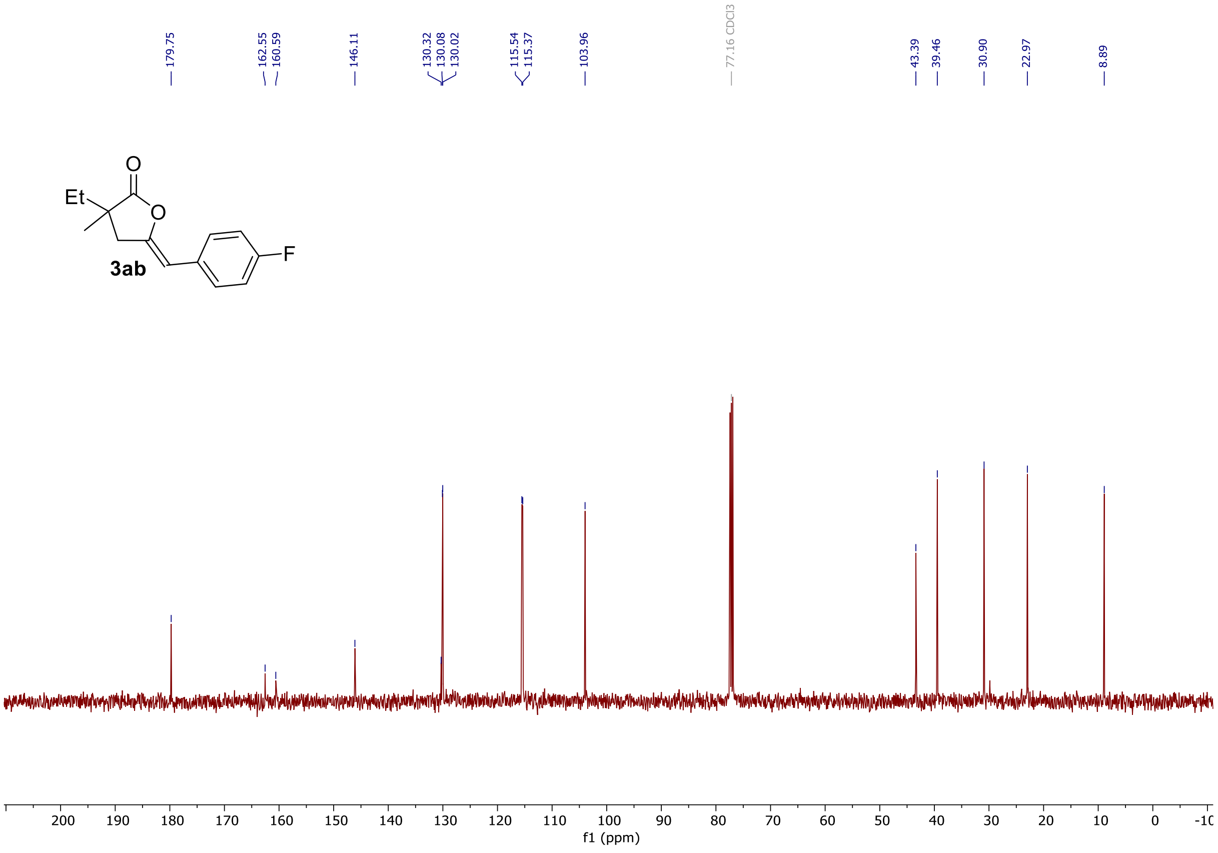
179.75
162.55
160.59
146.11
130.32
130.08
130.02
115.54
115.37
103.96
77.16 CDCl3
43.39
39.46
30.90
22.97
8.89
Et
O
O
F
3ab
f1 (ppm)

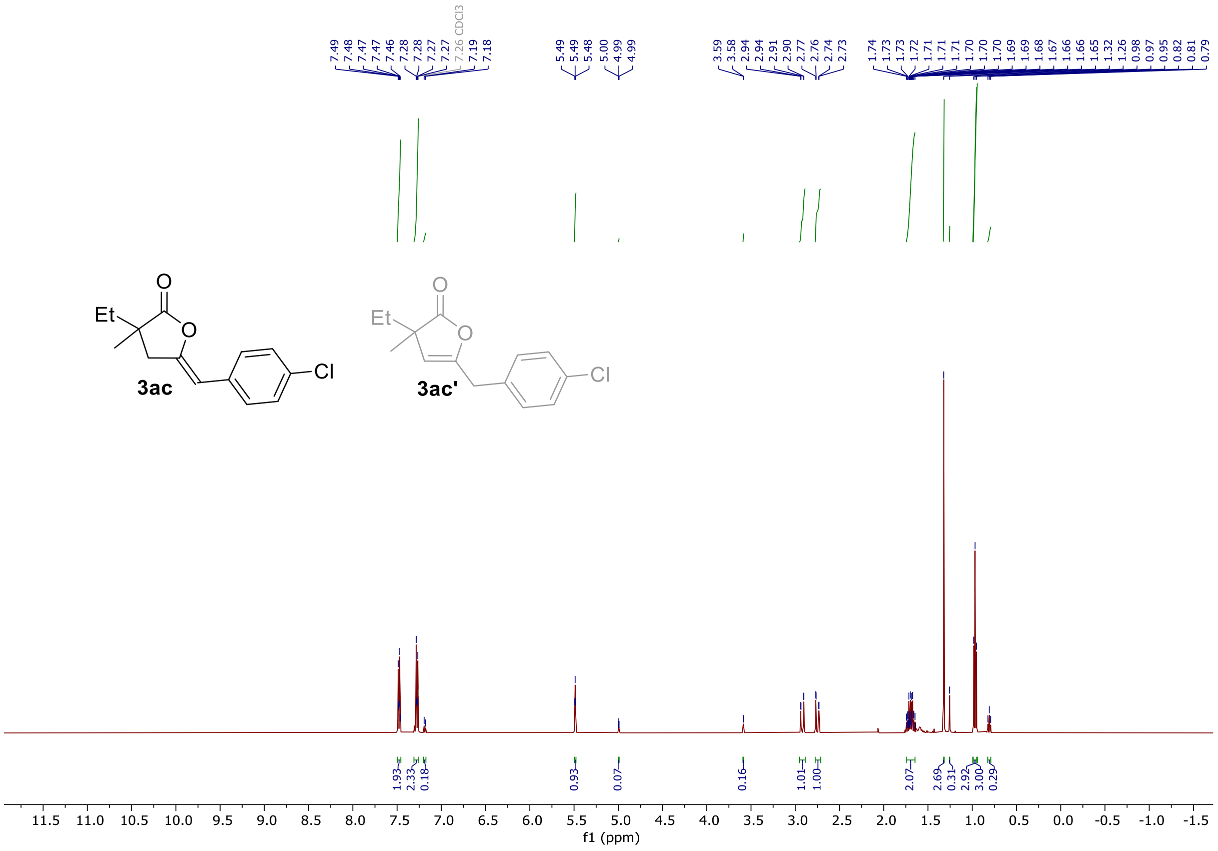
7.49 7.48 7.47 7.47 7.46 7.28 7.28 7.27 7.27 7.26 CDCl3 7.19 7.18
5.49 5.49 5.48 5.00 4.99 4.99
3.59 3.58 2.94 2.94 2.91 2.90 2.77 2.76 2.74 2.73
1.74 1.73 1.73 1.72 1.71 1.71 1.71 1.70 1.70 1.70 1.69 1.69 1.68 1.67 1.66 1.66 1.65 1.32 1.26 0.98 0.97 0.95 0.82 0.81 0.79
Et
O
Cl
3ac
3ac'
1.93 2.33 0.18 0.93 0.07 0.16 1.01 1.00 2.07 2.69 0.31 2.92 3.00 0.29
11.5 11.0 10.5 10.0 9.5 9.0 8.5 8.0 7.5 7.0 6.5 6.0 5.5 5.0 4.5 4.0 3.5 3.0 2.5 2.0 1.5 1.0 0.5 0.0 -0.5 -1.0 -1.5
f1 (ppm)

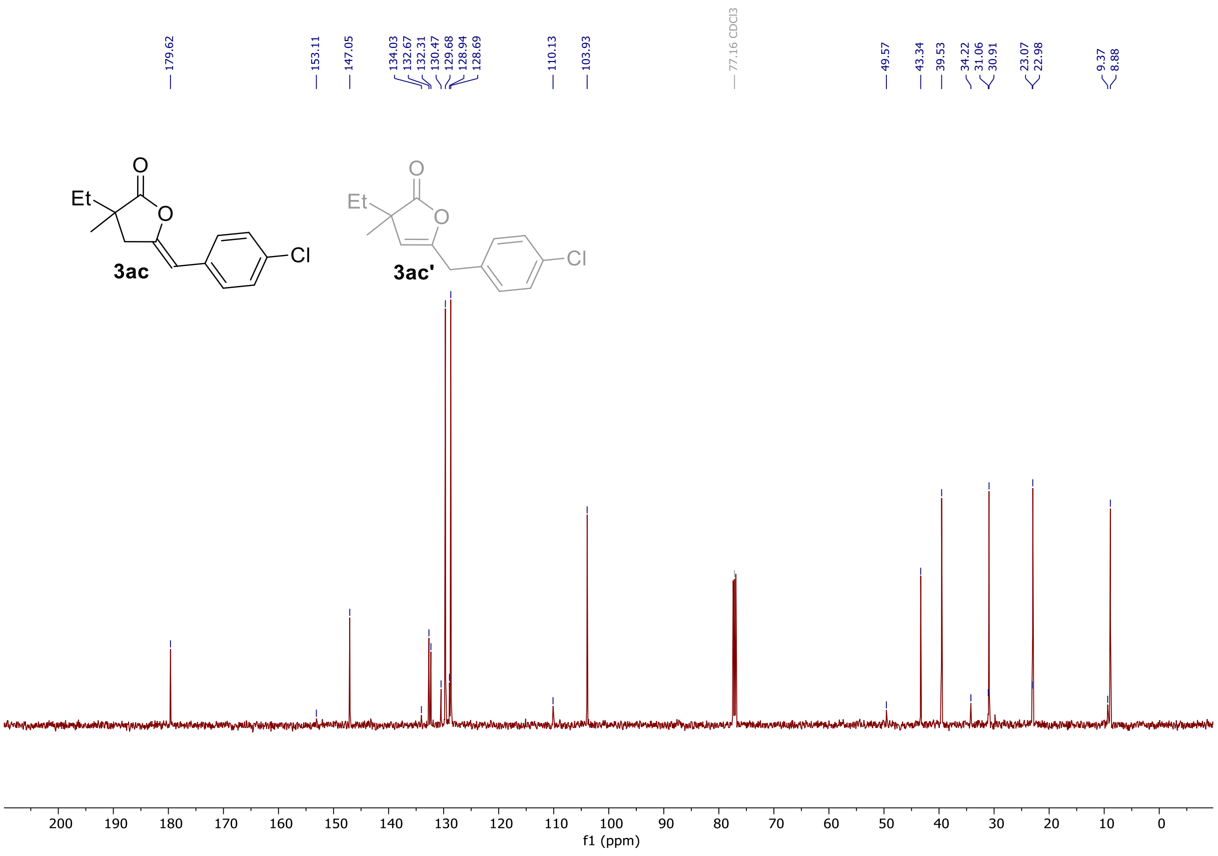
179.62
153.11
147.05
134.03 132.67 132.31 130.47 129.68 128.94 128.69
110.13
103.93
77.16 CDCl3
49.57
43.34
39.53
34.22 31.06 30.91
23.07 22.98
9.37 8.88
Et
O
Cl
3ac
3ac'
200 190 180 170 160 150 140 130 120 110 100 90 80 70 60 50 40 30 20 10 0
f1 (ppm)

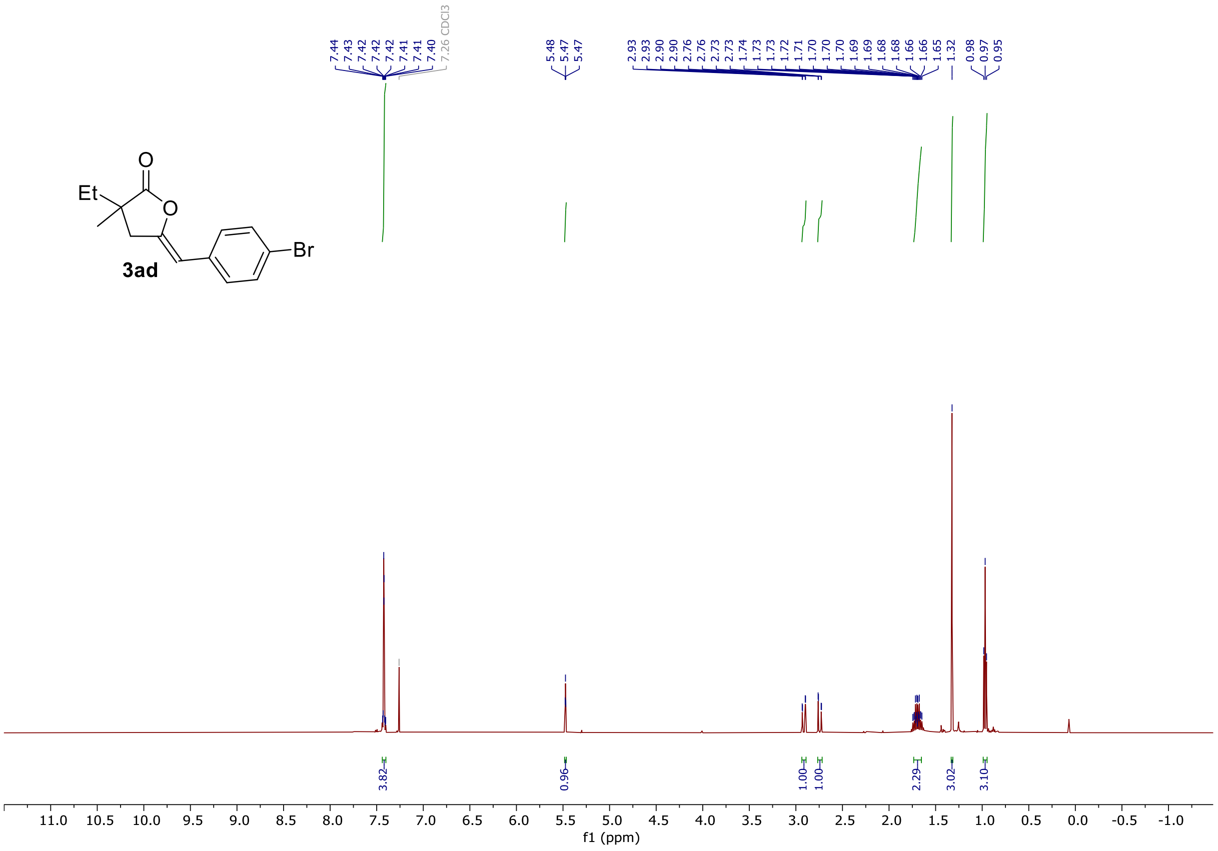

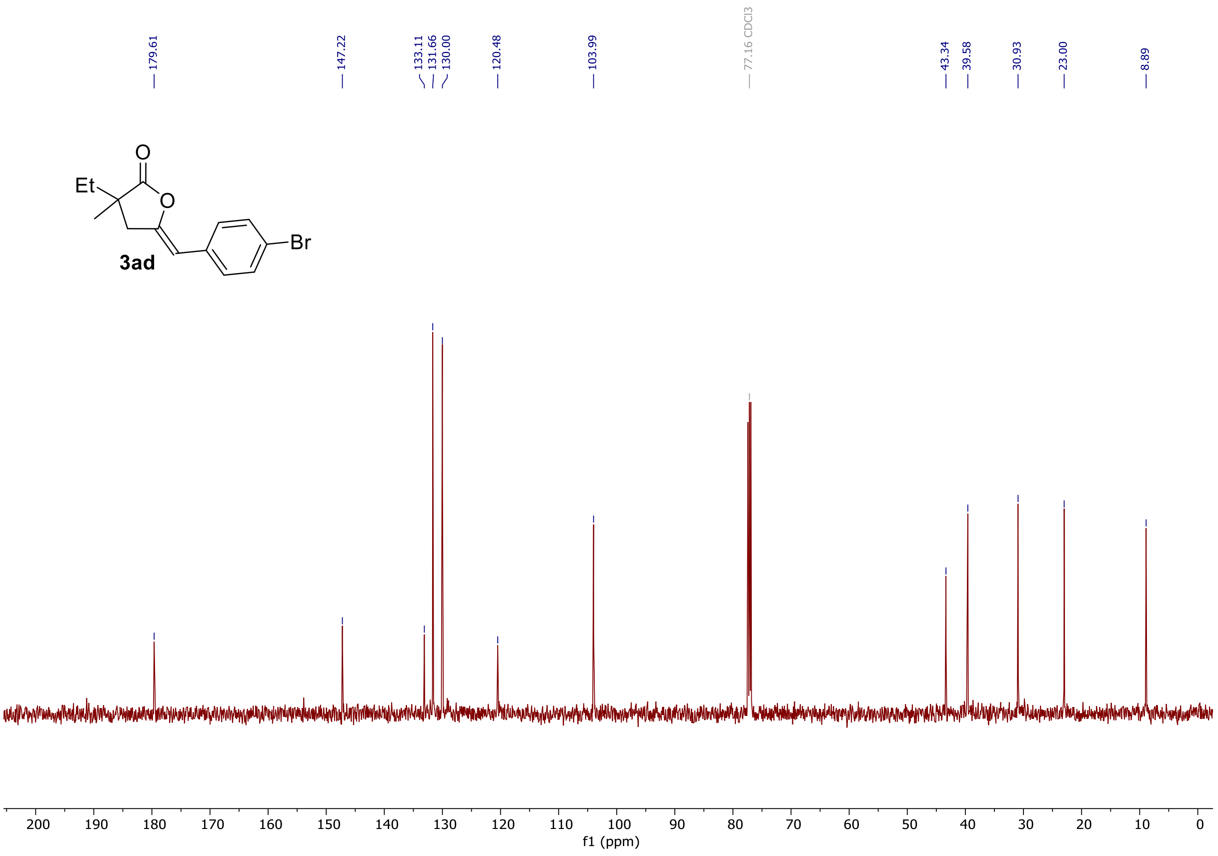

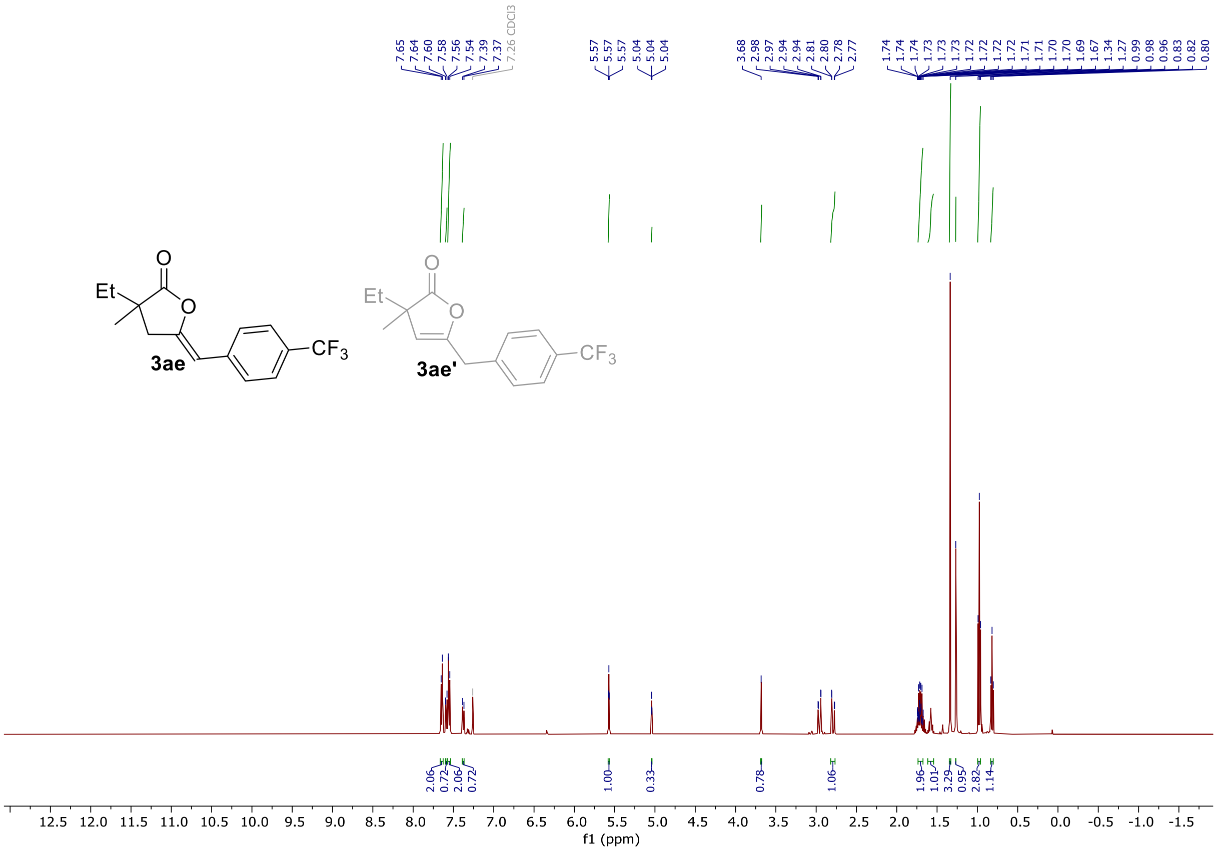
Et
O
CF3
3ae
3ae'
7.26 CDCl3
f1 (ppm)

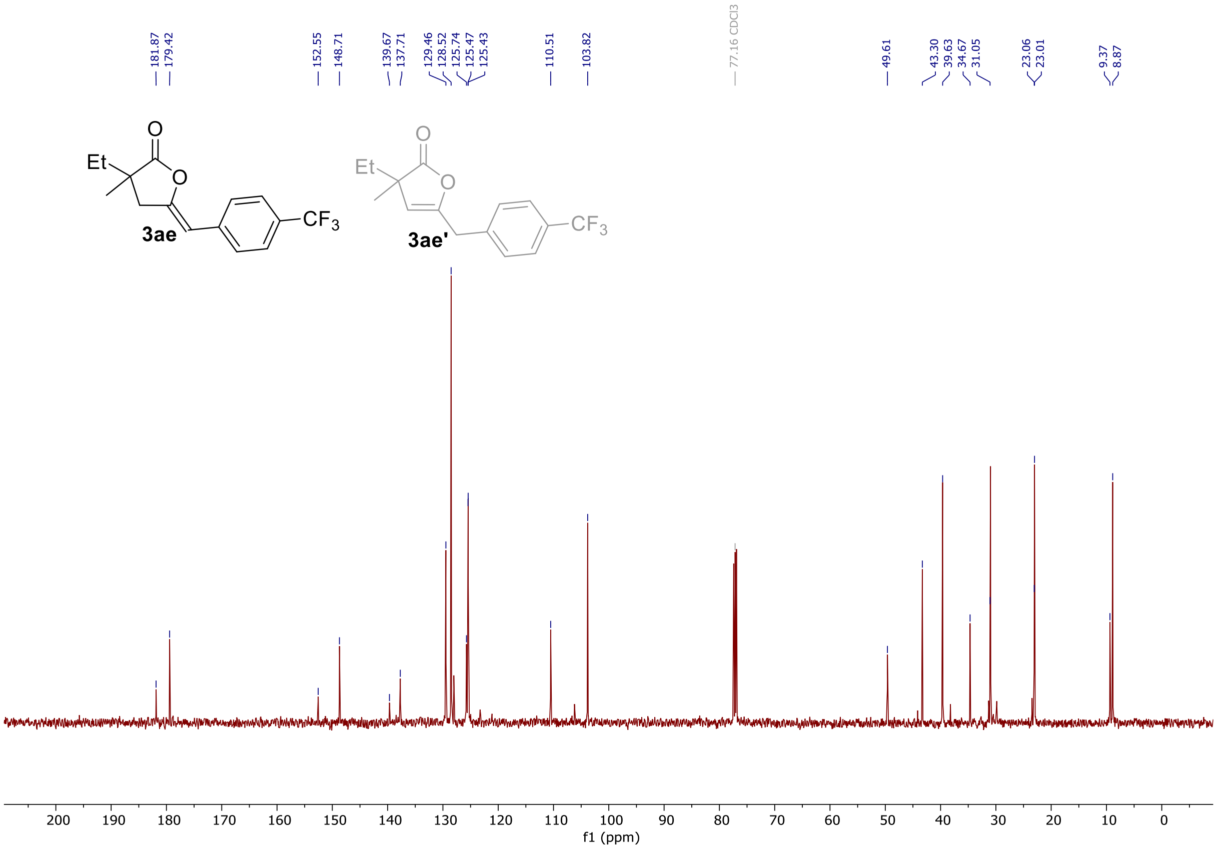
Et
O
CF3
3ae
3ae'
77.16 CDCl3
f1 (ppm)

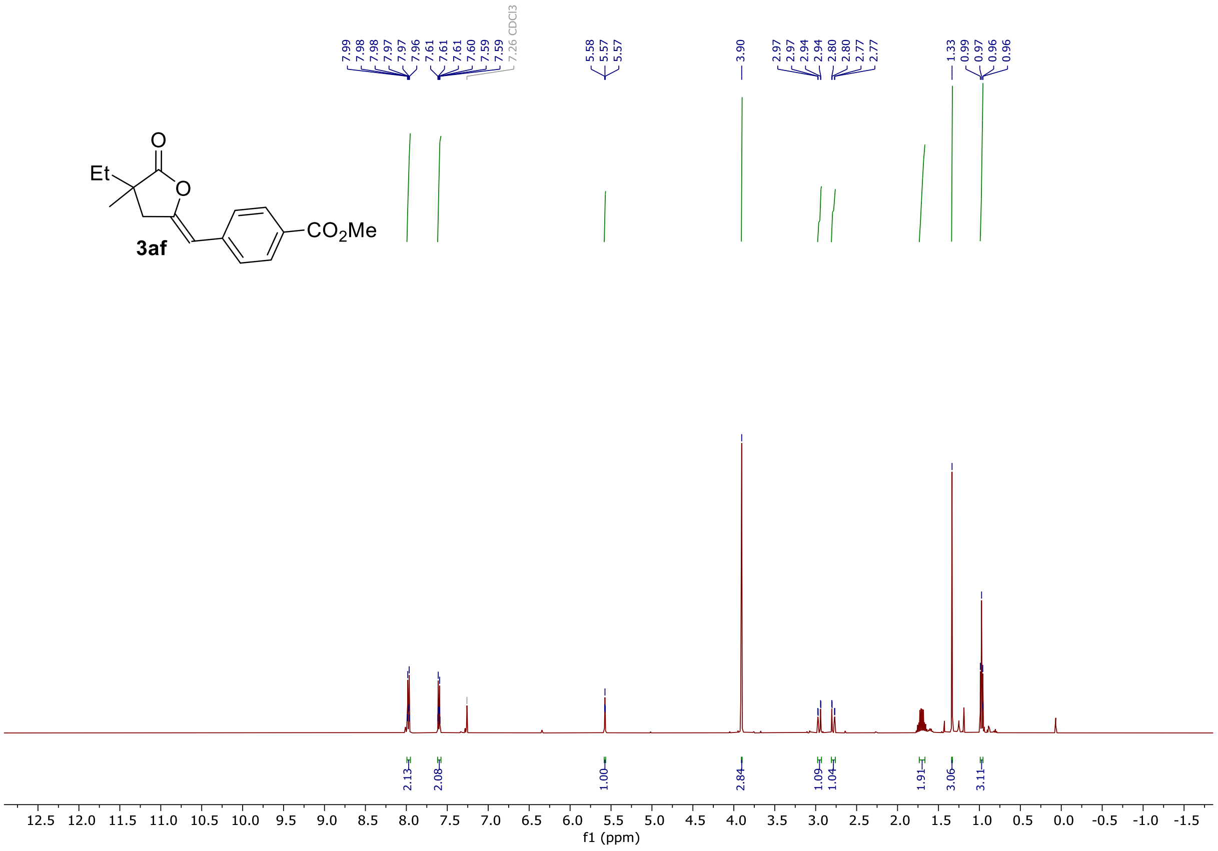
Et
O
O
CO2Me
3af
7.26 CDCl3
f1 (ppm)

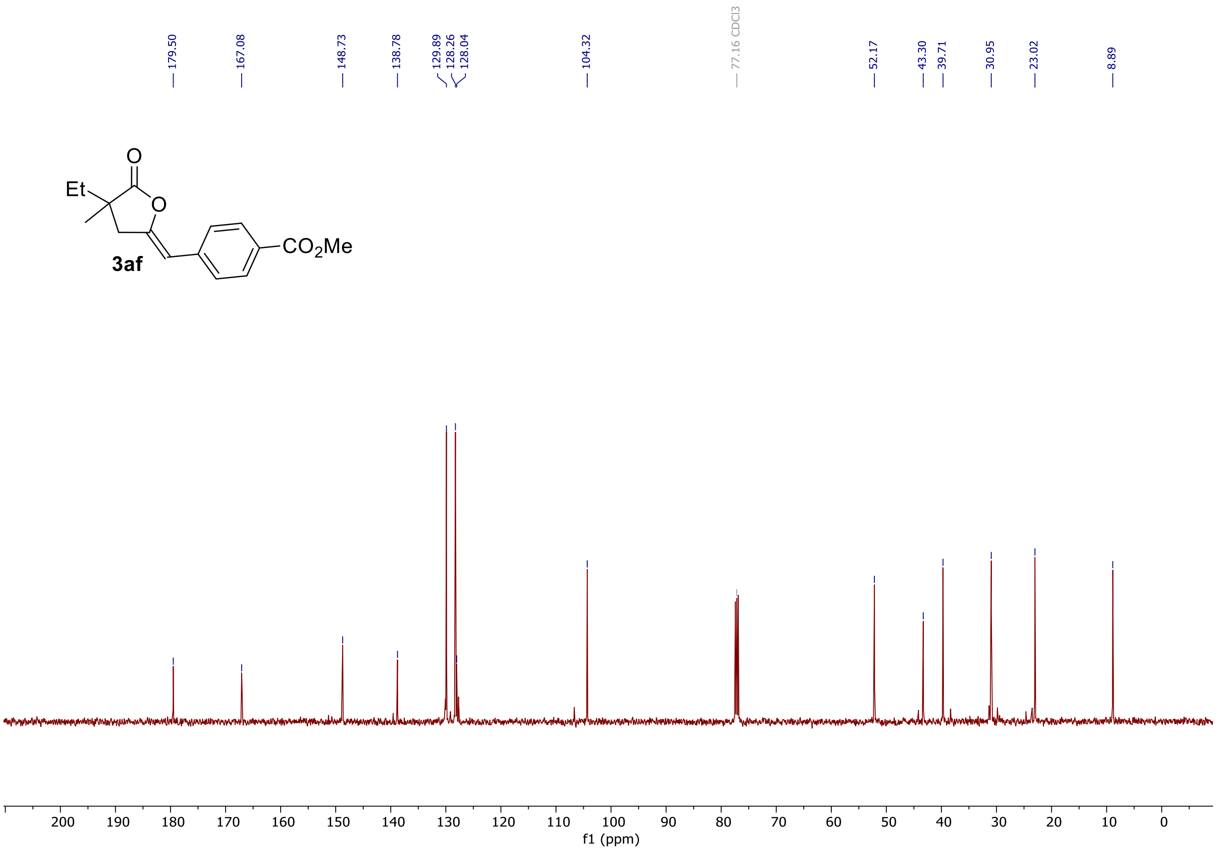
Et
O
O
CO2Me
3af
179.50
167.08
148.73
138.78
129.89
128.26
128.04
104.32
77.16 CDCl3
52.17
43.30
39.71
30.95
23.02
8.89
f1 (ppm)

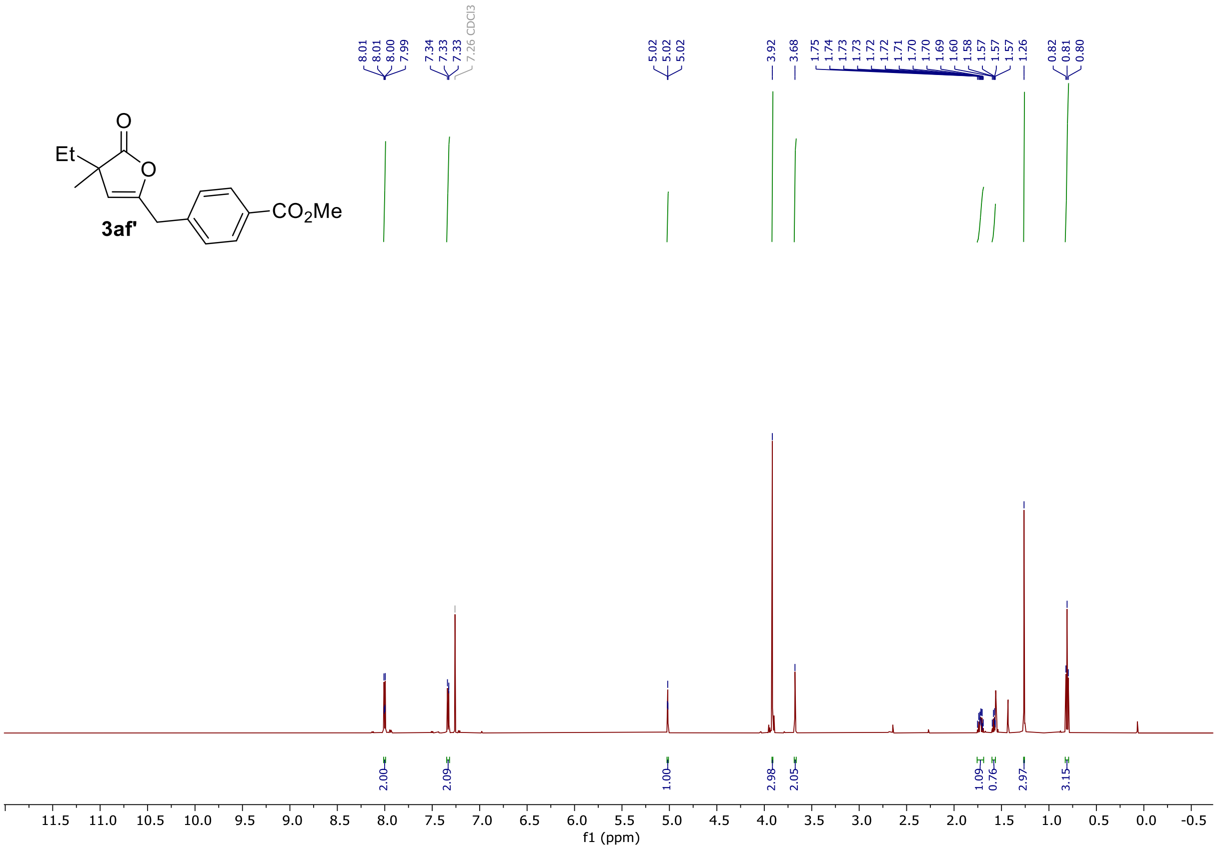
8.01
8.01
8.00
7.99
7.34
7.33
7.33
7.26 CDCl3
5.02
5.02
5.02
3.92
3.68
1.75
1.74
1.73
1.73
1.72
1.72
1.71
1.70
1.70
1.69
1.60
1.58
1.57
1.57
1.57
1.26
0.82
0.81
0.80
Et
O
O
CO2Me
3af'
2.00
2.09
1.00
2.98
2.05
1.09
0.76
2.97
3.15
11.5 11.0 10.5 10.0 9.5 9.0 8.5 8.0 7.5 7.0 6.5 6.0 5.5 5.0 4.5 4.0 3.5 3.0 2.5 2.0 1.5 1.0 0.5 0.0 -0.5
f1 (ppm)

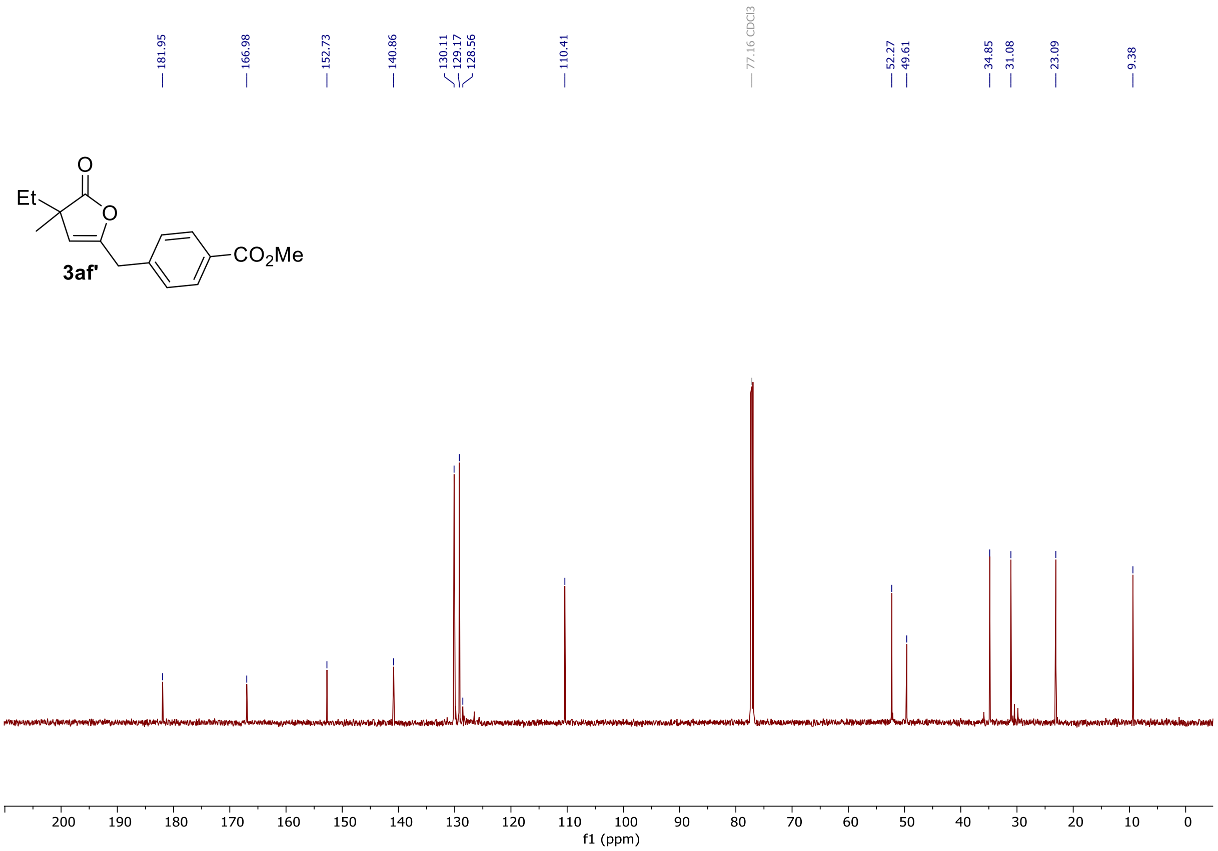
181.95
166.98
152.73
140.86
130.11
129.17
128.56
110.41
77.16 CDCl3
52.27
49.61
34.85
31.08
23.09
9.38
Et
O
O
CO2Me
3af'
200 190 180 170 160 150 140 130 120 110 100 90 80 70 60 50 40 30 20 10 0
f1 (ppm)

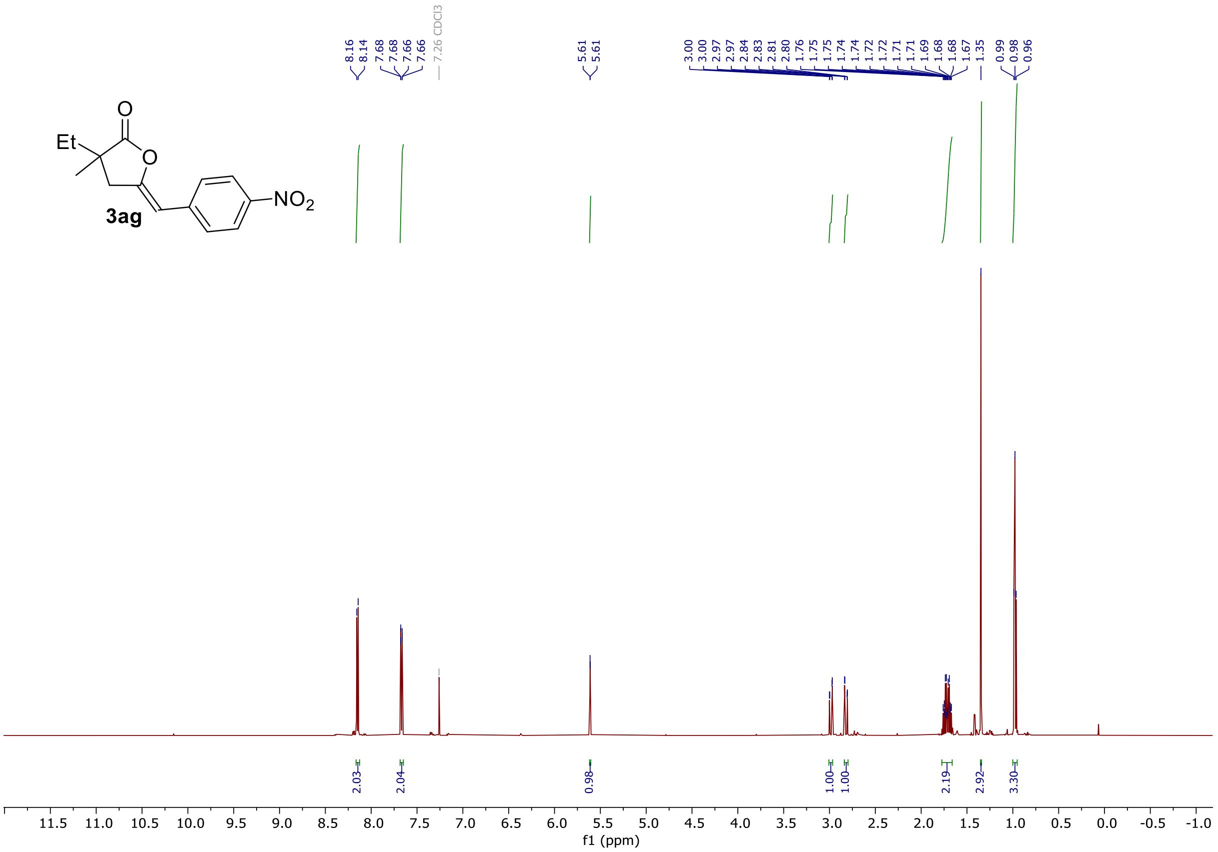
8.16
8.14
7.68
7.68
7.66
7.66
7.26 CDCl3
5.61
5.61
3.00
3.00
2.97
2.97
2.84
2.83
2.81
2.80
1.76
1.75
1.75
1.74
1.74
1.72
1.72
1.71
1.71
1.69
1.68
1.68
1.67
1.35
0.99
0.98
0.96
Et
O
O
NO2
3ag
2.03
2.04
0.98
1.00
1.00
2.19
2.92
3.30
f1 (ppm)

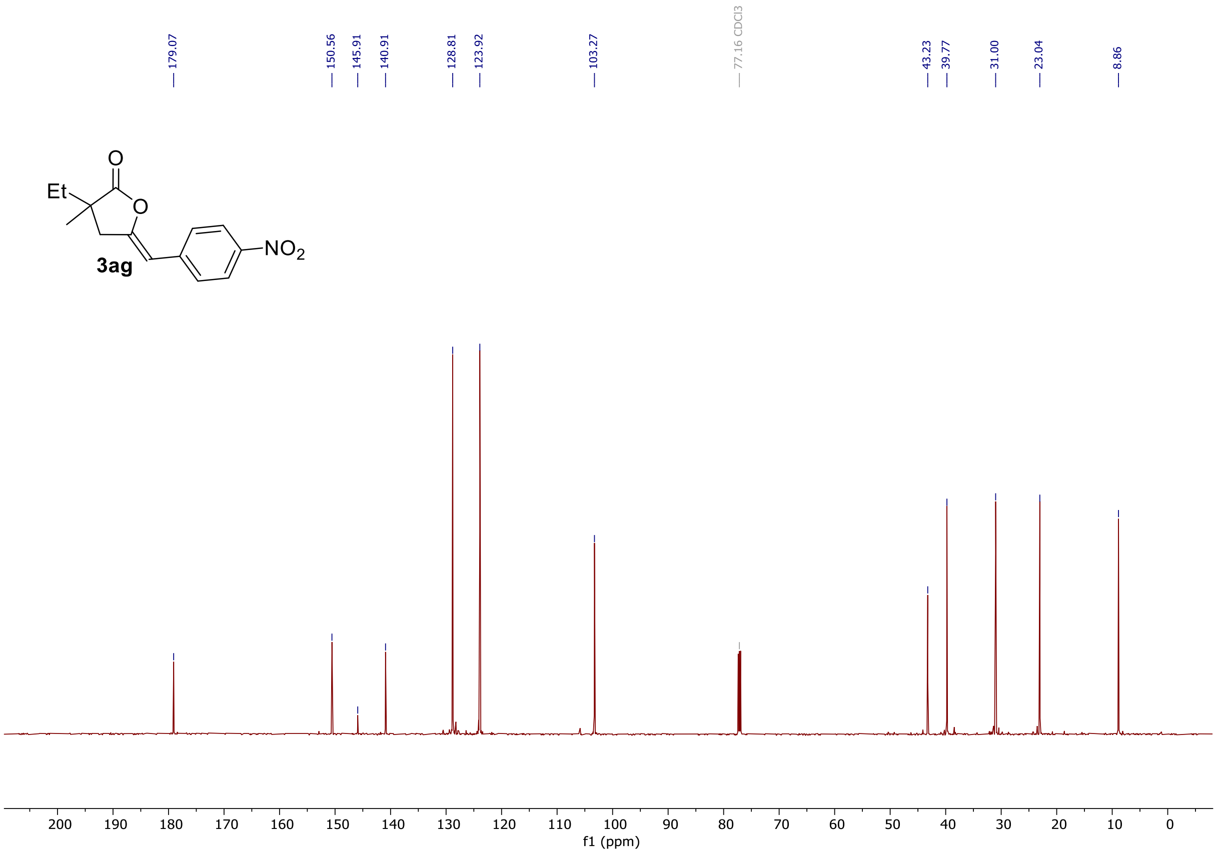
179.07
150.56
145.91
140.91
128.81
123.92
103.27
77.16 CDCl3
43.23
39.77
31.00
23.04
8.86
Et
O
O
NO2
3ag
f1 (ppm)

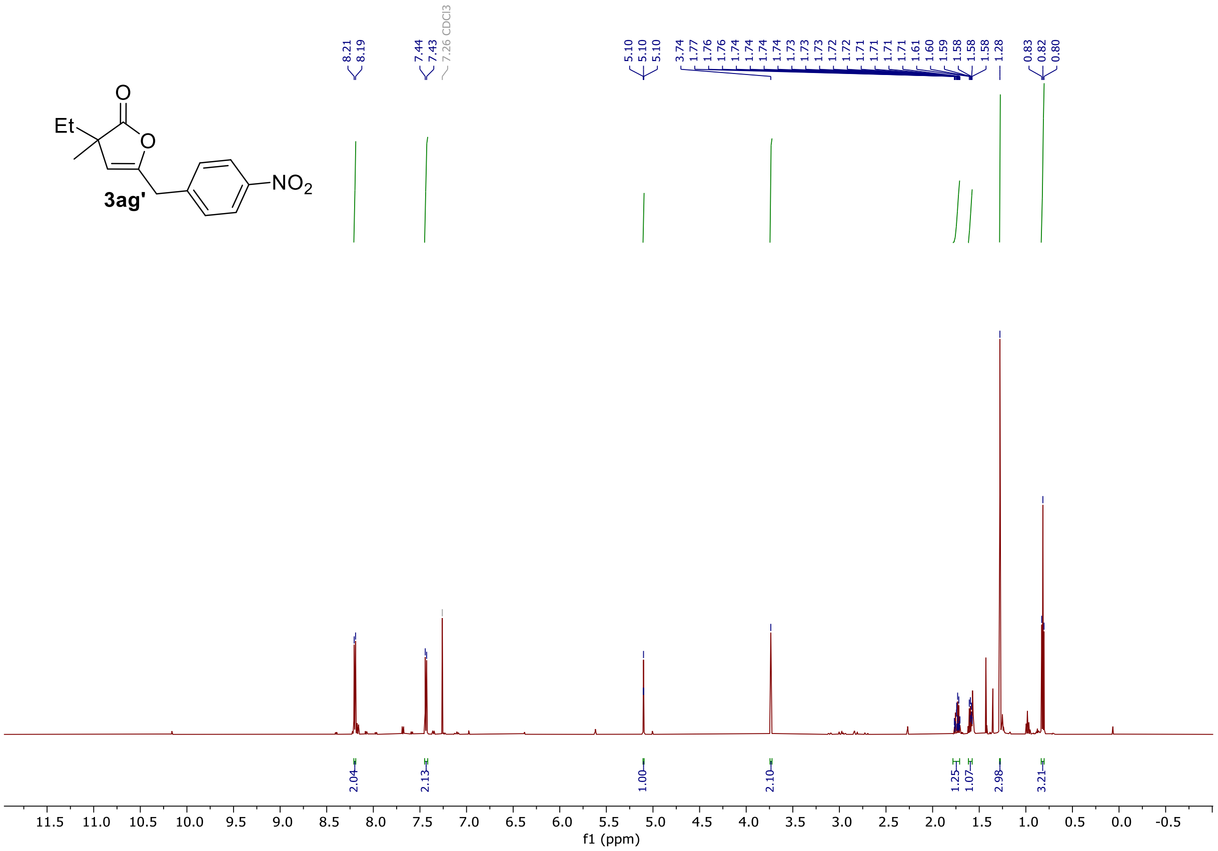
3ag'
f1 (ppm)

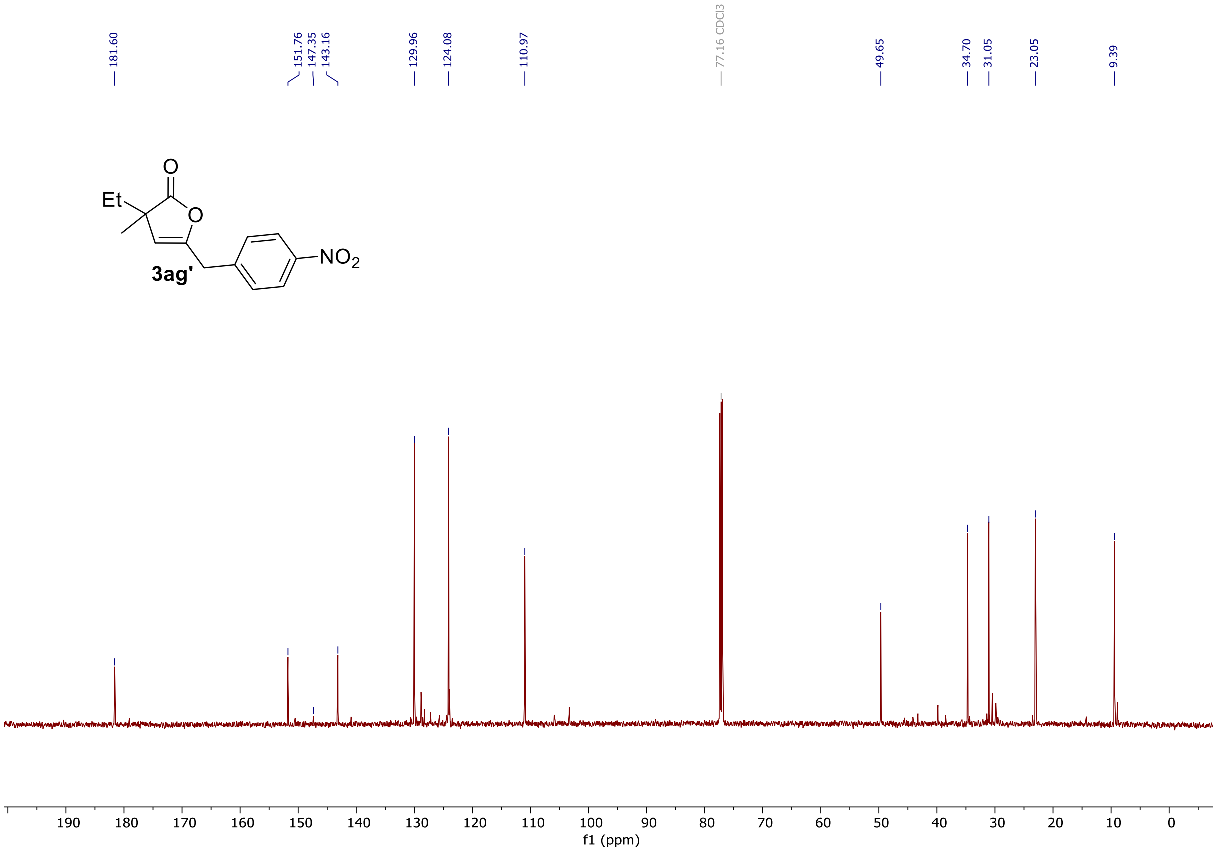
3ag'
f1 (ppm)

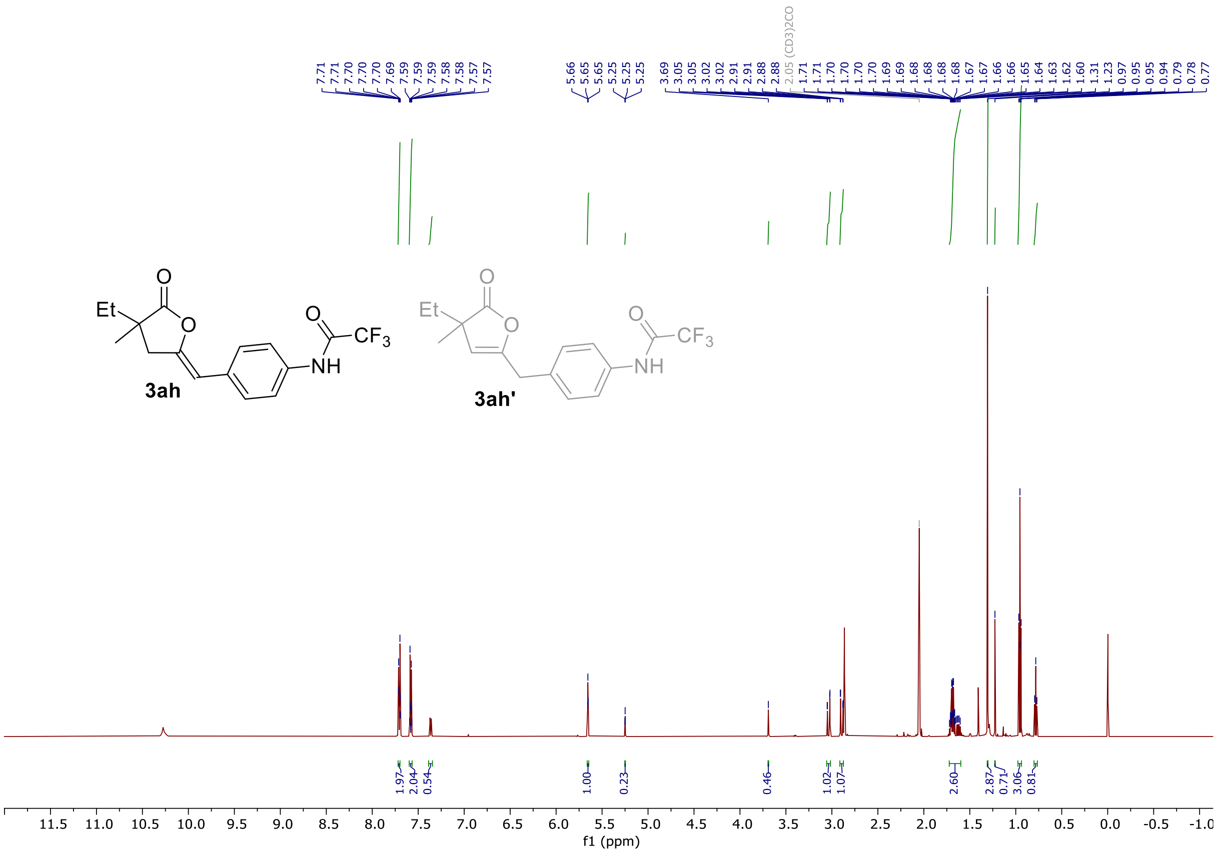
3ah
3ah'
f1 (ppm)

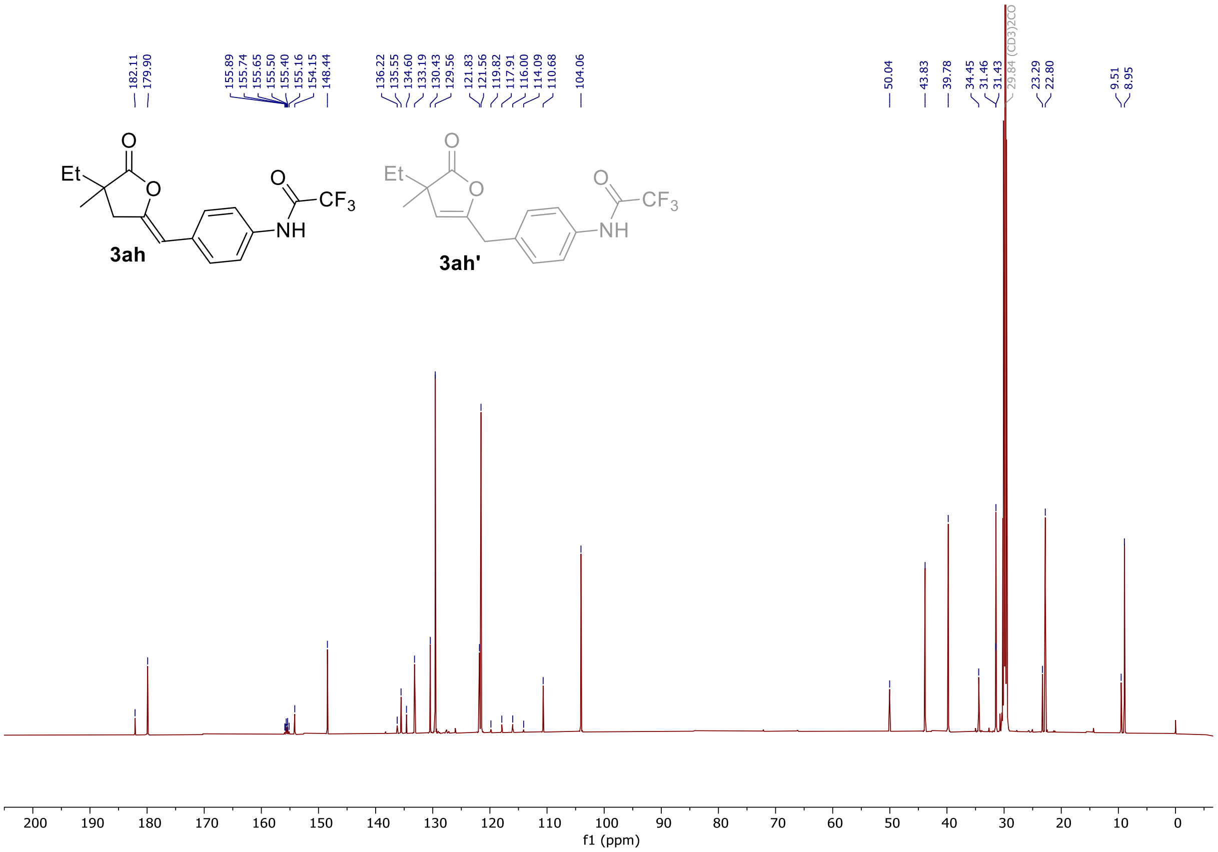
3ah
3ah'
f1 (ppm)

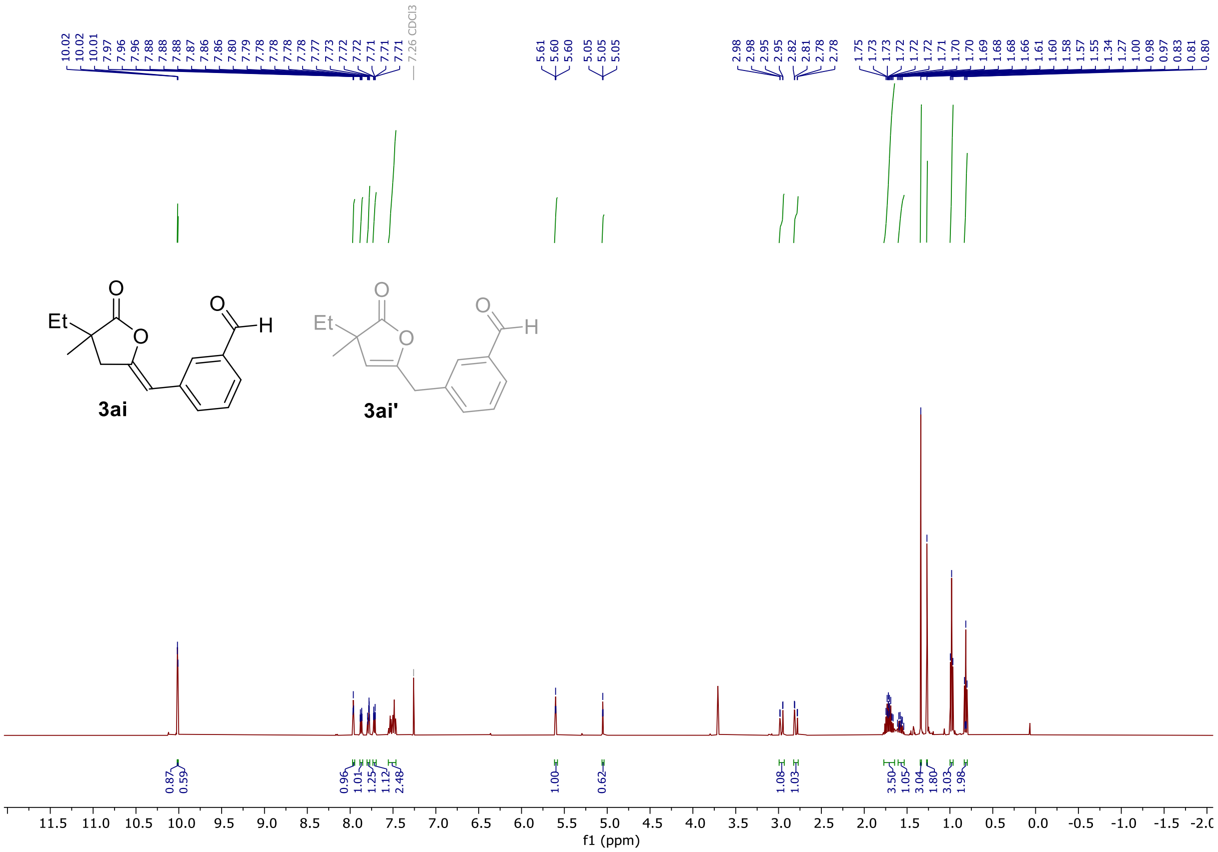
10.02 10.02 10.01 7.97 7.96 7.96 7.88 7.88 7.88 7.87 7.86 7.86 7.80 7.79 7.78 7.78 7.78 7.78 7.77 7.73 7.72 7.72 7.71 7.71 7.71
7.26 CDCl3
5.61 5.60 5.60 5.05 5.05 5.05
2.98 2.98 2.95 2.95 2.82 2.81 2.78 2.78
1.75 1.73 1.73 1.72 1.72 1.72 1.71 1.70 1.70 1.69 1.68 1.68 1.66 1.61 1.60 1.58 1.57 1.55 1.34 1.27 1.00 0.98 0.97 0.83 0.81 0.80
3ai
3ai'
0.87 0.59
0.96 1.01 1.25 1.12 2.48
1.00
0.62
1.08 1.03
3.50 1.05 3.04 1.80 3.03 1.98
f1 (ppm)

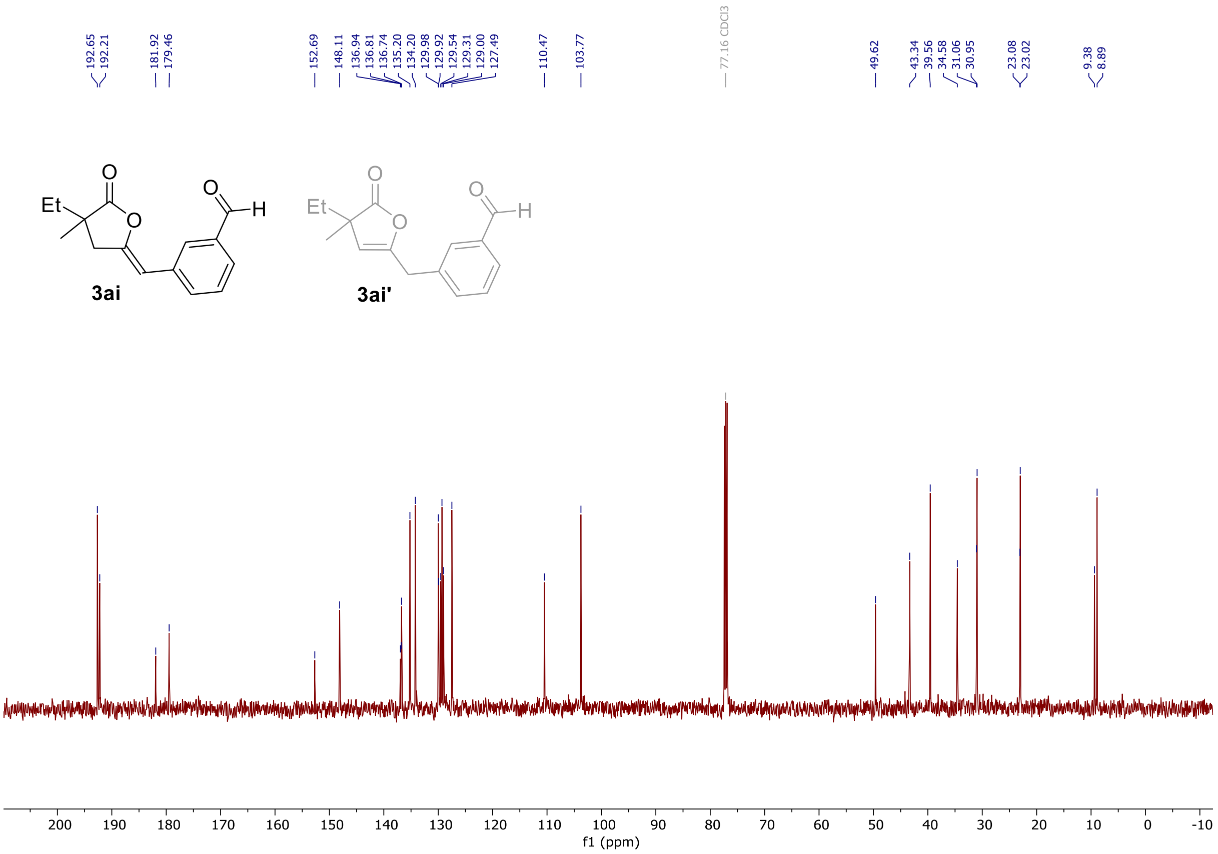
192.65 192.21
181.92 179.46
152.69 148.11 136.94 136.81 136.74 135.20 134.20 129.98 129.92 129.54 129.31 129.00 127.49
110.47
103.77
77.16 CDCl3
49.62
43.34 39.56 34.58 31.06 30.95
23.08 23.02
9.38 8.89
3ai
3ai'
f1 (ppm)

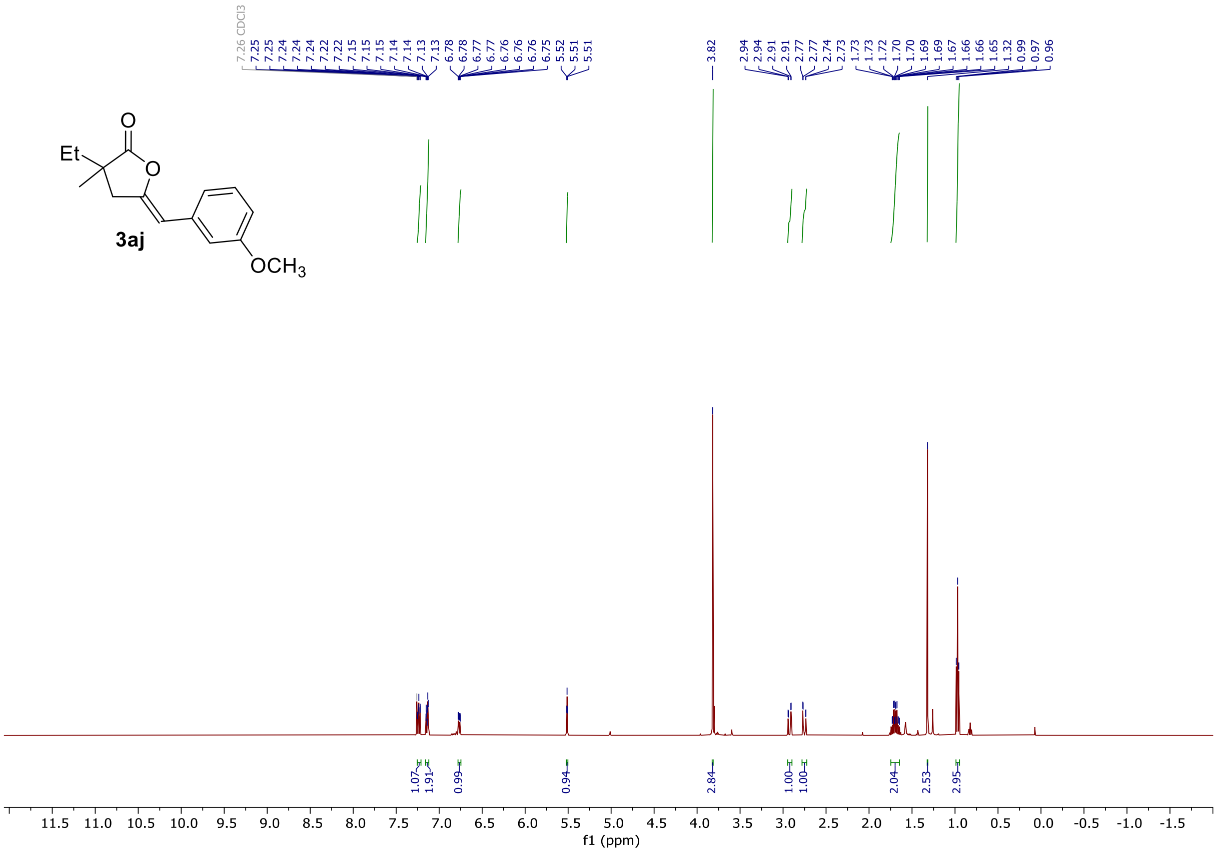
7.26 CDCl3
7.25 7.25 7.24 7.24 7.24 7.22 7.22 7.15 7.15 7.15 7.14 7.13 7.13 6.78 6.78 6.77 6.77 6.76 6.76 6.75 5.52 5.51 5.51
3.82
2.94 2.94 2.91 2.91 2.77 2.77 2.74 2.73 1.73 1.73 1.72 1.70 1.70 1.69 1.69 1.67 1.66 1.66 1.65 1.32 0.99 0.97 0.96
Et
O
OCH3
3aj
1.07 1.91 0.99 0.94 2.84 1.00 1.00 2.04 2.53 2.95
11.5 11.0 10.5 10.0 9.5 9.0 8.5 8.0 7.5 7.0 6.5 6.0 5.5 5.0 4.5 4.0 3.5 3.0 2.5 2.0 1.5 1.0 0.5 0.0 -0.5 -1.0 -1.5
f1 (ppm)

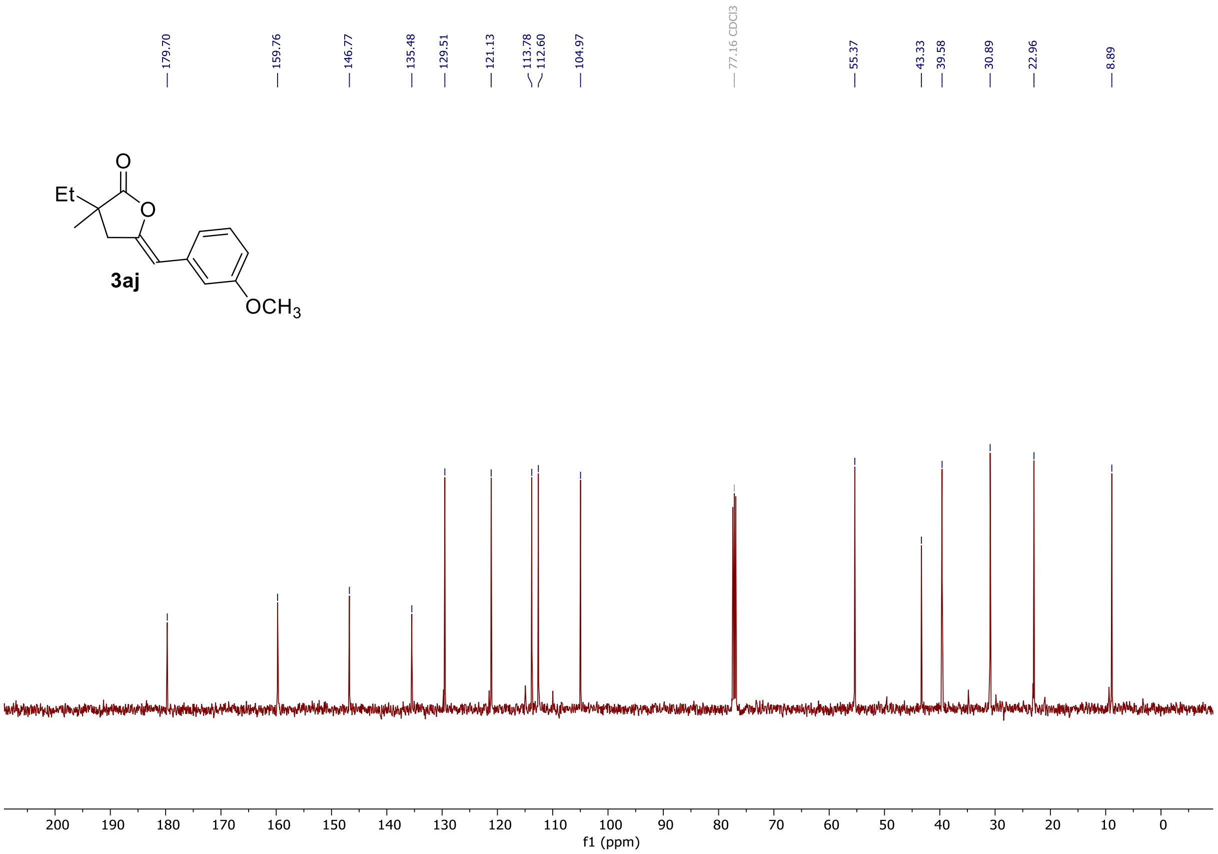
179.70
159.76
146.77
135.48
129.51
121.13
113.78
112.60
104.97
77.16 CDCl3
55.37
43.33
39.58
30.89
22.96
8.89
Et
O
OCH3
3aj
200 190 180 170 160 150 140 130 120 110 100 90 80 70 60 50 40 30 20 10 0
f1 (ppm)

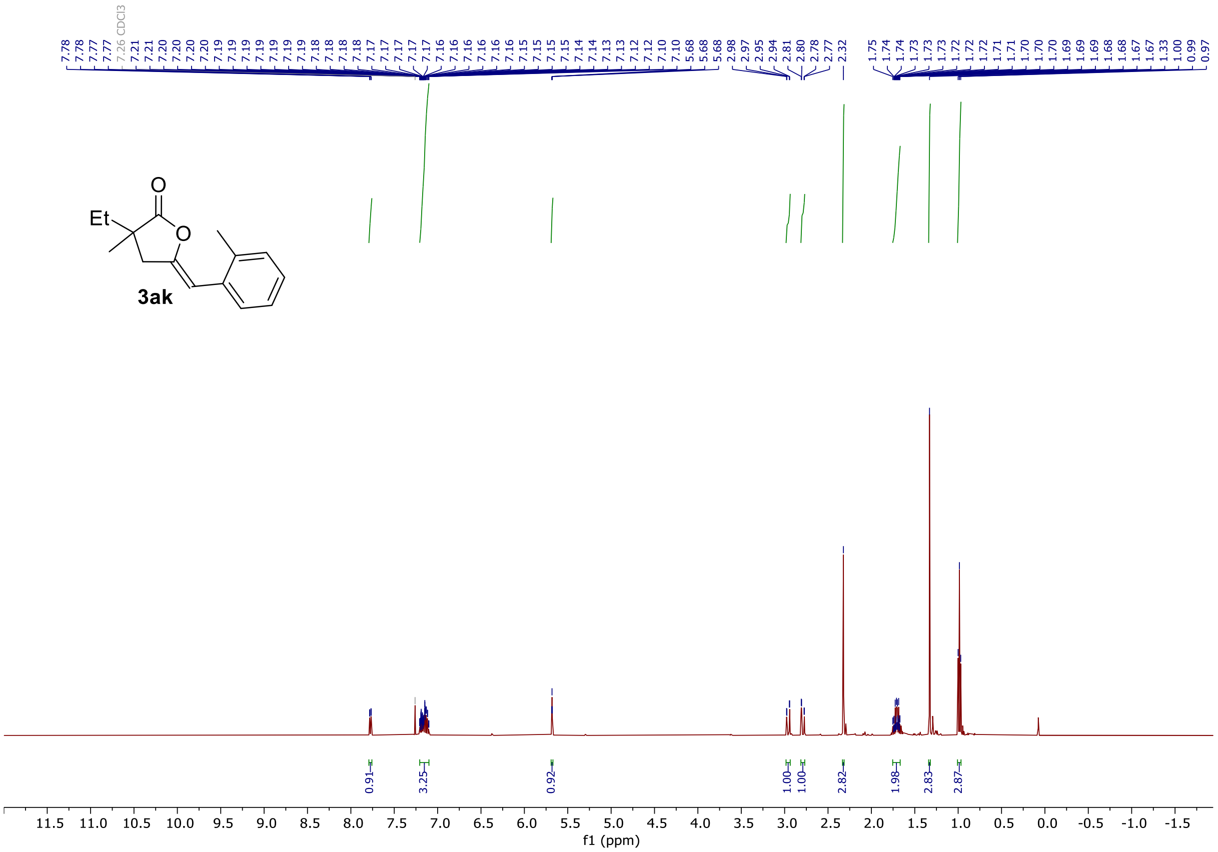
7.78 7.78 7.77 7.26 CDCl3 7.21 7.21 7.20 7.20 7.20 7.20 7.19 7.19 7.19 7.19 7.19 7.19 7.18 7.18 7.18 7.17 7.17 7.17 7.17 7.16 7.16 7.16 7.16 7.16 7.15 7.15 7.15 7.14 7.13 7.13 7.12 7.12 7.10 7.10 5.68 5.68 2.98 2.97 2.95 2.94 2.81 2.80 2.78 2.77 2.32 1.75 1.74 1.74 1.73 1.73 1.73 1.72 1.72 1.72 1.71 1.71 1.70 1.70 1.70 1.69 1.69 1.69 1.68 1.68 1.67 1.67 1.33 1.00 0.99 0.97
Et
O
O
3ak
0.91 3.25 0.92 1.00 1.00 2.82 1.98 2.83 2.87
11.5 11.0 10.5 10.0 9.5 9.0 8.5 8.0 7.5 7.0 6.5 6.0 5.5 5.0 4.5 4.0 3.5 3.0 2.5 2.0 1.5 1.0 0.5 0.0 -0.5 -1.0 -1.5
f1 (ppm)

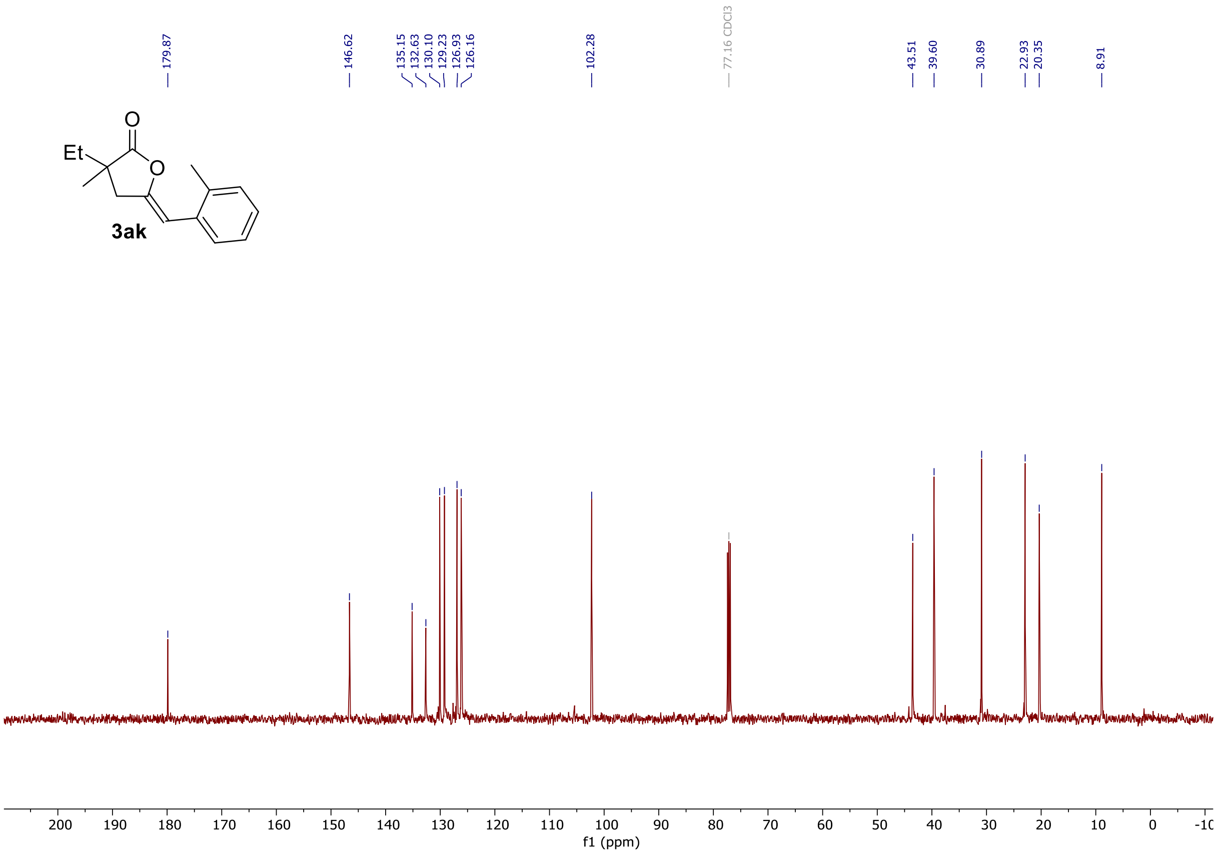
179.87 146.62 135.15 132.63 130.10 129.23 126.93 126.16 102.28 77.16 CDCl3 43.51 39.60 30.89 22.93 20.35 8.91
Et
O
O
3ak
200 190 180 170 160 150 140 130 120 110 100 90 80 70 60 50 40 30 20 10 0 -10
f1 (ppm)

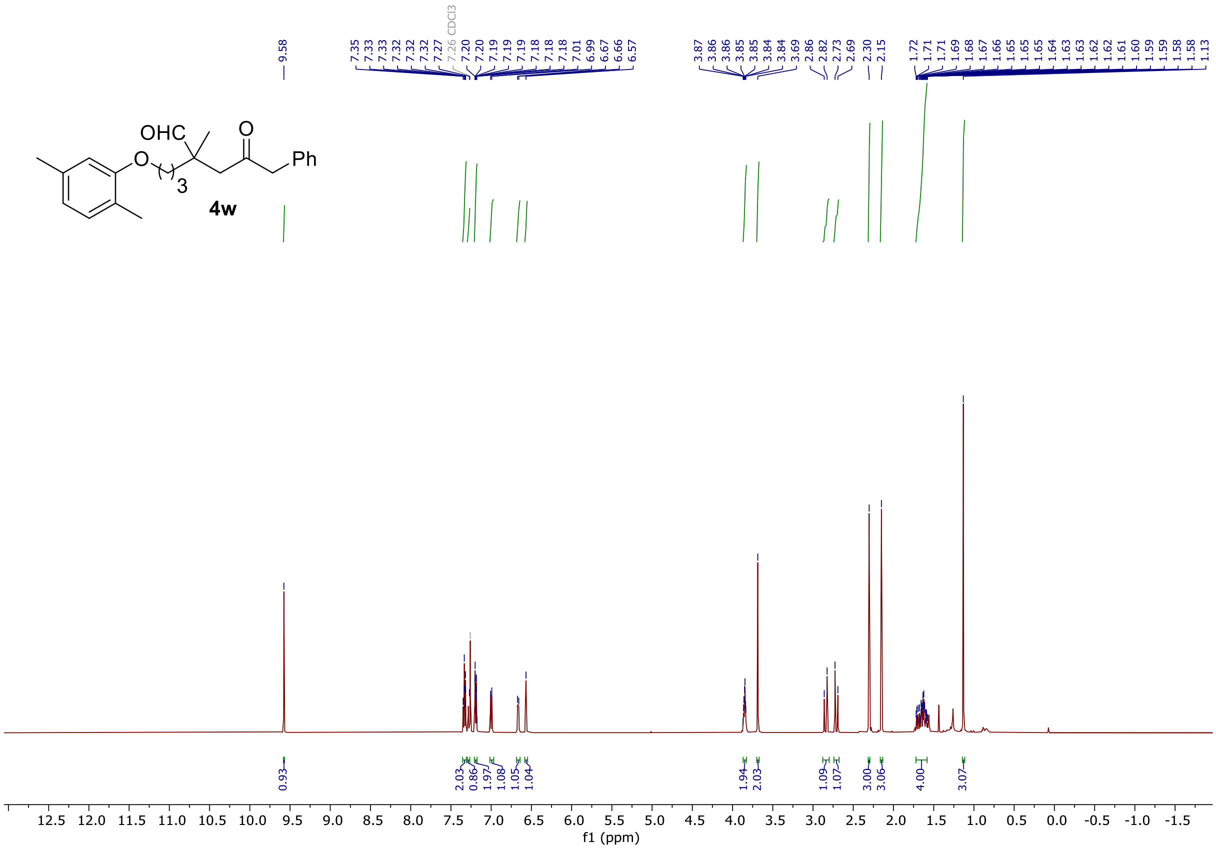
9.58
7.35 7.33 7.33 7.32 7.32 7.32 7.27 7.26 CDCl3 7.20 7.20 7.19 7.19 7.19 7.18 7.18 7.18 7.01 6.99 6.67 6.66 6.57
3.87 3.86 3.86 3.85 3.85 3.84 3.84 3.69 2.86 2.82 2.73 2.69 2.30 2.15
1.72 1.71 1.71 1.69 1.68 1.67 1.66 1.65 1.65 1.65 1.64 1.63 1.63 1.62 1.62 1.61 1.60 1.59 1.59 1.58 1.58 1.13
OHC
O
Ph
3
4w
0.93
2.03 0.86 1.97 1.08 1.05 1.04
1.94 2.03
1.09 1.07
3.00 3.06
4.00
3.07
f1 (ppm)

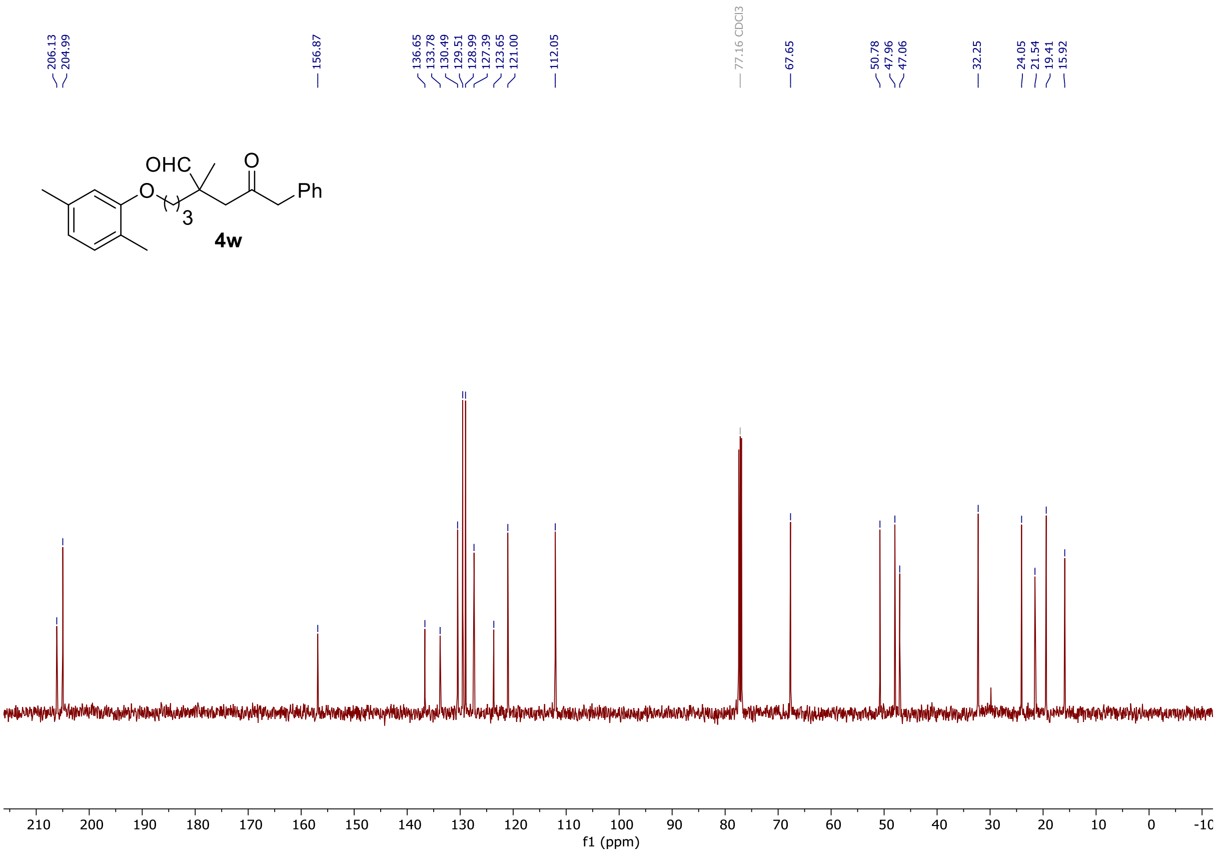
206.13 204.99
156.87
136.65 133.78 130.49 129.51 128.99 127.39 123.65 121.00
112.05
77.16 CDCl3
67.65
50.78 47.96 47.06
32.25
24.05 21.54 19.41 15.92
OHC
O
Ph
3
4w
f1 (ppm)

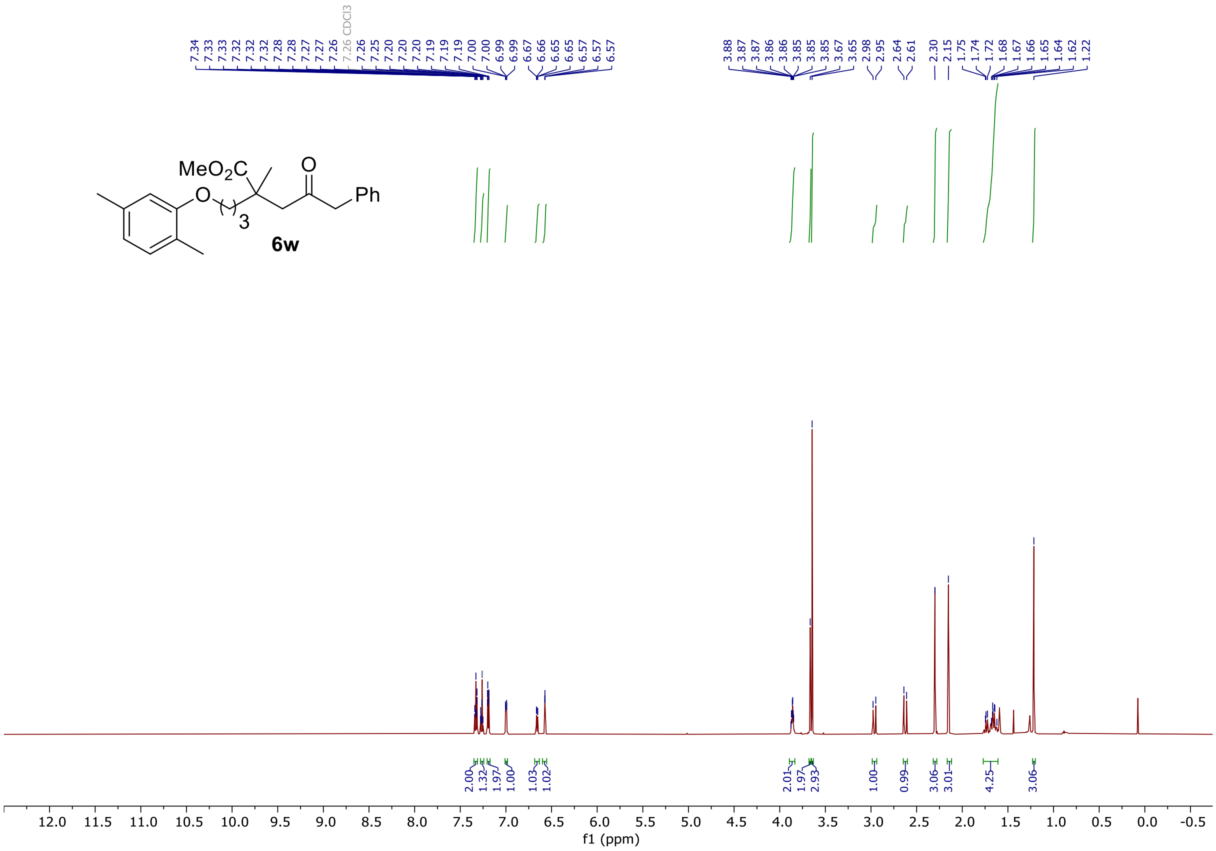
MeO2C
O
Ph
3
6w
f1 (ppm)

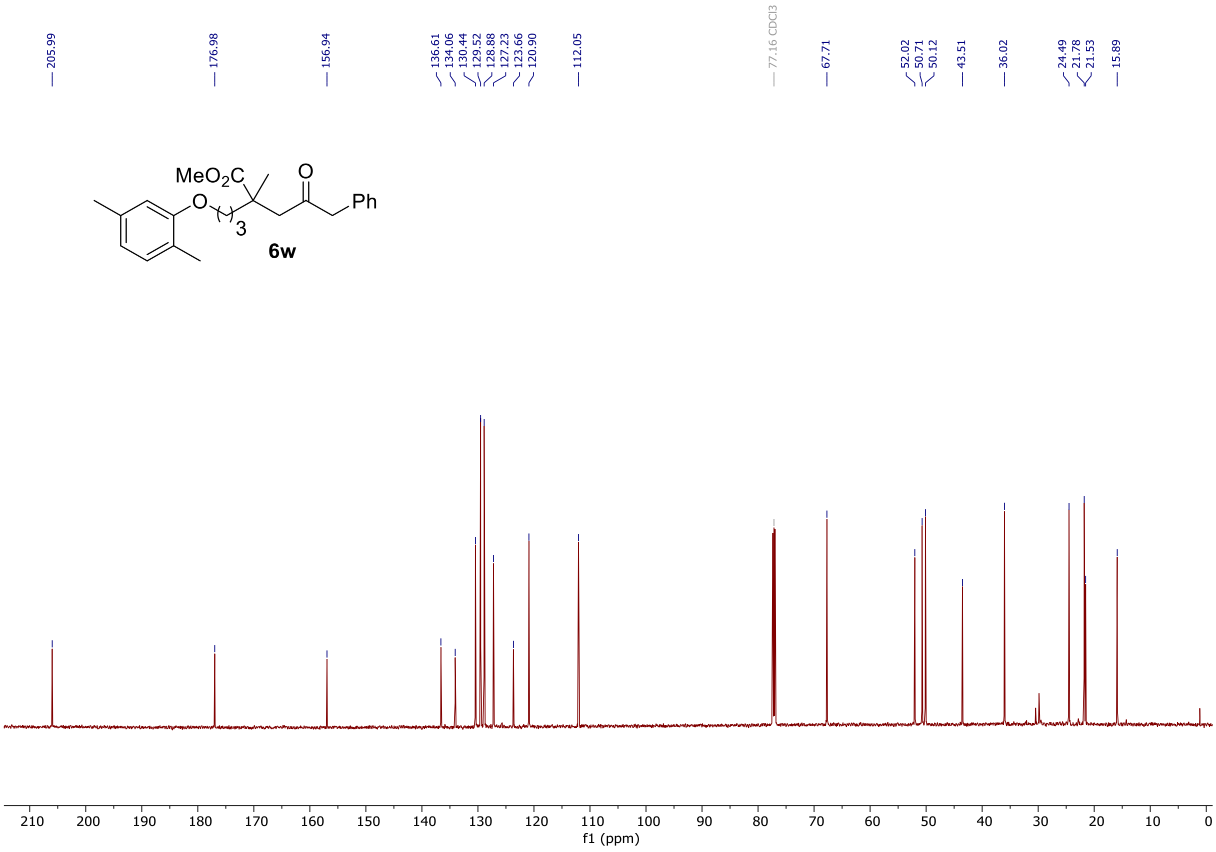
MeO2C
O
Ph
3
6w
f1 (ppm)

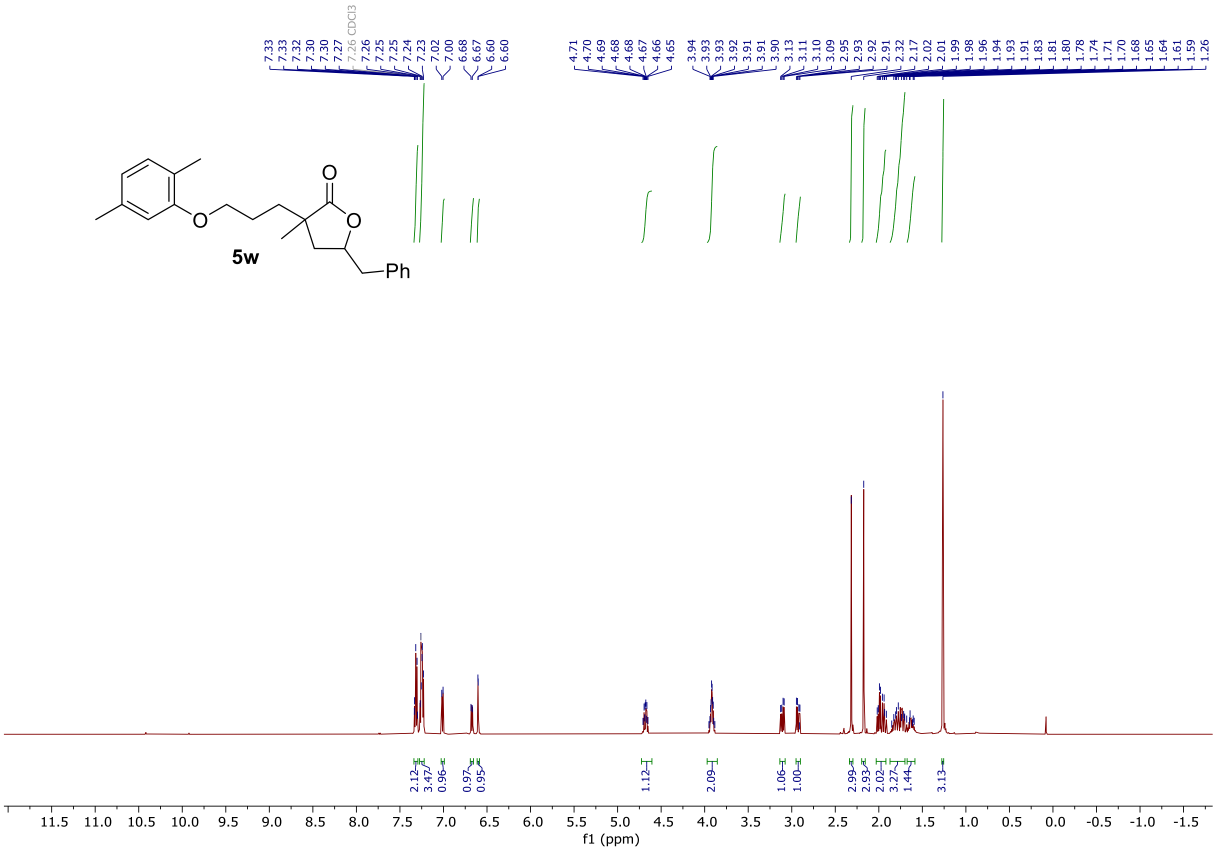
7.33
7.33
7.32
7.30
7.30
7.27
7.26 CDCl3
7.26
7.25
7.25
7.24
7.23
7.02
7.00
6.68
6.67
6.60
6.60
4.71
4.70
4.69
4.68
4.68
4.67
4.66
4.65
3.94
3.93
3.93
3.92
3.91
3.91
3.90
3.13
3.11
3.10
3.09
2.95
2.93
2.92
2.91
2.32
2.17
2.02
2.01
1.99
1.98
1.96
1.94
1.93
1.91
1.83
1.81
1.80
1.78
1.74
1.71
1.70
1.68
1.65
1.64
1.61
1.59
1.26
O
O
O
Ph
5w
2.12
3.47
0.96
0.97
0.95
1.12
2.09
1.06
1.00
2.99
2.93
2.02
3.27
1.44
3.13
11.5 11.0 10.5 10.0 9.5 9.0 8.5 8.0 7.5 7.0 6.5 6.0 5.5 5.0 4.5 4.0 3.5 3.0 2.5 2.0 1.5 1.0 0.5 0.0 -0.5 -1.0 -1.5
f1 (ppm)

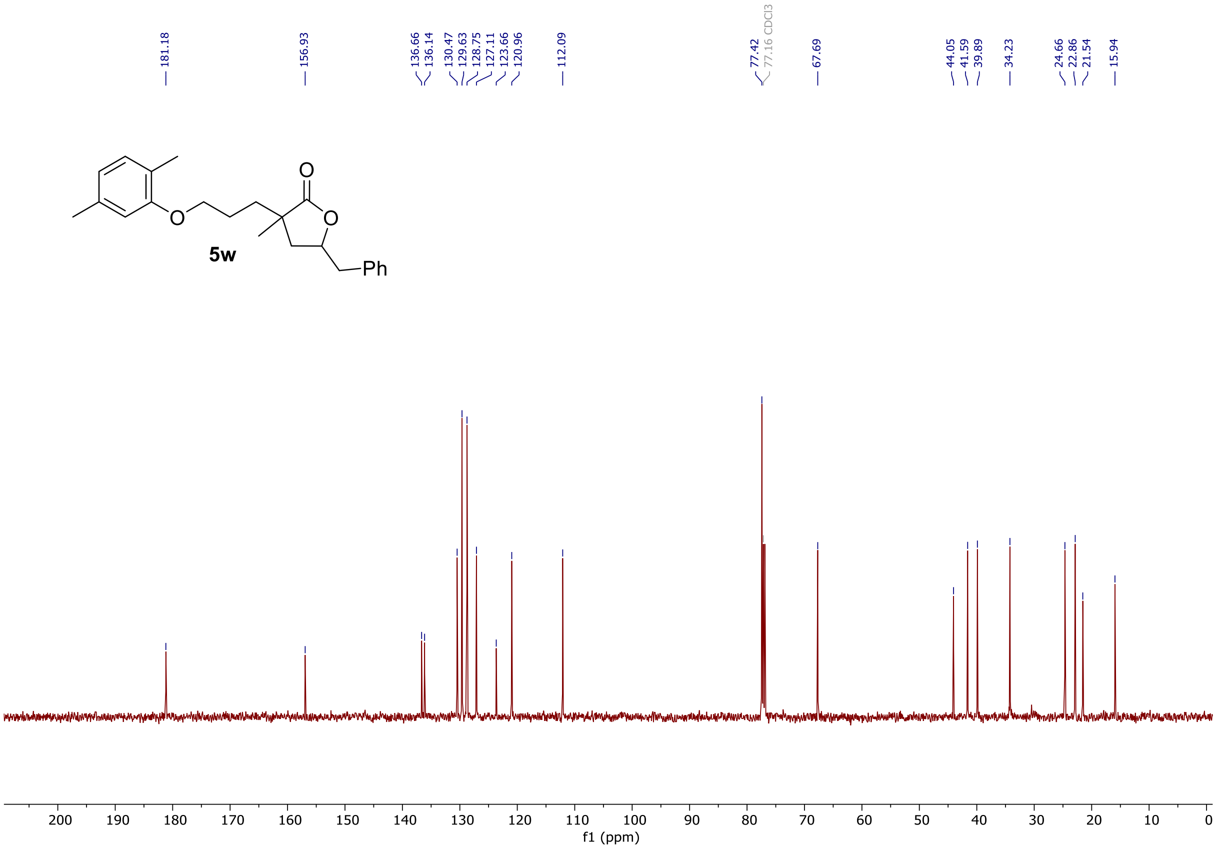
181.18
156.93
136.66
136.14
130.47
129.63
128.75
127.11
123.66
120.96
112.09
77.42
77.16 CDCl3
67.69
44.05
41.59
39.89
34.23
24.66
22.86
21.54
15.94
O
O
O
Ph
5w
200 190 180 170 160 150 140 130 120 110 100 90 80 70 60 50 40 30 20 10 0
f1 (ppm)

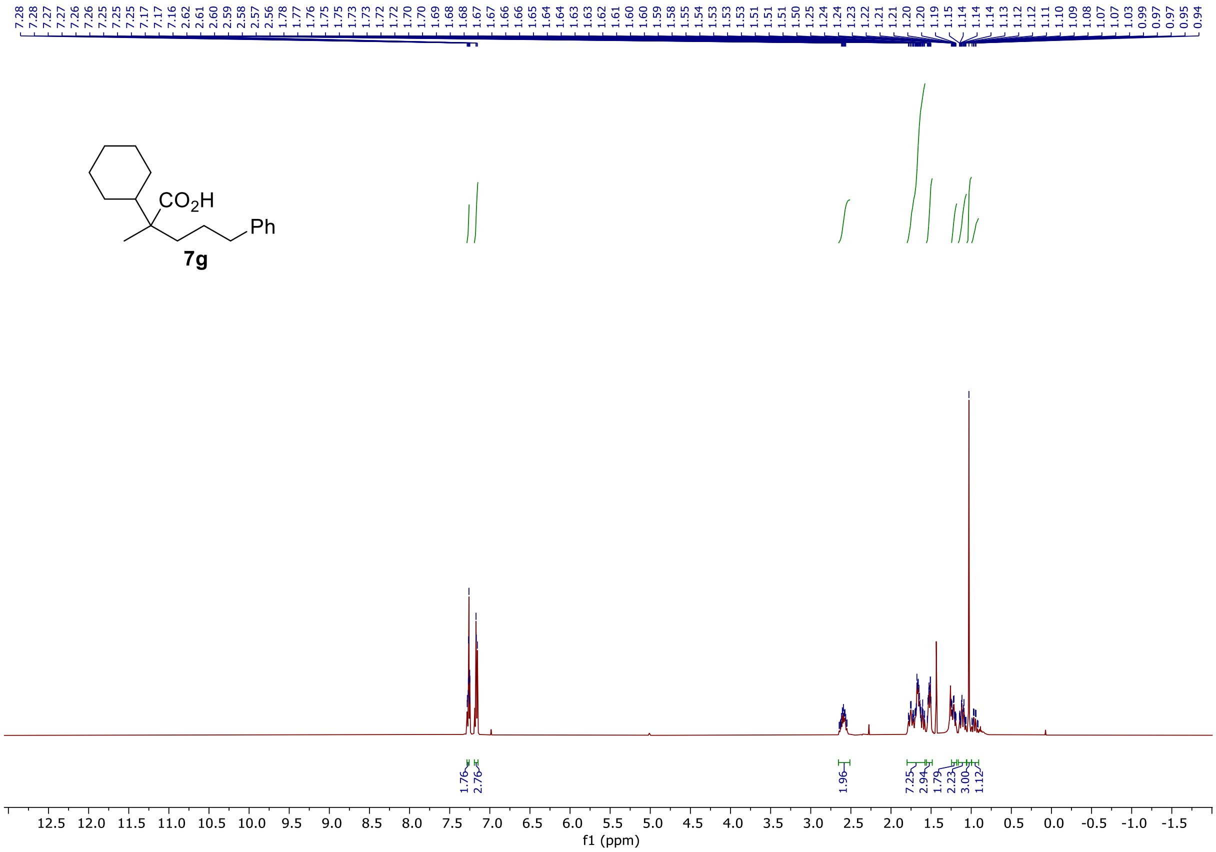
7.28 7.28 7.27 7.27 7.26 7.26 7.25 7.25 7.25 7.17 7.17 7.16 2.62 2.61 2.60 2.59 2.58 2.57 2.56 1.78 1.77 1.76 1.75 1.75 1.73 1.73 1.73 1.72 1.72 1.70 1.70 1.69 1.68 1.68 1.67 1.67 1.66 1.66 1.65 1.64 1.64 1.63 1.63 1.62 1.61 1.60 1.60 1.59 1.58 1.55 1.54 1.53 1.53 1.53 1.51 1.51 1.51 1.50 1.25 1.25 1.24 1.24 1.23 1.22 1.21 1.21 1.20 1.20 1.19 1.15 1.14 1.14 1.14 1.13 1.12 1.12 1.11 1.10 1.09 1.08 1.07 1.07 1.03 0.99 0.97 0.97 0.95 0.94
CO2H
Ph
7g
1.76 2.76 1.96 7.25 2.94 1.79 2.23 3.00 1.12
12.5 12.0 11.5 11.0 10.5 10.0 9.5 9.0 8.5 8.0 7.5 7.0 6.5 6.0 5.5 5.0 4.5 4.0 3.5 3.0 2.5 2.0 1.5 1.0 0.5 0.0 -0.5 -1.0 -1.5
f1 (ppm)

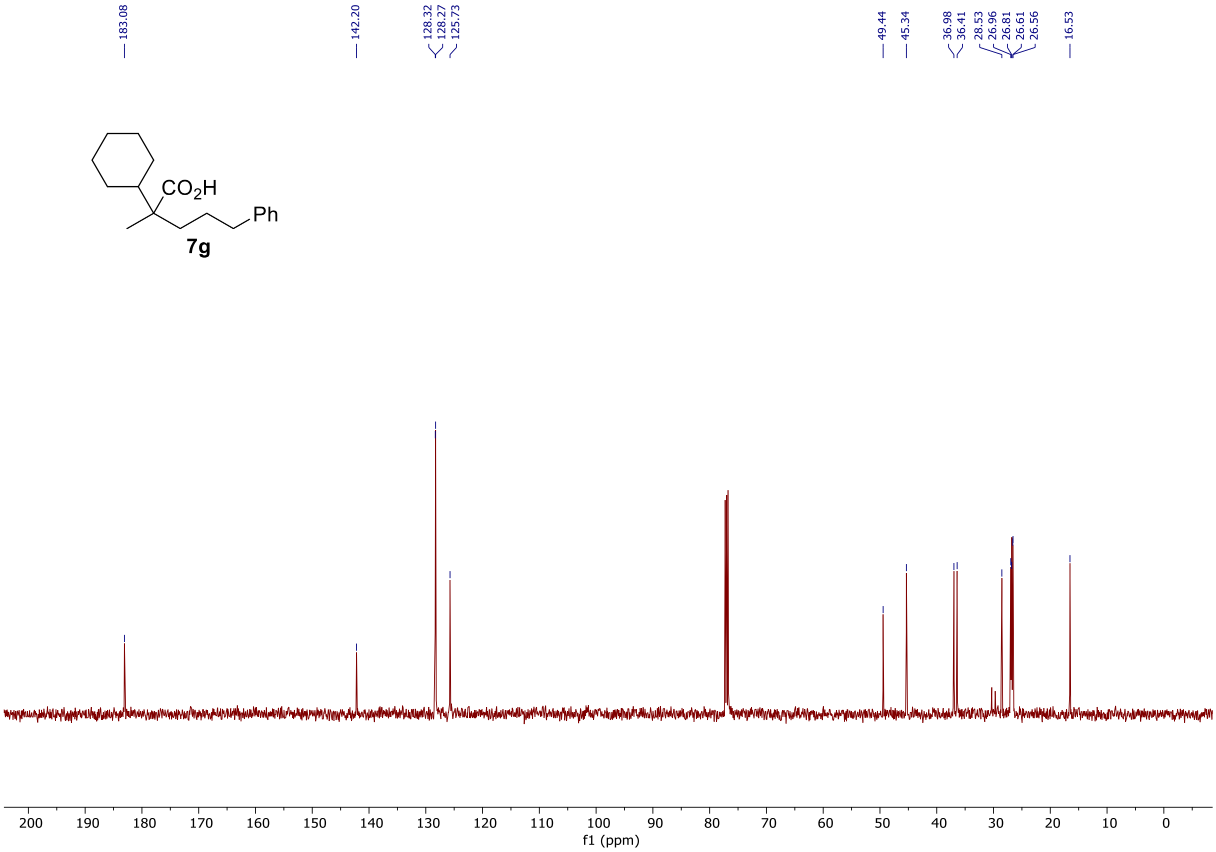
183.08
142.20
128.32 128.27 125.73
49.44
45.34
36.98 36.41 28.53 26.96 26.81 26.61 26.56
16.53
CO2H
Ph
7g
200 190 180 170 160 150 140 130 120 110 100 90 80 70 60 50 40 30 20 10 0
f1 (ppm)

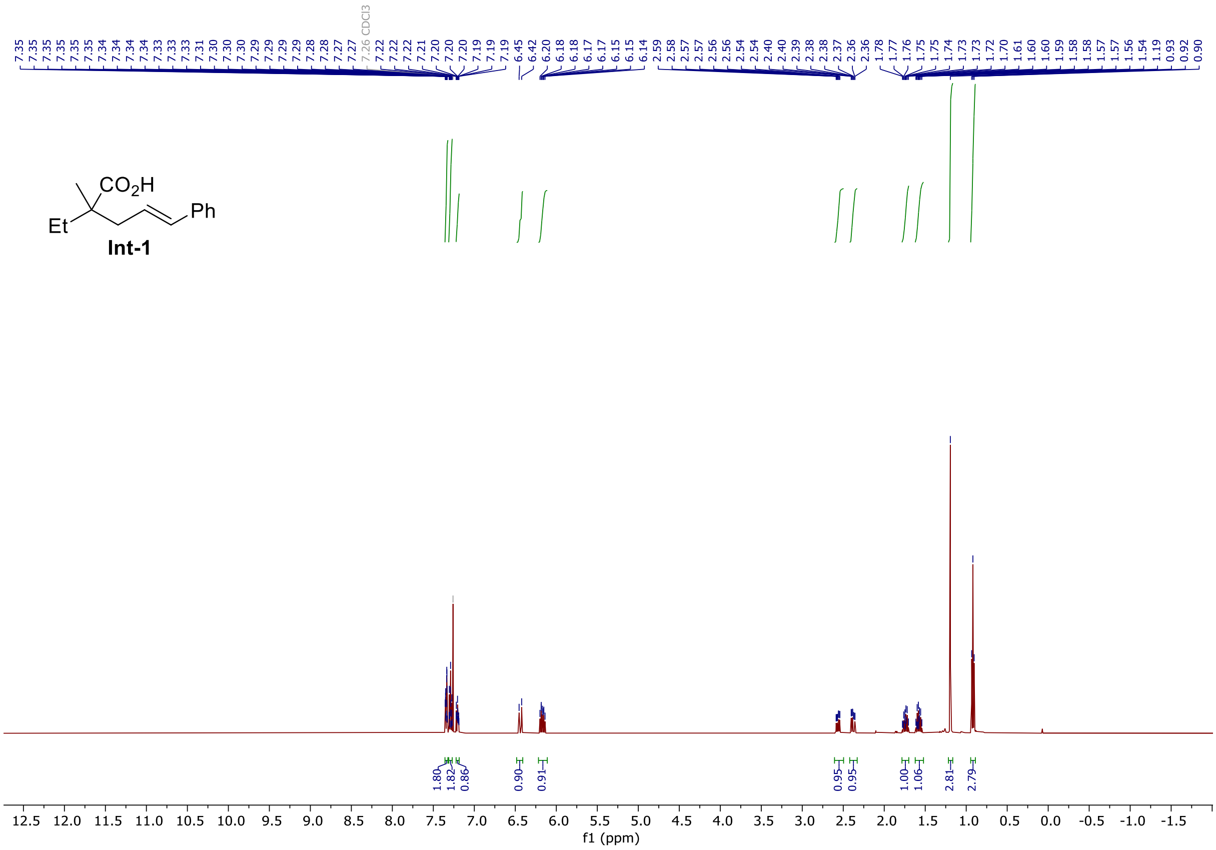
7.35 7.35 7.35 7.35 7.35 7.35 7.34 7.34 7.34 7.34 7.33 7.33 7.33 7.31 7.30 7.30 7.30 7.29 7.29 7.29 7.29 7.28 7.28 7.27 7.26 CDCl3 7.22 7.22 7.22 7.21 7.20 7.20 7.20 7.19 7.19 7.19
6.45 6.42 6.20 6.18 6.18 6.17 6.17 6.15 6.15 6.14
2.59 2.58 2.57 2.57 2.56 2.56 2.54 2.54 2.40 2.40 2.39 2.38 2.38 2.37 2.36 2.36
1.78 1.77 1.76 1.75 1.75 1.74 1.73 1.73 1.72 1.70 1.61 1.60 1.60 1.59 1.58 1.58 1.57 1.57 1.56 1.54 1.19 0.93 0.92 0.90
CO2H
Et
Ph
Int-1
1.80 1.82 0.86 0.90 0.91 0.95 0.95 1.00 1.06 2.81 2.79
12.5 12.0 11.5 11.0 10.5 10.0 9.5 9.0 8.5 8.0 7.5 7.0 6.5 6.0 5.5 5.0 4.5 4.0 3.5 3.0 2.5 2.0 1.5 1.0 0.5 0.0 -0.5 -1.0 -1.5
f1 (ppm)

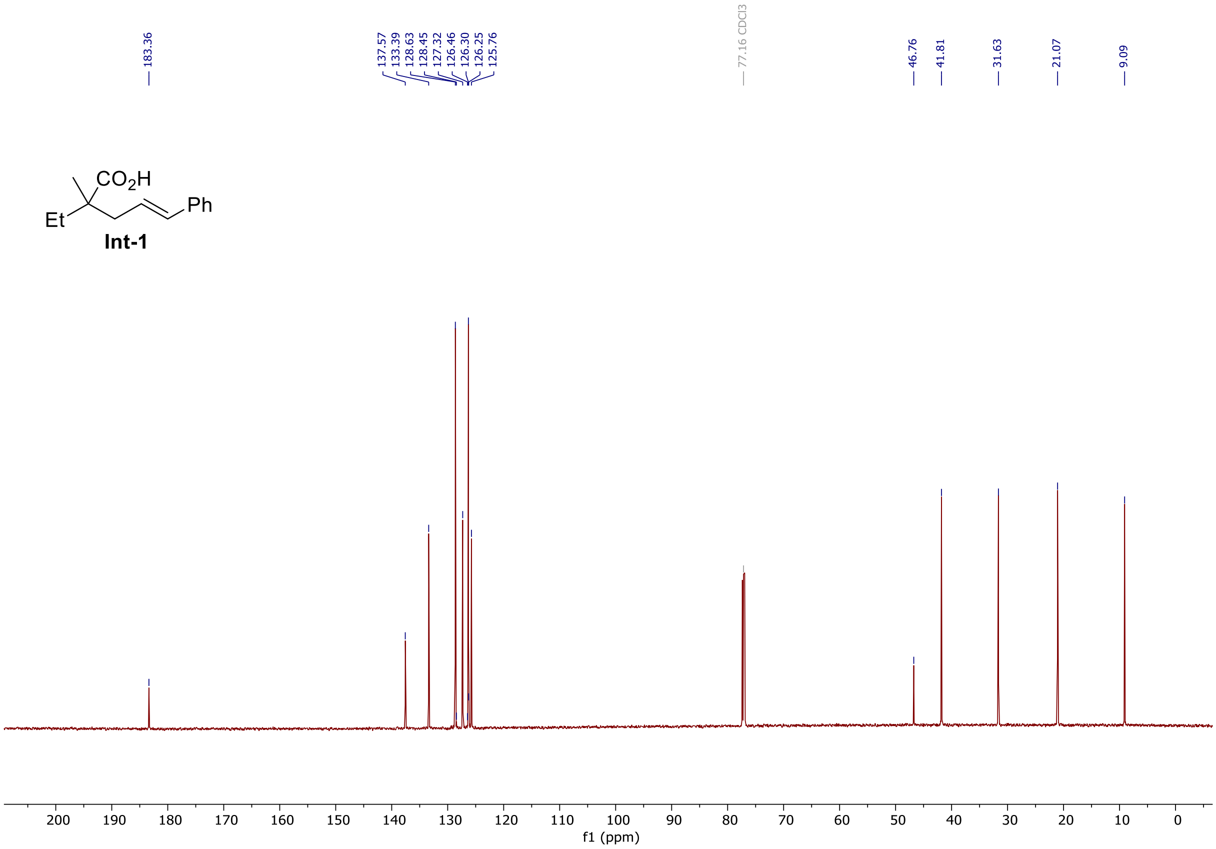
183.36
137.57 133.39 128.63 128.45 127.32 126.46 126.30 126.25 125.76
77.16 CDCl3
46.76 41.81 31.63 21.07 9.09
CO2H
Et
Ph
Int-1
200 190 180 170 160 150 140 130 120 110 100 90 80 70 60 50 40 30 20 10 0
f1 (ppm)

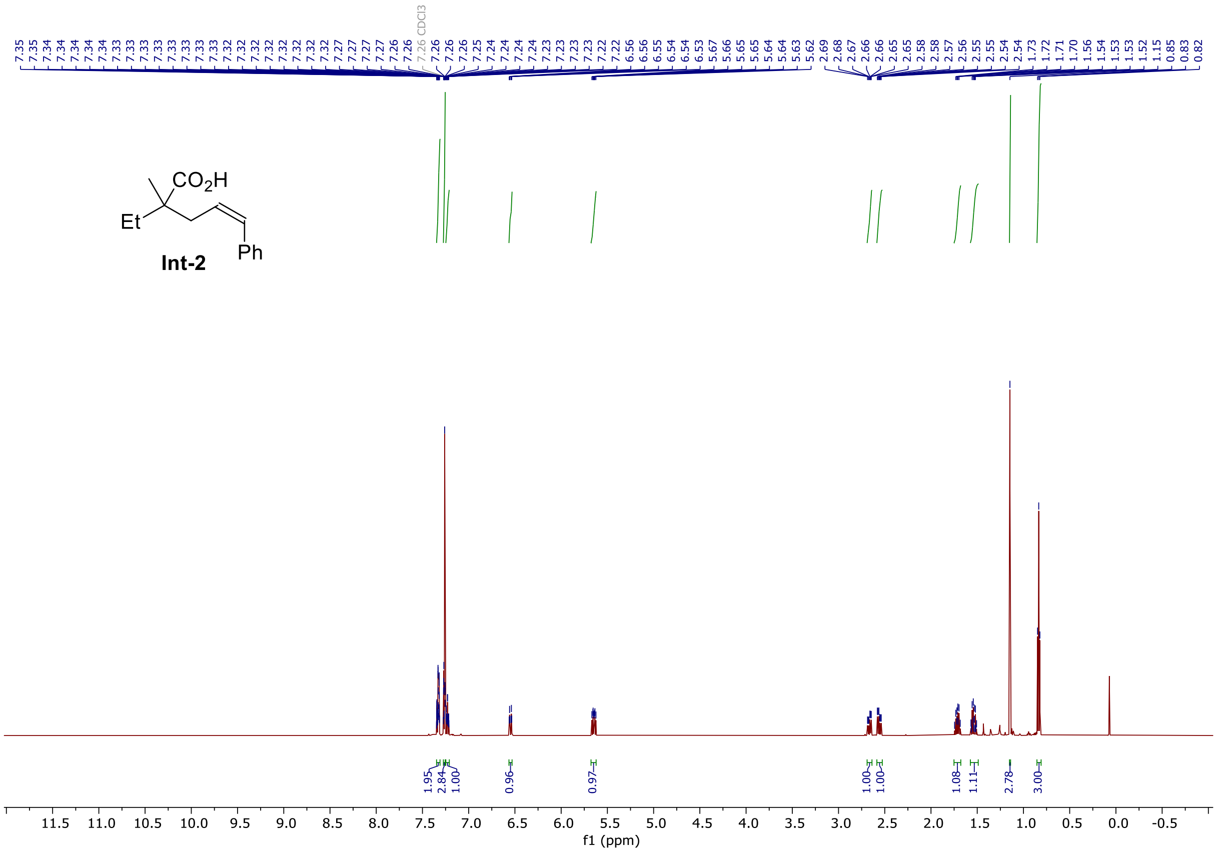
7.35 7.35 7.34 7.34 7.34 7.34 7.33 7.33 7.33 7.33 7.33 7.33 7.33 7.32 7.32 7.32 7.32 7.32 7.32 7.32 7.32 7.27 7.27 7.27 7.26 7.26 7.26 CDCl3 7.26 7.26 7.25 7.24 7.24 7.24 7.24 7.23 7.23 7.23 7.23 7.22 7.22 6.56 6.56 6.55 6.54 6.54 6.53 5.67 5.66 5.65 5.65 5.64 5.63 5.63 5.62 2.69 2.68 2.67 2.66 2.66 2.65 2.65 2.58 2.58 2.57 2.56 2.55 2.55 2.54 2.54 1.73 1.72 1.71 1.70 1.56 1.54 1.53 1.53 1.53 1.52 1.15 0.85 0.83 0.82
CO2H
Et
Ph
Int-2
1.95 2.84 1.00 0.96 0.97 1.00 1.00 1.08 1.11 2.78 3.00
11.5 11.0 10.5 10.0 9.5 9.0 8.5 8.0 7.5 7.0 6.5 6.0 5.5 5.0 4.5 4.0 3.5 3.0 2.5 2.0 1.5 1.0 0.5 0.0 -0.5
f1 (ppm)

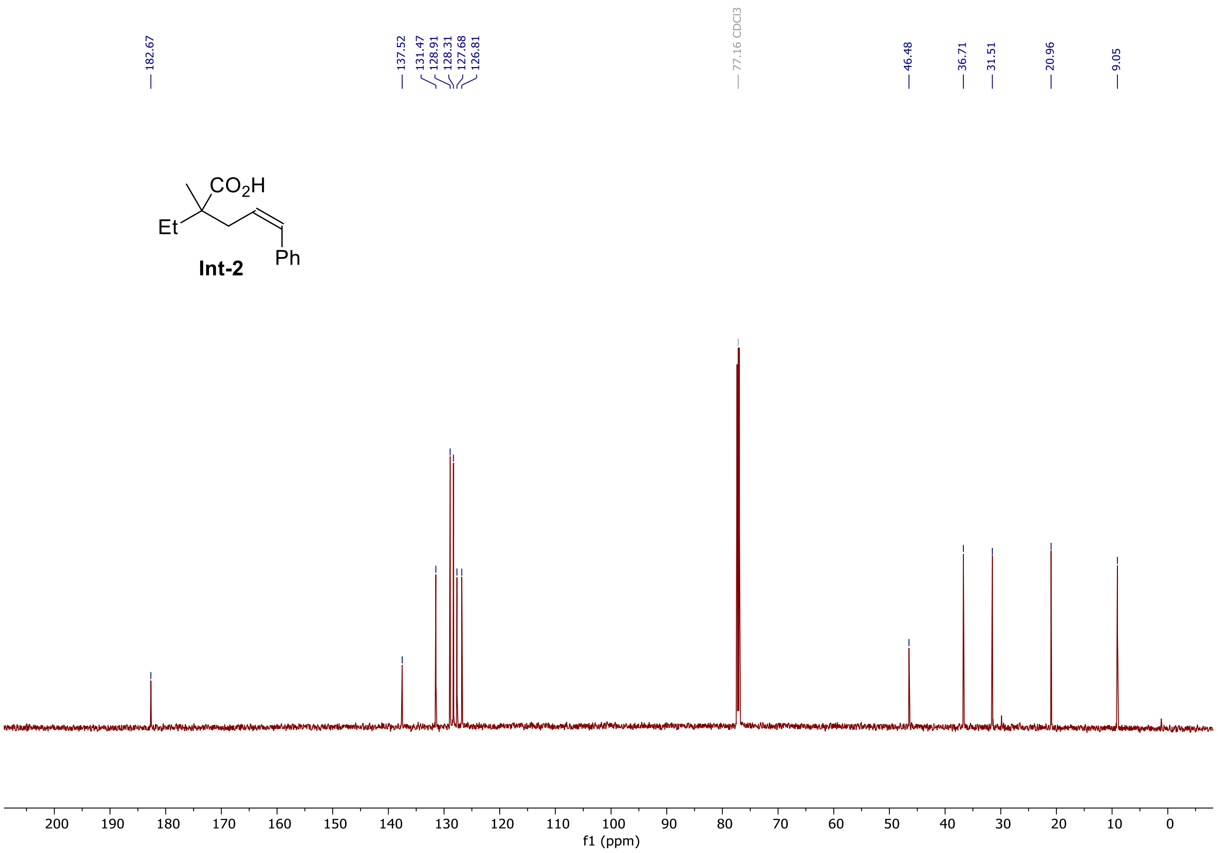
182.67
137.52 131.47 128.91 128.31 127.68 126.81
77.16 CDCl3
46.48
36.71
31.51
20.96
9.05
CO2H
Et
Ph
Int-2
200 190 180 170 160 150 140 130 120 110 100 90 80 70 60 50 40 30 20 10 0
f1 (ppm)
